# Translating a heart disease lifestyle intervention into the community: the South Asian Heart Lifestyle Intervention (SAHELI) study; a randomized control trial

**DOI:** 10.1186/s12889-015-2401-2

**Published:** 2015-10-16

**Authors:** Namratha R. Kandula, Swapna Dave, Peter John De Chavez, Himali Bharucha, Yasin Patel, Paola Seguil, Santosh Kumar, David W. Baker, Bonnie Spring, Juned Siddique

**Affiliations:** Department of General Internal Medicine, Northwestern University Feinberg School of Medicine, 750 N Lake Shore Drive, 10th Floor, Chicago, IL 60611 USA; Metropolitan Asian Family Services, 7541 N Western Avenue, Chicago, IL 60645 USA; Department of Preventive Medicine, Northwestern University, 680 N Lake Shore Drive, Suite 1400, Chicago, IL 60611 USA; National Heart, Lung, and Blood Institute (NHLBI), National Institutes of Health (NIH), Bethesda, MD USA

**Keywords:** Cardiovascular disease prevention, South Asian, Randomized clinical trial

## Abstract

**Background:**

South Asians (Asian Indians and Pakistanis) are the second fastest growing ethnic group in the United States (U.S.) and have an increased risk of atherosclerotic cardiovascular disease (ASCVD). This pilot study evaluated a culturally-salient, community-based healthy lifestyle intervention to reduce ASCVD risk among South Asians.

**Methods:**

Through an academic-community partnership, medically underserved South Asian immigrants at risk for ASCVD were randomized into the South Asian Heart Lifestyle Intervention (SAHELI) study. The intervention group attended 6 interactive group classes focused on increasing physical activity, healthful diet, weight, and stress management. They also received follow-up telephone support calls. The control group received translated print education materials about ASCVD and healthy behaviors. Primary outcomes were feasibility and initial efficacy, measured as change in moderate/vigorous physical activity and dietary saturated fat intake at 3- and 6-months. Secondary clinical and psychosocial outcomes were also measured.

**Results:**

Participants’ (*n* = 63) average age was 50 (SD = 8) years, 63 % were female, 27 % had less than or equal to a high school education, one-third were limited English proficient, and mean BMI was 30 kg/m2 (SD ± 5). There were no significant differences in change in physical activity or saturated fat intake between the intervention and control group. Compared to the control group, the intervention group showed significant weight loss (−1.5 kg, *p*-value = 0.04) and had a greater sex-adjusted decrease in hemoglobin A1C (−0.43 %, *p*-value <0.01) at 6 months. Study retention was 100 %.

**Conclusions:**

This pilot study suggests that a culturally-salient, community-based lifestyle intervention was feasible for engaging medically underserved South Asian immigrants and more effective at addressing ASCVD risk factors than print health education materials.

**Trial registration:**

NCT01647438, Date of Trial Registration: July 19, 2012

**Electronic supplementary material:**

The online version of this article (doi:10.1186/s12889-015-2401-2) contains supplementary material, which is available to authorized users.

## Background

Diet and physical activity behavioral interventions in persons with risk factors for atherosclerotic cardiovascular disease (ASCVD) have resulted in long-term improvements across important intermediate health outcomes (weight, blood pressure, cholesterol, and glucose) [1–5]. Individuals of South Asian-origin (India, Pakistan, Bangladesh, Sri Lanka, Nepal, Bhutan) are the second fastest growing ethnic group in the United States (U.S.); [6] South Asians have an elevated risk of early and aggressive ASCVD compared to other racial/ethnic groups [7–10]. Although genetics may play a role [11], poor diet, physical inactivity, and overweight/obesity still remain the major lifestyle risk factors in South Asians [12, 13]. In the U.S., South Asians are less physically active and have a higher prevalence of overweight/obesity than other Asian American groups [13, 14]. Importantly, South Asians develop ASCVD risk factors even with small amounts of weight gain [15], and thus, lifestyle interventions could substantially reduce their ASCVD risk.

Although diet and physical activity counseling is recommended for all individuals with ASCVD risk factors [16], few lifestyle interventions exist for the 3.4 million South Asians living in the U.S. The majority of evidence-based lifestyle interventions have been developed and tested in predominantly White or African American populations [17]. Research suggests that there may be differential effectiveness of diet and physical activity interventions in South Asian migrant populations compared to other ethnicities [18]. In addition, our prior research found that existing lifestyle interventions were not reaching South Asians because interventions did not address the sociocultural determinants of diet, physical activity, weight, and health [19–25]. Translating evidence-based lifestyle interventions across an increasingly diverse U.S. population and with underserved populations is a major challenge [26].

Given the paucity of clinical trial evidence on the effectiveness of lifestyle interventions in the South Asian population [18], this pilot study was designed to test the feasibility and initial efficacy of the South Asian Heart Lifestyle Intervention (SAHELI), a culturally-tailored group lifestyle intervention for medically underserved South Asians at-risk for ASCVD. The few lifestyle intervention trials that have been conducted in adult South Asian migrants have been from Europe [27–29], and almost none from the U.S [30]. To the best of our knowledge, the proposed study is one of the first to implement and pilot-test a culturally-salient, evidence-based behavioral lifestyle intervention to improve ASCVD risk factors in U.S. South Asian immigrants.

## Methods

### Primary research goals

The primary research goals were to pilot-test SAHELI, via a 2-arm randomized design, and examine its feasibility and initial efficacy to improve moderate/vigorous physical activity and saturated fat intake among South Asians in a community-based setting. Secondary outcomes included clinical risk factors (weight, waist circumference, blood sugar, hemoglobin A1c, lipids, and blood pressure) and psychosocial outcomes. The study protocol is available, see Additional file 1.

### Study framework

This study used a community-based participatory research (CBPR) framework. The community partner (Metropolitan Asian Family Services) and academic partner (Northwestern University) collaboratively chose the focus of this research because it was relevant and responsive to the needs of this South Asian community [31]. The partners had previously worked together on formative research to understand community needs and the socio-cultural context of South Asian ASCVD risk behaviors [19–25]. The study partners developed a memorandum of understanding, engaged in formal capacity building activities (e.g., trainings on human subjects research, study protocols, quality control and translation of study documents for populations with lower literacy), and formed a community advisory board to provide structure and oversight of the partnership and research study. The study partners and community advisory board were involved in study design, review of study materials and questionnaires to ensure cultural equivalence, implementation, recruitment and retention of participants, and evaluation. The study protocol and procedures were approved by the Institutional Review Board at Northwestern University.

### Participants and setting

The pilot study design and methods have been previously described in detail [32]. The term South Asian is used to group together individuals from India, Pakistan, Bangladesh, Sri Lanka, Nepal, and Bhutan [6]. Participants in this study were mainly from India and Pakistan. Indians and Pakistanis comprise 90 % of South Asians in the U.S. and in the Rogers Park neighborhood of Chicago, IL [6, 16], where this study took place. Rogers Park is a major entry hub for South Asian immigrants [16], many of whom are medically underserved, meaning that they face economic, linguistic, and cultural barriers to health care [33]. In 2008, the research team and community partners conducted a survey to assess the health needs of the local South Asian community and to guide planning of the intervention. In this convenience sample, 85 % of South Asians in Rogers Park were overweight/obese and 81 % had at least one ASCVD risk factor [21].

The study was conducted at Metropolitan Asian Family Services, a not-for-profit, community-based organization that provides comprehensive and integrated social services to immigrants. The organization serves about 1300 South Asian families, and all fall below 100 % of the federal poverty level.

### Eligibility

This study included individuals who self-identified as Asian Indian or Pakistani, were between 30 and 59 years, and had at least one ASCVD risk factor. ASCVD risk factors included: obesity (body mass index > 25 kg/m2- which is the cutoff for obesity in SA) [15], hypertension (Systolic blood pressure ≥140 or diastolic blood pressure ≥ 90) [34], hyperlipidemia (Total cholesterol ≥200 or LDL cholesterol ≥130) [35], pre-diabetes (Fasting plasma glucose >100 or Hemoglobin A1c between 5.7 and 6.4 %) [36], or diabetes (FPG > 126, Hemoglobin A1c >6.4 or on diabetes medications) [36]. Living in the same household was an exclusion criteria; however relatives from different households were not excluded.

### Randomization and masking

All eligible participants were randomized into one of the groups by a computer-generated list which was maintained at the academic site. Participants were stratified by gender in equal numbers. After randomization, participants in the intervention group were assigned to group classes based on language preference. Complete masking of participants and the study team was not possible. To help mitigate bias, baseline data collection occurred prior to randomization and each study group was assigned a non-revealing label for use on study documents. Investigators, research staff, and CBO staff were not blinded to the study hypothesis. Study participants were blinded to the study hypothesis. Because the community-based organization and community advisory board were concerned about the ethics of randomizing participants to a control group, the community advisory board modified the study protocol such that the control group received the intervention classes once the final 6-month assessments were completed.

### Intervention design

Using mixed-methods community-engaged research, the study team developed the South Asian Heart Lifestyle Intervention (SAHELI), a group lifestyle intervention that integrated evidence-based behavior change strategies with South Asians’ sociocultural context and beliefs. SAHELI was a 16-week lifestyle intervention that included group classes, experiential activities, behavior change counseling, and telephone support. The cultural adaptation of the intervention has been described in detail previously [20, 32].

Group classes were held weekly for 6 weeks and lasted between 60 and 90 min. Each class covered a different topic (#1: What is Heart Disease and Understanding Your Risk Factors; #2: How to Get More Exercise; #3: Eat Less Fat and Salt; #4: Enjoy Fruits, Vegetables, & Grains; #5: Maintain a Healthy Weight; #6: Taking Care of Stress and Tension). Classes taught participants goal-setting techniques for creating and maintaining specific, measurable, and realistic goals [37] with attention to physical activity, diet, weight, and stress management. Participants were taught about national recommendations for physical activity (e.g. 150 min of moderate intensity physical activity per week in bouts that are at least 10 min in duration) and were given pedometers. They were taught how to self-monitor daily steps and how to gradually increase activity. Diet sessions reviewed dietary recommendations (e.g. 7 servings of fruits and vegetables per day) and included experiential activities, such as meal planning, portion control, and a trip to a South Asian grocery store. Participants were encouraged to set realistic behavior change goals based on their current behaviors.

**Fig. 1:**
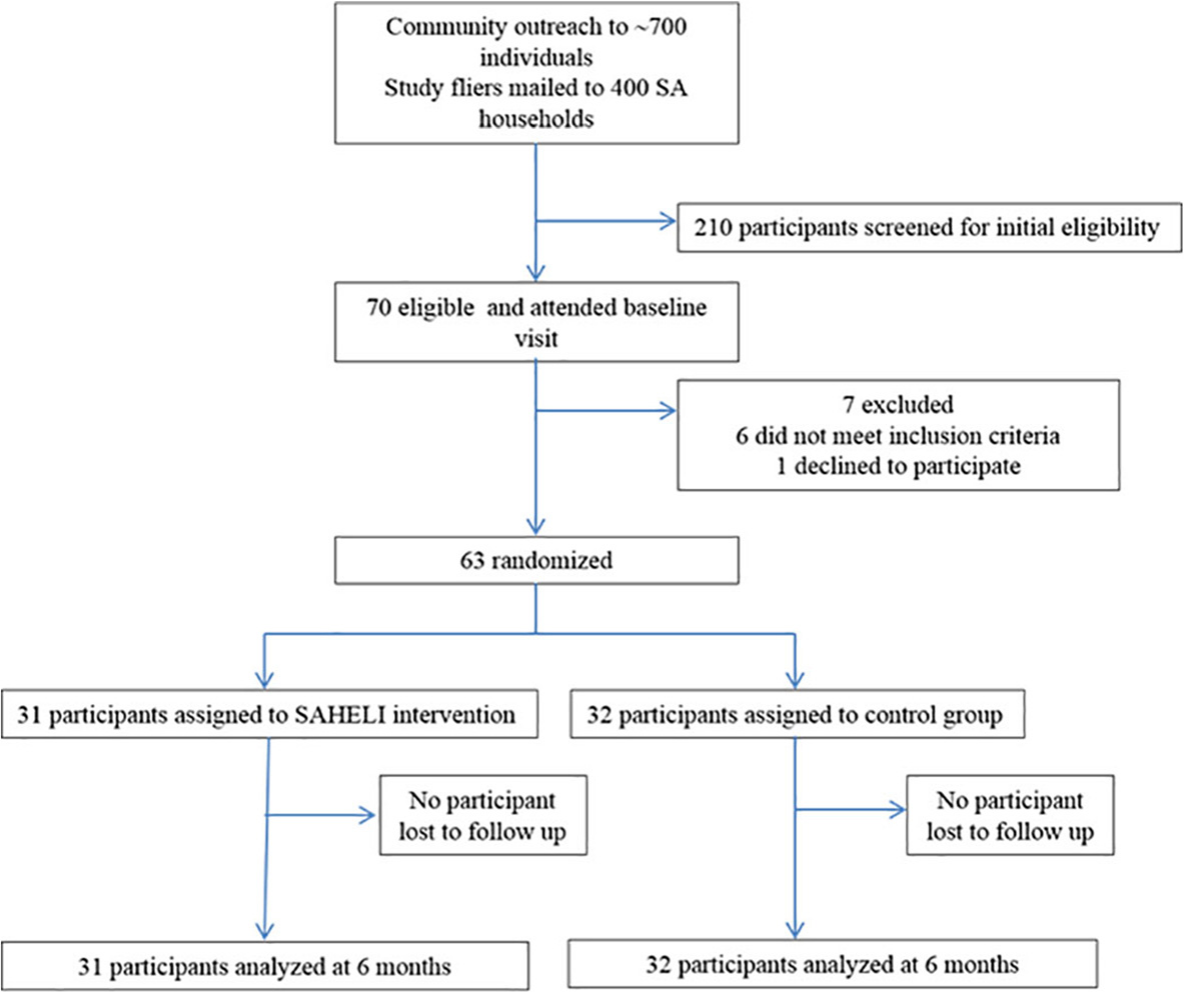
Flow of participants from outreach to completion, South Asian Heart Lifestyle Intervention, Chicago, IL, June 2012-November 2013

Individual telephone support started after classes ended and continued for 10 weeks (Fig. 1). Phone counseling used a motivational interviewing framework to focus on self-reflection, behavior goals, and problem solving [38]. Calls followed a semi-structured script, were digitally-recorded, and systematically tracked.

As part of SAHELI, the study team also conducted 4 heart health *melas* over the course of 12 months. *Mela* means ‘to meet’ in Hindi and Urdu. *Melas* are festive gatherings that can be religious, cultural, or social. Those held for the study were open to intervention participants and their families and were held at the CBO or nearby Park District locations. The *melas* incorporated culturally-salient activities which were designed to reinforce healthy behaviors and increase group cohesion and support [39]. Activities included yoga, healthy cooking with a South Asian chef, aerobic exercise that built on South Asian folk dance and competitions with prizes (e.g. who can make the tastiest whole grain dish and salad).

### Intervention and control arms

All participants, in the intervention and control arms, were given their test results and told to follow-up with a health care provider after receiving their test results. The intervention participants received the group classes, telephone counseling and heart health *melas*. Participants in the control arm were mailed their test results, a brief explanation of results, and translated, pre-existing print education materials about ASCVD, diet, exercise, and weight loss.

### Study measurements

We assessed all outcomes at baseline and at 3- and 6-month follow-ups (Fig. 1). At baseline, we obtained demographic information and a brief medical history. Trained, bilingual research assistants administered questionnaires and performed clinical and anthropometric measurements using standardized protocols.

Physical activity was assessed using accelerometers. Accelerometers (Actigraph, model 7164) were worn by study participants at baseline, 3- and 6-month assessments for 7 consecutive days. We examined change in bout-corrected moderate-vigorous physical activity [40]. For comparison with national physical activity recommendations, 10-min activity bouts were defined as 10 or more consecutive minutes above the relevant threshold, with allowance for interruptions of 1 or 2 min below threshold. This is referred to as a modified 10-min bout. All models for bout-corrected PA controlled for accelerometer wear-time and weekends. Total caloric intake, percent kilocalories from saturated fat, and other dietary intake data was collected using three sets of 24-h food recalls at baseline, 3- and 6-months, including one in-person recall and two phone recalls (including one weekend day). Dietary data were analyzed using Nutrition Data System for Research (Version 2011, Nutrition Coordinating Center (NCC), University of Minnesota, Minneapolis, MN.) Participants underwent a fasting blood draw for hemoglobin A1C, plasma glucose and serum cholesterol at each assessment. We used established questionnaires to measure exercise-related confidence [41] and coping [42]. All study questionnaires were translated into Hindi. Participants received a $25 gift card after returning the accelerometers at baseline, 3-, and 6 month assessments.

### Statistical analysis

The analytic plan was designed to compare SAHELI to the control arm on feasibility and initial efficacy 3- and 6-months following randomization. Data were analyzed on an intent to treat basis. Retention rate was calculated as the total study retention rate and the retention rate for each study arm. Program Adherence was measured as class attendance and completion of follow-up phone calls. We compared retention rates between the 2 arms using a Chi-square test or Fisher’s exact test. To test for differences in physical activity and % kilocalories from saturated fat by treatment group at 3- and 6-months, we used mixed-effects models for longitudinal data [43]. Specifically, treating time as a nominal variable, we examined changes across time as well as the treatment (SAHELI vs control arm) by time interaction to test whether change in outcomes over time differed by treatment condition. To provide a flexible fit to the data, our models assumed an unstructured residual covariance matrix. All models controlled for sex. Models for the analysis of bout-corrected moderate-vigorous physical activity controlled for accelerometer wear time and weekend day. Changes in clinical and psychosocial outcomes were also compared by treatment group at 3- and 6- months. Analyses mirrored those described for physical activity and diet.

### Power

In terms of power for longitudinal designs, we used formulas [44] for comparing SAHELI versus the control group for the efficacy outcomes of physical activity and saturated fat intake. Sample size estimates for this pilot study assumed equal allocation to experimental groups, intra-class correlation between repeated measures of 0.5, 90 % retention at 6 months, 80 % power, and 2-tailed α equal to .05. For physical activity, we assumed a standard deviation of 91 min of weekly moderate activity [45]. For saturated fat, we assumed a standard deviation of 4.3 % for daily % of kilocalories from saturated fat [46]. Based on these assumptions and given the final study sample size of 63, the minimum detectable difference between treatment groups was 60 min of weekly moderate/vigorous activity and 2.8 daily % kilocalories from saturated fat.

## Results

The community-based organization staff and community advisory board distributed study fliers to approximately 700 individuals at community events and community sites. Study fliers were also mailed to 400 South Asian households who were on the community-based organization’s mailing list. Interested participants were asked to call the community organization staff. In addition, the community-based organization’s study staff called any household that received a study mailing to determine if there was an interested participant in the household. Only one individual per household was invited to participate. Two-hundred and ten potential participants were screened for initial eligibility by telephone. Of the 112 participants who met initial eligibility, 70 were still interested after the telephone screening and attended a baseline study visit at the community organization. After the baseline visit results were reviewed, 64 were found to meet the eligibility criteria. One person declined further participation. The remaining 63 individuals were randomly assigned to SAHELI or to the control group between October 2012 and July 2013, with follow-up assessments occurring from December 2012 to November 2013 (Fig. 1).

Overall, participants average age was 50 (SD *±* 8) years, 63 % were female, 27 % had less than or equal to a high school education, and one-third had limited English proficiency (Table 1). At baseline, mean BMI was 30 kg/m2 (SD *±* 4.9), hemoglobin A1c 6.3 % (SD *±*1.2), total cholesterol 185 mg/dl (SD *±* 32), and systolic blood pressure 129 mmHg (SD ± 17). Accelerometer data showed that participants were not meeting national physical activity recommendations at baseline.

**Table 1:** Baseline participant characteristics in the SAHELI study, Chicago, Il, October 2012-November 2013

	Intervention	Control	*P*-value
	(*n* = 31)	(*n* = 32)	
Male, %	35 %	38 %	0.87
Age, y (SD)	50 (8)	50 (7)	0.92
Born outside the United States, (%)	100 %	100 %	--
Years in the United States, mean (SD)	10 (10)	12 (10)	0.58
Educational achievement: Less than high school, %	26 %	28 %	0.84
Limited English proficiency, %	19 %	53 %	<0.01
Trouble understanding health information, (%)	65 %	55 %	0.44
Minutes of moderate vigorous physical activity per week, mean (SD)^a^	191 (188)	141 (141)	0.14
Minutes of bout-corrected moderate vigorous physical activity per week, mean (SD)^a^	56 (114)	44 (105)	0.50
% Kilocalories from saturated fat, mean (SD)	7.7 (1.9)	7.9 (2.5)	0.77
Weight, kg (SD)	72.6 (10.9)	77.1 (14.5)	0.18
Waist circumference, cm (SD)	95 (13)	98 (11)	0.32
BMI, kg/m^2^ (SD)	29 (5)	30 (5)	0.43
Hemoglobin A1c %	6.2 (0.9)	6.4 (1.5)	0.72
Fasting glucose, mg/dl (SD)	109 (21)	114 (44)	0.57
Total cholesterol, mg/dl (SD)	185 (32)	185 (32)	0.99
Systolic blood pressure, mm HG (SD)	127 (17)	130 (17)	0.39
Minutes of sedentary per week, mean (SD)^a^	3374 (689)	3495 (775)	0.42
Minutes of total accelerometer wear time per week, mean (SD)	6062 (1376)	5970 (1268)	0.65
Kilocalories per day, mean (SD)	1167 (424)	1254 (567)	0.49
Fruit and vegetable servings per day, mean (SD)	3 (2)	2 (1)	0.16
Current tobacco use, (%)	0 %	13 %	0.11
Coping, mean (SD), range (1–5)^b^	3.4 (1.1)	3.5 (1.1)	0.64
Exercise-related confidence, mean (SD), (range 5–30)^b^	21.9 (5.5)	19.7 (7.9)	0.21

^a^Models control for daily wear time

^b^Higher values means better coping and more exercise-related confidence

At 3- and 6-month follow-up, the retention rate in each arm was 100 % (Fig. 1). On average, intervention participants attended 5 out of 6 classes (range 1–6). Only 16 % of intervention group participants completed at least 3 telephone counseling calls, and none of the intervention participants completed the maximum number of six phone calls. Two-thirds of the intervention participants attended at least one of four *melas. Mela* attendance was 48 % at the first *mela,* but dropped at each subsequent *mela* and was 12 % by the fourth *mela*.

We observed no significant differences between treatment arms for change in moderate-vigorous physical activity or saturated fat intake which were the primary efficacy outcomes (Table 2). The intervention group lost significantly more weight than the control group (Table 2). At 6 months, the intervention group had lost an average of 1.6 kg (Standard Error (SE) = 0.5), and members of the control group had lost an average of 0.2 kg (SE = 0.5) (difference between arms in weight change: 1.5 kg (SE = 0.7, *p* < 0.05). Six intervention participants (19 %) lost at least 5 % of their baseline weight in 6 months; only 3 control participants (9 %; *p* = 0.26) achieved this. Although waist circumference decreased significantly within the intervention group, the difference between intervention and control did not reach significance (Table 2).

**Table 2:** Change in outcomes, baseline to 3- and 6-months in the SAHELI study, by treatment group

Primary outcomes^a,b^	Month 3	Month 6
Change in bout-corrected moderate-vigorous physical activity, min/week		
Control, mean (95 % CI)	7.3 (−19.00, 33.56)	4.4 (−23.08, 31.83)
Intervention, mean (95 % CI)	15.5 (−13.06, 44.07)	9.5 (−19.49, 38.53)
Adjusted mean difference between arms, (95 % CI)	8.2 (−29.23, 45.68)	5.1 (−32.98, 43.26)
Change in percent kilocalories from saturated fat intake, %		
Control, mean (95 % CI)	0.12 (−0.76, 1.01)	0.58 (−0.42, 1.59)
Intervention, mean (95 % CI)	−0.24 (−1.15, 0.68)	0.37 (−0.64, 1.39)
Adjusted mean difference between arms, (95 % CI)	−0.36 (−1.60, 0.88)	−0.21 (−1.59, 1.17)
Secondary outcomes		
Change in weight, kg		
Control, mean (95 % CI)	−0.1 (−0.77, 0.49)	−0.2 (−1.14, 0.78)
Intervention, mean (95 % CI)	−1.0 (−1.65, −0.36)	−1.6 (−2.59, −0.64)
Adjusted mean difference between arms, (95 % CI)	−0.86 (−1.76, 0.03)	−1.5 (−2.81, −0.07)*
Change in waist circumference, cm		
Control, mean (95 % CI)	1.2 (−1.19, 3.52)	1.6 (−0.97, 4.19)
Intervention, mean (95 % CI)	0.1 (−2.31, 2.47)	−0.7 (−3.32, 1.91)
Adjusted mean difference between arms, (95 % CI)	−1.1 (−4.15, 1.98)	−2.3 (−5.61, 0.96)
Change in systolic blood pressure, mm HG		
Control, mean (95 % CI)	−0.4 (−5.07, 4.27)	−3.4 (−7.58, 0.77)
Intervention, mean (95 % CI)	−6.1 (−10.87, −1.25)	−3.6 (−7.84, 0.61)
Adjusted mean difference between arms, (95 % CI)	−5.7 (−12.28, 0.96)	−0.2 (−5.43, 5.00)
Change in total cholesterol, mm HG		
Control, mean (95 % CI)	2.1 (−2.84, 6.97)	3.5 (−3.76, 10.79)
Intervention, mean (95 % CI)	−1.4 (−6.44, 3.68)	−0.4 (−7.75, 7.02)
Adjusted mean difference between arms, (95 % CI)	−3.4 (−10.47, 3.59)	−3.9 (−13.88, 6.12)
Change in hemoglobin A1c, %		
Control, mean (95 % CI)	0.1 (−0.06, 0.18)	0.2 (−.03, 0.41)
Intervention, mean (95 % CI)	−0.2 (−0.28, −0.04)	−0.2 (−.46, −0.01)
Adjusted mean difference between arms, (95 % CI)	−0.2 (−0.39, −0.06)**	−0.4 (−0.74, −0.11)**
Change in fasting glucose, mg/dl D)		
Control, mean (95 % CI)	1.1 (−4.38, 6.67)	12.4 (−2.61, 27.36)
Intervention, mean (95 % CI)	−4.0 (−9.68, 1.74)	−1.8 (−17.06, 13.38)
Adjusted mean difference between arms, (95 % CI)	−5.1 (−13.05, 2.83)	−14.2 (−35.47, 7.05)
Change in energy intake, kcalories/day		
Control, mean (95 % CI)	−52 (−170.92, 66.60)	−99 (−214.72, 16.84)
Intervention, mean (95 % CI)	−182 (303.61, −59.67)	−173 (−290.33, −55.75)
Adjusted mean difference between arms, (95 % CI)	−129 (−293.40, 34.44)	−74 (−223.03, 74.84)
Change in fruit and vegetable intake, servings/day		
Control, mean (95 % CI)	0.1 (−0.45, 0.62)	0.5 (−0.07, 1.03)
Intervention, mean (95 % CI)	0.5 (−0.01, 1.09)	0.04 (−.52, 0.60)
Adjusted mean difference between arms, (95 % CI)	0.5 (−0.23, 1.14)	−0.4 (−1.15, 0.26)
Change in exercise-related confidence		
Control, mean (95 % CI)	−2.2 (−4.25, −0.17)	−1.4 (−3.62, 0.83)
Intervention, mean (95 % CI)	0.4 (−1.67, 2.52)	1.1 (−1.14, 3.38)
Adjusted mean difference between arms, (95 % CI)	2.6 (−0.01, 5.29)	2.5 (−0.47, 5.51)
Change in coping		
Control, mean (95 % CI)	−0.2 (−.63, 0.18)	0.6 (0.17, 0.96)
Intervention, mean (95 % CI)	0.1 (−0.34, 0.48)	0.4 (−0.002, 0.80)
Adjusted mean difference between arms, (95 % CI)	0.3 (−0.22, 0.80)	−0.2 (−0.63, 0.29)

a. All models are sex-adjusted; Change in moderate-vigorous physical activity is adjusted for accelerometer wear time; *p-value <0.05; ** *p*-value <0.01

The intervention group had a significantly greater decrease in hemoglobin A1C compared to the control group at 6-months (difference in HbA1c: −0.43 (SE = 0.16), *p* < 0.01). We observed no significant differences between treatment arms for changes in blood pressure, total cholesterol, glucose levels, other dietary measures, or psychosocial outcomes (Table 2).

## Discussion

In partnership with Chicago’s South Asian community, we used community input, formative data, and a CBPR framework, to develop, implement, and evaluate the **S**outh **A**sian **He**art **L**ifestyle **I**ntervention (SAHELI) among medically underserved South Asian immigrants in Chicago. This pilot study suggests that the SAHELI intervention may be more effective at improving weight loss and hemoglobin A1C than print education materials. We also judged that the culturally-salient lifestyle intervention was feasible, and that the use of CBPR helped us to enroll underserved South Asians and retain 100 % of the participants in a 6-month lifestyle intervention trial. Although we did not observe significant differences between treatment arms for changes in primary outcomes, the intervention effect on physical activity and saturated fat intake appeared to be in the hypothesized direction, with improvements seen at 3 and 6-months. Changes in other intermediate clinical outcomes may have been difficult to detect given that participants’ average blood pressure and cholesterol were already at recommended levels at baseline.

Interestingly, the control group’s hemoglobin A1c increased at 3- and 6-months, even though this group lost a small amount of weight and improved physical activity. Because there was a significant difference between the intervention and control arms in English proficiency, we repeated our analyses adjusting for limited English proficiency, and our results did not change. It is also plausible that the increased hemoglobin A1c in the control group was a result of the control group’s increased waist circumference from baseline to 6 months. Others have found that waist circumference was more strongly associated with cardiometabolic risk in South Asians than body weight [47, 48]. Future intervention studies could help to determine if waist circumference loss confers greater metabolic benefits than weight loss in South Asians.

The observed weight loss in SAHELI intervention participants was much more modest than what has been reported in the intensive, year-long Diabetes Prevention Program (DPP) study and in other community-based lifestyle interventions of longer duration [49]. We hypothesized that this was partly due to SAHELI being a shorter intervention than the DPP because our results are similar to lifestyle interventions of shorter duration [50]. The modest weight loss in SAHELI could also be to differential effectiveness of lifestyle interventions in South Asians arising from differential uptake and adherence to intervention components [18]. For example, attendance at group classes was high, but few participants completed any of the follow-up telephone counseling calls or attended the *melas*. Others have found that telephone calls and text messaging were effective for scaling-up behavioral interventions and supporting long-term behavior change [51–53]. Although our study team’s formative work informed the content and shorter duration of SAHELI, we still found many barriers to telephone calls, such as lack of time among participants who were working long shifts, mobile phones being shared among family members, and that participants may not have understood the purpose of the follow-up phone calls. Study staff were also contacting participants by telephone to remind them about study assessments and accelerometers, and to conduct 24-h diet recalls, all of which created significant participant burden and may have led to confusion among participants about the purpose of follow-up telephone counseling calls. These findings suggest that simply adapting effective interventions or technologies for minority groups may not produce similar results as in the general population. In addition, a clear need exists to balance pragmatic and sustainable implementation with the science of long-term behavior change.

A recent meta-analysis found a dearth of randomized control trial evidence addressing diet, physical activity and overweight/obesity in migrant and native South Asian populations [18, 27, 28, 44]. In the Indian Diabetes Prevention Program (IDPP), individuals in India with impaired glucose tolerance were randomized to a lifestyle intervention or lifestyle intervention plus metformin [44]. The lifestyle intervention included individual advice on physical activity and diet modification and monthly telephone contacts. Study subjects in the IDPP had a relatively low BMI (26 kg/m2) at baseline and did not lose any weight over the course of the intervention [44]. However, IDPP intervention participants reported improved adherence to physical activity and diet recommendations and had a significantly lower incidence of diabetes compared to the control group. The IDPP results suggest that increased physical activity, even in the absence of significant weight loss, may be important for diabetes prevention in South Asians. Research studies specifically testing which components of multicomponent lifestyle interventions are most effective for reducing cardiometabolic risk factors in South Asians are still needed.

The Prevention of Diabetes and Obesity in South Asians (PODOSA) study, a family-cluster randomized control trial of a culturally adapted diet and physical activity intervention for South Asians at high risk for diabetes in the United Kingdom, found similar results to ours, but over a longer period. In PODOSA, the intervention group lost more weight than the control group at 3-year follow-up (adjusted mean difference = −1.6 kg), with weight loss of 1.1 kg in the intervention group and a weight gain of 0.5 kg in the control group [27]. Families in the intervention group had 15 home visits from a dietitian over 3 years. PODOSA was the longest of any lifestyle interventions targeting migrant South Asians, and its sustained effect on weight loss at 3 years may be due to the intervention being family-based and home-based. Unlike PODOSA and SAHELI, a culturally adapted lifestyle intervention for South Asians in the Netherlands showed minimal weight loss in intervention participants (−0.2 kg.) and no difference in weight change between the intervention and control group [28]. The intervention in the Netherlands study was primary-care based and moderately intensive, including 8–10 individual counseling sessions plus a 20-week physical activity program. Future studies may want to evaluate how the intervention setting (i.e. home, community, or clinic) influences outcomes.

Project Reaching Immigrants through Community Empowerment (RICE) delivered a community health worker led diabetes prevention intervention, consisting of 6 education sessions and follow-up telephone calls, to Asian Indians in the U.S. The intervention group had greater decreases in glucose than the control group and the between-group differences for changes in weight approached significance at 6 months (−2.2 kg in intervention vs −0.5 kg in control) [30]. Similar to the results of Project RICE and PODOSA, the SAHELI pilot study results show promise for improving weight and cardiometabolic risk factors in South Asians. Although there are very few relevant intervention studies in South Asians, there is a pattern that is starting to emerge, of little or modest change in risk factors in South Asians following lifestyle interventions [27, 28, 30, 54]. Results of these trials raise key questions about which aspects of cultural adaptation are most effective, the relative benefits of community-, clinic-, or home-based lifestyle interventions, and how interventions implemented in real-world settings can yield sustained weight loss and metabolic benefits without over-burdening participants and sites.

### Strengths and limitations

This study has several strengths. First, the CBPR approach helped to engage and retain underserved South Asian immigrants in a clinical research trial; others have reported that recruitment and retention of South Asian immigrants into research studies has been a challenge [28, 55–57]. In addition, the study team included multilingual and culturally-concordant staff who were trained in intervention delivery and quality control. SAHELI was implemented in a real world setting which increased external validity and provided information on strategies needed for future translation of lifestyle intervention research into community settings. Lastly, this study included objective physical activity measurement and achieved high quality data collection.

Limitations of this pilot study are inclusion of only a single site in Chicago, and that the study team reach was only able to reach and enroll 20 % of the individuals who were initially targeted. Both these factors limit generalizability of the results and should be addressed when conducting a larger confirmatory trial. In addition, the psychosocial questionnaires that were used to measure confidence and coping have not been validated in South Asian populations. It is also clear that study participants under-reported dietary intake during the 24-h diet recalls, and this under-reporting made it difficult to accurately capture baseline kilocalories, macronutrients, and fruit and vegetable intake, as well as subsequent changes in diet [30].

## Conclusion

A community-based, culturally-salient lifestyle intervention was effective at reaching medically underserved South Asian immigrants who may not be reached by traditional biomedical and clinic-based prevention interventions. The study partners’ future work will evaluate strategies to increase and sustain weight loss in intervention participants. We are also planning a larger scale randomized control trial to confirm these preliminary results, provide information on effective intervention components, and inform implementation and scalability. The translation and sustainability of lifestyle interventions into diverse community and clinical settings for South Asian immigrant populations is a public health priority.

## Additional file

Additional file 1:
**SAHELI Study Protocol.** The additional document details the protocol related to the South Asian Heart Lifestyle Intervention (SAHELI) Study. (PDF 8865 kb)
