# Supplementary material for: Translating a heart disease lifestyle intervention into the community: the South Asian Heart Lifestyle Intervention (SAHELI) study; a randomized control trial
Source: BMC Public Health. 2015 Oct 16;15:1064. doi: 10.1186/s12889-015-2401-2 (PMC4609098; doi:10.1186/s12889-015-2401-2)
Supplement: Additional file 1: — SAHELI Study Protocol. The additional document details the protocol related to the South Asian Heart Lifestyle Intervention (SAHELI) Study. (PDF 8865 kb) [file 12889_2015_2401_MOESM1_ESM.pdf]

South Asian Heart Lifestyle Intervention (SAHELI) study protocol

Principal Investigator: Namratha Kandula, MD, MPH

TABLE OF CONTENTS

CHAPTER 1: STUDY ORGANIZATION……………………………………………..…2
CHAPTER 2: RECRUITMENT AND INTERVIEW GUIDELINES……………………….7
CHAPTER 3: TELEPHONE ELIGIBILITY INTERVIEW……………………………...14
CHAPTER 4: BASELINE STUDY VISIT………………………………………….....18
CHAPTER 5: RANDOMIZATION……………………………………………………47
CHAPTER 6: ANTHROPOMETRY……………………………………………….…49
CHAPTER 7: BLOOD PRESSURE…………………………………………………...55
CHAPTER 8: ACCELEROMETERS………………………………………………….66
CHAPTER 9: 24 HOUR DIETARY FOOD RECALL…………………………………..75
CHAPTER 10: SOCIAL NETWORK QUESTIONNAIRE..…....112
CHAPTER 11: LINKAGE TO PRIMARY CARE………………………………………115
CHAPTER 12: CONTROL GROUP…………………………………………………….117
CHAPTER 13: SAHELI INTERVENTION……………………………………………..121
CHAPTER 14: 3 AND 6 MONTH ASSESSMENT……………………………………..229
CHAPTER 15: INDIVIDUAL TELEPHONE SUPPORT………………………………282
CHAPTER 16: LABORATORY PROCEDURES……………………………………....306

CHAPTER 1: STUDY ORGANIZATION

OVERVIEW

The Translating a heart disease lifestyle intervention into the community (SAHELI) study has been developed with participating units who are committed to conducting the study in a uniform manner, adhering to the common protocol, Operations Manual, and National Institutes of Health and Good Clinical Practice Procedures. Standardization, supervision and coordination of all procedures will be enhanced through peer review and quality control mechanisms.

This study includes two study sites, a Community Advisory Board, and the Sponsor, the National Heart, Lung, and Blood Institute at the National Institutes of Health.

A directory is included in Appendix 2A.

PARTICIPATING UNITS

Study Sites

Two study sites are participating in this study:
Northwestern University (NU)
Metropolitan Asian Family Services (MAFS)

The study sites are responsible for recruitment, enrollment, assessment and follow-up of a total of 60 study participants according to the study protocol and operations manual; transmitting data to Northwestern University in a timely fashion with the lowest error rate attainable; addressing queries in a timely fashion, completing data forms and participating in the scientific aspects of the study including design, analysis, presentations and publications.

The Principal Investigator at each study site is responsible for the conduct of the study at her site and for gaining and maintaining local IRB approval in accordance with the principles of Good Clinical Practice, HIPAA regulations, the Study Protocol, Operations Manual and any additional relevant study documentation.

National Heart, Lung, and Blood Institute (NHLBI)

The NHLBI is the sponsor of the study, which is administered through a R21 HL113743-01. The NHLBI has provided NU with a grant (PI: Dr. Namratha Kandula), and Dr. Kandula is responsible for administering the fiscal affairs of the study.

Laboratory Services

Blood samples will be collected during the MASALA study for outcome measures, and will be analyzed at Quest laboratories. Laboratory procedures are described in Chapter 16, Laboratory.

Quest Laboratories: Clinical site is responsible for supplying all necessary materials for blood tests and will utilize their local Quest Laboratory to perform selected laboratory tests. These tests are performed with the same standard assay methods and similar normal ranges will be utilized. Principal Investigator will review all abnormal and ‘alert’ lab results. The clinical sites are responsible for ordering

venipuncture supplies from Quest and managing the shipment of samples from the clinical site to Quest Labs. Clinical sites should contact Quest Labs directly for questions about results, re-supply of laboratory materials, or sample pickup.

ADMINISTRATION

Community Advisory Board (CAB)

Appendix 2B describes the SAHELI CAB structure. The CAB is composed of the PI and Co-Investigator, and 2 male community members and 2 female community members. CAB members represent the diversity of the South Asian community and are stakeholders, business owners and community organizations. CAB members have an expertise in local business, community work, community health education and social work.

The CAB is responsible for all decisions concerning the scientific and technical conduct of the study. Some of its specific responsibilities are:

- To achieve consensus on the design of the study
- To approve all substantive revisions or changes in the study protocol
- To assure that the study is conducted according to the highest scientific principles and good clinical practice
- To ensure study results are disseminated in the community
- To provide advice on sustainability of the study

The chairperson of the CAB is the Principal Investigator of the study, Dr. Namratha Kandula. The CAB will meet quarterly, or as needed, by conference call.

REGULATORY REQUIREMENTS

The following items should be kept on file at the clinical sites:

- Protocols and Amendments
    - All versions should be IRB approved
  - Informed Consents, HIPAA
  - IRB Approvals and Correspondence
    - Approvals: Original and any revisions
    - Yearly Renewal Letters
  - Site Staff CVs, and Medical Licenses
- Research Team Signature List, including Staff ID#
- Serious Adverse Event Reports
- Recruitment Advertisements
- Laboratory/Facility Certifications
    - Local Quest Laboratory

Please file Memos as you receive them in the Memo Binder.

APPENDIX 2A: SAHELI STUDY DIRECTORY

Namratha Kandula, MD
Principal Investigator

Santosh Kumar,LLM
Site Co-Investigator

Study Sites

Site	Institution	Principal Investigator
1	Northwestern University	Namratha Kandula, MD
2	Metropolitan Asian Family Services	Santosh Kumar,LLM

CONFIDENTIAL- Do not distribute, developed by Northwestern University

SAHELI DIRECTORY
Updated: March 2nd 2012

	Institution	
Site 1	**Northwestern University**	
	Namratha Kandula, MD, MPH<br>Principal Investigator	Phone: 312-503-6470<br>Email: NKandula@nmff.org<br>Address: Division of General Internal Medicine (GIM)<br>Northwestern University<br>750 North Lake Shore Drive, 10th Floor<br>Chicago, IL 60611
	David Baker, MD<br>Co- Investigator	Phone: 312-503-6407<br>Email:DBaker1@nmff.org<br>Address: GIM
	Bonnie Spring, PhD<br>Co-Investigator	Phone: 312-908-2293<br>Email: bspring@northwestern.edu<br>Address: Department of Preventive Medicine (DPM)<br>680, North lake sore Dr, Suite 1220<br>Chicago, IL-60611
	Juned Siddique, Dr.P.H<br>Co-Investigator	Phone: 312-908-9241<br>Email: siddique@northwestern.edu<br>Address: DPM
	Swapna Dave, M.B.B.S, MPH<br>Project Coordinator	Phone : 312-503-6995<br>Email: swapna-dave@northwestern.edu<br>Fax : 312-503-3350<br>Address: GIM
	Yasin Patel, BS<br>Project Coordinator	Phone : 312-503-3387<br>Cell phone :<br>Email: Yasin.patel@northwestern.edu<br>Address: GIM
	Paola Seguil<br>Health educator	Phone: 312-503-3363<br>Email: paola.seguil@northwestern.edu

	Institution	
Site 2	**Metropolitan Asian Family Services**	
	Santosh Kumar, B.Sc, LL.B (Hons), LL.M<br>Site Principal Investigator	Phone: 773-465-3105<br>Fax: (773) 465- 0158<br>Email: santosh1250@hotmail.com<br>Address: Metropolitan Asian Family Services<br>7541 N Western Ave<br>Chicago, IL 60645
	Promila Mehta, MA<br>Project Coordinator	Phone: 773-465-3105<br>Fax: (773) 465- 0158<br>Email: promilam@mafsinc.com
	Saba Kazi<br>Study recruiter	Phone: 773-465-3105<br>Fax: (773) 465- 0158<br>Email: sabk@mafsinc.com
	Himali Bharucha<br>Project coordinator	Phone: 773-465-3105<br>Fax: 773-465-0158<br>Email: himalib@mafsinc.com
	Meena Krishan<br>Study recruiter	Phone: 773-465-3105<br>Fax: 773-465-0158<br>Email: meenaj@mafsinc.com
	Hanifa Goder<br>Community Advisory Board member	Phone:<br>Email: hanifagoder@yahoo.com
	ManMohan Sharma<br>Community Advisory Board member	Phone:773-465-3105<br>Email:manmohan.mms999@gmail.com
	Nalini Goyal<br>Community Advisory Board member	Phone:<br>Email: nallugoyal@yahoo.com

	Mansoor Tejani<br>Community Advisory Board member	Phone:<br>Email: mtejani07@gmail.com

CHAPTER 2: RECRUITMENT AND INTERVIEW GUIDELINES

OVERVIEW
The SAHELI Study will be utilizing a face to face and mail/telephone recruitment of South Asians from Chicago land communities. To enhance recruitment, the study team will inform local community leaders in temples, community centers, and city politicians of the study aims and goals to elicit their support. Local religious leaders of the community will be asked to introduce our study to their congregations and encourage participation.

LANGUAGES
SAHELI will include English and Hindi speaking participants from South Asia (India, Pakistan, Bangladesh, Sri Lanka, Burma).

RECRUITMENT

Face- to-face recruitment: Community outreach at community and religious organizations will be conducted by distributing the study flyer to seek interested participants. If an individual is interested they will be given the study flyer and will be asked to provide their name and contact information. The recruitment coordinator will contact the participant by telephone and follow the guidelines for telephone recruitment below.

Mail and telephone recruitment: Permission will be obtained from Metropolitan Asian Family Services (MAFS) clients and people who go to MAFS to make contact with them about the research study. People who have given permission for contact will be contacted. The first contact with selected households will be made by mail. Each household will be sent an introductory letter and brochure that describes the main features of SAHELI. In some cases, the letter will be personalized, directed to age-eligible persons in the household. In other cases, the names or number of age-eligible persons will not be known, so the letter will be generic (addressed to "Dear Friend" or "Dear Resident").

For households with listed telephone numbers, a letter and a brochure will be sent to the head of household to briefly describe the rationale and design of the study and to encourage participation in the study. One week after sending out the letter, a telephone call will be made to enumerate the number of eligible and interested candidates in the household. After a short interview to determine eligibility and willingness to participate, individuals will be invited to participate in the study. If there is more than one eligible person in the household, only one person will be selected. If a particular age/sex stratum for recruitment has been filled, then another eligible household member can be accepted for participation.

Age and sex stratification methods
In order to have adequate numbers of both men and women and all age groups represented in the study sample, each site will be asked to recruit people in each age/sex stratum until that stratum is filled.

The recruitment targets by sex and age stratum are:

	MAFS Site	
**Age strata:**	**Men n=32**	**Women n=32**
30-45 (50%)	20	20
45-60 (50%)	20	20

Northwestern University will monitor the recruitment by age and sex strata to ensure that we recruit the target numbers of individuals per stratum. Once a stratum has been filled, no further participants should be recruited into that stratum.

Due to difficulty in recruiting men into the study we may recruit more women into the study.

Initial Contact
The initial personal contact will be made by telephone or in-person. The goal of the initial contact is to complete the eligibility forms. Once it has been established that an age-eligible person who speaks a language available at that Clinical Center (English or Hindi) resides in the household, the interviewer will enumerate all age-eligible persons in the household (typically two, but occasionally more), using the Household Enumeration Form in the recruitment database. It is important for interviewers to recognize the multi-purpose nature of first contact:

- to introduce or re-introduce SAHELI and raise the respondent's level of interest in the study
- to enumerate age-eligible persons in the household
- to determine the SAHELI eligibility status of each age-eligible household member
- to recruit eligible respondents
- to obtain some information about non-respondents

Unreachable Household
In this era of aggressive telemarketing, some attempted calls will never be answered, because of screening technology, such as "caller ID." It might be helpful, if possible, to ensure that the clinic phone is identified as a university phone number (rather than "unknown caller"). A minimum of three phone calls should be attempted at different times of the day before declaring a household unreachable by phone.

Recruitment Letters
The text of an introductory recruitment letter has to be approved by the Institutional Review Board (IRB) and a template is available at the Northwestern University. Text cannot be changed without approval of the IRB.

INTERVIEWING GUIDELINES
The interviewer plays a critical role in a research study, often being the first contact a participant has with the study personnel. The attitude, personality and skill of the interviewer can directly influence the participant's decision to enter the screening process and later, to remain in the study. The ultimate goal of the research interview is to obtain standardized, accurate and reliable data, often from multiple sites. The skill with which the interviewer conducts the interview helps to ensure this goal is met.

This section contains general guidelines and techniques for recruiting and interviewing study participants, specific suggestions for maximizing the success of the interview procedures and ideas for handling difficult interviews.

Please note: People who volunteer for research studies are "*participants*", not "*patients*".

General Interviewing Principles

Interviewer Bias
Interviewer bias is any preference or inclination that creates a systematic difference between responses obtained by different interviewers. It can be affected by:

a. respondent's perception of the interviewer and his/her reaction to that
b. interviewer's perception of the respondent and his/her reaction to that

Characteristics of a Good Interviewer

a. The interviewer creates a friendly, but businesslike atmosphere.

b. The respondent is at ease. These factors must be kept in mind:
   - The respondent may view a female interviewer as less threatening.
   - The respondent may view a much older interviewer as judgmental.

c. The interviewer obtains the answer to the question that is asked by:
   - Proper use of probes
   - Repeating a question rather than interpreting it

d. The interviewer obtains clarification of confusing answers.

e. The interviewer gives only neutral responses to the respondent's answers.

f. The interviewer accurately records responses.

Special Skills Required for Interviewing

a. The ability to ask questions at the correct pace and in a conversational tone.

b. A thorough knowledge of the questions and response categories (this will keep the interview flowing smoothly).

c. Knowledge of how and when to use probes.

d. The ability to think as an interviewer and to temporarily put aside other roles (e.g., researcher, health care provider).

e. The ability to maintain a positive attitude about the interview (this lets the respondent know that the interview is important).

f. The ability to keep some level of control over the interview process (e.g., by rewarding the respondent for answering questions but not for other behavior).

g. Additional interviewer attributes:
   - Mobility (for personal interviews)
   - Flexibility over schedule
   - Neat, pleasant, professional appearance
   - Not too timid, not too aggressive

Interviewer Training

a. Training must cover all aspects of the interview, including:
   - Introducing oneself
   - Handling people who are reluctant "at the door"
   - Following instructions for selection of respondent
   - Obtaining consent
   - Answering questions

- Obtaining privacy for the interview
- Setting respondent at ease
- Administering the interview
- Ending the interview

b. Role playing, using both standard and problematic situations is an important aspect of training and allows trainees to discuss and solve problem that could arise in an interview with a participant.

Interviewing Techniques

Standardizing Interviewing Techniques

a. In order to produce data that can be considered collaborative, SAHELI study designers must develop and use standardized approaches to train interviewers and collect information about participants. Standardization is achieved by using scripts in training, establishing qualifications for interviewers, reviewing collected data, and observing interviewers in the field.

b. Trainers will use scripts to teach probing techniques and to determine if interviewers are following skip patterns in the forms and adhering to the protocol. Interviewers will be trained to avoid leading or providing answers for the study respondent.

Recruiting the Study Respondent
SAHELI participants represent a variety of people, ranging in age from 30 to 60. The first contact with the study respondent may be by telephone. Because telephone contact can make it easy for the respondent to decline, interviewers will be trained in effective telephone technique. They will also be taught to overcome respondents' objections and deal with difficult situations, some of which are described below.

Suspicions About the Project
Many people will not be aware that NHLBI is doing a study in the community and will seem suspicious. Interviewer's thorough understanding of the study will help to allay the respondent's fears or suspicions. Interviewer must learn to put the respondent at ease and to establish legitimacy of the call.

Difficulty Understanding
Some of the calls will be with persons who have difficulty understanding questions. Questions should be read slowly and distinctly, and respondent should be given adequate time to answer. If necessary, questions should be repeated, but care should be taken not to insult the respondent by suggesting that he/she does not understand.

Diffusing Sensitive Questions
Some respondents may hesitate or decline to answer questions they consider intrusive or sensitive (e.g., questions about alcohol consumption or income).Interviewer's professional handling of a sensitive issue can help to alleviate their fears. The more secure interviewer feel about the confidentiality of the study, the more apt he/she will be to give a sense of security to the study respondent. However, if all else fails, the interviewer may simply offer them the option to decline answering a specific question.

Getting Permission from Study Respondent's Family
It is important that the family of the respondent be clear about what is involved in the process of recruitment. Both family members and respondent should be encouraged to raise questions or concerns about the study and the respondent's participation in it. Encouraging them to ask questions, and the interviewer's thorough and thoughtful responses to those questions, will help to alleviate their concerns.

The Interview
The following procedures are recommended for a successful interview:

1. Prior to making a call, prepare all materials (e.g., appropriate forms, reference materials, scripts) that will be necessary for the interview.

2. Find an area where you can talk and write comfortably with minimal distractions.

3. Make sure that the participant understands the questions and that you are interpreting the responses accurately. Do this by restating what you think the participant is telling you or asking him/her to restate the question you are asking. At the same time, be careful not to impose your interpretations on the interview questions or the participant's comments.

4. Convey your interest in the participant's thoughts and feelings, but do your best to keep him/her focused on the interview questions. When the participant strays from a question, try to use what he/she is saying to redirect the conversation back to the interview questions. Give positive reinforcement for direct answers. If necessary, set time limits at the outset of the interview to encourage the participant to stay on track.

5. Participants may try to convince you to answer certain questions for them. Let the participants know that you are interested in their answers.

6. Be aware of any hearing and vision impairments and their effects on the participant's understanding of the interview questions. If necessary, read the interview questions to participants who have visual impairments or limited reading ability.

7. Communicate with other interviewers and the project director to share ideas about how to deal with difficult situations and to agree on consistent explanations for questions that are frequently misunderstood by participants.

8. Encourage, but do not force, participants to answer to all questions.

9. If non-participants are present during the visit, address the participant directly and do not encourage conversation with other parties. If necessary, ask that you and the participant be left alone for a brief time to complete the questionnaire.

10. Be able to adapt to interruptions. Let the participant know that you are willing to continue the interview after the interruptions are completed.

11. Make the interview a positive experience for the participant. React favorably to answers and give compliments, when appropriate.

12. Give the participant clear information about when the next clinic visit will be conducted and follow through with the plans that you make. If a change is necessary, be sure the participant is informed and understands the reason for the change.

Tackling Difficult Questions
The following are examples of, and suggested responses to, questions you may encounter.

"How do I know you and the survey are legitimate?"

If the respondent is concerned about the legitimacy of the survey, repeat your introduction, remind him/her about the introductory letter, and offer to mail another one if necessary. Also point out that local health officials are aware of the survey, and offer to mail to the respondent a reproduction of newspaper clippings and/or endorsements.

"What's this study about?"

This study is to teach people how to improve their diet and physical activity and control their blood pressure, cholesterol and diabetes.

"I don't want to buy anything."

Explain that we are not selling anything. We are doing an important research study and all the tests and services offered will be done free of charge.

"I can't help you because I have never had heart disease."

Explain that the study is enrolling people who do not already have heart disease and that you would like to talk to the respondent about his/her health.

"Will this affect my medical care?"

Explain that we are doing research and that the study will not affect any medical care the respondent now receives. The clinic is only gathering data, not doing diagnostic work. All results of the medical examination will be forwarded to his/her doctor, if so desired. Also explain that all information is held in strict confidence and that public reporting of the findings of this study will contain only statistical information.

"My doctor may not want me to be in the study."

Explain that all area doctors support SAHELI. Suggest that the respondent contact his/her physician to discuss the study.

"How long will it take to complete?"

Explain that the interview this day will take about 15 minutes and that they will be invited to come in for a 2 hour baseline visit at MAFS. The baseline visit will include a fasting blood draw, Blood pressure, weight, height and waist measurements, health and diet surveys, receipt of an accelerometer to track physical activity. Participant's eligibility will be determined based on the findings from the baseline visit and participants will be randomly assigned to either a control group or the intervention group. Participants in the control group will receive their test results and a referral to a primary care provider. The intervention group will attend group classes once a week for a total of 6 weeks about physical activity, diet, weight and stress management. Each class will last for about 90 minutes.

"Where did you get my name?"

Explain that because they receive services from MAFS, we are offering this program to all MAFS clients.

"I already have a doctor and s/he examines me regularly."

Explain that all information gathered in the study will be made available to his/her physician. Restate that these classes are not offered in a doctor's office. This education will help you improve your health and reduce the chance of heart disease. These examinations may or may not turn up important information about his/her health; if they do, we will share it with the respondent and his/her physician.

CHAPTER 3: TELEPHONE ELIGIBILITY INTERVIEW

OVERVIEW

This form is administered to potential SAHELI study participants. In most cases the form will be administered over the phone, but in some cases it will be administered in person. Please note that the respondent might not have any prior knowledge of the study or, in some cases, might be familiar with the main features of the study. The purpose of the Screening/Recruitment Questionnaire is to determine eligibility for SAHELI study but please remember that at the same time you will be introducing the study and trying to raise the respondent's interest.

PROCEDURES

General Instructions

1. The Screening/Recruitment Questionnaire is printed in English. A copy of the form in Hindi is used for translation.

2. Complete form using a black ink pen or complete directly on the recruitment database that has been constructed for the SAHELI study. Please write legibly and make sure that all circles are completely filled in.

3. Try to adhere to the scripts included on the questionnaire as much as possible, but feel free to use common sense. (For example, don't repeat information that the participant obviously already knows.) If the participant asks questions or requests a clarification, try to answer to the best of your ability. If you don't know the answer, tell the person that you don't know the answer and that you will check with your supervisor and get back to him/her.

   Remember: you are the first study representative to interact with a potential participant. The person's decision about whether or not to join SAHELI study is dependent, in part, on his/her interaction with you.

4. Unless instructions on the form specifically direct you to end the interview or to skip a question, complete the entire form for each participant.

Completing the Form on the Recruitment Database

1. For ***date*** enter date of the call. For example, July 7, 2000 would be entered 07/07/2000.

2. For ***phone number called*** enter the three-digit area code followed by the seven-digit phone number.

3. Record the Participant ID number in the box provided.

Record the date(s) and time(s) of attempted phone calls on a phone call log. An Interviewer should make at least three attempts to reach a household before the number is determined to be unreachable.

Questions from Screening Candidates

Questions people might ask about why you are calling, and some sample responses:

I don’t want to buy anything!

"We are not selling anything. We are calling about a new medical research study that we want to tell you about."

I'm too busy!

"I realize that people are very busy these days. Are there other days and times that are better for you?"

How long will this take?

"It will only take about 10 to 15 minutes of your time today. I'll move through the questions as quickly as I can." Immediately ask the first question.

RECRUITMENT DATABASE

Database overview

The main purposes of the recruitment database are to:

1. Manage recruitment lists
2. Perform eligibility screening of potential participants
3. Record appointments and manage follow-up of eligible participants
4. Assign participants to cohorts
5. Randomization of participants into the study
6. Log phone contacts.

The features of the forms are detailed below.

Form Details

<u>Main Menu</u>

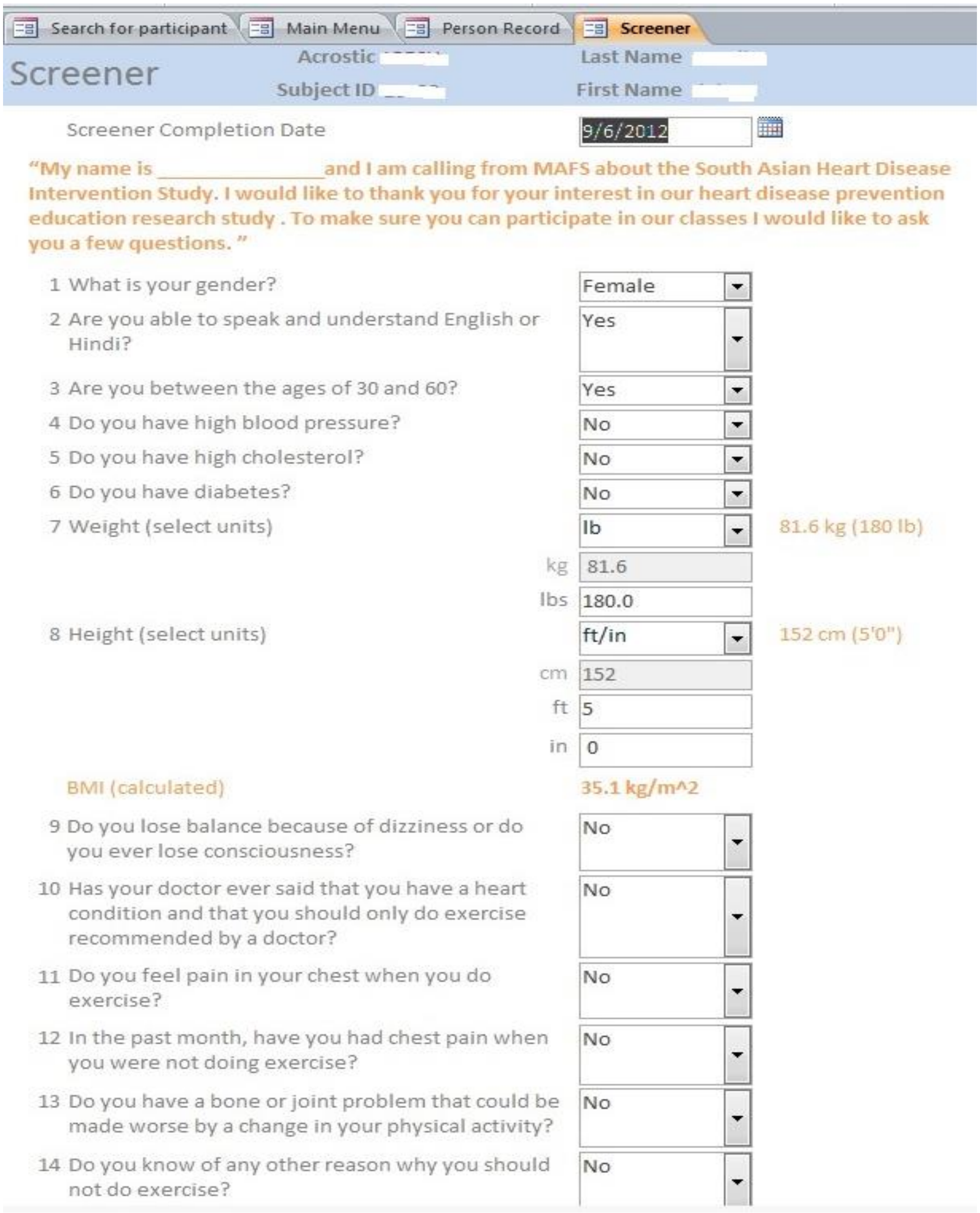

Search for participant | Main Menu | Person Record | Screener

Screener

Acrostic [illegible] Last Name [illegible]

Subject ID [illegible] First Name [illegible]

Screener Completion Date: 9/6/2012

"My name is ________________and I am calling from MAFS about the South Asian Heart Disease Intervention Study. I would like to thank you for your interest in our heart disease prevention education research study . To make sure you can participate in our classes I would like to ask you a few questions. "

1 What is your gender?	Female	
2 Are you able to speak and understand English or Hindi?	Yes	
3 Are you between the ages of 30 and 60?	Yes	
4 Do you have high blood pressure?	No	
5 Do you have high cholesterol?	No	
6 Do you have diabetes?	No	
7 Weight (select units)	lb	81.6 kg (180 lb)
kg	81.6	
lbs	180.0	
8 Height (select units)	ft/in	152 cm (5'0")
cm	152	
ft	5	
in	0	
BMI (calculated)	35.1 kg/m^2	
9 Do you lose balance because of dizziness or do you ever lose consciousness?	No	
10 Has your doctor ever said that you have a heart condition and that you should only do exercise recommended by a doctor?	No	
11 Do you feel pain in your chest when you do exercise?	No	
12 In the past month, have you had chest pain when you were not doing exercise?	No	
13 Do you have a bone or joint problem that could be made worse by a change in your physical activity?	No	
14 Do you know of any other reason why you should not do exercise?	No	

Question	Answer
15 Do you take insulin to manage your diabetes?	No
16 Have you ever had a stroke?	No
17 Have you ever had a Heart Attack?	No
18 Are you legally blind?	No
19 Do you plan to move out of the area in the next 2 years?	No
20 Do you have a family member enrolled in this study?	No
21 Do you have any problems sitting for 2 hours?	No
22 Are you pregnant or breast-feeding?	No

The patient is ineligible if they have answered YES to any of the above questions except question 1 to 9.

Is Patient Eligible or Ineligible? Eligible — Predicted: Eligible

If INELIGIBLE: "Based on what you told me, it looks like you may not be able to participate in the education classes. However would it be ok if I contacted you for future programs or research studies

Phone Number

Alternative Phone Number

If ELIGIBLE: "Great, It looks like this research will be helpful to you. Now I am going to tell you the dates and times these classes will be held.
(USE THE GOOGLE CALENDAR TO SCHEDULE APPOINTMENT)

CHAPTER 4: BASELINE STUDY VISIT

OVERVIEW

The visit will include several questionnaires and procedures (i.e., anthropometry, blood pressure measurement, fasting blood collections, etc). We estimate that the complete visit will require between 2 to 2.5 hours and must be completed in one day.

VISIT GUIDELINES

General

- Urine pregnancy test to be conducted for women who are menstruating.
- Anthropometry and blood collection should be performed while the participant is fasting. (If participant is not fasting, record date and time he/she last ate or drank.) Blood pressure measurement should be done before venipuncture. Questionnaires do not require fasting.
- Blood drawing should be done after a minimum of a 10-hour fast and before 10:30am.

Examination Guidelines

Guidelines for baseline visit are listed below.

- Seated blood pressure and urine collection for urine pregnancy test should be done immediately following the greeting and informed consent, and before venipuncture.
- Resting blood pressures should be obtained after the participant has been in the seated position for at least five minutes.
- Venipuncture should be performed in the fasting state after blood pressure measurement. If a participant comes to the clinic non-fasting, exam components that do not require fasting may be completed, and then schedule the participant for another visit for fasting blood collection.
- Provide a snack to participant after the blood draw.
- Questionnaires and other exam procedures may be administered after the fasting blood draw. During the interviews, make every effort to avoid distractions, ensure privacy, and maintain confidentiality for the participant. Do not conduct interviews during the lunch or in the waiting area in the clinic.

BEFORE THE VISIT

Visit 1 Forms

The coordinator should check the Daily Calendar showing clinic visits scheduled for the following day. The Calendar should list the preferred language of each person scheduled for a clinic visit, which will help determine how many sets of each form is needed for the day.

Print a set of the forms that will be completed for interviews and procedures. These will be pre-printed with participant IDs, which will be matched to an individual when he/she begins the exam. Forms can be printed somewhat in advance of upcoming visits, and it is advisable to have enough printed forms on hand for all the participants. Forms will be printed in English, with the Hindi translations available. For each participant, gather all the forms required for a visit, including the informed consent and their screening (telephone interview) form, and place into a folder labeled with his/her participant ID.

Supplies and Equipment

- Make sure the accelerometers are ready and completely charged before handing out to the participants. The instructions for the accelerometer should also be printed and laminated.
- Prepare the examination room for the anthropometry measurement, and seated BP.
- Check all instruments that will be used for the examination e.g. Amount estimation tools, phlebotomy supplies.

Staffing

- Prepare staff assignment sheet and make sure everyone knows his/her responsibilities.

Suggested Baseline visit schedule

Participant Arrives	9:00 am
Informed Consent and Participant Contact Information	9:00 – 9:20 am
Urine Sample and Pregnancy Test (if applicable)	09:20 - 09:25 am
Seated Blood Pressure	09:25 – 09:35 am
Fasting labs	09:35 - 09:40 am
Anthropometry	09:40 – 09:45 am
Snack	09:45 – 09:55 am
Questionnaires	09:55 – 10:40 am
24 hour food recall	10:40 – 11:20 am
Accelerometer usage instructions	11.20 – 11.30 pm

Instructions to Participants Before the Visit

Mail the Pre-Visit Instructions (Appendix 1A) to the participant 5–7 days before baseline visit and explain to them over the telephone when you schedule the visit. If possible, make a reminder call to the participant the day before baseline visit and reiterate the instructions. (If the participant is acutely ill—e.g. “flu” or bronchitis—when you make this reminder call, *tell him/her not to come to the clinic.* Arrange to contact him/her again to reschedule when he/she has recovered.) Before the examination, make sure the participants understand the following instructions.

1. Participants must fast for at least 10 hours before the examination. This restriction applies to all food and beverages (except water), including alcohol. Instruct them to consume dinner at least 10 hours before their scheduled appointment at the clinic. Only water and prescription medications are allowed from dinner until the start of the examination the next morning. Diabetic patients should *not* take their hypoglycemic medications the morning of the clinic visit; they should *bring the morning dose to the clinic* to be taken after venipuncture.

2. Participants should not smoke on the morning of the visit.

3. Participants should bring all current medications, both prescription and over-the-counter, including vitamin preparations and dietary supplements, to the clinic. If the participant forgets to bring the medications, schedule another visit to obtain this information or collect the information over phone.

4. Participants should bring the name and complete address of their personal physician or health plan, particularly if they wish to have examination results sent to that provider.

5. Participants should wear or bring loose-fitting clothes, preferably t-shirt, sweat pants, and slip-on shoes or sneakers.

VISIT RECEPTION

The reception process is very important in setting the participant's frame of mind for the rest of the exam day. Greet each participant warmly as soon as he/she arrives at the clinic. (If a participant arrives at the clinic acutely ill—e.g., "flu" or bronchitis—do not continue with the clinic examination. Make arrangements to contact him/her to reschedule the appointment after he/she has recovered.)

Informed Consent

An informed consent must be obtained from each potential participant before any procedures are initiated.

PURPOSE: Informed consent assures that candidates are fully informed about all of the requirements of the study, understand any risks associated with participation, and freely choose to enter the study. Extensive efforts will be made to inform candidates fully of all aspects of the study and potential risks and benefits. After the candidate has had the opportunity to discuss the study with clinic personnel, s/he will be asked if s/he wishes to continue with the visit.

PROCEDURE: Ask the candidate what language s/he is most comfortable reading, English or Hindi, and give her/him the appropriate informed consent. After the candidate has confirmed that all questions have been answered, each candidate will be asked to read, fill out and sign the SAHELI Informed Consent Form in accordance with Federal Regulations and the guidelines set by the local IRB. Encourage each candidate to ask questions if s/he does not understand any aspect of the form. It is very important that the candidate is fully informed and enters the study of her/his own free will.

Health Information Portability and Accountability Act (HIPAA)

PURPOSE: The Health Information Portability and Accountability Act (HIPAA) is designed to protect the confidentiality of protected health information (identifiable private information from medical records) that research screenees give during the study.

PROCEDURE: The completed HIPAA form should be filed in the screenee's study binder.

Eligibility Check

The participant's eligibility should be reviewed after the participant has signed the informed consent. Review the Eligibility Check form on database with the participant and confirm that the participant was

eligible to come for the baseline visit. If a participant is not eligible upon completion of this form, please thank them for their time and tell them that they are not eligible to participate in the study.

Participant Contact Form

The Participant Contact Form (Appendix 1B) allows us to collect information (name, address, telephone number, and email address) on the participant, his/her health care provider(s), and any proxies or contacts he/she may designate. We will use this information to contact and communicate with the participant and his/her physician(s), proxies, or other contacts. This form should remain in the participant's study folder.

This is an interviewer administered form. You should emphasize that the contact form needs to be filled out as completely and accurately as possible. The participant should not use a nickname in place of a full, legal name. He/she should provide an area code with each phone number, even if within the local calling area. Boxes for items of information that are not applicable should be left blank. You should verify health care provider information using a local telephone directory. Obtain missing information over the phone.

Do not assume you know how to spell a participant's name. Ask the participant: **"Please spell your first name, middle name and last name for me."**

PARTICIPANT CONTACT INFORMATION

Name: ______________________________

DOB: _________ / ________ / __________
Month Day Year

Marital Status: ☐ Single ☐ Married ☐ Divorced ☐ Widowed

Preferred language: ☐ English ☐ Hindi ☐ Urdu ☐ Other: ____________

Address: ______________________________ **Apt./Room #:**_______

City: ______________________ **State:** _______ **Zip Code:** ___________

Day Phone: (____) _____ - ________ **Evening Phone:** (____) _____ - ________
Area Code Number Area Code Number

Mobile Phone: (____) _____ - ________ **Email:** ______________________
Area Code Number

Best time to call: ____________________ OK to leave message? ☐ Yes ☐ No

Preferred name: ______________________________

After completing the name, DOB, preferred language, address, telephone number, and email address section, the participant will need to answer the following questions:

1. *Do you plan to be out of this area for an extended period of time (a month or longer) within the next year?*
    - If no, continue to the next question.
    - If yes, approximately when will you leave and when will you return? (provide month/year for both)

2. *Will there be a change in your local address within the next three months?*

- If yes, what will your new address be? (provide street address, city, state, and ZIP code)

A. Emergency Contact Information

"Please provide the following information on two people who are familiar with the status of your health AND who could help us contact you, if necessary. If possible, please include one person who lives with you and one who does not."

The participant should provide as much information as possible. Assist him/her, if necessary, in obtaining information.

EMERGENCY CONTACT INFORMATION

"Please provide the following information on two people who are familiar with the status of your health AND who could help us contact you, if necessary. If possible, please include one person who lives with you and one who does not."

Emergency Contact Name: ____

Emergency Contact Phone: (________) ____________ - ______________________
Area Code Number

Emergency Contact Name: ____

Emergency Contact Phone: (________) ____________ - ______________________
Area Code Number

B. Health Care Provider Information

"Please provide the following information about your health care provider."

- ***"Do you have a clinic, doctor, nurse, or physician's assistant who provides your usual medical care?"***
  If no, the form is completed.
  If yes, please provide the following information for this clinic or person (provide name, title, address, telephone number) and then continue to the next question.

- ***"When were you last seen by this person?*** "(provide month/year)

- ***"Would you like us to send your test results to this person?"***
  If yes, arrange to send a copy of the anthropometry/BP results and Quest lab results, to the physician in one packet when all results are available. All letters mailed should be tracked in the participant recruitment database.

HEALTH CARE PROVIDER INFORMATION

☐ M.D. ☐ R.N.P. ☐ P.A. ☐ Other: ________________________

First name:________________________ **Last name:** ________________________

Clinic Address:____ **Suite #:**_______

City: ___________________________________**State:**___ ___**Zip Code:**__ __ __ __ __

Clinic Phone: (________) ____________ - _______________________
Area Code Number

Date of last visit: _________ / ________
Month Year

Does the participant want results sent to their PCP? ☐ Yes ☐ No

DEMOGRAPHICS

Purpose: The DEMOGRAPHICS form collects descriptive information about the study population, as well as addressing miscellaneous study criteria.

Procedure: This is an interviewer-administered form. The participant will be asked to give his/her date of birth, relationship status, racial/ethnic background, religion, and highest educational level achieved.

Ask the participant each question and record his/her answer. For primary language spoken at home, only one answer should be selected. If a participant gives more than one answer, explain to him/her that the form requires that s/he select only one answer. If more than one bubble is selected, the form will be rejected from the database.

Questions:
Q1. *What is your date of birth?*

Eligibility note: Participants must be between 30 and 60 years of age to be eligible. Date will depend on when they are being screened.

Q2. What language will the interview be conducted in?
Please be sure to have the appropriate forms available for the participant.

Q3. *What is your gender?* Fill in the appropriate circle.

Q4. Where were you born?
Select one of the following:

- Bangladesh
- India
- Nepal
- Pakistan.
- Sri Lanka
- United States
- Other (please specify country). Write in space provided.

5. If born in another country: *"how many years have you lived in the United States?"*
Record number of years in box provided.

6. *What language is primarily spoken in your household?* Fill in the appropriate circle. If "other," specify language.

7. In SAHELI, we are tracking spousal pairs. Please ask the participant *"Is your spouse or other family member enrolled in SAHELI?"*

8. *How do you best describe your religion?* Please mark all that apply.

9. *What is the highest degree or level of school you have completed?* Fill in the appropriate circle. If currently enrolled, mark the highest grade completed or highest degree received.

DEMOGRAPHICS
SAHELI study

Visit: ○ Baseline 3 month 6 month

Coordinator Instructions: Please use a black pen and fill in bubbles completely.

1. What is your date of birth?

☐☐/☐☐/☐☐☐☐
Mo Day Year

2. Language interview will be conducted in
   - ○ English
   - ○ Hindi

3. What is your gender?
   - ○ Male
   - ○ Female

4. Where were you born?
   - ○ Bangladesh
   - ○ India
   - ○ Nepal
   - ○ Pakistan
   - ○ Sri Lanka
   - ○ United States → Skip to Question 6
   - ○ Other -please specify: ☐☐☐☐☐☐☐☐☐☐☐☐☐☐☐☐

5. If born in another country:

   How many years have you lived in the United States? ☐☐ years

6. Which language is primarily spoken in your household? (Mark only one)

7. Is your spouse or other family member enrolled in SAHELI study?
   - ○ Yes → What is your relationship to him/her ☐☐☐☐☐☐☐☐☐☐☐☐☐☐☐☐
   - ○ No

8. How would you best describe your religion? (Mark all that apply)

9. What is the highest degree or level of school you have completed? *If currently enrolled, mark the highest grade completed or highest degree received.*

URINE SAMPLE FOR PREGNANCY TEST

Purpose: All women of child bearing potential must have a negative pregnancy test prior to randomization into the study. (Note: Women of reproductive age who have had a hysterectomy or tubal ligation do not require a pregnancy test.) Clinic staff should follow the directions on the pregnancy test kit.

Procedure: See Chapter 16, Laboratory Procedures for instructions.

SEATED BLOOD PRESSURE

Purpose: Blood pressure (BP) level is a major risk factor for coronary heart disease, congestive heart failure, and stroke. Heart rate reflects autonomic nervous system function and cardiovascular fitness. The measured BP level is subject to biological and observer variability.

Only study staff that are trained and certified can take blood pressure measurements for the SAHELI study.

Procedure: Refer to Chapter 7, Blood Pressure for instructions.

PHLEBOTOMY

Purpose: To measure traditional (cholesterol, glucose, fasting sugar) risk factors for cardiovascular disease.

Procedure: Refer to Chapter 16, Laboratory Procedures

ANTHROPOMETRY

Purpose: To measure body weight, height, abdominal girth and hip girth. The measures of weight and height will be used to calculate body mass index; waist and hip circumferences are other measures of body composition that have been linked to cardiovascular disease risk.

Procedure: Refer to Chapter 6, Anthropometry

QUESTIONNAIRES

Purpose: At Visit 1, participants will be asked to complete 11 interviewer-conducted questionnaires that are listed below.

INTERVIEWER-ADMINISTERED QUESTIONNAIRES TO BE ADMINISTERED IN THE ORDER BELOW:

- DEMOGRAPHICS
- MEDICAL HISTORY
- PERSONAL HISTORY
- CULTURAL TRADITIONS
- 24 HOUR FOOD RECALL
- MEDICATION QUESTIONNAIRE AND INVENTORY
- PHYSICAL ACTIVITY
- HEART DISEASE QUESTIONS

SOCIAL NETWORK SURVEY
EXERCISE CONFIDENCE SURVEY
COPE SURVEY
TOBACCO USE

Medical History

Purpose: The Medical History identifies the participant's medical conditions and provides other information at Baseline Visit that may:

- be criteria for ineligibility (although the vast majority of ineligible persons should already have been identified);
- classify persons who are not candidates for certain incident non-CVD disease endpoints (such as diabetes or cancer);
- be used to adjust for co-morbidity;
- characterize the participant's access to medical care; and
- characterize family history of CVD

Participant ID #	Acrostic	Date of Interview	Staff ID #
		___/___/_____ Mo Day Year	

SAHELI STUDY
MEDICAL HISTORY

BASELINE 3 MONTH 6 MONTH

Coordinator Instructions: Read these instructions to the participant. The following are some questions about your medical history. Some of the questions may refer to things that happened or began a long time ago, so please answer to the best of your knowledge.

1. On a scale of 1 to 10, with 1 being poor and 10 being excellent, how would you describe your own health?

1 **2** **3** **4** **5** **6** **7** **8** **9** **10**
Poor **Good** **Excellent**

Interviewer Only:
Please record the point on the scale to which the participant has drawn the line here:

Has a doctor ever told you that you had any of the following?:

2. Emphysema	O Yes	O No	O Don't know
3. Asthma	O Yes	O No	O Don't know
4. Arthritis	O Yes	O No	O Don't know
5. Cancer	O Yes	O No	O Don't know
**IF YES to Q.5**, which type:	O Yes	O No	O Don't know
a. Prostate cancer	O Yes	O No	O Don't know
b. Breast cancer	O Yes	O No	O Don't know
c. Lung cancer	O Yes	O No	O Don't know
d. Colon cancer	O Yes	O No	O Don't know
e. Non-melanoma skin cancer	O Yes	O No	O Don't know
f. Blood cancer (leukemia, lymphoma, or other)	O Yes	O No	O Don't know
g. Other cancer	O Yes	O No	O Don't know

Personal History

Purpose: The Personal History form is used to collect information on socio-economic status (SES) which is related to an individual's risk of cardiovascular disease.

Procedures: Interviewer administered. If he/she is unsure about an exact answer tell him/her to give a best estimate.

Participant ID #	Acrostic	Date of Interview	Staff ID #
		Mo / Day / Year	

PERSONAL HISTORY
SAHELI STUDY

Visit: Baseline 3 Month 6 Month

Participant Instructions: Please use a black pen and fill in bubbles completely.

This form is intended to collect information about your background and lifestyle which may impact your risk of cardiovascular disease. Please complete all items except those which you are specifically instructed to skip. If you are unsure about the answer to a specific question, please estimate the answer to the best of your ability. If you have a question about a particular item, please write a small "x" in the margin of the form, making sure not to write it near any of the response bubbles, and then ask a staff member for clarification of those items after you have completed the rest of the form.

1. On a scale of 1 to 10, with 1 being not at all and 10 being very well, how well do you speak English?

1 2 3 4 5 6 7 8 9 10
Not at all Poor Fair Well Very well

Interviewer Only:
Please record the point on the scale to which the participant has drawn the line here:

2. How often do you have someone like a family member, friend, hospital or clinic worker or caregiver, help you read hospital materials?
   - o Always
   - o Often
   - o Sometimes
   - o Rarely
   - o Never

3. How often do you have problems learning about your medical condition because of difficulty

Cultural Traditions

Purpose: The Cultural Traditions is designed to measure characteristics that may be important in understanding the causes of cardiovascular disease.

- Cultural traditions and beliefs is an area of interest in the SAHELI study. Our preliminary work found that a few of the questions we developed for the study were good predictors of high disease risk.

Participant ID #	Acrostic	Date of Interview	Staff ID #
☐☐☐☐☐	☐☐☐☐	☐☐/☐☐/☐☐☐☐<br>Mo Day Year	☐☐☐

SAHELI CULTURAL TRADITIONS

Visit: ○ Baseline 3 Month 6 Month

Instructions: Please use a black pen and fill in bubbles completely.

1. In the future, how much do you wish these traditions from India/Pakistan would be practiced in America?

	Absolutely	Very much so	Quite a bit	A little bit	Not at all
A. Performing religious ceremonies or rituals	○	○	○	○	○
B. Serving Indian/Pakistani sweets for ceremonies or rituals	○	○	○	○	○
C. Fasting on specific occasions	○	○	○	○	○
D. Living in a joint family	○	○	○	○	○
E. Having an arranged marriage	○	○	○	○	○
F. Having a staple diet of chapattis, rice, daal, vegetable, and yogurt	○	○	○	○	○
G. Using Ayurveda, Homepathy, Unani or Herbal medicines for healing and health	○	○	○	○	○

2. How often do you fast (not eat once a day or all day)?
   - ○ Two or three times per week
   - ○ About once a week
   - ○ About once or twice per month
   - ○ Less than once a month
   - ○ Once a year for a specific period of time
   - ○ Almost never or never

3. What food do you normally or usually eat at home?
   - ○ Only Indian/Pakistani food
   - ○ Mostly Indian/Pakistani food
   - ○ Equally Indian/Pakistani and Other
   - ○ Mostly Other food
   - ○ Only Other food
   - ○ Never eat at home

24 Hour Food Recall

Purpose: The 24 Hour Food Recall will record the participants' food and beverage intake of the previous day.

Procedure: Refer to Chapter 9, 24 Hour Food Recall

MEDICATIONS QUESTIONNAIRE AND MEDICATIONS INVENTORY

Purpose: At Baseline Visit , participants will be asked to bring all medications that they are currently taking to the clinic, including prescription medications, over-the-counter (OTC) medications, vitamins, herbs and dietary supplements.

Procedure: Follow the directions on the data forms.

Filling out the MEDICATIONS QUESTIONNAIRE

Question 1:

- All prescription medications, over-the-counter (OTC) medications, vitamins, herbs and dietary supplements will be recorded on the MEDICATIONS INVENTORY FORM.
- ***If participant has brought in some, but not all of their medications***, record them on the MEDICATIONS INVENTORY FORM and complete the form when the participant brings in or calls you with the remaining medications.
- ***If participant is currently not taking any medications*** do not complete a MEDICATIONS INVENTORY FORM.

Filling out the MEDICATIONS INVENTORY

CONFIDENTIAL- Do not distribute, developed by Northwestern University

On each completed MEDICATION INVENTORY, indicate the page number of each form by filling in the corresponding bubble. (For the first form, fill in the "1" bubble, for the second form, fill in the "2" bubble, etc.) The ***total*** number of forms will be recorded on the MEDICATIONS QUESTIONNAIRE.

Participant ID #	Acrostic	Date of Interview	Staff ID #
		Mo / Day / Year	

SAHELI MEDICATION INVENTORY

Visit: Baseline 3 month 6 month

Page ○ **1** ○ **2** ○ **3** ○ **4** ○ **5**

1. Record the trade name (or generic name, if necessary). If the name of the medication is longer than the number of data entry boxes on the form, just fill out as much as possible.

2. Indicate the frequency the medication is taken.

1. Name:

Frequency ○ Daily ○ Weekly ○ Monthly ○ As needed

SPECIAL INSTRUCTIONS

Issue	Resolution
Participant took a medication 1 week ago but stopped taking it	• Record the medication
Participant has not brought in all of his/her medications or has forgotten to bring all of them	• Record the medications the Participant has with him/her and ask the participant to call you later with the name of the other medication(s).
Participant is unable to list or name all of his/her herbal remedies and supplements	• Ask the participant why s/he is taking the medication

Physical Activity

Purpose: The Physical Activity questionnaire will measure the level of physical activity across different activity categories (household chores, gardening, dancing, exercise etc.) and different intensities (light, moderate, and heavy).

Participant ID #	Acrostic	Date of Interview	Staff ID #
		Mo / Day / Year	

MEDICATION QUESTIONNAIRE

SAHELI BASELINE VISIT

PHYSICAL ACTIVITY

Coordinator Instructions: Please use a black pen and fill in bubbles completely.

Think about the types of activities you did in a typical week in the past month. For each activity, note which of these activities you did in a typical week by filling in the circle for YES or NO. For each item you mark as YES, fill in the circle for the number of DAYS in a typical week you did these activities and the AVERAGE TIME per day in hours and/or minutes you did these activities.

Intensity Levels:
Light → easy effort
Moderate → harder than light but not all-out effort
Heavy → all-out effort

Example:
Conditioning Activities
Moderate Effort::
Low impact aerobics, slow bicycling, rowing, leisurely swimming, health club machines - moderate intensity

Y N	Days/Week	Hours/Day	Minutes/Day
Y ● N ○	1 ○ 2 ○ 3 ● 4 ○ 5 ○ 6 ○ 7 ○	1 ● 2 ○ 3 ○ 4 ○ 5 ○ 5+ ○	5 ○ 15 ○ 30 ● 45 ○

In this example, the activity was done 3 days per week, 1 hour and 30 minutes per day.

SOCIAL NETWORK SURVEY

SEE CHAPTER 10 FOR DETAILS

Participant ID #	Acrostic	Date of Interview	Staff ID #
		__/__/____ Mo Day Year	

SAHELI SOCIAL NETWORK

Visit: Baseline 3 month 6 month

SAHELI-Net: Social and cultural influences on dietary patterns and physical activity

Overview

The purpose of this interview is to better understand how social relationships affect beliefs, attitudes and behaviors, and how these relationships affect physical and mental health. We will ask about your own and your social network members' health attitudes, beliefs, and behaviors.

As a reminder, your participation in this study is completely voluntary. You may decide to skip questions you prefer not to answer or end the survey at any time. Your decision to participate or not will in no way affect your future treatment or participation in research at this facility. We are very grateful for your assistance with this project.

We hope you will answer as many questions as you can, because your answers will help us to understand how social relationships influence health-related behaviors. This information will be used to design specific programs to directly improve community health.

Do you have any questions before we get started? Is it okay to begin?

Introduction:

"Now we are going to ask you some questions about your relationships with other people. We will begin by identifying some of the people you interact with on a regular basis. You do not have to give me the names of these people, simply their initials."

"From time to time, most people discuss things that are important to them with others. For example, these may include good or bad things that happen to you, problems you are having, or important concerns you may have. Looking back over the last 12 months, who are the people with whom you most often discussed things that were important to you? (**Prompt if do not know:** This could be a person you tend to talk to about things that are important to you. This can include your family, friends, relatives, neighbors, co-workers or anyone else you think you can discuss important matters about yourself.")

Roster A: **Initials of Persons:**

1.	5.	7.	9.
2.	6.	8.	10.

(**Prompt:** "Is there anyone else that you discuss important matters about yourself?")

HEART DISEASE QUESTIONS

Purpose: This is designed to understand the existing knowledge and perceptions of heart disease among South Asians. Perceptions are measured using a scale ranging from Strongly agree to Strongly disagree. Knowledge is measured based on the response to questions on heart disease prevention and risk factors for heart disease.

Participant ID #	Acrostic	Date of Interview	Staff ID #
		Mo / Day / Year	

SAHELI HEART DISEASE QUESTIONS

Visit: ○ Baseline 3 Month 6 Month

Coordinator Instructions: Read the question and answers (except for #10 on page 4, write the response given). Circle or fill in the answer given by the participant.
Read these instructions to the participant: The following are some questions about heart disease.

Perceptions

	Strongly agree	*Agree*	*Neither agree nor disagree*	*Disagree*	*Strongly disagree*
1. Asian Indians and Pakistanis are more likely to have heart attacks compared to people from other communities	1	2	3	4	5
2. Regular exercise can reduce my chances of having a heart attack	1	2	3	4	5
3. An older person does not need to exercise	1	2	3	4	5
4. If your heart starts to beat fast while you are exercising, you should stop.	1	2	3	4	5
5. Men need more exercise than women	1	2	3	4	5
6. Eating fruits, vegetables and whole grains will help prevent a heart attack	1	2	3	4	5
7. Eating less fat and salt will help prevent a heart attack	1	2	3	4	5
8. Being overweight increases my chances of having a heart attack	1	2	3	4	5

Participant ID #	Acrostic	Date of Interview	Staff ID #
		___/___/___ Mo Day Year	

SAHELI HEART DISEASE QUESTIONS

Visit: ○ Baseline 3 Month 6 Month

Knowledge

1. True or False: A person who has high blood sugar or diabetes is more likely to have a heart attack.
2. Major risk factors for heart disease and stroke include:
    1. High cholesterol
    2. Chewing or smoking tobacco (e.g. cigarettes, gutkha, bidi)
    3. Family history of heart disease
    4. All of the above
3. True or False: Everyone's body makes cholesterol.
4. A person with high blood pressure:
    1. Has high cholesterol
    2. Has a higher risk of stroke and heart attack
    3. Has a nervous condition
    4. Has a fast heart beat
5. Which blood pressure level is too high?
    1. 130/80
    2. 140/90
    3. 150/85
    4. Both b & c
6. How much exercise is recommended to keep your heart healthy?
    1. 20 minutes every day
    2. 90 minutes a day once you are in shape
    3. 30 minutes everyday
    4. It depends on your age
7. True or False: Medium or fast exercise keeps your heart strong and prevents a heart attack
8. Which of these people should exercise regularly? SHOW PARTICIPANT PAGE 5

1. 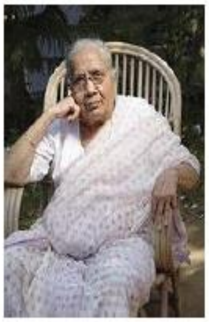
2. 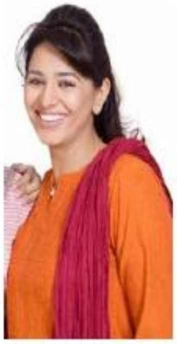
3. 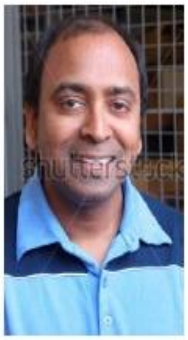
4. All of the above

EXERCISE CONFIDENCE SURVEY

Purpose: Will help understand how sure the participants feel that they will be able to follow the exercise routine.

Participant ID #	Acrostic	Date of Interview	Staff ID #
☐☐☐☐☐	☐☐☐☐	☐☐/☐☐/☐☐☐☐	☐☐☐

SAHELI EXERCISE CONFIDENCE SURVEY

VISIT: BASELINE 3 MONTH 6 MONTH

Below is a list of things people might do while trying to increase or continue regular exercise. We are interested in exercises like running, swimming, brisk walking, bicycle riding, aerobics classes or yoga.

Whether you exercise or not, please rate how confident you are that you could really motivate yourself to do things like these consistently, for at least six months.

Please circle one number for each question.

How sure are you that you can do these things?

	I know I can		*Maybe I can*		*I know I cannot*	*Does not apply*
1. Get up early, even on weekends, to exercise.	1	2	3	4	5	(8)
2. Stick to your exercise program after a long, tiring day at work.	1	2	3	4	5	(8)
3. Exercise even though you are feeling depressed	1	2	3	4	5	(8)
4. Set aside time for a physical activity program; that is, walking, jogging, swimming, biking, or other continuous activities for at least 30 minutes, 3 times per week.	1	2	3	4	5	(8)
5. Find a friend, family member, or group to exercise with.	1	2	3	4	5	(8)
6. Stick to your exercise program when your family is demanding more time from you.	1	2	3	4	5	(8)
7. Stick to your exercise program when you have household chores to attend to.	1	2	3	4	5	(8)

COPE SURVEY

PURPOSE :

The COPE survey helps identify the processed that people use in coping with stressful situations. In this study it will help us understand how the South Asian community copes with stressful encounters of everyday living. In a variety of studies, researchers have used it to investigate the components and determinants of coping.

Participant ID #	Acrostic	Date of Interview	Staff ID #
☐☐☐☐☐	☐☐☐☐	☐☐/☐☐/☐☐☐☐	☐☐☐

SAHELI STUDY

COPE QUESTIONNAIRE

Baseline 3 Month 6 Month

Introduction: There are lots of ways to try to deal with stress. Indicate what you generally do and feel, when you experience stressful events. Please circle the correct response.

	All of the time	*Most of the time*	*Sometimes*	*Rarely*	*Never*	*Does not apply*
1. I try to get advice from someone about what to do.	1	2	3	4	5	(8)
2. I put my trust in God.	1	2	3	4	5	(8)
3. I make a plan of action	1	2	3	4	5	(8)
4. I just give up trying to reach my goal	1	2	3	4	5	(8)
5. I try to come up with a strategy about what to do.	1	2	3	4	5	(8)
6. I look for something good in what is happening.	1	2	3	4	5	(8)
7. I do something I enjoy to take my mind off the stress.	1	2	3	4	5	(8)
8. I ask people who have had similar experiences what they did.	1	2	3	4	5	(8)
9. I try to find comfort in my religion.	1	2	3	4	5	(8)
10. I talk to someone about how I feel.	1	2	3	4	5	(8)
11. I do what has to be done, one step at a time.	1	2	3	4	5	(8)

TOBACCO USE QUESTIONAIRRE

To determine the use of tobacco products in the study participants.

Participant ID #	Acrostic	Date of Visit	Staff ID #
		Month / Day / Year	

TOBACCO USE

Coordinator Instructions: Read these instructions to the participant.
The following questions are about your use of tobacco. They will help us better understand the role of tobacco use in the risk of cardiovascular disease.

1. Have you smoked at least 100 cigarettes in your lifetime?
   - O Yes
   - O No ⟶ ***skip to Q. 6***
2. How old were you when you first started smoking cigarettes?
   - ☐☐ years old
3. Have you smoked cigarettes in the last 30 days?
   - O Yes ⟶ ***skip to Q. 5***
   - O No
4. FOR FORMER SMOKERS: How old were you when you quit smoking cigarettes?
   - ☐☐ years old
5. On average, about how many cigarettes a day do/did you smoke?
   - ☐☐☐ cigarettes per day
6. Have you ever used any other tobacco products? (E.g. cigars, pipes, snuff, chewing tobacco, bidis)
   - O Yes
   - O No

Accelerometer instructions and handouts

After the completion of the questionnaires the participants will be given instructions to use the accelerometer. The accelerometers will be loaned and an appointment will be scheduled for the return of the accelerometer.

Participants will be informed that they will receive their test results when they come after one week to return the accelerometer.

See Chapter 8 for accelerometer instructions

REPORTING OF TEST RESULTS WHEN PARTICIPANTS COME TO RETURN THE ACCELEROMETER

Giving Participants Results of Visit Measurements and Lab Values

The participant will be given anthropometry and blood pressure results when they come to return their accelerometers, a week after the baseline visit. See Appendix 1C.
The blood test from quest and physical exam results will be ready in an envelope when they are scheduled to come for their appointment for returning their accelerometer.

ALERTS AND ABNORMAL RESULTS

The purpose of defining medical alerts is to make sure that the participant and his/her physician are aware of any significant medical findings that arise as a result of the SAHELI exam.

Definitions

1. Alert: Any of the medical findings, listed in Table 1 that may have adverse health consequences to the participant if untreated.

2. Immediate Referrals: Medical emergencies which require immediate notification of both the participant and his/her primary physician. Participants receiving immediate referrals should be considered as those who would go directly from the Clinical Site to their physician or hospital. Immediate notification of the participant should occur during the clinic visit. Immediate notification of the participant's physician should be accomplished by telephone, to be completed before the participant leaves the clinic. A follow-up letter documenting information discussed by phone should also be sent to the participant's physician for findings included in the initial report, or immediately upon receipt from the central agency for findings on the later reports.

3. Urgent Referrals: Urgent referrals are made for abnormalities detected which require medical attention but not on an emergency basis. Urgent notification of the participant should occur before the participant leaves the clinic (for findings included in the initial participant report), or immediately upon receipt from the central laboratory (for findings on later reports). Urgent notification of the participant's physician should be sent within the week.

Table 1. SAHELI Alerts and Alert Levels

Finding	Alert Level
Systolic BP > 210	Immediate
Diastolic BP > 120	Immediate
180 < Systolic BP < 211	Urgent
110 < Diastolic BP < 121	Urgent
Total cholesterol > 360 mg/dL	Urgent
Triglyceride > 1000 mg/dL	Urgent
HDL cholesterol < 20 mg/dL	Urgent
Calculated LDL cholesterol > 260 mg/dL	Urgent
Fasting glucose < 50 mg/dL or > 400 mg/dL	Urgent

Methods

1. General Instructions

Whenever one of the alerts listed above is identified for a participant, the actions defined under Immediate or Urgent Referrals, above, must be completed. Actions taken must be documented with a copy of the letter to the participant archived in the participant file.

2. Specific Instructions for Urgent or Immediate Referrals
   - 2.1 If an "Urgent" or "Immediate" Referral result occurs, the Study Coordinator should notify the NU PI as soon as possible.
   - 2.2 The NU PI should telephone the participant within 24 hours and instruct the participant to contact their primary care provider for follow-up immediately.
   - 2.3 The NU PI should document the date and time of the telephone call in the participant's file.
   - 2.4 A letter and a copy of the test should be mailed to the participant. (Appendix 3C)

If any other actions were taken or unusual circumstances were involved, please document this in the participant file.

3. "Other" alerts: If any unusual incidents occur in the clinic, please document this in the participant file. These should include anything that would be important to have documented later, including a participant fall in the clinic, participant feeling dizzy or fainting during the blood draw, etc. If in doubt, document!

BASELINE VISIT CHECKLIST

Purpose: The BASELINE VISIT CHECKLIST provides an outline of all procedures that should be completed during the Visit. After completion of each component of BASELINE VISIT, staff will complete the BASELINE VISIT CHECKLIST.

The BASELINE VISIT CHECKLIST gives a suggested order of procedures for the visit. Since the DEMOGRAPHICS form provides important information that will be used in a report to the grantor agency, it is **required** that this be the very first form completed during the study visit. It is highly recommended that the interviewer follow the order of the checklist for the remainder of the visit.

Procedure: Complete and check for accuracy and missing information.

Filling out the form:

- Indicate participant status at the top of this form. If the interviewer is unable to contact the candidate to complete Visit 1, the interviewer must use his/her judgment to decide the appropriate time to indicate participant status as "Visit 1 scheduled but did not show."

- If a participant completes the visit, make sure all procedures on the checklist were marked "Yes." *Do not leave any fields blank.*

- If the participant becomes ineligible at any point during the visit, check off which procedures were fully or partially done by marking "Yes." Mark "No, other" for the rest of the incomplete procedures. *Do not leave any fields blank.*

Participant ID #	Acrostic	Date of Interview	Staff ID #
☐☐☐☐☐	☐☐☐☐	☐☐ / ☐☐ / ☐☐☐☐<br>Mo Day Year	☐☐☐

SAHELI VISIT CHECKLIST

BASELINE 3 MONTH 6 MONTH

Coordinator Instructions: For checklist items, mark only one bubble per line. *Do not leave any lines blank.* Items in **bold** are teleforms therefore all pages must be faxed in if procedure was marked as done.

Participant Status from Visit 1:

O Visit completed
O Visit not completed, specify reason ____
O Visit scheduled but did not show

PARTICIPANT ID# ☐☐☐☐☐☐

Item	Please mark if done			Comments
	Yes	No	Not applicable	
**PRIOR TO THIS VISIT**				
1. Print forms with Participant ID & Acrostic	O	O	O	
**AT THE VISIT**				
2. Review and sign Informed Consent	O	O	O	
3. Review and sign HIPAA	O	O	O	
4. Review Participant Contact Information	O	O	O	
5. Demographics	O	O	O	
6. Urine pregnancy test (if applicable)	O	O	O	
7. Medication Questionnaire	O	O	O	
8. Medication Inventory Form(s) (if applicable)	O	O	O	
9. Seated Blood Pressure	O	O	O	
10. Urine/Phlebotomy	O	O	O	
11. Fasting Blood Draw	O	O	O	Time: ___ : ___ AM/PM*<br>Enter draw time on the Quest Lab Form.

APPENDIX 4A: APPOINTMENT LETTER

South Asian Heart Disease Prevention Study
Instructions for the visit

CLINIC APPOINTMENT REMINDER
Appointment Date: 01/07/2010
Time: 8:15 am

Begin Fasting Date: 01/08/2010
Time: 8:15 pm
(10 hrs before appointment)

Date:

Dear Mr/Ms.__________,

Thank you very much for agreeing to participate in the “Translating a heart disease lifestyle intervention into the community” study. You visit is scheduled at Metropolitan Asian Family Services on ____________, **Date**________ at **time**_____. Directions from your residence to the facility are attached.

Below are some items to keep in mind prior to your visit:

1. You will have a fasting blood test during your visit. **Do not eat anything after 10 p.m. the day before your visit.** You will not be eligible for blood test if you do not fast.
2. If you take diabetes medicine, **do not take your diabetes medicine the morning of your visit.**
3. Take all other medicine.
4. We ask that you **bring all current medications to the visit.**
5. If you have a doctor and you want your results sent to your doctor, bring your doctors information.
6. Wear comfortable cloths.
7. If you need glasses for reading, please bring them with you.
8. If you are ill, please call us at (312) 503-6995 to reschedule your visit.

1

APPENDIX 4B : PARTICIPANT CONTACT INFORMATION

Participant ID #	Acrostic	Date of Interview	Staff ID #
☐☐☐☐☐	☐☐☐☐	☐☐/☐☐/☐☐☐☐<br>Mo Day Year	☐☐☐

SAHELI PARTICIPANT CONTACT INFORMATION

Name: ____

DOB: _________ / ________ / __________
Month Day Year

Marital Status: ☐ Single ☐ Married ☐ Divorced ☐ Widowed

Preferred language: ☐ English ☐ Hindi ☐ Urdu ☐ Other: ______________

Address: ______________________________ **Apt./Room #:**_______

City: ________________________ **State**: _______ **Zip Code:** ___________

Day Phone: **(____)** _____ **-** ________ **Evening Phone:** **(____)** _____ **-** ________
Area Code Number Area Code Number

Mobile Phone: **(____)** _____ **-** ________ **Email:** ___________________________
Area Code Number

Best time to call: ____________________ OK to leave message? ☐ Yes ☐ No

Preferred name: ____

1. **Do you plan to change your name within the next year?**
   ○ Yes → If yes, what will your new last name be? ____________________
   ○ No

2. **Do you plan to be out of this area for an extended period of time (a month or longer) within the next year?**
   ○ Yes → If yes, approximately when will you leave and when will you return?
   Departure: _________ / ________ Return: _________ / ________
   Month Year Month Year

   ○ No → If no, continue to the next question.

CONFIDENTIAL- Do not distribute, developed by Northwestern University

APPENDIX 4C : EXAMINATION RESULTS FORM

The South Asian Heart Disease Prevention Study

Physical Exam and Blood test Results

Date:________ ID # ____________________

Dear ____________________

We appreciate your participation in The South Asian Heart Lifestyle Intervention Study. The purpose of this letter is to provide you with some of the results of your examination.

HEIGHT: __________feet________inches

WEIGHT: _________pounds

BODY MASS INDEX: ___________**kg/m²**

A body mass index of 25 kg/m² or greater is considered overweight, and 30 kg/m² or greater is considered obese by the National Institutes of Health.

WAIST:___________inches

Normal waist measurement for men < 35 inches

Normal waist measurement for women < 31 inches

BLOOD PRESSURE: ___________**mmHg**

These values indicate that your blood pressure is:

□ Normal (SBP < 120 and DBP < 80)

□ Borderline Elevated (SBP 120-139 or DBP 80-90)

□ Elevated (SBP > 140 or DBP > 90)

BLOOD TEST RESULTS: (See Quest lab results enclosed)

Some of your blood test results that were out of normal range were:

1. xxx
2. xxx

We recommend that you discuss any abnormal results with your doctor.

Sincerely,

Namratha R. Kandula

Namratha Kandula, MD, MPH

Principal Investigator

Northwestern University

CHAPTER 5: RANDOMIZATION

WHAT IS RANDOMIZATION

Randomization is a process used in research studies to test the effectiveness of a new intervention (for example: a new medication or a new program) compared to a standard intervention. Participants will usually be evenly placed in different study groups. Participants do not have control over which group they may be assigned to. People who take part in studies requiring randomization are generally placed in a group based on chance, not choice. Some participants are placed in the “experimental” group, and the others are placed in the “control” group. The control group will receive standard treatment, a placebo, or other intervention based on the design of the study. In some studies the participants do not know to which group they have been assigned. This process, called randomization, allows researchers to compare results between the two groups. Randomization helps us increase our knowledge about which treatments or programs are better than others.

SAHELI STUDY PROCESS OF RANDOMIZATION

In this study we will be randomly assigning equal number of men and women into the control group and intervention group. Participants in both groups receive results of their blood work and referral to a primary care physician if they do not have one. The control group is the group that will receive printed health education materials and the intervention group will receive the group education classes.

To understand how this works, imagine that our total goal for enrollment in the study is 80 people. Each participant is randomly assigned to either the intervention group or the control group. To do this, we use a computer database program which is similar to “coin toss”. Half of the people are assigned to the intervention group and half are assigned to the control group. We can then compare the results of the two groups to see which type of health education works better. We also try to figure out why it works better.

The program uses a Visual Basic for Applications (VBA) random number generator to produce customized sets of random alphanumeric characters.

The randomization process in the study works in the following ways.

MAFS staff will call people who are interested to participate in the study

↓

Interested participants will be invited to the baseline screening visit

↓

Participants test results will be reviewed by Dr. Namratha Kandula

↓

Participants will return their accelerometers 7 days after baseline visit

↓

Check if participants have enough valid hour/days accelerometer data in order to participate

↓

Once we reach the target number of people (n=16) to initiate a cohort

↓

We use the database to randomly assign all 16 people into either the control or intervention group.

↓

Randomization will be complete. No changes can be made

↓

In case a person is not interested in participating in the study after randomization

↓

Inform Dr. Namratha Kandula

↓

Only Dr. Namratha Kandula can authorize a deletion of record after any participant is assigned in the study. She will contact Andrew Cooper

CHAPTER 6: ANTHROPOMETRY

OVERVIEW

The purpose of the anthropometry procedures is to acquire general measures of body size, shape, and obesity.

MATERIALS AND EQUIPMENT

- Stadiometer (Accu-Hite Measure Device with level bubble) (height ruler with triangle level is used at some centers)
- Balance Scale in lbs/kg OR Digital Scale
- Gulick II 150 cm anthropometric tape
- Full length mirror
- ANTHROPOMETRY FORM (Appendix 7A)

METHODS

General Instructions

For all measurements, participants should wear light clothing but no shoes (thin socks or “pillow slippers” are OK). Have participants completely empty their pockets and remove excessive amounts of jewelry that could affect the weight measurement. Provide lockers with locks for valuables.

Pregnant women should not be measured, regardless of gestational stage (check exclusion criteria for pregnancy). The Clinic Coordinator should ascertain pregnancy status, both for measurements and for subsequent coronary calcification measurement.

Take a single measurement at each body site and record on the anthropometry form using specific rounding rules for each procedure. Record any modifications in measurement techniques (e.g. height decreased from a hunched posture or weight that exceeds the capacity of the scale).

- Make sure to verify participant ID and acrostic
- Verify or record date of the procedure at the top of the form
- Enter Staff ID# of the staff member who is completing the measurement
- Fill in visit bubble 1.

Standing Body Height

Equipment
- Stadiometer (Accu-Hite Measure Device with level bubble)

Preparation
- Before measuring height, make sure the floor is level, the wall is at a 90 degree angle to the floor, the wall is straight, and the Stadiometer is mounted perpendicular to the floor.

Measurement
For accurate measurement of height, the participant must be standing in a vertical plane. To achieve this position, have the participant stand erect on the floor or horizontal platform, with back against the vertical

Stadiometer, heels against the wall, and feet or knees together—whichever come together first. Have the participant look straight ahead, with head in the Frankfort horizontal plane (Figure 1).

- Place the headboard over the crown of the head, with the headboard forming a right angle to the scale. The headboard should touch the scalp lightly.
- Ask the participant to step out from under the headboard. Record the participant's height to the nearest 0.1 centimeter in Box 1a of the ANTHROPOMETRY FORM.

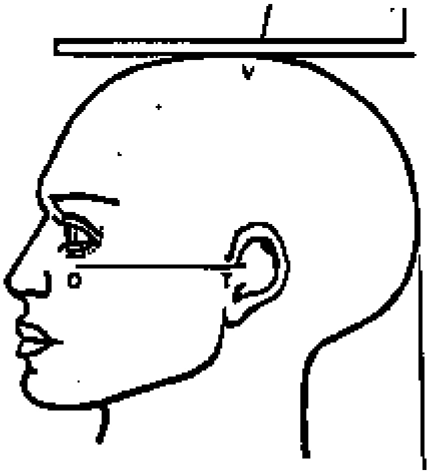

The Frankfort Plane includes the lower margin of the bony orbit (the bony socket containing the eye) and the most forward point in the supratragal notch (the notch just above the anterior cartilaginous projections of the external ear)—also referred to as the upper margin of the external auditory meatus (the hole in the ear).

Figure 1. Frankfort Plane for Measuring Body Height

- If you are unable to measure the actual height of the participant because the headboard does not rest directly over the scalp, estimate height to the nearest 0.1 cm, record in Box 1a of the ANTHROPOMETRY FORM and answer "yes" to question 1b. "Was there a modification in protocol?"
- Record the results, to the nearest tenth (0.1) of a cm, in question1a on the ANTHROPOMETRY FORM.
- If any modification was made to obtain height, record "yes" to the question, "Was there a modification in protocol?"

Completing the Data Form for Height
Question 1.a. Measure the participant's height to the nearest tenth of a centimeter.

Question 1.b. Any modification in measurement techniques should be specified by filling in the "yes" bubble, and will be taken into consideration for data analysis.

1. Height

a. ☐☐☐.☐ CM

b. Was there a modification in protocol? ○ Yes ○ No

Body Weight

Equipment

- Digital Scale or Balance Scale in lbs/kg

Preparation

- Always balance the scale so that the indicator is at zero when no weight is on the scale. The scale should be on a firm, level surface (not on a carpet, for example).

Measurement

A. Procedures Using Digital Scale
  - Instruct the participant to stand in the middle of the platform of the scale, with head erect and eyes looking straight ahead. Adjust the weight on the indicator until it is balanced. Record the results, to the nearest 0.1lbs, in Box 2a.
  - Record the weight in 0.1 pounds as indicated on the digital display
  - Ask the participant to step off the scale and check that the digital display returns to zero

B. Procedures Using Balance Beam Scale
  - Be sure the scale is balanced so that the indicator is at zero when no weight is on the scale.
  - The scale should be level and on a firm surface (not a carpet).
  - Instruct the participant to stand in the middle of the platform of the scale, with head erect and eyes looking straight ahead. Adjust the weight on the indicator until it is balanced. Record the results, to the nearest 0.1lbs, in Box 2a.
  - Adjust the weight on the indicator until it is balanced.
  - Record the weight in 0.1 pounds as indicated

C. Protocol Modifications
  - If the participant is too obese to stand securely on the scale’s platform when looking straight ahead, he/she may stand sideways on the scale to take the weight measurement; facing to the side rather than the front will provide the participant a wider base and more stability.
  - If a participant has a prosthetic limb or breast prosthesis, measure weight with the prosthesis on.
  - If a participant is frail or unsteady, measure weight while participant is lightly steadied by you or an assistant.
  - If a participant is unable to stand on the scale for a weight measurement, do not attempt a weight measurement.
  - If any modification were made to obtain weight, record “yes” to the question 2b., “Was there a modification in protocol?”

Completion of Data Form for Weight

Question 2.a. The measurement should be recorded to the nearest one tenth of a pound on the ANTHROPOMETRY form Question 2.a.

Question 2.b. Any modification in measurement techniques should be specified by filling in the “yes” bubble, and will be taken into consideration for data analysis.

2. Weight

a. ☐☐☐.☐ lbs

b. Was there a modification in protocol? ○ Yes ○ No

Girth Measurements

Equipment
- Gulick II 150 cm anthropometric tape
- Full length mirror

Technique
Do not take abdominal and hip girth measurements over loose clothing. It is ok if taken over light well-fitted clothes.

Abdominal Girth
- Use the Gulick II anthropometric tape
- Move to the participant's right side to take the measurement; do not take this measurement from the front. Be sure to keep the tape horizontal while making the measurement; use the wall-mounted mirror to assure horizontal placement on all sides.
- Take the measurement tape around the abdomen horizontally at midpoint between highest point of the iliac crest and lowest part of the costal margin in the mid-axillary line.
- Mark the midpoint on both sides using a washable marker.
- The participant may be asked to assist passing the tape around the abdomen by holding the end of the tape in position
- When the tape is positioned in the horizontal plane at the correct height, the participant should be asked to keep their arms at their side and breathe in and out naturally, holding at the end of a normal exhalation.
- Be sure to keep the tape horizontal while making the measurement; use the wall-mounted mirror to assure horizontal placement on all sides.
- For participants with a slim abdomen, this horizontal plane will be at the level of the umbilicus---for those with a larger abdomen, the umbilicus will likely be lower.
- Round abdominal girth measurement to the nearest 0.1cm and record in Box 3a, measurement 1.
- Repeat the abdominal girth measurement and record in Box 3a, measurement 2.
- If the circumference exceeds 150 cm, record "yes" for the question, "Was there a modification in protocol?"

Hip Girth
- Take the hip girth measurement from the participant's right side; do not take this measurement from the front. Instruct the participant to stand with his/her feet together. Measure hip girth at the maximum circumference of the buttocks. Check to see that the tape is level in front and back.
- Round hip girth measurement to the nearest 0.1cm and record in Box 3b, measurement 1.
- Repeat the hip girth measurement and record in Box 3b, measurement 2.
- If the circumference exceeds 150 cm, record "yes" for the question, "Was there a modification in protocol?"

Completion of Data Form for Girth
Question 3a: Enter first and second waist circumference measurements in centimeters.

Question 3b: Enter first and second hip circumference measurements in centimeters.

Question 3c: Any modification in measurement techniques should be specified by filling in the "yes" bubble, and will be taken into consideration for data analysis.

3. Girths

a. Waist: Measurement 1 [ ][ ][ ] . [ ] CM Measurement 2 [ ][ ][ ] . [ ] CM

b. Hip: Measurement 1 [ ][ ][ ] . [ ] CM Measurement 2 [ ][ ][ ] . [ ] CM

c. Was there a modification in protocol? ○ Yes ○ No

Comments/Modifications to the Protocol

If you have comments or if there have been modifications to the protocol as described above, answer “yes” to question 4 on the ANTHROPOMETRY FORM and record comments in the space provided. If there are no comments or modifications, answer “no” to question 4.

4. Comments/modification to the protocol?

○ Yes ○ No

Please specify:

QUALITY CONTROL

All SAHELI Staff performing anthropometry measurements must be trained and certified. Please see Chapter, Quality Control.

ROUNDING RULES

Round decimals to the nearest tenth (rounding position) using standard rounding rules:

- If the number to the right of the rounding position is 5 or greater, increase the number in the rounding position by 1 (round up).
  - Example 1: 90.88 should be rounded to 90.9.
  - Example 2: 99.99 should be rounded to 100.0
- If the number to the right of the rounding position is less than 5, leave the number in the rounding position alone (round down).
  - Example 1: 90.44 should be rounded to 90.4
- If the value is exactly between two such units, round up if the lower unit is even and round down if the lower unit is odd
  - Example 1: if weight is 150.55 pounds, record as 150.5 pounds,
  - Example 2: if weight is 150.65 pounds record as 150.7 pounds

APPENDIX 6A: ANTHROPOMETRY DATA FORM

SAHELI STUDY

Participant ID #	Acrostic	Date of Visit	Staff ID #
☐☐☐☐☐	☐☐☐☐	☐☐ / ☐☐ / ☐☐☐☐<br>Month Day Year	☐☐☐

ANTHROPOMETRY

VISIT: ○ 1 ○ 2 ○ 3 ○ 4 ○ 5

Coordinator Instructions: Please use a black pen and fill in bubbles completely.

1. Height
   a. ☐☐☐.☐ CM
   b. Was there a modification in protocol? ○ Yes ○ No

2. Weight
   a. ☐☐☐.☐ lbs
   b. Was there a modification in protocol? ○ Yes ○ No

3. Girths
   a. Waist: Measurement 1 ☐☐☐.☐ CM Measurement 2 ☐☐☐.☐ CM
   b. Hip: Measurement 1 ☐☐☐.☐ CM Measurement 2 ☐☐☐.☐ CM
   c. Was there a modification in protocol? ○ Yes ○ No

4. Comments/modification to the protocol?

   ○ Yes ○ No

   ↓

   Please specify: ☐☐☐☐☐☐☐☐☐☐☐☐☐☐☐☐☐☐

CHAPTER 7: BLOOD PRESSURE

OVERVIEW

Blood pressure (BP) level is a major risk factor for coronary heart disease, congestive heart failure, and stroke. The purpose of a specific measurement protocol, or training and certifications of technicians, and of ongoing quality control is to minimize variability due to known exogenous factors and to reduce imprecision and biases in measurement. The Dinamap® automated device will continue to be used for consistency and to reduce the potential for observer biases.

In addition, measurements of lean body mass and fat mass will be measured by bioelectrical impedance. The purpose is to provide a more accurate and direct measure of body fat than can be provided by calculation of the body mass index.

MATERIALS AND EQUIPMENT

- OMRON HEM -907XL sphygmomanometer
- 4 cuffs
- Gulick II tape measure (for arm circumference).
- Measuring tape
- Alcohol wipes
- Tissues
- Water soluble markers
- Watch or stop watch (to time five-minute rest and resting heart rate).
- SEATED BLOOD PRESSURE form (Appendix 8A)

CLASSIFICATION OF THE PARTICIPANT'S BLOOD PRESSURE WITHIN THE JNC VI CATEGORIES AND CRITERIA FOR ALERTS AND REFERRALS

This classification and the criteria for alerts have not changed from Exam 1. However, they are important and are reiterated here.

The 1997 Report of the Joint National Committee on Detection, Evaluation, and Treatment of High Blood Pressure (JNC VI) defines categories of blood pressure and recommends follow-up according to the following criteria:

Table 1. Classification of BP in Adults Aged 18 Years or Older*.

BP Category	SBP (mm Hg)		DPB (mm Hg)	Action
Optimal	<120	and	<80	Recheck in 2 years
Normal	<130	and	<85	Recheck in 2 years
High-normal	130–139	or	85–89	Recheck in 1 year
Hypertension**				
Stage 1	140–159	or	90–99	Refer within 2 months
Stage 2	160–179	or	100–109	Refer within 1 month
Stage 3	>180	or	>110	Refer within 1 week or immediately

* When recommendation for follow-up of DBP and SBP are different, the shorter recommended time for recheck and referral should take precedence. This classification applies only to participants not taking antihypertensive drugs.

** Diagnosis of hypertension must be based on two or more readings taken at each of two or more visits following an initial screening.

SBP= systolic blood pressure. DBP= diastolic blood pressure.

1. Alert levels requiring **immediate referral** (send participant directly to a physician or hospital) for SAHELI participants are:

   - **Systolic BP >210 mm Hg**
   - **Diastolic BP >120 mm Hg**

2. Alert levels requiring *urgent referral* (*within one week*) are:

   - *Systolic BP 180–210 mm Hg*
   - *Diastolic BP 110–120 mm Hg*

3. Alert levels requiring follow-up within two months time, and, therefore, we recommend physician notification for systolic or diastolic BP above these levels.

   - BP >140/90 mm Hg

4. JNC VI states that blood pressure classifications and referral recommendations are based on the average of two or more readings on two or more occasions. In SAHELI we intend to use the average of the 2nd and 3rd blood pressure readings (see below) in order to reduce the impact of reactivity (higher first reading) on the estimate of the value of the underlying blood pressure. Thus, in deciding whether a participant meets criteria for an alert level, the average of the 2nd and 3rd readings should be used. This will require on-the-spot arithmetical manipulation of the systolic and diastolic values. A hand calculator may be useful. The data forms include fields for these averaged values and for any actions taken.

METHODS

Preparation

A. Complete the banner of the SEATED BLOOD PRESSURE form during the five-minute rest period.

- Verify participant ID and acrostic is printed correctly.
- Record the date of the procedure.
- Enter Staff ID# of the staff member who is completing the measurement

INSERT FORM

SAHELI STUDY

Participant ID #	Acrostic	Date of Visit	Staff ID #
		Month / Day / Year	

SEATED BLOOD PRESSURE

VISIT: Baseline 3 Month 6 Month

Coordinator Instructions: Please use a black pen and fill in bubbles completely.

Dinamap number:

Equipment and Supplies

Equipment
OMRON HEM -907XL sphygmomanometer
4 cuffs
Gulick II tape measure
Foot stool

Supplies
Wipes
Alcohol
Tissues
Water soluble ink pens
Gauze (4x4)

Blood Pressure Measurement Procedures

Position Participant for Blood Pressure Measurements

Ask the participant to sit in the chair, adjust the chair if necessary and rest quietly for 5 minutes prior to blood pressure measurement. The health interviewer will explain the exam to the participant using a script: “Before taking your first blood pressure reading, there will be a 5 minute waiting period. When I inflate the cuff, it may feel tight and you will feel some pressure on your upper arm. While we are

measuring your blood pressure, we ask you not to talk and I will not talk either because talking and moving changes your blood pressure. Do you have any questions?"

The right arm and back should be supported and the legs should be uncrossed with both feet flat on the floor. The right arm should be bared and unrestricted by clothing with the palm of the hand turned upward and the elbow slightly flexed.

The arm should be positioned so that the midpoint of the upper arm is at the level of the heart. Small or short participants may have to raise their body to the correct position by changing the chair position up or down. If necessary, especially with short participants, place the participant's feet on the footstool to stabilize their feet in a flat position. Very tall participants may need to place their arm on a book or pillow to bring their upper arm to the correct position.

For the purpose of standardization, both pulse and blood pressure are measured in the right arm unless one of the following conditions applies:

1. The participant indicates that the blood pressure procedure should not use the right arm (for example, because of a history of mastectomy or fistula).

In the above condition, the blood pressure measurement must be taken in the left arm. Use of the right or left arm must be recorded on the blood pressure (BP) recording form. BP measurements are not done on any arm that has rashes, small gauze/adhesive dressings, casts, is puffy, has tubes, open sores, hematomas, wounds, arteriovenous (AV) shunt, or any other intravenous access device. Also, women who have had a unilateral radical mastectomy do not have their blood pressure measured in the arm on the same side as the mastectomy was performed. In all cases, if there is a problem with both arms, the blood pressure is not measured.

Locating the Pulse Points

Locate the brachial artery by palpation and mark the skin with a small dot, using a black pen. (The brachial artery is usually found just medial and superior to the cubital fossa posterior to the biceps muscle and slightly towards the body). For brachial artery palpitation, fingertips or thumb may be used.

Cuff Size Selection and Application

It is important to select the appropriate size cuff that properly fits the participant's arm. The length and width of the bladder inside the cuff should encircle at least 80 percent and 40 percent of an arm respectively. The index lines on the cuff are not used in this study. Using a centimeter tape, determine the midpoint of the upper arm by measuring the length of the arm between the acromion and olecranon process (between the shoulder and elbow).

Measurement of Arm Circumference

Have a participant remove his/her upper garment, or clear the upper arm area so that an unencumbered measurement may be made. If the upper garment is removed, a gown must be provided to the patient.

i. Have the participant stand, with the right arm hanging and bending the elbow so that the forearm is horizontal (parallel) to the floor.
ii. Measure arm length from the acromion (bony protuberance at the shoulder) to the olecranon (tip of the elbow), using the Gulick II anthropometric tape.

iii. Mark the midpoint on the dorsal surface of the arm.
iv. Have the participant relax the arm along side of the body.
v. Draw the tape snugly around the arm at the midpoint mark. NOTE: Keep the tape horizontal. Tape should not indent the skin.
vi. Pull the gulick II tape measure until one red ball is visible. This is the measurement of arm circumference that should be used.

Choosing the Correct Cuff Size

Identify the measured arm circumference under the column I in Table 1 below. Use the cuff size from column 2 associated with the arm circumference in column 1. (Example: If the arm circumference at midpoint is 36cm, use the large adult cuff marked CL19.)

Table 1.

Arm Circumference (cm)	OMRON Cuff Size
17.0 to 21.9	index 17-22cm (CS19)
22.0 to 32.5	index 22-32cm (CR19)
32.5 to 42.5	index 32-42cm (CL19)
42.6 to 50.0+	index 42-50cm (CX19)

Wrapping the Blood Pressure Cuff around the Arm

Position the rubber bladder with the “art” label on the bottom of the cuff just above the pen mark over the brachial artery pulse determined earlier at least one inch above the crease of the elbow. The cuff tubing should be at the outer (lateral) edge of the arm if the cuff is placed correctly.

For short or fat conical arms, if the cuff that matches the arm circumference is too wide to fit on the upper arm with space above the brachial artery pulse point at the cubital fossa then choose the next smaller cuff size and enter the cuff size chosen on the BP recording form.

Place the “artery” marker on the inner part of the cuff directly over the brachial artery. The cuff should be wrapped in a circular manner. Do not wrap the cuff in any spiral direction. Check the fit of the cuff to ensure that it is secure but not tight.

Procedure for the OMRON HEM-907XL

This protocol is written for use with the OMRON HEM-907XL automated blood pressure monitor. Special attention must be placed on assessment and maintenance of the instrument’s accuracy as per the manual that accompanies the instrument. The design and operation of the OMRON HEM-907XL are based upon the combined principles of compression of the brachial artery under an elastic, inflatable cuff and estimation of the systolic and diastolic blood pressure levels by detection of oscillometric waves.

Setting up the OMRON

At a start of each session: Check that the monitor is attached to the AC adapter to the DC jack and plugged in and AC sign is visible in the lower window.

When the power is OFF, push the ON/OFF (power) button for more than three seconds while holding the START button simultaneously: F1 is displayed in the first window and three inflation (3) is displayed in the middle window. If needed push the DEFLATION (deflation control)/Measurement Result Display Switch Button to change the set value to 3 inflations.

Push the START button and F2 function is displayed in the first window and 0 waiting time is displayed in the middle window.

If needed, push the DEFLATION (deflation control)/Measurement Result Display Switch Button and change the set value to 0 sec waiting time. Push the START button and F3 function is displayed in the first window and inflation interval 30 second time is displayed in the bottom window.

If needed, push the DEFLATION (deflation control)/Measurement Result Display Switch Button and change the set value to 30 sec measurement interval.

Table 2

Function #	Items to Set	Set Value
F1	Number of inflations	3 times
F2	Waiting time to start the first inflation	0 sec
F3	Inflation interval	30 sec

Measuring the Blood Pressure

Once these settings are ensured the exam can start. Turn off the OMRON by pushing the ON/OFF button. To measure blood pressure in the AVG mode, push the ON/OFF button to turn on the power. Set the MODE selection to AVG. Set the P-SET (inflation level) knob to AUTO.

Connect the air tube to the cuff.

For all cuff sizes, connect the air tube to the main unit by attaching the air plug to the base of the air connector. Connect the cuff to the air tube attached to the OMRON unit.
Wrap and secure the appropriate cuff to the participant's upper right arm as described above.

Push the START button to Start the Measurements
The cuff will inflate automatically and deflation will begin after the OMRON detects no osillometric waves. The dial will show sequentially in the bottom panel of the LCD screen. Three measures will be taken with 30 seconds between each.

After each inflation and deflation the systolic blood pressure, diastolic blood pressure and pulse rate will be displayed in the top, middle and bottom sections of the LCD screen.

After the first and second measurements are displayed there will be a preset 30 second interval before the beginning of the next measurement. During this time have the participant raise their cuffed arm all the way above their head for the count of 5 seconds and then return to the original resting position with the arm supported with the cubital fossa at heart level. Do not clench the fist. This action is to avoid venous congestion in the arm that may not have dissipated after inflation of the cuff – which in turn could increase the pressure recorded on subsequent measurements.

Recording the OMRON Results
After all the inflations are finished, each time the DEFLATION button is pushed the measurement results for each reading will be displayed along with a small display in the bottom window indicating the order of display: Avg, 1, 2, 3 inflations. Record all the systolic and diastolic blood pressure levels for the first, second, and third measurements separately on the Sitting Blood Pressure (SBP) form. Record the average systolic and diastolic blood pressure measurements and the average pulse rate, respectively.

Push the ON/OFF button. This terminates the exam and you are ready for the next participant.

The blood pressure results will be reviewed with the participant at the time of their discharge from the study visit. If the average blood pressure is equal to or greater than 200 mmHg systolic or equal to or greater than 120 mmHg diastolic the technician records the value in the data entry system, closes out the data entry screen per protocol and immediately page Dr. Namratha Kandula (pager 312-695-8630) or a covering physician. The health interviewer will arrange appropriate follow-up after speaking with Dr. Kandula or the covering physician, including accompanying the participant to the nearest emergency room for medical evaluation of the participant's blood pressure.
The participant's blood pressure values are not discussed at the blood pressure station nor during the measurement process. If pressed, the technician can add that the research protocol requires that results not be discussed during the examination. The OMRON display and the computer monitor should be turned away from the participant so that the blood pressure values being recorded are not easily visible.

Equipment Maintenance

The following sections specifically state the steps that technicians follow to check equipment and maintain equipment used for the technician examination.

OMRON HEM-907XL

Weekly – Wipe the monitor with a soft, damp cloth diluted with disinfectant alcohol, or diluted detergent. Complete cleaning by wiping the monitor with a soft, dry cloth.

Blood Pressure Cuffs: Check the inflation cuff for cleanliness, and wipe between each use with disinfectant wipes.

Calibration

Monthly calibration is required for the OMRON machine.

Weekly calibration consists of the comparing readings between ***THE PRESSURE GUAGE TO MAKE THIS COMPARISON HAS YET TO BE CHOSEN*** and the OMRON at the pressure of 0, 60, 100, 150, 200, and 250 mm Hg.

Instructions are listed in the following Sections:

Daily Check points

1. Check function settings on the OMRON machine (0 waiting, 3 inflations, 30 seconds interval between inflations)
2. Check Mode and P-setting on OMRON unit

3. Make sure that the AC adapter cord of the OMRON unit is securely plugged in (it has a tendency to get disconnected from the unit).
4. Check the OMRON unit AC adapter cord and tubing for cracks
5. Clean all the equipment

Glossary

Systolic BP is defined as the highest arterial blood pressure of a cardiac cycle occurring immediately after contraction of the left ventricle of the heart.

Diastolic BP is the lowest arterial blood pressure of a cardiac cycle occurring during the passive rhythmical expansion or dilation of the cavities of the heart during which they fill with blood.

Auscultatory Method detects sounds of pulsatile blood flow in the artery using a stethoscope held over the artery just below an inflation BP cuff. As the BP cuff gradually deflated, pulsatile blood flow is re-established and accompanied by sounds that can be detected by the stethoscope. The pulsatile sound corresponds to a reading of a mercury column (mercury sphygomomanometer) or a dial (aneroid) device connected to the BP cuff.

Oscillometric Method uses a transducer to measure the oscillations of pressure in the BP cuff corresponding to the pulsatile blood flow in the artery under the cuff. The oscillometric method is used by all automated BP machines.

Certification Checklist

Baseline and Follow-up Visits

BIOMARKER RISK ASSESSMENT IN VULNERABLE OUTPATIENTS

Sitting Blood Pressure Measurement

1. ☐ Makes sure that the arm is entirely exposed, asking the participant to change into a hospital gown if needed.
2. ☐ Asks patient to stand with the arm bent 90 degrees at elbow.
3. ☐ Uses Gulick tape measure to measure length of upper arm.
4. ☐ Measures length between the acromion (bony protuberance at top of shoulder located somewhat anteriorally) and the olecranon (tip of elbow).
5. ☐ Marks midpoint of that length with pen.
6. ☐ Asks patient to let the arm hang relaxed at side.
7. ☐ Uses Gulick tape measure to measure circumference of upper arm at the midpoint that was marked with the pen.
8. ☐ Pulls tape to tautness that causes exactly one red ball to show in the window indicator.
9. ☐ Records arms circumference on data page.
10. ☐ Notes which size blood pressure cuff to use and marks this on data page.
11. ☐ Asks patient to sit in chair and adjusts height of chair if needed. Patient should be positioned so that the right arm and back are supported. The feet should not be crossed and should rest with the bottom of both feet on the floor. For shorter individuals, a telephone book to support their feet if needed.

12. ☐ Locates the brachial pulse in the antecubital fossa by palpation. Marks the location of the artery with a pen.
13. ☐ Puts on the appropriate blood pressure cuff. Ensures that the “art” label on the blood pressure cuff is directly above the pen mark. Ensures that there is one inch between the bottom of the blood pressure cuff and the crease of the elbow.
14. ☐ Checks for proper positioning of arm. The arm should be positioned so that the antecubital fossa is at the level of the heart. Small or short participants may have to raise their body to the correct position by changing the chair position up or down. Very tall participants may need to rest their arm on a book or pillow to achieve proper positioning.
15. ☐ Explain that patient needs to relax and be quiet for a 5-minute rest period. Says the following verbatim, “Before taking your first blood pressure reading, there will be a 5 minute waiting period. When I inflate the cuff, it may feel tight and you will feel some pressure on your upper arm. While we are measuring your blood pressure, we ask you not to talk and I will not talk either because talking and moving changes your blood pressure. Do you have any questions?”
16. ☐ Leaves for 5 minutes.
17. ☐ Returns and sets up Omron blood pressure machine, making sure it is plugged in.

18. ☐ Press On/Start buttons together for 3 seconds for F1, F2, F3.

19. ☐ F1 refers to the number of inflations and should be set to '3'. If necessary, the deflation button can be used to change the number of inflations.
20. ☐ Press "start" to show the value for F2. F2 is the waiting time before starting inflations and should be set to zero. If needed, the deflation button can be used to change the setting for F2.
21. ☐ Press "start" to show the value for F3. F3 is the time interval between each blood pressure reading and should be set to 30 seconds. If needed, the 'deflation" button can be used to change the value for F3 to 30.
22. ☐ Once these three settings (F1, F2, and F3) have been correctly established, turn the Omron machine off.
23. ☐ Turn the Omron machine back on by pressing "start".
24. ☐ Waits for 2 minutes between the first and second blood pressure readings
25. ☐ Waits for 2 minutes between the second and third blood pressure readings
26. ☐ When all three readings are finished, record all three systolic/diastolic blood pressure readings and the average of last 2 systolic/diastolic blood pressure and pulse.
27. ☐ If the average systolic blood pressure reading is equal to or greater than 170 mm Hg OR if the average diastolic blood pressure reading is equal to or greater than 110 mm Hg, the coordinator stops immediately and pages Dr. Kandula.

APPENDIX 8A SEATED BLOOD PRESSURE FORM

SAHELI STUDY

Participant ID #	Acrostic	Date of Visit	Staff ID #
		Month / Day / Year	

SEATED BLOOD PRESSURE

VISIT: Baseline 3 Month 6 Month

Coordinator Instructions: Please use a black pen and fill in bubbles completely.

Dinamap number:

1. Arm circumference: .

2. Cuff size
   - ○ Small Adult (19-25cm)
   - ○ Large Adult (33.1-40cm)
   - ○ Adult (25.1-33cm)
   - ○ Thigh (40.1-50cm)

Seated Blood Pressure

3. First reading: SBP DBP Record time of day :
4. Second reading: SBP DBP
5. Third reading: SBP DBP

Please use 24 hour clock

6. Are you currently taking blood pressure medication? ○ Yes ○ No ○ Don't know

Alerts and Referrals

Use the mean of the second and third blood pressure measurements for alerts and follow the criteria below:

Blood Pressure Values	Action
1. Diastolic blood pressure greater than 120mmHg OR Systolic blood pressure greater than 210mmHg	1. *Immediate referral to a health care provider*
2. Diastolic blood pressure of 110-119mmHg OR Systolic blood pressure of 180-209mmHg	2. *Referral to a health care provider within one week*
3. Blood pressure greater than 140/90mmHg	3. *Requires follow-up within two months*

Page 1 of 1

CHAPTER 8: ACCELEROMETERS

OVERVIEW

The accelerometer (motion sensor) is used to measure the participant's level of activity. A team member will instruct the participant to wear the motion sensor during *all waking hours* (at least 10 hours/day) for the next *seven (7) days*. To increase the likelihood that participants wear the motion sensors properly, a team member will explain the purpose of the motion sensor, fit the participant with a belt and sensor and give instructions for wearing the sensor. At the baseline visit, the team member will also provide instructions for returning the accelerometer.

ACCELEROMETER SETUP

There are many types of motion sensors. In this study we will be using the GT3X+ motion sensor developed by ActiGraph. Data collected by ActiGraph motion sensors are accessible only through the ActiGraph software. The initial set up of the motion sensor has to be done before the participant comes in for each visit (baseline, 3- and 6 months).

PLACEMENT OF ACTIGRAPH

The Actigraph motion sensor comes with a waistband that will be worn around the hip. It should be worn snuggly around the hip (as close as possible to the right hip bone). (See Appendix 9A for details). It is important that the accelerometer is fastened tightly or measurements will be inaccurate. The motion sensor can be worn either over or under clothing.

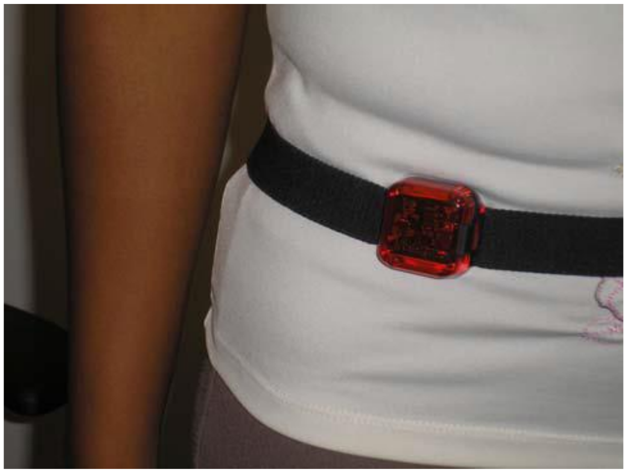

PREPPING THE PARTICIPANT

The participant will be responsible for wearing and removing the motion sensor. Show the participant how to wear the motion sensor (directions in Appendix 8A). Provide the necessary instructions and contact information to the participant. Review the instructions with the participant and ask them to wear the motion sensor for at least 7 days. Give the participant the reminder card (Appendix 8C) to place in an area where they will see it every morning and remember to wear the motion sensor. Specifically point out that they take off the sensor before going to sleep and put it on again in the morning and to take it off before a shower or swim. Schedule a visit for the participant to come in and return the motion sensor.

COMPLETE THE ACTIGRAPH DISTRIBUTION FORM

Each time you give the participant the ActiGraph, complete the ActiGraph Distribution form (Appendix 8D). Record the person's full name on the form, whether or not the participant received an ActiGraph and the serial number of the ActiGraph.

INTERIM TELEPHONE CONTACT

Participants will be called three times during the 7 days they are wearing the motion sensor (see Appendix 8B for phone call script). First call will be next day morning to make sure the participant understands how to wear the motion sensor. Second call will be on the 3rd or 4th day of the week to make sure everything is going well and answer any questions the participant may have. Third call will be one day before the participant is scheduled to return the motion sensor to remind them of their appointment with the project staff.

An effort will be made to schedule these reminder calls with the 24-hour food recall (see Chapter 10 for details on 24-hour food recall telephone calls).

COMPLETE THE ACTIGRAPH RETURN AND READING FORM

At the next appointment, 7 to 8 days after the first appointment, the participants will return the motion sensor. Please complete the Return and Reading Form (Appendix 8E) when the participant returns the sensor.

APPENDIX 8A
ACCELEROMETER SCRIPT

The following pages contain the script to lead the team member and participant through the orientation meeting. Topics covered include:

1. Instructions for when and how to wear the motion sensor
2. Fitting the sensor
3. Review of instructions and returning the sensor

To ensure consistency of instructions that are given to each participant, please follow the script – spoken words are in italics and additional instructions to the team member are in brackets.

1. Explain How and When to Wear the Sensor

"As a part of this study, we are asking you to wear a motion sensor every day for the next seven days in a row. Today I will show you how to wear the motion sensor and go over instructions for when to wear it."

"Let's go over the instructions for wearing the monitor in detail now."

"You should wear the motion sensor during ***all hours that you are awake*** *over the next* ***seven days****. Put the sensor on first thing in the morning when you wake up, and take it off at night for bed. Put the sensor somewhere* ***safe,*** *where you will see it in the morning and put it on again right away."*

"There is no on/off button or anything you need to adjust during the next seven days."

"Wear the sensor during all the hours that you are awake – at least 10 hours per day. The sensor needs to stay dry, so take it off to shower, take a bath or swim. Make sure to put the sensor back on when you are done."

"Be careful when changing clothes, going to the bathroom or other types of activities where you could drop the sensor or it could get hit hard. The sensor will not break if it is bumped, but please be careful when using the bathroom that the sensor does not fall into the toilet."

"It is very important that you go about your normal, everyday activities this week, and you do not make changes to your routines. You should do your daily activities just as you would without the sensor."

"Do you have any questions about when to wear the sensor?"

2. Fit the Sensor

"Now let's fit you with a motion sensor. The sensor should be worn around the body at about hip level on the right side using this elastic belt."

"To best position the sensor, draw an imaginary line from the center of your right knee cap up the

front of your leg to your right hipbone."

"The sensor should be worn over your right hip at this spot. Once you put the belt on, slide the sensor to this spot. The belt should be snug enough to hold the sensor in place. Make sure to ***always*** *wear the belt. Let's have you put on a belt and sensor and check the fit."*

"Do you have any questions about how to put the monitor on or where to put it on your body?"

"You can keep the sensor on now. We are going to have you start wearing the monitor for the rest of today so you can get used to it. Remember to put it somewhere safe when you go to bed tonight and put it on first thing tomorrow morning again. A staff member will call you tomorrow morning to give you a reminder."

3. Review Instructions and Set up Appointment to Return Sensor

"Do you remember the instructions we went over earlier for wearing the monitor?"

"Make sure to go about your activities during the day just like you usually would in any other week, okay?"

"If you have any questions during the next week, you can call this telephone number or ask the staff member when they call you." [Telephone number is on the reminder card – Appendix 8C]

"A member of the staff will be calling you three times over the next week to check-in and make sure wearing the motion sensor is going okay for you. The three calls will be:

1. *Right away tomorrow morning – to make sure you are all set for the week.*
2. *On the 3rd or 4th day for mid-week check-in to answer any questions you have.*
3. *On the last day – to see how the week went and remind you of your appointment."*

"Is it okay for us to call you during the week?"

"We need to get this sensor back from you – this is a required step for participation in the study and to receive all your incentive money. Even though the sensor looks like a toy, it is an expensive research tool. It cannot be used by itself, and it has no monetary value if it is lost, stolen or sold."

"When you are done with your seven days, bring the motion sensor back to MAFS. We can give you your incentive money when we see you."

"When the staff member contacts you during the week, they will remind you when your return appointment is, and you can ask them any questions."

"Do you have any questions about anything we went over today?"

"You are all set with your sensor and instructions. Wear your sensor for the rest of today, and ***then put it where you will see it and put it on first thing tomorrow morning.*** *Thank you."*

APPENDIX 8B
ACCELEROMETER REMINDER CALL PHONE SCRIPT

(If 24-hour food recall is done during the same call, complete the accelerometer reminder ***after*** the food recall has been completed).

"Hello, this is [full name], from the South Asian Heart Disease Prevention study. May I speak to [participant's name]? I'm calling to check if everything is going well with wearing the motion sensor. Are you wearing the sensor? Is everything okay with wearing sensor every day?"

If yes,	***"That's great. Do you have any questions about wearing the sensor? Do you have any other questions?"*** (Address any questions the participant has).<br><br>(Terminate the call)*
If no,	***"Are you having problems wearing the sensor? Do you remember how to put it on correctly?"*** (If the participant is having problems remembering the correct way to wear the sensor, review instructions using the script in Appendix 9A).<br><br>***"Do you think you are clear on how to wear the sensor now? Do you have any other questions?"*** (Address any questions the participant has).<br><br>(Terminate the call)*
If the participant is having a hard time remembering to wear the sensor	***"What do you think are some things you can do to remember to put on the sensor first thing in the morning?"*** (See if participant can come up with some strategies. If not, give some examples of things that may help – leaving it on your dressing table, putting it next to your phone or keys, effectively using the reminder card etc. If the participant thinks some of these ideas may help, encourage them to try it for the remaining days).<br><br>***"Try to use these reminder ideas and see if they help you. If you continue to have problems please give us a call using the number on the reminder card. Do you have any questions?*** (Address any questions the participant has).<br><br>(Terminate the call)*
If the participant says they do not like wearing the sensor or they do not want to wear it	***"In order to participate in this study, it is very important that you wear the sensor for at least 10 hour each day for the next 7 days. If you do not wear the sensor, we may not be able to enroll you in this study. Is there any way we can help so you wear the sensor?"*** (Work with the participant to come with solutions to any issues/barriers they have with wearing the accelerometer).<br><br>***"Do you think you feel more comfortable wearing the sensor for the remaining days?***

<table>
<tr><td></td><td><table><tr><td>If yes,</td><td>“Great. (Terminate the call)*</td></tr><tr><td>If no,</td><td>“Okay, I will check with Dr. Kandula and see what we can do. We will give you a call later. Is it okay to call you later about this? (Terminate the call).

(Document the problem and discuss with the study team).</td></tr></table></td></tr>
<tr><td>*Terminate the call</td><td>“Thank you. Please remember to wear the sensor every day, for at least 10 hours. I will call you again in a few days. If you need to call us about anything, please use the number on the reminder card.”</td></tr>
</table>

APPENDIX 8C
REMINDER CARD

REMEMBER…

- ☑ Wear the monitor around your waist in the morning
- ☑ Do *not* wear the monitor while showering
- ☑ Keep the motion sensor on all day
- ☑ Wear the monitor on weekend *and* your day off work
- ☑ Remove the monitor when you go to bed at night

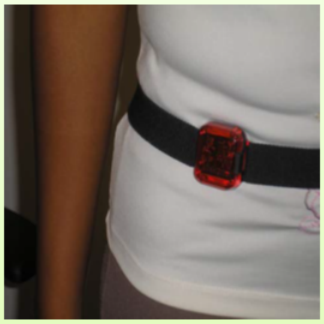

PLEASE DO *NOT* GET THE SENSOR WET!

If you have any questions, please call
Yasin Patel at 312-503-3387

याद रक्ये…

- ☑ मोनिटर को सुबहमे अपनी कमर पे पहने
- ☑ नहाने के वक़्त मोनिटर को ना पहने
- ☑ पूरा दिन मोनिटर को पहने रखे
- ☑ मोनिटर को सनिवार और रविवार और छुट्टी के दिन बे पहने
- ☑ शोने शे पहले मोनिटर को निकाल दे

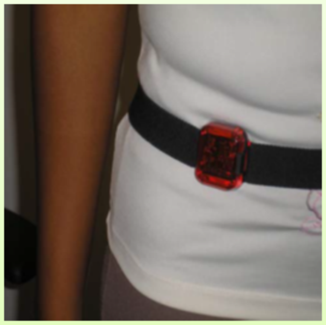

कृप्या करके मोनिटर को गिला ना होने दे

कोईभी प्रश्न के लिए फोन कीजिये
Yasin Patel (312) 503-3387

APPENDIX 8D
ACTIGRAPH DISTRIBUTION FORM

Participant ID #	Acrostic	Date of Interview	Staff ID #
__|__|__|__|	__|__|__|__|	__|__|/__|__|/__|__|__|__|	__|__|__|

SAHELI ACTIGRAPH DISTRIBUTION

Visit: Baseline 3 month 6 month

1. Did the participant receive an ActiGraph?

O Yes → Please record last four digits of serial number:

O No → Why didn't participant receive an Actigraph?
(Note: Mark all that apply.)

- O Participant refused
- O Cognitive impairment
- O Physical/medical problems

(Please specify: ________________________)

APPENDIX 8E
ACTIGRAPH RETURN AND READING FORM

Participant ID #	Acrostic	Date of Interview	Staff ID #
__|__|__|__|	__|__|__|__|	__|__|/__|__|/__|__|__|__|	__|__|__|

SAHELI ACTIGRAPH RETURN AND READING

Visit: Baseline 3 month 6 month

1. Date participant returned ActiGraph:

   ☐☐/☐☐/☐☐☐☐
   Mo Day Year

2. How many days was the ActiGraph worn?
   (Examiner Note: After retrieving the data from the Actigraph, review the ActiGraph Event Editor information on the computer screen and record "Days Recorded.")

   ☐☐ Days

3. How many of the days that the Actigraph was worn were valid?
   (Examiner Note: After retrieving the data from the Actigraph, review the ActiGraph Event Editor information on the computer screen and record "Valid Days.")

   ☐☐ Days

4. Was any activity collected and recorded on the ActiGraph?
   O Yes O No ↓

Why wasn't any activity collected and recorded on the ActiGraph? **(Please mark <u>all</u> that apply.)**

- O Cognitive problems
- O Dexterity problems
- O Vision problems
- O Travel plans interfered
- O Participant took off; did not replace or restart correctly
- O Equipment failure; periods not recorded
- O ActiGraph lost
- O Participant refused
- O Other **(Please specify: ______________________________________)**

CHAPTER 9: 24 Hour DIETARY FOOD RECALL

OVERVIEW

The purpose of a 24-hour dietary recall is to gain information regarding the participant's diet. Study staff will be trained to conduct 24-hour food recalls in order to examine the initial efficacy of the South Asian Heart Lifestyle Intervention (SAHELI) to change saturated fat intake and fruit and vegetable consumption among South Asians. For the 24-hour dietary recall, the participant is asked to remember and report all the foods and beverages consumed in the preceding 24 hours (from midnight to midnight). The recalls will be conducted by interview, both in-person and by telephone, using a semi-scripted form. The information collected during the 24-hour recall interview will be entered into the Nutrition Data System for Research (NDSR) 2011, a computer based software application created by the University of Minnesota Nutrition Coordinating Center (NCC), for nutritional analysis and assessment.

24-HOUR DIETARY RECALL COLLECTION METHOD

Schedule

Three 24-hour dietary recall interviews (2 weekdays and 1 weekend day) will be collected from each study participant in both the control and intervention group at baseline, 3-month and 6-month. The first 24-hour food recall will be conducted in-person, at the baseline visit. Staff will then contact study participants within one week of the visit to conduct two 24-hour food recalls over the telephone. The 24-hour food recalls conducted on a weekend day will be made over the telephone. The dates and times of these recalls will be unannounced (unscheduled) so that participants do not change their normal eating pattern. However, during the visits, the participants will be asked to indicate the preferred days of the week and times of day to be called and the telephone number(s) at which they may be reached. Email addresses will also be collected and used when participants are difficult to reach by phone.

	**Baseline**	**3-month**	**6-month**
**Day of visit**	One in-person 24-hour dietary recall		
**Within one week of visit**	Two over-the-phone 24-hour dietary recalls (one on weekend day)		

A calendar should be available for each dietary interviewer and participant. This can be used at the beginning of the in-person dietary interview to assist the participant in recalling yesterday's dietary intake and in looking at availability for the telephone dietary recalls. The participant's availability for the telephone dietary recalls will be recorded on a calendar and be given to the participant.

Multiple-Pass Approach Description

The multiple-pass method enhances and improves the 24-hour dietary recall by providing respondents with multiple cues and opportunities to help them remember and describe foods they consumed. It minimizes underreporting of dietary intake and facilitates the collection of recalls in a standardized fashion. For this study, we will be using the multiple-pass approach for data collection as described in the NDSR 2011 manual which uses four distinct passes. A total of four passes which means the first pass involves obtaining from the participant a listing of all foods and beverages consumed in the previous 24 hours. This listing is reviewed with the participant for completeness and correctness in the second pass. During the third pass, the interviewer then collects detailed information about each reported food and beverage, including the amount consumed and method of preparation. Finally, in the fourth pass, the detailed information is reviewed for completeness and correctness.

Dietary Recall Form Description

The Dietary Recall Form includes a semi-scripted multiple-pass approach (as described above) for the 24-hr dietary recall and food outline tables to record the date, location, time, and the dietary information provided by the participant, specifically the food, detailed description of food, quantity and unit of food. The dietary interviewer should be familiar with this form prior to conducting a food recall. Since the form is simply a guide to be used during the interview, the dietary interviewer should have read and know the procedures as outlined in this chapter. See Appendix 10A for the Dietary Recall Form.

<u>Dietary Recall Form:</u>

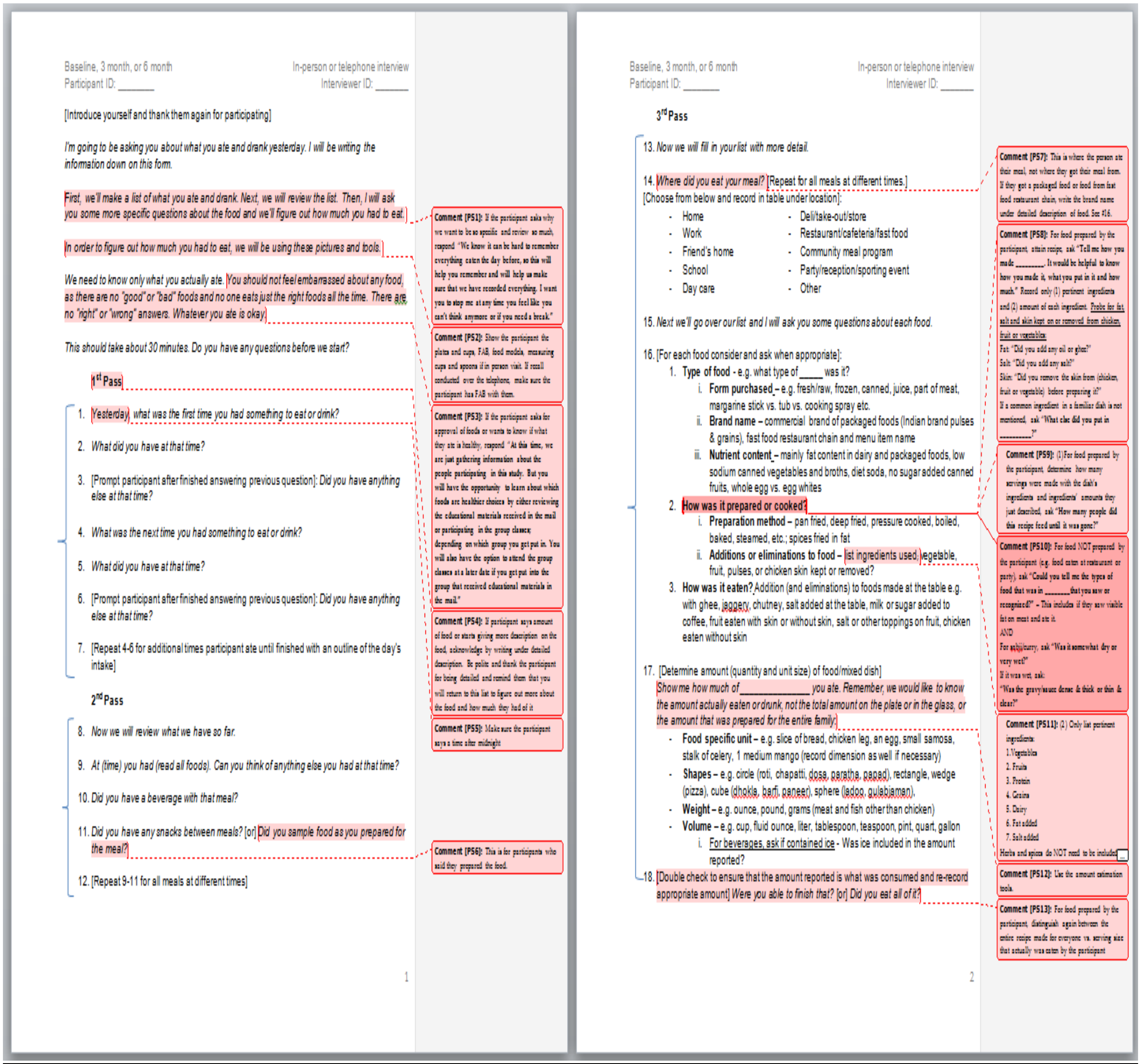

Baseline, 3 month, or 6 month — In-person or telephone interview
Participant ID: _______ — Interviewer ID: _______

[Introduce yourself and thank them again for participating]

I'm going to be asking you about what you ate and drank yesterday. I will be writing the information down on this form.

First, we'll make a list of what you ate and drank. Next, we will review the list. Then, I will ask you some more specific questions about the food and we'll figure out how much you had to eat.

In order to figure out how much you had to eat, we will be using these pictures and tools.

We need to know only what you actually ate. You should not feel embarrassed about any food, as there are no "good" or "bad" foods and no one eats just the right foods all the time. There are no "right" or "wrong" answers. Whatever you ate is okay.

This should take about 30 minutes. Do you have any questions before we start?

1st Pass

1. *Yesterday, what was the first time you had something to eat or drink?*
2. *What did you have at that time?*
3. [Prompt participant after finished answering previous question]: *Did you have anything else at that time?*
4. *What was the next time you had something to eat or drink?*
5. *What did you have at that time?*
6. [Prompt participant after finished answering previous question]: *Did you have anything else at that time?*
7. [Repeat 4-6 for additional times participant ate until finished with an outline of the day's intake]

2nd Pass

8. *Now we will review what we have so far.*
9. *At (time) you had (read all foods). Can you think of anything else you had at that time?*
10. *Did you have a beverage with that meal?*
11. *Did you have any snacks between meals?* [or] *Did you sample food as you prepared for the meal?*
12. [Repeat 9-11 for all meals at different times]

1

Comment [PS1]: If the participant asks why we want to be so specific and review so much, respond "We know it can be hard to remember everything eaten the day before, so this will help you remember and will help us make sure that we have recorded everything. I want you to stop me at any time you feel like you can't think anymore or if you need a break."

Comment [PS2]: Show the participant the plates and cups, FAB, food models, measuring cups and spoons if in person visit. If recall conducted over the telephone, make sure the participant has FAB with them.

Comment [PS3]: If the participant asks for approval of foods or wants to know if what they ate is healthy, respond "At this time, we are just gathering information about the people participating in this study. But you will have the opportunity to learn about which foods are healthier choices by either reviewing the educational materials received in the mail or participating in the group classes; depending on which group you get put in. You will also have the option to attend the group classes at a later date if you get put into the group that received educational materials in the mail."

Comment [PS4]: If participant says amount of food or starts giving more description on the food, acknowledge by writing under detailed description. Be polite and thank the participant for being detailed and remind them that you will return to this list to figure out more about the food and how much they had of it

Comment [PS5]: Make sure the participant says a time after midnight

Comment [PS6]: This is for participants who said they prepared the food.

Baseline, 3 month, or 6 month — In-person or telephone interview
Participant ID: _______ — Interviewer ID: _______

3rd Pass

13. *Now we will fill in your list with more detail.*
14. *Where did you eat your meal?* [Repeat for all meals at different times.]
[Choose from below and record in table under location]:
    - Home
    - Work
    - Friend's home
    - School
    - Day care
    - Deli/take-out/store
    - Restaurant/cafeteria/fast food
    - Community meal program
    - Party/reception/sporting event
    - Other
15. *Next we'll go over our list and I will ask you some questions about each food.*
16. [For each food consider and ask when appropriate]:
    1. **Type of food** - e.g. what type of _____ was it?
        i. **Form purchased** – e.g. fresh/raw, frozen, canned, juice, part of meat, margarine stick vs. tub vs. cooking spray etc.
        ii. **Brand name** – commercial brand of packaged foods (Indian brand pulses & grains), fast food restaurant chain and menu item name
        iii. **Nutrient content** – mainly fat content in dairy and packaged foods, low sodium canned vegetables and broths, diet soda, no sugar added canned fruits, whole egg vs. egg whites
    2. **How was it prepared or cooked?**
        i. **Preparation method** – pan fried, deep fried, pressure cooked, boiled, baked, steamed, etc.; spices fried in fat
        ii. **Additions or eliminations to food** – list ingredients used; vegetable, fruit, pulses, or chicken skin kept or removed?
    3. **How was it eaten?** Addition (and eliminations) to foods made at the table e.g. with ghee, jaggery, chutney, salt added at the table, milk or sugar added to coffee, fruit eaten with skin or without skin, salt or other toppings on fruit, chicken eaten without skin
17. [Determine amount (quantity and unit size) of food/mixed dish]
Show me how much of _____________ you ate. Remember, we would like to know the amount actually eaten or drunk, not the total amount on the plate or in the glass, or the amount that was prepared for the entire family.
    - **Food specific unit** – e.g. slice of bread, chicken leg, an egg, small samosa, stalk of celery, 1 medium mango (record dimension as well if necessary)
    - **Shapes** – e.g. circle (roti, chapatti, dosa, paratha, papad), rectangle, wedge (pizza), cube (dhokla, barfi, paneer), sphere (ladoo, gulabjaman).
    - **Weight** – e.g. ounce, pound, grams (meat and fish other than chicken)
    - **Volume** – e.g. cup, fluid ounce, liter, tablespoon, teaspoon, pint, quart, gallon
        i. <u>For beverages, ask if contained ice</u> - Was ice included in the amount reported?
18. [Double check to ensure that the amount reported is what was consumed and re-record appropriate amount] *Were you able to finish that?* [or] *Did you eat all of it?*

2

Comment [PS7]: This is where the person ate their meal, not where they got their meal from. If they got a packaged food or food from fast food restaurant chain, write the brand name under detailed description of food. See #16.

Comment [PS8]: For food prepared by the participant, attain recipe, ask "Tell me how you made ________. It would be helpful to know how you made it, what you put in it and how much." Record only (1) pertinent ingredients and (2) amount of each ingredient. <u>Probe for fat, salt and skin kept on or removed from chicken, fruit or vegetables:</u>
Fat: "Did you add any oil or ghee?"
Salt: "Did you add any salt?"
Skin: "Did you remove the skin from (chicken, fruit or vegetable) before preparing it?"
If a common ingredient in a familiar dish is not mentioned, ask "What else did you put in ________?"

Comment [PS9]: (1)For food prepared by the participant, determine how many servings were made with the dish's ingredients and ingredients' amounts they just described, ask "How many people did this recipe feed until it was gone?"

Comment [PS10]: For food NOT prepared by the participant (e.g. food eaten at restaurant or party), ask "Could you tell me the types of food that was in _______ that you saw or recognized?" – This includes if they saw visible fat on meat and ate it.
AND
For sabji/curry, ask "Was it somewhat dry or very wet?"
If it was wet, ask:
"Was the gravy/sauce dense & thick or thin & clear?"

Comment [PS11]: (2) Only list pertinent ingredients:
1.Vegetables
2. Fruits
3. Protein
4. Grains
5. Dairy
6. Fat added
7. Salt added
Herbs and spices do NOT need to be included ...

Comment [PS12]: Use the amount estimation tools.

Comment [PS13]: For food prepared by the participant, distinguish again between the entire recipe made for everyone vs. serving size that actually was eaten by the participant

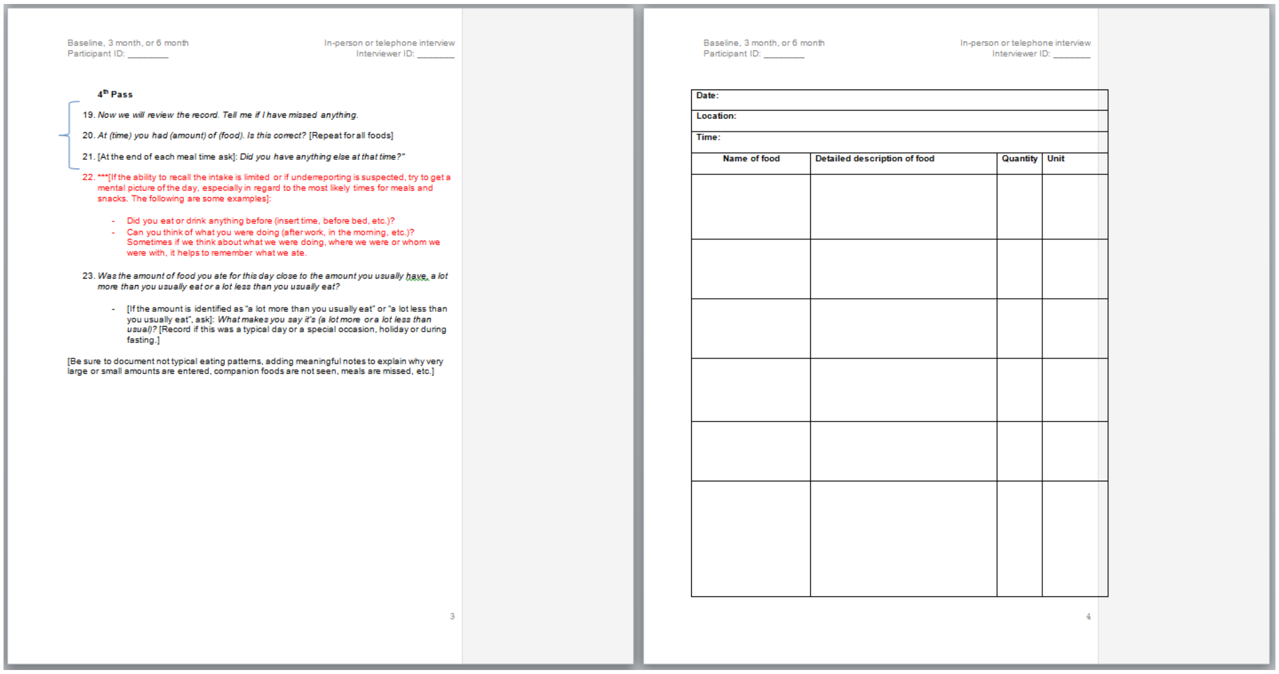

Baseline, 3 month, or 6 month
Participant ID: ________

In-person or telephone interview
Interviewer ID: ________

4th Pass

19. *Now we will review the record. Tell me if I have missed anything.*
20. *At (time) you had (amount) of (food). Is this correct?* [Repeat for all foods]
21. [At the end of each meal time ask]: *Did you have anything else at that time?"*
22. ***[If the ability to recall the intake is limited or if underreporting is suspected, try to get a mental picture of the day, especially in regard to the most likely times for meals and snacks. The following are some examples]:
    - Did you eat or drink anything before (insert time, before bed, etc.)?
    - Can you think of what you were doing (after work, in the morning, etc.)? Sometimes if we think about what we were doing, where we were or whom we were with, it helps to remember what we ate.
23. *Was the amount of food you ate for this day close to the amount you usually have, a lot more than you usually eat or a lot less than you usually eat?*
    - [If the amount is identified as "a lot more than you usually eat" or "a lot less than you usually eat", ask]: *What makes you say it's (a lot more or a lot less than usual)?* [Record if this was a typical day or a special occasion, holiday or during fasting.]

[Be sure to document not typical eating patterns, adding meaningful notes to explain why very large or small amounts are entered, companion foods are not seen, meals are missed, etc.]

3

Baseline, 3 month, or 6 month
Participant ID: ________

In-person or telephone interview
Interviewer ID: ________

**Date:**			
**Location:**			
**Time:**			
**Name of food**	**Detailed description of food**	**Quantity**	**Unit**

4

Amount Estimation Tools for In-person Interviews

Accurate quantification of foods and beverages is essential for obtaining useful dietary data. Some participants are more adept than others in accurately estimating amounts of foods and beverages but all should have access to the same tools to aid in this process. For in-person interviews, each interview station should have a complete set of amount estimation tools. This set will include an assortment of household cups, glasses, spoons, bowls, a ruler, replicas of select foods (Nasco models), and a copy of the Food Amounts Booklet (FAB) available from NCC. To promote participant understanding of how to use the FAB, the dietary interviewers will briefly describe the pages before proceeding to collect the 24-hour recall. Also, the participant will be given a copy of the FAB to take home for use during the telephone recalls. The interviewer copy includes specific amount measure labels on each picture as well as a one page conversion guide. To retain scale, the FAB must be printed on 8.5x11 inch paper, 1 page per sheet of paper. Also, make sure the page scaling is set to 'none' and the 'Auto-Rotate and Center' button is on. To make sure the booklet was printed to scale, measure the circles on page 2 of the booklet to confirm they match the measurements specified on that page of the interviewer copy of the booklet. See Appendix 10B for the Food Amounts Booklet.

Amount Estimation Tools for Telephone Interviews

The Food Amount Booklet is given to participants at the in-person interview to be used during the telephone interviews. As mentioned earlier, during the in-person interview, orientation to the FAB will occur in conjunction with other amount estimation tools to help the participant understand how to recall and estimate the quantities of foods consumed.

<u>Household measuring cups and spoons:</u>

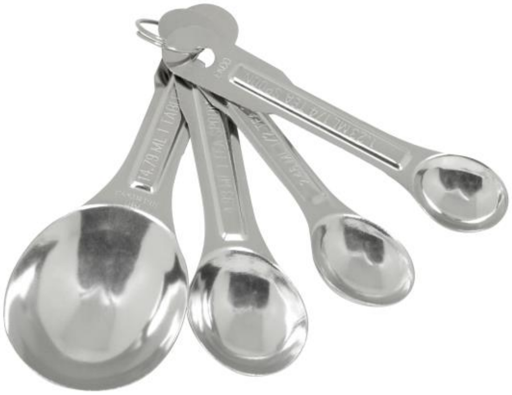

South Asian Food Kit

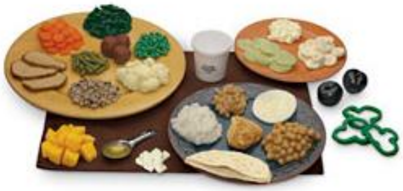

Banana, Whole

Developed by Nasco

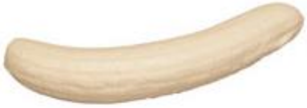

Nasco Food models:

Okra

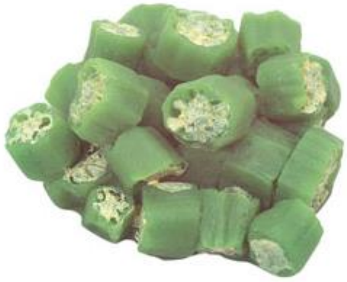

Indian Fry Bread

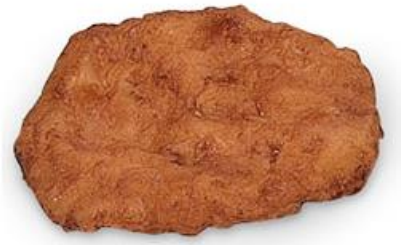

CONFIDENTIAL- Do not distribute, developed by Northwestern University

Food Amount Booklet Photos (not inscale):

Chicken

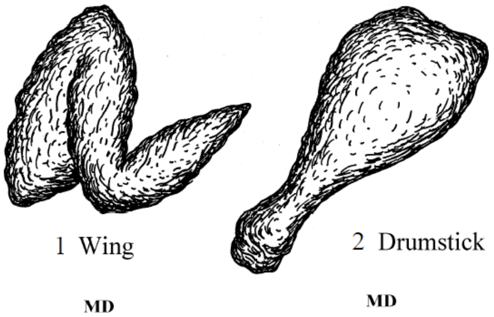

Fish

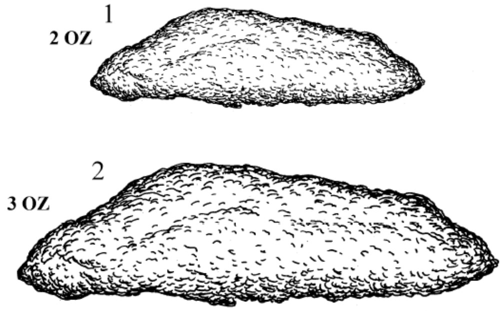

Mug

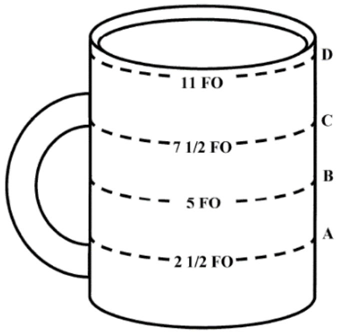

Bowls

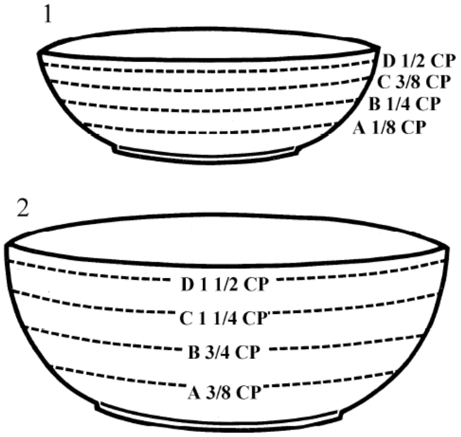

Squares and Rectangles

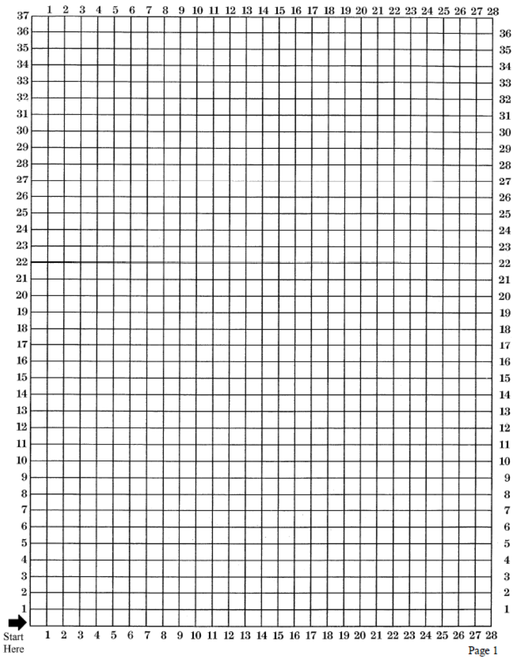

Circles

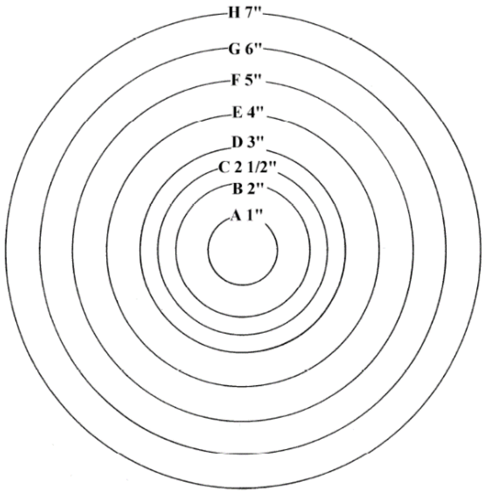

GENERAL GUIDELINES FOR CONDUCTING 24-HOUR DIETARY RECALLS

Because each participant is the primary respondent for the dietary recall, it is important for the dietary interviewer to be able to motivate him/her to provide complete and accurate information. He/she must always remain neutral and not let anything in words or manner express criticism, surprise, approval, or disapproval related to the participant's responses during the recall interview. Dietary interviewers should be sensitive by adapting a style and approach to make the participant comfortable. Every effort should be made to keep the dietary data collection as objective and non-judgmental as possible. He/she should avoid congratulating participants for eating certain foods or reacting with dismay to reports of other foods. The dietary interviewer should stress that he/she wants to know what the participant really ate and that honesty is appreciated. He/she should look for both verbal and non-verbal responses, be a good listener, and thank the participant for the information provided.

Above all, the dietary interviewer should let the participant think and say whatever comes to mind about the previous day's intake, avoiding interruptions that may be distracting to the participant. The dietary interviewer will begin by asking open ended questions as seen in the scripted form, for example "What is the first thing you have to eat or drink?" Open ended or neutral questions require a narrative or explanatory response and allow the client to talk about information that he/she feels is important. Open ended questions start with what, how or when or an open statement. Asking open or neutral questions may result in storytelling and could take the interview off track. The dietary interviewer should refocus the interview by acknowledging what the participant has said but reminding them what was being discussed before and referring back to the original question.

The dietary interviewer should save closed ended questions for later while reviewing foods. Closed ended questions require only a yes or no answer, provide no additional information, and can be used to ask for specific information. These questions start with 'do you' or 'have you'. For example, "Do you add salt to your food at the table?" Dietary interviewers should avoid leading questions where he/she makes suggestions about the desired or expected answer such as "You don't drink whole milk, do you?" Also, dietary interviewers should try to avoid labeling meals such as breakfast, lunch, or dinner.

As the dietary interviewer conducts the 24-hour dietary recall, he/she provides positive reinforcement by stating, for example, “you are doing a good job” as appropriate. The dietary interviewer should maintain a pleasant tone of voice and avoid responding to the participant in any negative ways. If it is necessary to ask the participant to repeat what he/she said, the dietary interviewer should ask him/her to do so in a gentle way and take ownership by saying: “Sometimes it’s hard for me to hear things. Could you please tell me that again?”

PREPARING FOR THE 24-HOUR DIETARY INTERVIEW

Before the participant arrives for the in-person dietary interview, the dietary interviewer should prepare the dietary recall form and fill in the participant ID and interviewer ID, circle in-person interview, choose whether it is baseline, 3 month, or 6 month, and include yesterday’s date on the food outline table. The dietary interviewer should check the interview station to ensure that the amount estimation tools are appropriately organized, that all necessary forms are in place, and that a current calendar is available for use in the in-person dietary recall and in planning for the telephone recall.

When conducting a telephone dietary recall, each dietary interviewer should have his/her own headset. Before dialing the participant’s phone, the dietary interviewer will prepare the dietary recall form and fill in participant ID and interviewer ID, circle telephone interview, choose whether it is baseline, 3 month, or 6 month, and include yesterday’s date on the food outline table. The dietary interviewer should check the interview station to ensure that the interviewer’s copy of the Food Amount Booklet is available and that all necessary forms are in place. If the participant is reached, the dietary interviewer introduces him/herself, explains why he/she is calling, and asks the participant if it is a good time for them to complete the interview, which should take about 30 minutes of their time.

THE 24-HOUR DIETARY RECALL INTERVIEW

Introducing Participant to the Dietary Recall Interview

When the participant arrives, the dietary interviewer welcomes the participants to the dietary recall portion of the visit. He/she introduces himself/herself in a friendly but professional manner and gives basic information about the dietary interview to the participant and responds to any questions the participant may have. The dietary interviewer will explain that he/she will be asking the participant what they ate and drank yesterday and will be recording this information on the form. The dietary interviewer should always give neutral responses to whatever the participant tells them and should help the participant feel comfortable to encourage honesty. It can be hard to remember everything eaten in the last 24 hours so allow for questions and comments.

When conducting a telephone interview ask the participant if they have the Food Amounts Booklet (FAB) with them or can take a minute to go find it for use during the interview. If the participant can’t find the booklet, write down the participant’s mailing address and let him/her know that a new booklet will be mailed to them. Thank them for their time and let them know you’ll call them back after the booklet has been mailed. If the participant has been difficult to reach by phone (e.g. repeated attempts have been made to reach the participant for the telephone recalls), proceed with the interview without the booklet. Please note that interview was conducted without FAB.

The First Pass

The first pass is used to collect an outline of the previous day’s intake. It is designed to get participants to begin thinking about what and when they ate. The dietary interviewer asks when was the first time the participant had something to eat or drink and what they had at that time. The interviewer prompts the participant by asking them if they had anything else at that time. The interviewer then asks the same questions for the next time(s) they had something to eat or drink.

The Second Pass
In the second pass, the interviewer reviews the list by reading each meal time and foods they had at each meal time. The dietary interviewer prompts the participant by asking them again if they had anything else at those times, if they had a beverage with any meals, if they had any snacks between meals or if they sampled food as they prepared for the meal.

The Third Pass
During the third pass, the interviewer informs the participant that now they will look at the foods on the list with more detail. The interviewer starts by asking where they had each meal for each different meal time. Based on the participant's response, the interviewer can select from the following locations: home, work, friend's home, school, day care, restaurant/cafeteria/fast food, deli/take-out/store, community meal program, party/reception/sporting event, or other. The meal location helps to determine which food variables to enter when entering the 24-hour dietary recall information into NDSR. For example, many foods in NDSR differentiate between home prepared and restaurant prepared foods. Collecting information on the source of the meal aids in the food description process.

Food Description
For each food listed, the interviewer will determine which questions to ask. For individual food items such as milk, juice, cheese, meat snack foods like potato chips, the interviewer will ask what type of food it was to elicit responses such as name of specific food like chocolate milk or 100% orange juice and to see if foods had varying nutrient content for example, skim vs. 2% milk, 85% lean ground turkey, low fat or fat free yogurt, whole egg or egg whites, low sodium canned goods, sugar free Jello, etc. It is also important to ask the form purchased (e.g. fresh, frozen, canned, dry, etc.) especially for foods like fruits, vegetables and beans and to ask for brand name if any. Make sure to ask if anything was added to the food during prep or at the table. These are things like solid fats, oils, dressings, cream, sugar, or condiments. For a mixed dish made or prepared by the participant, ask the participant for recipe details such as what ingredients were used, amounts of ingredients used and method of preparation (e.g. boiled, baked, fried, breaded, etc.) Also, determine the difference between the servings made and the amount eaten by the participant. The amount eaten by the participant will be verified in the next step when determining quantity and unit size, however, if the participant is able to give recipe details, it is important to note how many serving sizes that the recipe made so the information can be appropriately entered in NDSR later. If a mixed dish such as a sandwich or taco is reported to be from a national fast food restaurant chain, record the name of the restaurant. It is not necessary to ask the participant for each ingredient in the food, though. Any modifications made to national fast food restaurant meals, such as not eating the bun for a McDonald's hamburger or ordering a Subway sandwich with only vegetables, should be recorded.

Food Amount
For each food or mixed dish, record the amount, both quantity and unit size. For example, 1 large egg ('1' is the quantity and 'large' is the unit size). Unit size includes food specific units, geometric food shapes, weight, and volume.

If it is the first interview, the dietary interviewer should introduce the study amount estimation tools. The dietary interviewer should explain to the participant that the FAB includes examples of eating and serving spoons, glasses, mugs and bowls which can be different sizes compared to measuring spoons and cups. Showing the Nasco food models helps the participant visualize the amount of a specific food. If a participant is describing the amount of food, it is also helpful for him/her to see the 'Mounds' pictures on pages 14 and 15 of the FAB. Nasco meat food models will be available but for further assistance in determining size and portion of meat, the dietary interviewer can refer to the 'Meats' pictures on page 16 through 18 of the FAB. Emphasize that these tools are not always needed for estimating amounts and give

examples of amounts that can be expressed using food-specific unit such as a “large” apple or a “thin slice” of tomato. The dietary interviewer should remind the participant that he/she needs to know the amount actually eaten or drunk, not the total amount on the plate or in the glass, or the amount that was prepared for the entire family. When an amount is given by the participant, the dietary interviewer asks if he/she ate all or only a part of that amount.

Interviewers should not specify to the participant a particular method to use to estimate the amount of food or beverage. First, he/she asks a completely open-ended question such as “how much juice did you drink?” This type of question encourages the participant to express the amount in his/her own words. If the participant appears to be having difficulty answering the question, the dietary interviewer may then suggest that he/she try to visualize the juice container and compare the amount with one of the amount estimation tools. If the participant continues having difficulty expressing the amount, the dietary interviewer might mention one or more of the options listed as examples depending on the food.

Food specific units, e.g., slices, each, small, single serving bag, piece, are available for many foods in NDSR and can be recorded as such. Food specific units are most frequently available for packaged items such as one piece of hard candy or an ice cream bar. In general, it is better to use the “small”, “medium”, or “large” food specific unit to describe pieces of chicken and fruit. These foods tend to be overestimated when dimensions are used because the visual image includes refuse (e.g. bone, core, peel). Foods that may seem “standard” can come in several sizes (e.g. nugget, regular, small, extra-large). Therefore, it is preferable to have the participant describe the portion consumed using the amount estimation tools (i.e. measuring cups and spoons, Nasco food models, the FAB). For example, if the participant reports having a small doughnut, record that the participant reported small and use the Food Amounts Booklet to get a rough idea of the size and record the dimensions. The person entering the 24 hour dietary recall data will then determine if that dimension is associated with the food specific unit. See photo below for an example of the food specific units of a doughnut and how they are further described using dimensions.

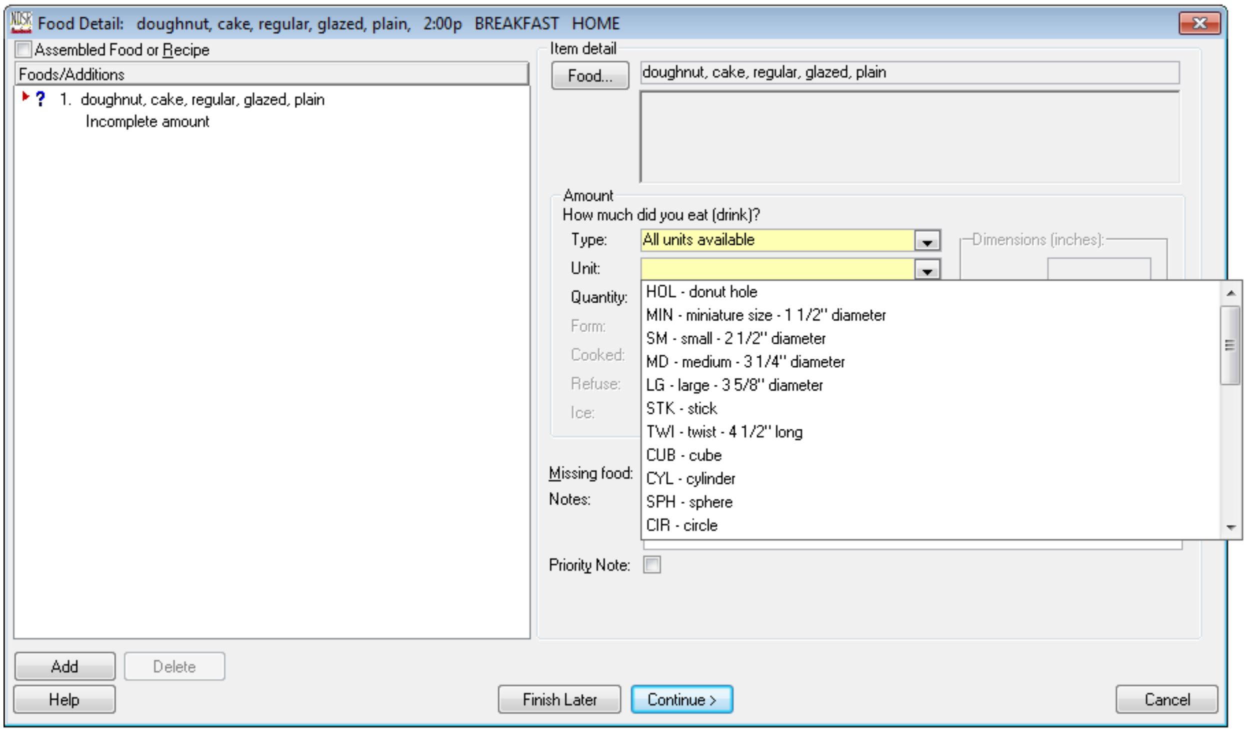

Shapes (e.g. circle, rectangle, wedge, cube) allow the participant to use two or three dimensions to describe the amount of food eaten. Food shapes in NDSR are measured using inches to describe length, width, height or diameter depending on the food. In the event that a participant reports the food shape in centimeters, the dietary interviewer refers him/her to the ruler that has inches and centimeters. Notes should be taken for any unusual dimensions, making clear that the dietary interviewer has visualized the food and is aware of any unusual dimensions and has probed sufficiently to be sure the participant has given the best description for the amount consumed. Shapes are not permitted for describing liquid and in many situations; the food specific unit is a better way to quantify the food item because few foods have true geometric shapes.

Weight measurements (e.g., ounce, pound, grams) should only be used if the exact weight is available from a package label or if the participant reports an amount using an amount estimation tool that represents weight for that specific food. Pictures or meat replicas can be used to assist participants in describing their portion sizes of cooked meat including roast, steak, pork chop, and ham. (Note: chicken parts/pieces in the Food Amounts Booklet do not have weights associated with them and are considered food-specific units.) The meat replica pictures in the Food Amounts Booklet should not be used to represent cold cuts, sausage, meat loaf, or fish because the same volume for these items (meat replicas) will have different weight. NDSR will require if the meat portions include either bones or fat, and if the fat was eaten. Consequently, the dietary interviewer should clarify with the participant if the amount of meat or fish envisioned by looking at the picture or the replica includes bone or other refuse.

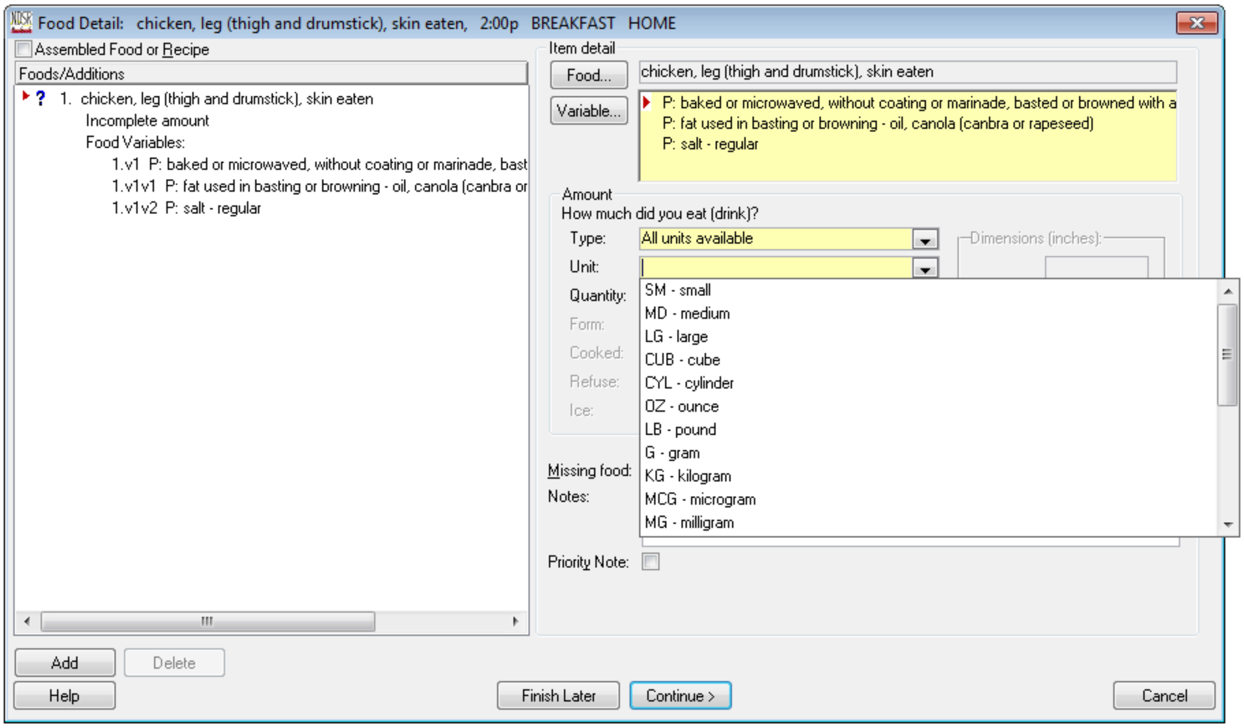

Volume measures (e.g., cup, fluid ounce, tablespoon, teaspoon, pint) are used to describe amounts for all liquid items, beverages, and non-liquid foods served or quantified in bowls, cups, or glasses. Examples in the Food Amounts Booklet include pictures of measuring cups and spoons, bowls, and glasses. If volume measurements are being used to describe non-liquid foods, include the quantity and form in which the food was eaten (e.g., sliced, diced, solid). The form determines the amount that can be placed in a particular container and factors in the density of the food item. For most beverages, NDSR will ask if the amount included ice, consequently the dietary interviewer should clarify if the amount the participant

reports includes ice. When using measuring cups or the bowls or beverage containers with the markings, dietary interviewers do not assume that the container was full. Always ask "To what line?" before entering the amount information. If the participant reports eating more than one of an item that could be different sizes, the dietary interviewer needs to remember to ask if they were the same size.

The amount the participant actually consumed is what should be entered, not what was served or cooked for the entire family. Double check to ensure that the amount reported is what was consumed. Asking "Were you able to finish that?" or "Did you eat all of it?" help to identify the unconsumed portions. The dietary interviewer must be able to visualize the amount reported and confirm as needed any questionable amounts using the amount estimation tools or by making reference to other familiar items or recognizable standards. For example, 1/16 of a hamburger should have a note saying, "ate only one bite" or 8 cups of popcorn should have a note saying, "ate entire box."

Things to Consider When Attaining Food Description and Amount

If something sounds unusual, the dietary interviewer should question it and not blindly accept the initial response. Redirecting questions and presenting appropriate alternatives from the amount estimation tools permit the participant to restate his/her initial response and allow the interviewer to verify, confirm, or correct an unusual portion. Confirmation of any unusual intake or portions should be documented by stating which amount estimation tool or picture in the Food Amounts Booklet was used.

As a general rule, the dietary interviewer should accept the participant's level of detail or opinion about the foods and beverages eaten. Participants would not be expected to be able to provide the answers to specific questions, especially regarding preparation methods or other details as part of a restaurant meal. When it becomes clear that the participant is unable to provide the level of detail asked of them, please write "unknown". NDSR unknown will then be selected when entering dietary recall information. For example, if the participant had a meat empanada from a restaurant, the percent of fat will be unknown. It is essential to probe participants to determine the accurate nutrient content of foods however, asking too many questions that cannot possibly be answered may lead the participant to respond inappropriately just to provide an answer to the question.

The Fourth Pass

The fourth pass involves a final review of the record. The dietary interviewer should go through each food with the reported amount at each meal time and ask if it is correct. At the end of each meal time, the dietary interviewer should ask if the participant had anything else at that time. The dietary interviewer should try to get a mental picture of the day, looking especially for time gaps of more than four hours between eating. Notes should be made to indicate skipped meals or to explain large time gaps. Notes should also be used to record the absence of foods, beverages or typical condiments served with food.

When the dietary interviewer notices a large time gap, he/she should ask: "Did you have anything to eat or drink after the last meal? Anything before (insert time) and (before bed)?" If the participant hesitates and can't remember eating anything for a long period of time, the dietary interviewer may say: "Can you think of what you were doing (after work, at dinner/supper time, etc.)? Sometimes if we think about what we were doing, where we were or whom we were with, it helps to remember what we ate."

When the 24-hour dietary recall interview is completed, ask the participant, "in terms of the amount of food you ate, would you say this was close to the amount that you usually eat, a lot more than you usually eat, or a lot less than you usually eat?" This question refers to the overall amount of food for the day, not the type of food. The dietary interviewer records the participant's response. If the amount is identified as

“a lot more than they usually eat” or “a lot less than they usually eat”, ask what makes them say it’s a lot more or a lot less than usual.

ENTERING 24-HOUR DIETARY RECALL DATA INTO NDSR

The 24-Hour dietary recall data will be entered by a trained dietetic student/intern under the supervision of a Registered Dietitian. The dietetic student/intern will learn about the NCC Food and Nutrient database, review the NDSR 2011 Manual, and practice using the software.

APPENDIX 9A: DIETARY RECALL FORM

Baseline, 3 month, or 6 month — In-person or telephone interview

Participant ID: ________ — Interviewer ID: _______

[Introduce yourself and thank them again for participating]

I'm going to be asking you about what you ate and drank yesterday. I will be writing the information down on this form.

First, we'll make a list of what you ate and drank. Next, we will review the list. Then, I will ask you some more specific questions about the food and we'll figure out how much you had to eat.

In order to figure out how much you had to eat, we will be using these pictures and tools.

We need to know only what you actually ate. You should not feel embarrassed about any food, as there are no "good" or "bad" foods and no one eats just the right foods all the time. There are no "right" or "wrong" answers. Whatever you ate is okay.

This should take about 30 minutes. Do you have any questions before we start?

1st Pass

1. *Yesterday, what was the first time you had something to eat or drink?*
2. *What did you have at that time?*
3. [Prompt participant after finished answering previous question]: *Did you have anything else at that time?*
4. *What was the next time you had something to eat or drink?*
5. *What did you have at that time?*
6. [Prompt participant after finished answering previous question]: *Did you have anything else at that time?*
7. [Repeat 4-6 for additional times participant ate until finished with an outline of the day's intake]

2nd Pass

8. *Now we will review what we have so far.*
9. *At (time) you had (read all foods). Can you think of anything else you had at that time?*
10. *Did you have a beverage with that meal?*
11. *Did you have any snacks between meals?* [or] *Did you sample food as you prepared for the meal?*
12. [Repeat 9-11 for all meals at different times]

Comment [PS1]: If the participant asks why we want to be so specific and review so much, respond **"We know it can be hard to remember everything eaten the day before, so this will help you remember and will help us make sure that we have recorded everything. I want you to stop me at any time you feel like you can't think anymore or if you need a break."**

Comment [PS2]: Show the participant the plates and cups, FAB, food models, measuring cups and spoons if in person visit. If recall conducted over the telephone, make sure the participant has FAB with them.

Comment [PS3]: If the participant asks for approval of foods or wants to know if what they ate is healthy, respond **"At this time, we are just gathering information about the people participating in this study. But you will have the opportunity to learn about which foods are healthier choices by either reviewing the educational materials received in the mail or participating in the group classes; depending on which group you get put in. You will also have the option to attend the group classes at a later date if you get put into the group that received educational materials in the mail."**

Comment [PS4]: If participant says amount of food or starts giving more description on the food, acknowledge by writing under detailed description. Be polite and thank the participant for being detailed and remind them that you will return to this list to figure out more about the food and how much they had of it

Comment [PS5]: Make sure the participant says a time after midnight

Comment [PS6]: This is for participants who said they prepared the food.

3rd Pass

13. *Now we will fill in your list with more detail.*

14. *Where did you eat your meal?* [Repeat for all meals at different times.]
[Choose from below and record in table under location]:

- Home
- Work
- Friend's home
- School
- Day care
- Deli/take-out/store
- Restaurant/cafeteria/fast food
- Community meal program
- Party/reception/sporting event
- Other

15. *Next we'll go over our list and I will ask you some questions about each food.*

16. [For each food consider and ask when appropriate]:

1. **Type of food** - e.g. what type of _____ was it?
   i. **Form purchased** – e.g. fresh/raw, frozen, canned, juice, part of meat, margarine stick vs. tub vs. cooking spray etc.
   ii. **Brand name** – commercial brand of packaged foods (Indian brand pulses & grains), fast food restaurant chain and menu item name
   iii. **Nutrient content** – mainly fat content in dairy and packaged foods, low sodium canned vegetables and broths, diet soda, no sugar added canned fruits, whole egg vs. egg whites
2. **How was it prepared or cooked?**
   i. **Preparation method** – pan fried, deep fried, pressure cooked, boiled, baked, steamed, etc.; spices fried in fat
   ii. **Additions or eliminations to food** – list ingredients used; vegetable, fruit, pulses, or chicken skin kept or removed?
3. **How was it eaten?** Addition (and eliminations) to foods made at the table e.g. with ghee, jaggery, chutney, salt added at the table, milk or sugar added to coffee, fruit eaten with skin or without skin, salt or other toppings on fruit, chicken eaten without skin

17. [Determine amount (quantity and unit size) of food/mixed dish]
Show me how much of ________________ you ate. Remember, we would like to know the amount actually eaten or drunk, not the total amount on the plate or in the glass, or the amount that was prepared for the entire family.

- **Food specific unit** – e.g. slice of bread, chicken leg, an egg, small samosa, stalk of celery, 1 medium mango (record dimension as well if necessary)
- **Shapes** – e.g. circle (roti, chapatti, dosa, paratha, papad), rectangle, wedge (pizza), cube (dhokla, barfi, paneer), sphere (ladoo, gulabjaman),
- **Weight** – e.g. ounce, pound, grams (meat and fish other than chicken)
- **Volume** – e.g. cup, fluid ounce, liter, tablespoon, teaspoon, pint, quart, gallon
  i. For beverages, ask if contained ice - Was ice included in the amount reported?

18. [Double check to ensure that the amount reported is what was consumed and re-record appropriate amount] *Were you able to finish that?* [or] *Did you eat all of it?*

Comment [PS7]: This is where the person ate their meal, not where they got their meal from. If they got a packaged food or food from fast food restaurant chain, write the brand name under detailed description of food. See #16.

Comment [PS8]: For food prepared by the participant, attain recipe, ask **"Tell me how you made _________. It would be helpful to know how you made it, what you put in it and how much."** Record only (1) pertinent ingredients and (2) amount of each ingredient. Probe for fat, salt and skin kept on or removed from chicken, fruit or vegetables:
Fat: "Did you add any oil or ghee?"
Salt: "Did you add any salt?"
Skin: "Did you remove the skin from (chicken, fruit or vegetable) before preparing it?"
If a common ingredient in a familiar dish is not mentioned, ask "**What else did you put in __________?"**

Comment [PS9]: (1)For food prepared by the participant, determine how many servings were made with the dish's ingredients and ingredients' amounts they just described, ask **"How many people did this recipe feed until it was gone?"**

Comment [PS10]: For food NOT prepared by the participant (e.g. food eaten at restaurant or party), ask **"Could you tell me the types of food that was in ________that you saw or recognized?"** – This includes if they saw visible fat on meat and ate it.
AND
For sabji/curry, ask **"Was it somewhat dry or very wet?"**
If it was wet, ask:
"Was the gravy/sauce dense & thick or thin & clear?"

Comment [PS11]: (2) Only list pertinent ingredients:
1.Vegetables
2. Fruits
3. Protein
4. Grains
5. Dairy
6. Fat added
7. Salt added
Herbs and spices do NOT need to be included ...

Comment [PS12]: Use the amount estimation tools.

Comment [PS13]: For food prepared by the participant, distinguish again between the entire recipe made for everyone vs. serving size that actually was eaten by the participant

Baseline, 3 month, or 6 month
Participant ID: _________

In-person or telephone interview
Interviewer ID: ________

4th Pass

19. *Now we will review the record. Tell me if I have missed anything.*

20. *At (time) you had (amount) of (food). Is this correct?* [Repeat for all foods]

21. [At the end of each meal time ask]: *Did you have anything else at that time?"*

22. ***[If the ability to recall the intake is limited or if underreporting is suspected, try to get a mental picture of the day, especially in regard to the most likely times for meals and snacks. The following are some examples]:

    - Did you eat or drink anything before (insert time, before bed, etc.)?
    - Can you think of what you were doing (after work, in the morning, etc.)? Sometimes if we think about what we were doing, where we were or whom we were with, it helps to remember what we ate.

23. *Was the amount of food you ate for this day close to the amount you usually have, a lot more than you usually eat or a lot less than you usually eat?*

    - [If the amount is identified as "a lot more than you usually eat" or "a lot less than you usually eat", ask]: *What makes you say it's (a lot more or a lot less than usual)?* [Record if this was a typical day or a special occasion, holiday or during fasting.]

[Be sure to document not typical eating patterns, adding meaningful notes to explain why very large or small amounts are entered, companion foods are not seen, meals are missed, etc.]

Baseline, 3 month, or 6 month
Participant ID: _________

In-person or telephone interview
Interviewer ID: ________

**Date:**			
**Location:**			
**Time:**			
**Name of food**	**Detailed description of food**	**Quantity**	**Unit**

APPENDIX 9B: FOOD AMOUNTS BOOKLET

Note: The following pages are just for reference and are not to be printed and used during the 24-hour dietary recall as they are not in scale. Print the saved FAB interviewer and FAB participant PDF downloaded from the NDSR website and follow the instructions for printing in scale.

Food

Amounts

Booklet

Interviewer Copy

Food Amounts Booklet
Conversion Guide for Dietary Interviewers

When the participant shows a picture in the Food Amounts Booklet, convert to NDSR amounts per the following information.

Squares and Rectangles 1/4 inch grid (pg 1)	Circles (pg 2)		
Enter as fractions, e.g., 9/4 wide x 12/4 long x 5/4 high or use page 4 for thickness.<br>Each square is 1/4" x 1/4".<br>Select shape, cube (3 dimensions) or rectangle (2 dimensions) per NDSR.	Select shape, circle or sphere.<br>Enter diameter in inches (") per the following:		
	A = 1 inch<br>B = 2 inches<br>C = 2 1/2 inches	D = 3 inches<br>E = 4 inches<br>F = 5 inches	G = 6 inches<br>H = 7 inches

Wedges (pg 3)						
**STEP 1: Select the number that corresponds to the radius. Enter the radius in inches in NDSR**	**STEP 2: Find the number selected in step 1. Next, find the letter that matches to the length of the arc. Enter the number in inches you obtained as the width in NDSR**					
**1** = 4" radius (8" D[1])<br>**2** = 4 1/2" radius (9" D)<br>**3** = 6" radius (12" D)<br>**4** = 8" radius (16" D)<br>**5** = 9" radius (18" D)		**A**	**B**	**C**	**D**	**E**
	**1**	0.6"	1.1"	2.1"	2.6"	3.9"
	**2**	0.7"	1.3"	2.4"	2.9"	4.4"
	**3**	0.9"	1.6"	3.1"	3.9"	5.9"
	**4**	1.1"	2.1"	4.1"	5.1"	7.8"
	**5**	1.3"	2.4"	4.7"	5.8"	8.9"
For 3D[2] wedge, also use **Thickness** (pg 4, height=x/16) or **Squares and Rectangles** (pg 1, height=x/4)						

Thickness (pg 4)	Measuring Spoons (pg 5)	Eating and Serving Spoons (pg 6)	Measuring Cups (pg 7)
Each unit is 1/16" thick.<br>Enter thickness as fraction.<br>E.g.: 1 = 1/16"<br>2 = 2/16"<br>3 = 3/16"<br>18 = 18/16"	<u>Standard measures</u><br>1/2 teaspoon (TS)<br>1 teaspoon<br>1/2 tablespoon (TB)<br>1 tablespoon	<u>Teaspoons:</u><br>Level = 1 TS<br>Heaping = 2 TS<br><u>Tablespoon:</u><br>Level = 1 TB<br>Heaping = 2 TB	Standard measures<br>1/4 cup (CP)<br>1/3 CP<br>1/2 CP<br>1 CP

Glasses (pg 8-9) (Fluid Ounces, FO)			
1 A = 1 1/4 FO<br>1 B = 2 1/2 FO<br>1 C = 3 3/4 FO<br>1 D = 5 FO	2 A = 2 1/2 FO<br>2 B = 5 FO<br>2 C = 7 1/2 FO<br>2 D = 10 FO	3 A = 3 FO<br>3 B = 6 FO<br>3 C = 9 FO<br>3 D = 12 FO	4 A = 8 FO (1 CP)<br>4 B = 16 FO (2 CP)<br>4 C = 24 FO (3 CP)<br>4 D = 32 FO (4 CP)

Bowls (pg 10-11)				Wine Glass (pg12)	Mug (pg 13)
1 A = 1/8 CP<br>1 B = 1/4 CP<br>1 C = 3/8 CP<br>1 D = 1/2 CP	2 A = 3/8 CP<br>2 B = 3/4 CP<br>2 C = 1 1/4 CP<br>2 D = 1 1/2 CP	3 A = 3/4 CP<br>3 B = 1 1/2 CP<br>3 C = 2 1/4 CP<br>3 D = 3 CP	4 A = 2 CP<br>4 B = 4 CP<br>4 C = 6 CP<br>4 D = 8 CP	A = 2 FO<br>B = 4 FO<br>C = 6 FO<br>D = 8 FO	A = 2 1/2 FO<br>B = 5 FO<br>C = 7 1/2 FO<br>D = 11 FO

Mounds (pg 14-15)	Meats (pg 16) (Ounces, OZ)	Chicken (pg 17) (Medium, MD)	Fish (pg 18)
1 = 1 CP<br>2 = 3/4 CP<br>3 = 1/2 CP<br>4 = 1/3 CP<br>5 = 1/4 CP	All are edible portion<br>1 = 3 OZ<br>2 = 3 OZ<br>3 = 1 1/2 OZ	Edible portion<br>Wing = 1 MD<br>Drumstick = 1 MD<br>Thigh = 1 MD<br>Breast = 1 MD	Edible portion<br>1 = 2 OZ<br>2 = 3 OZ<br>3 = 1/2 OZ<br>4 = 1 OZ

[1] D = Diameter
[2] 3D = three dimensional

Squares and Rectangles

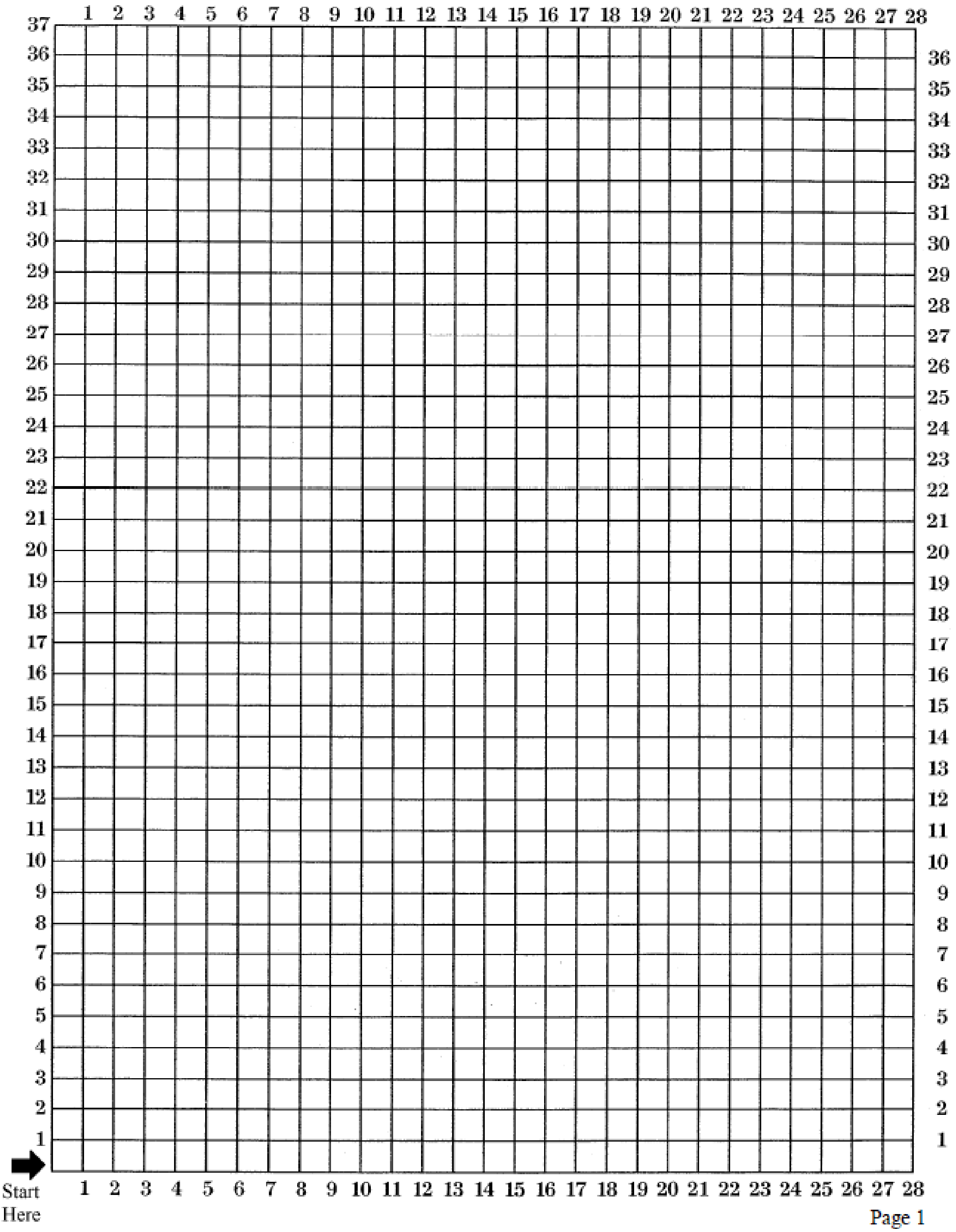

Circles

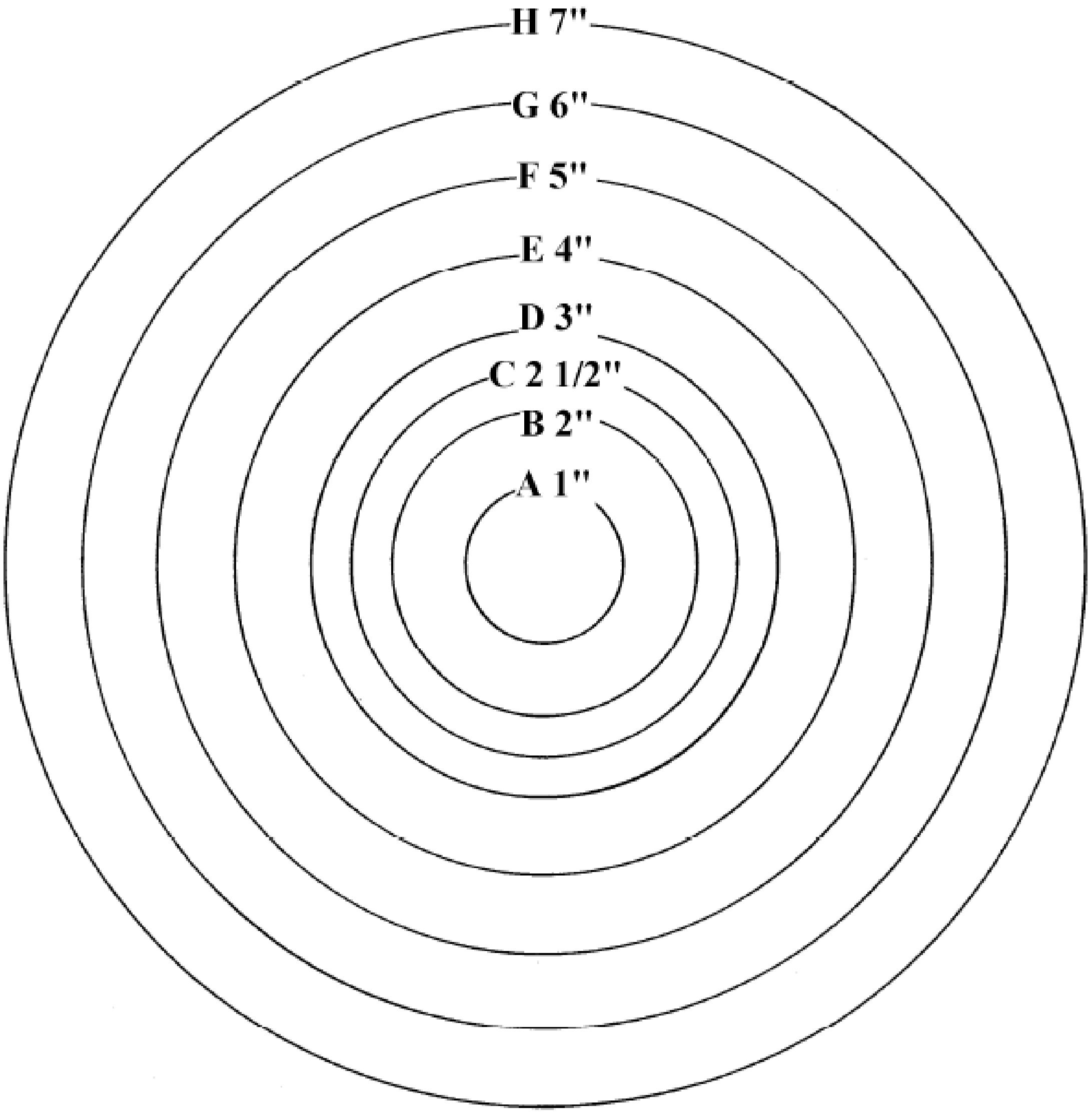

CONFIDENTIAL- Do not distribute, developed by Northwestern University

Wedges

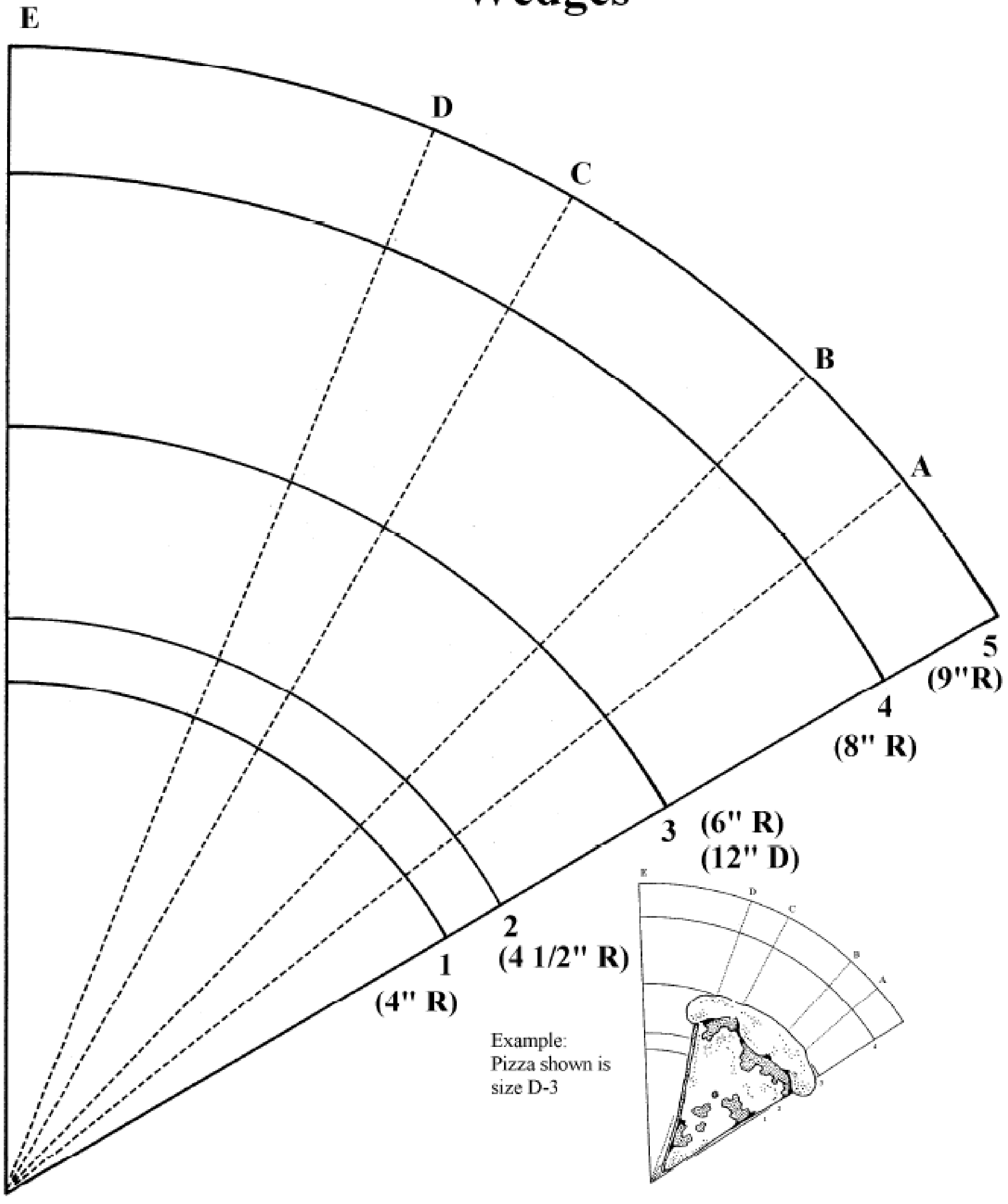

Page 3

Thickness

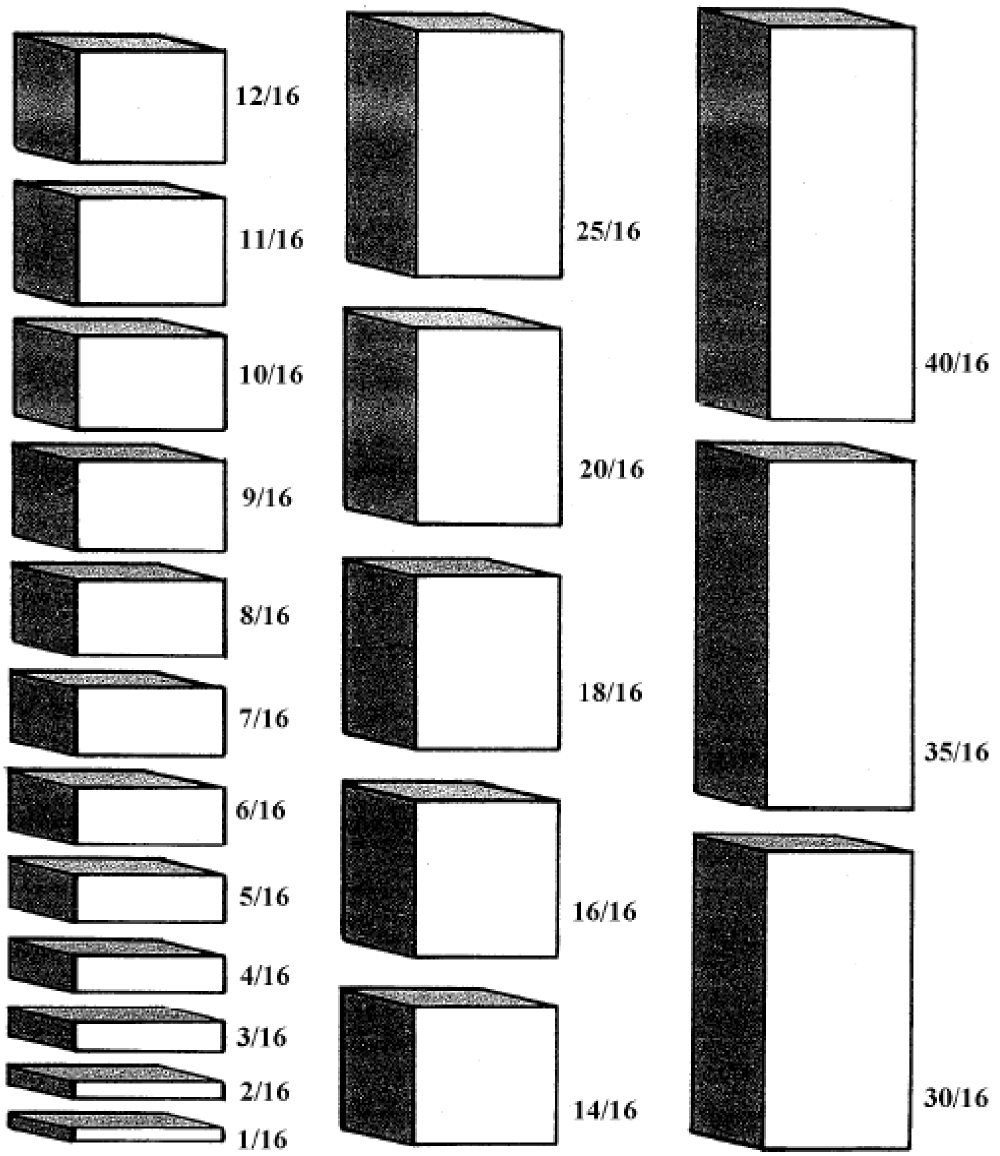

CONFIDENTIAL- Do not distribute, developed by Northwestern University

Measuring Spoons

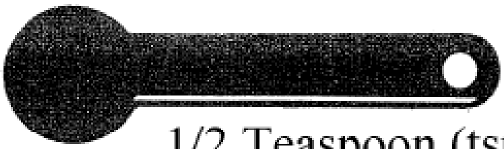

1/2 Teaspoon (tsp)

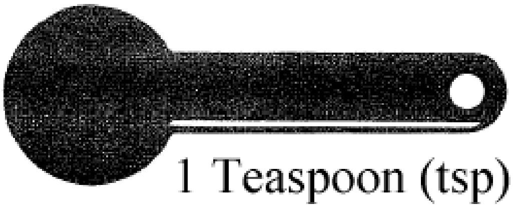

1 Teaspoon (tsp)

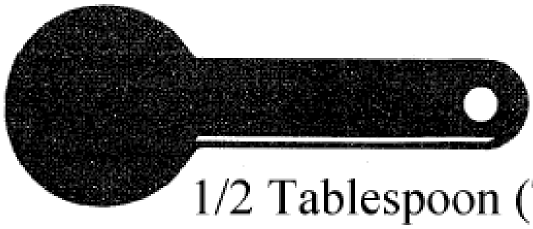

1/2 Tablespoon (Tbsp)

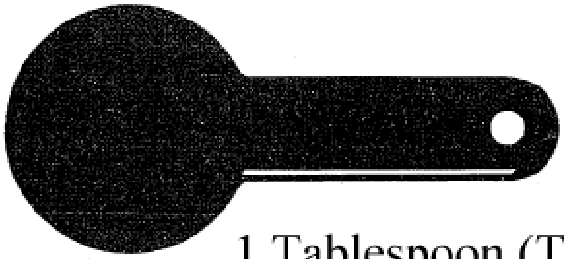

1 Tablespoon (Tbsp)

CONFIDENTIAL- Do not distribute, developed by Northwestern University

Eating and Serving Spoons

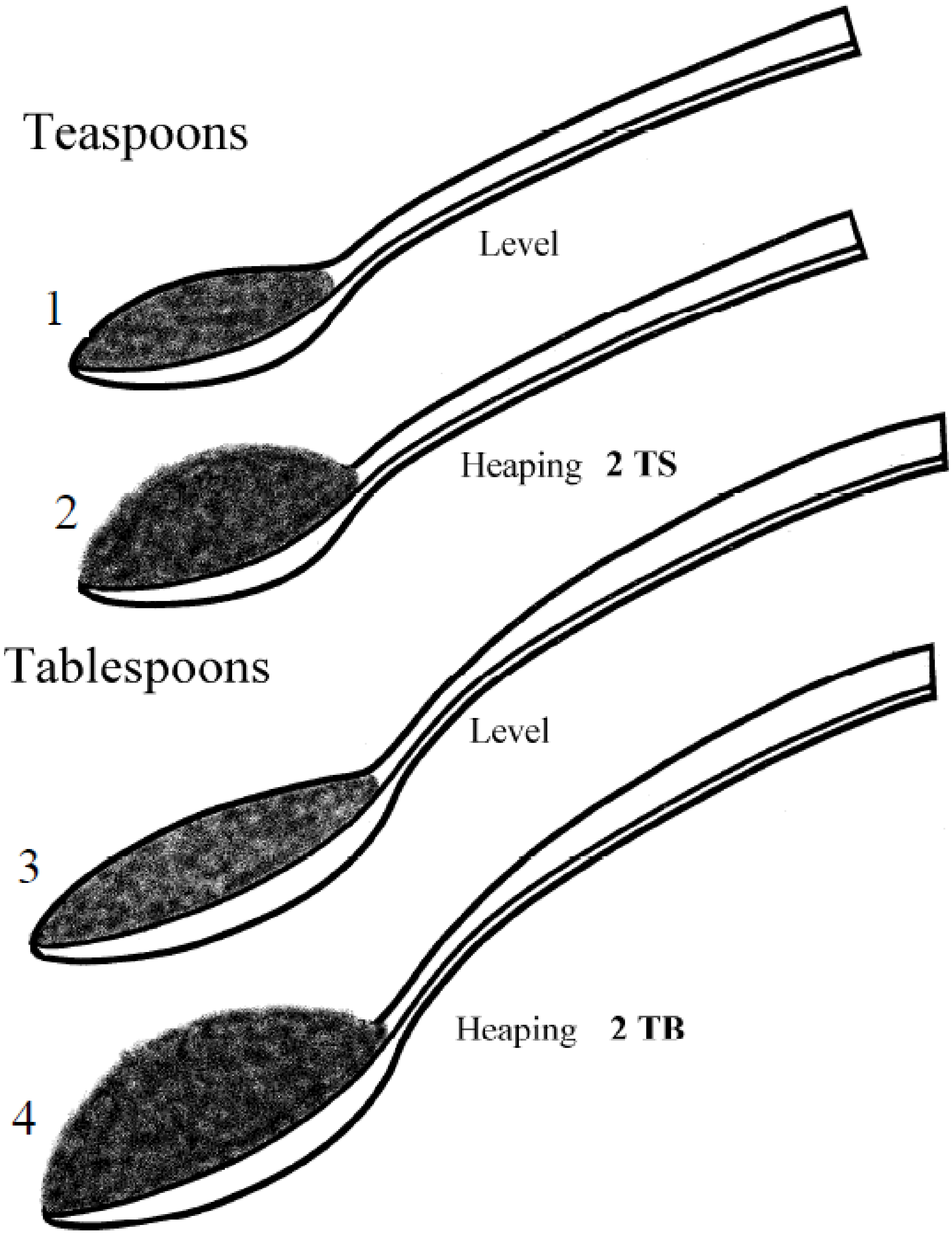

CONFIDENTIAL- Do not distribute, developed by Northwestern University

Measuring Cups

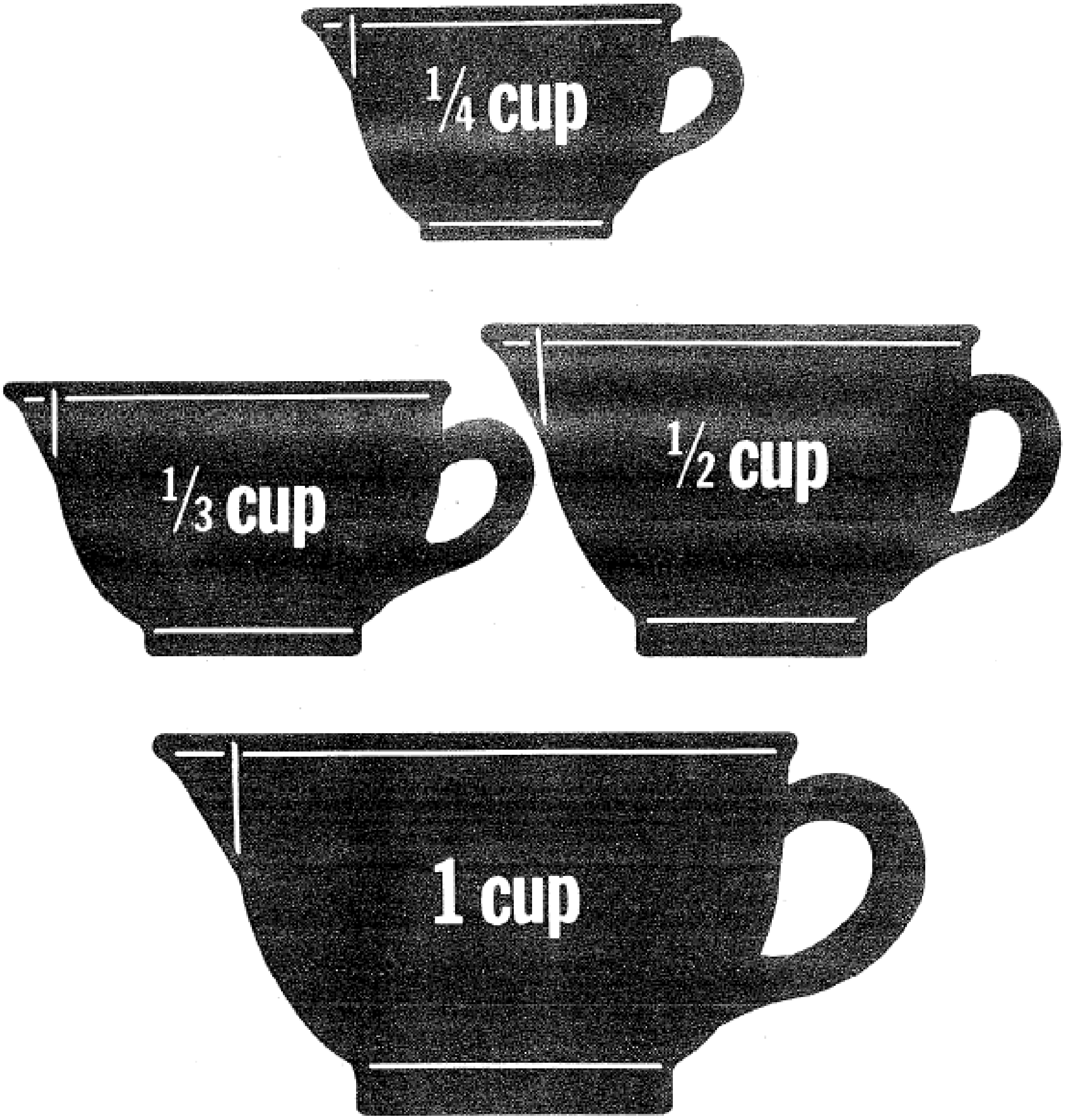

CONFIDENTIAL- Do not distribute, developed by Northwestern University

Glasses

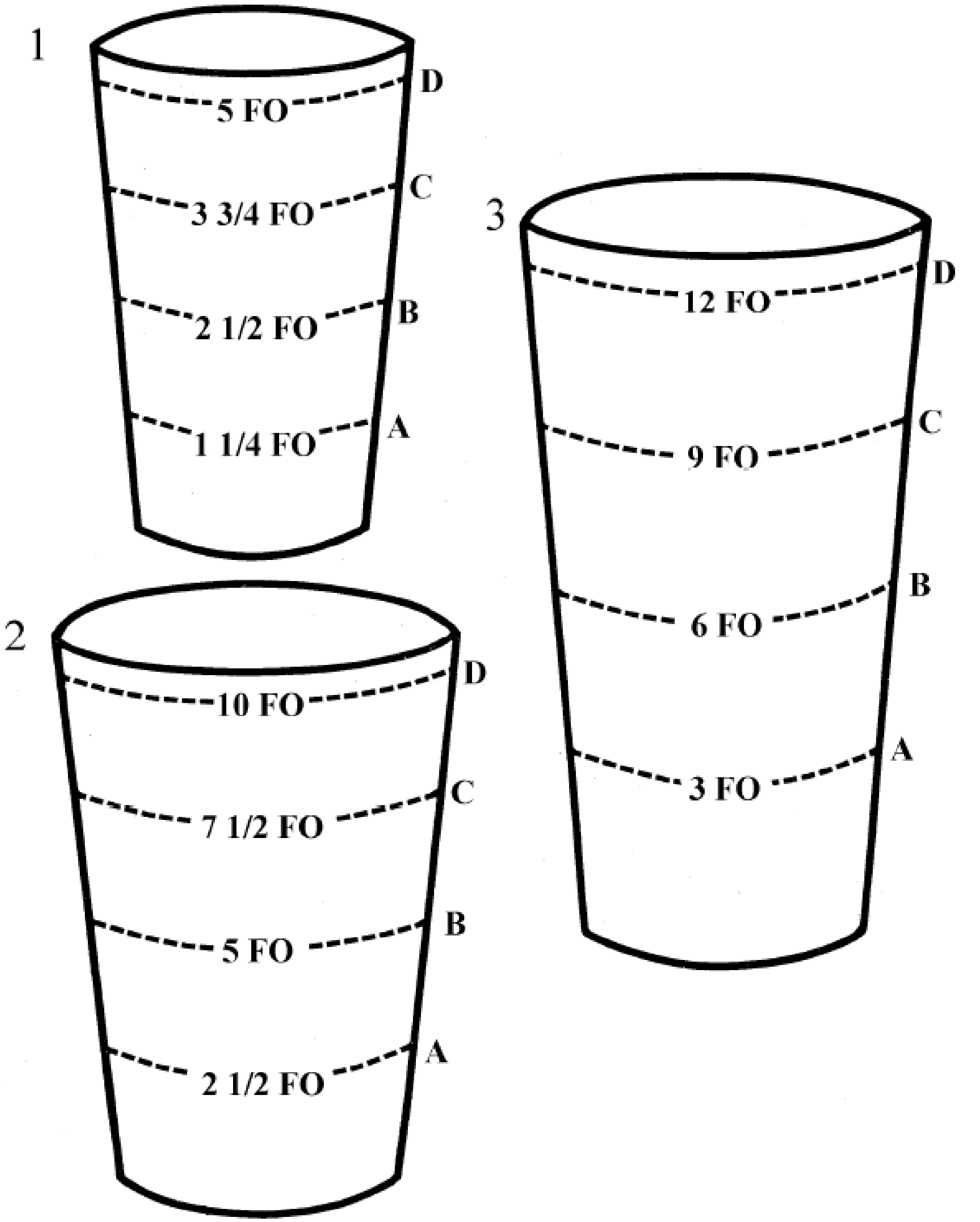

CONFIDENTIAL- Do not distribute, developed by Northwestern University

Glasses

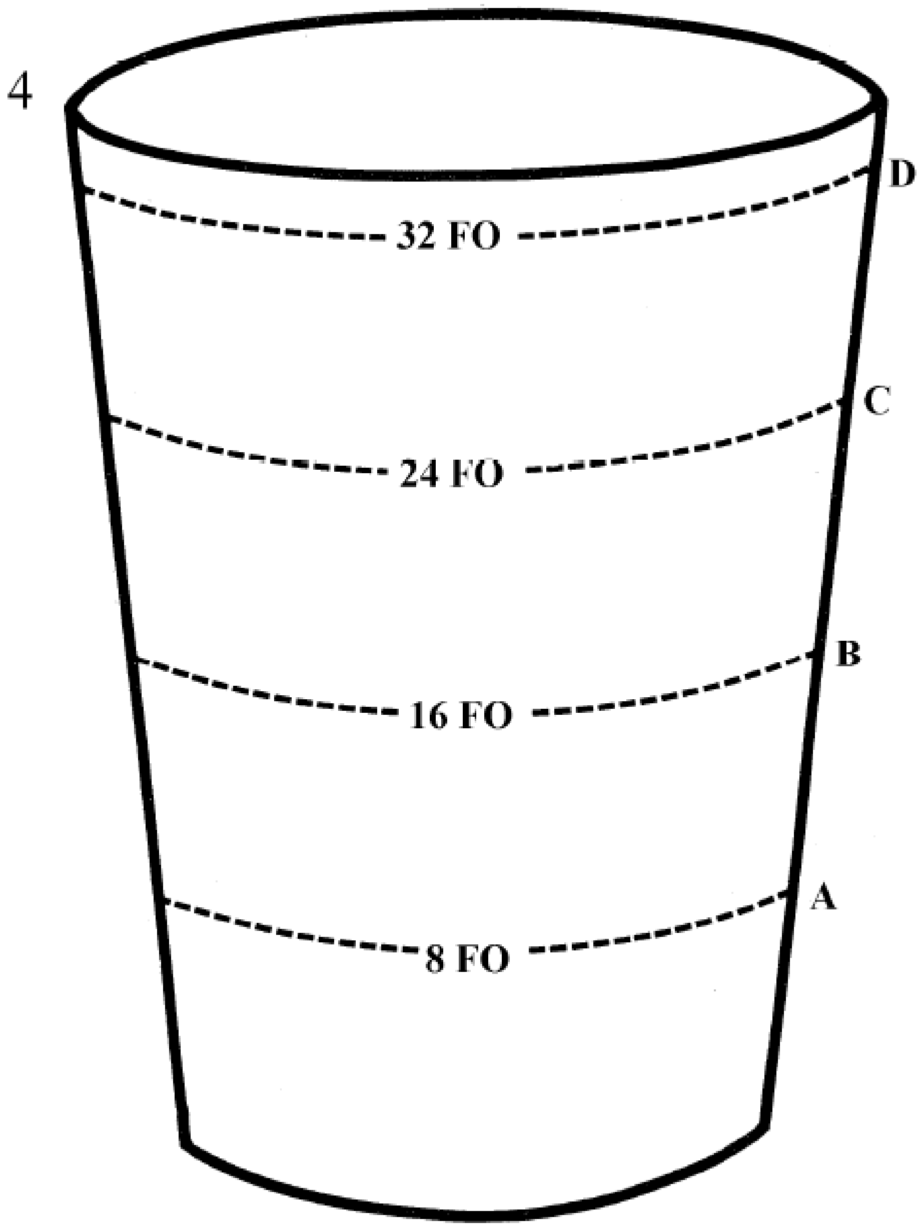

CONFIDENTIAL- Do not distribute, developed by Northwestern University

Bowls

1

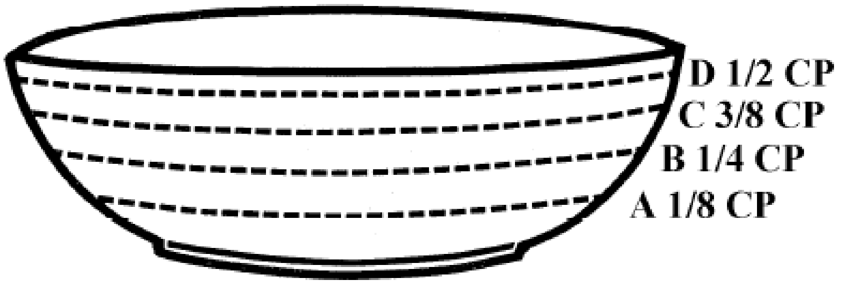

2

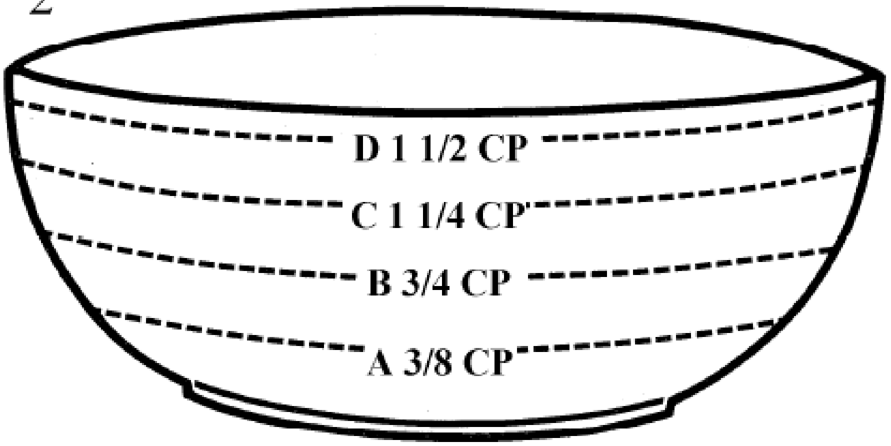

3

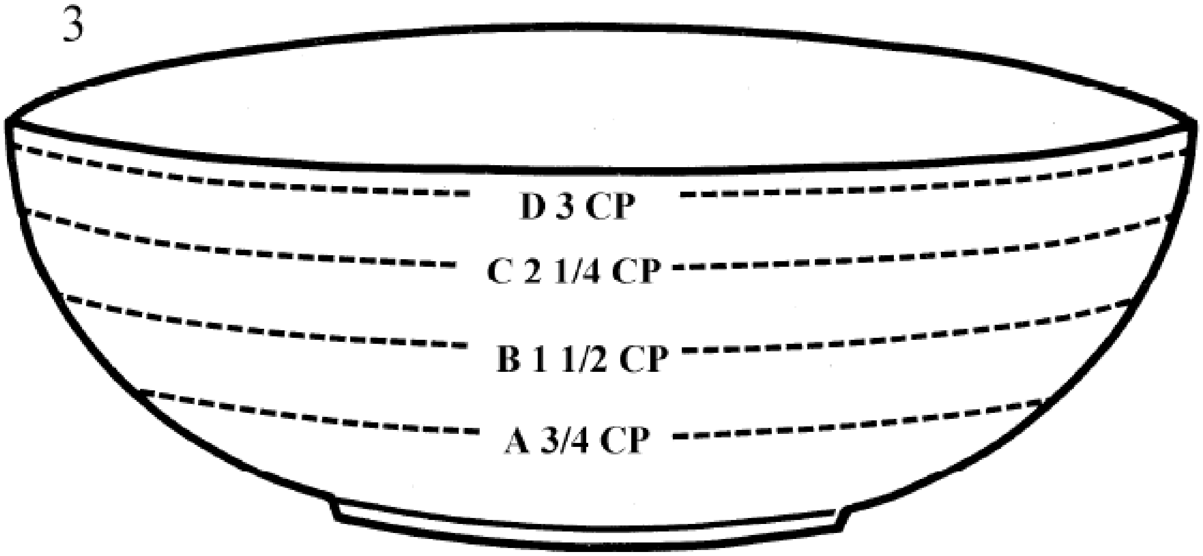

CONFIDENTIAL- Do not distribute, developed by Northwestern University

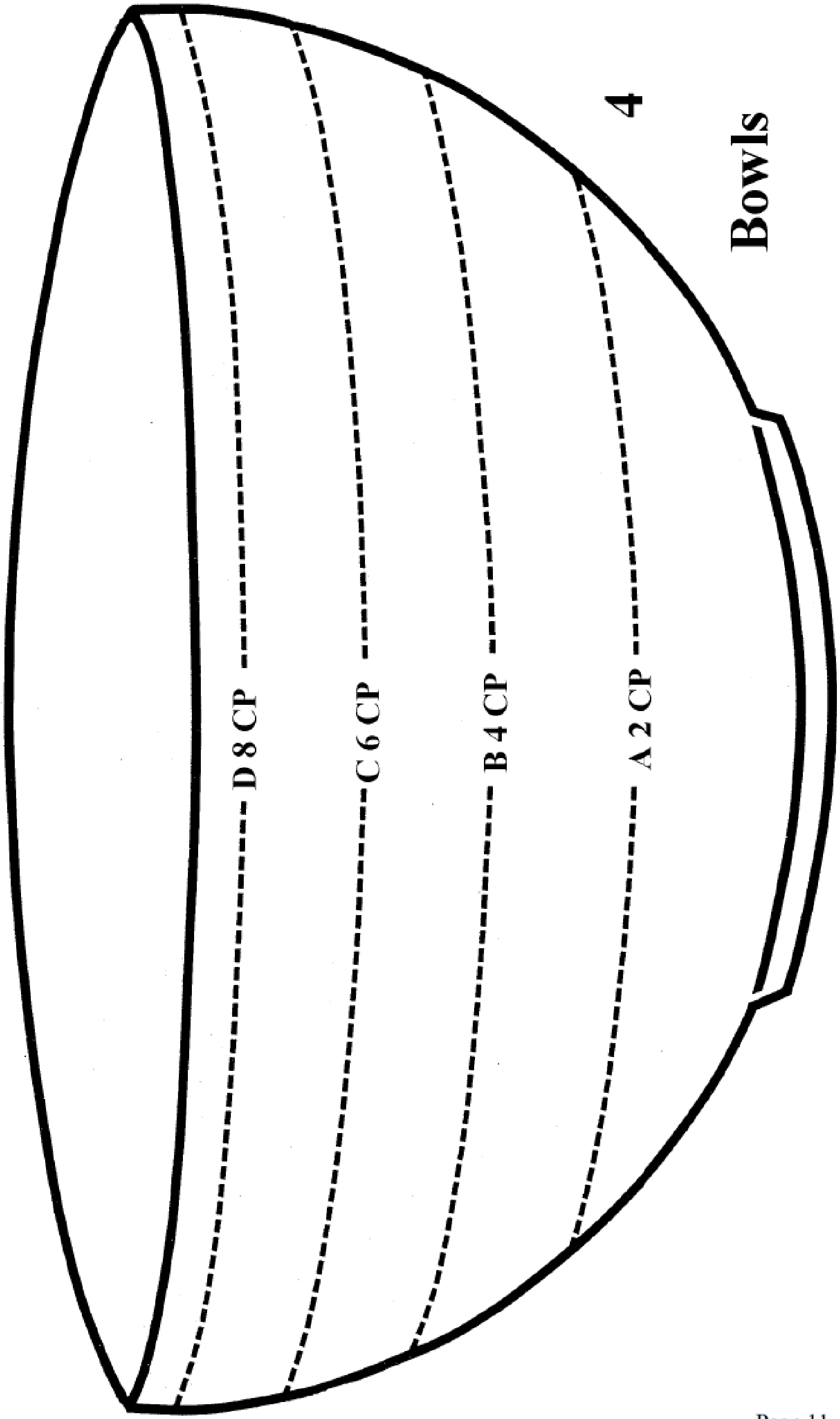

Page 11

Wine Glass

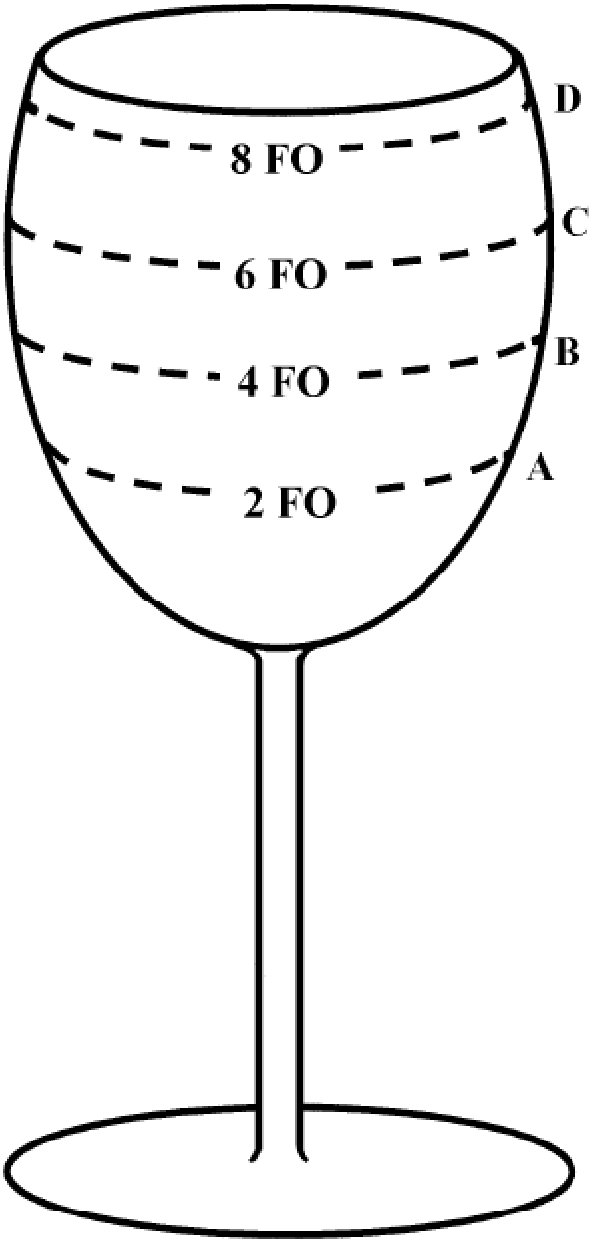

CONFIDENTIAL- Do not distribute, developed by Northwestern University

Mug

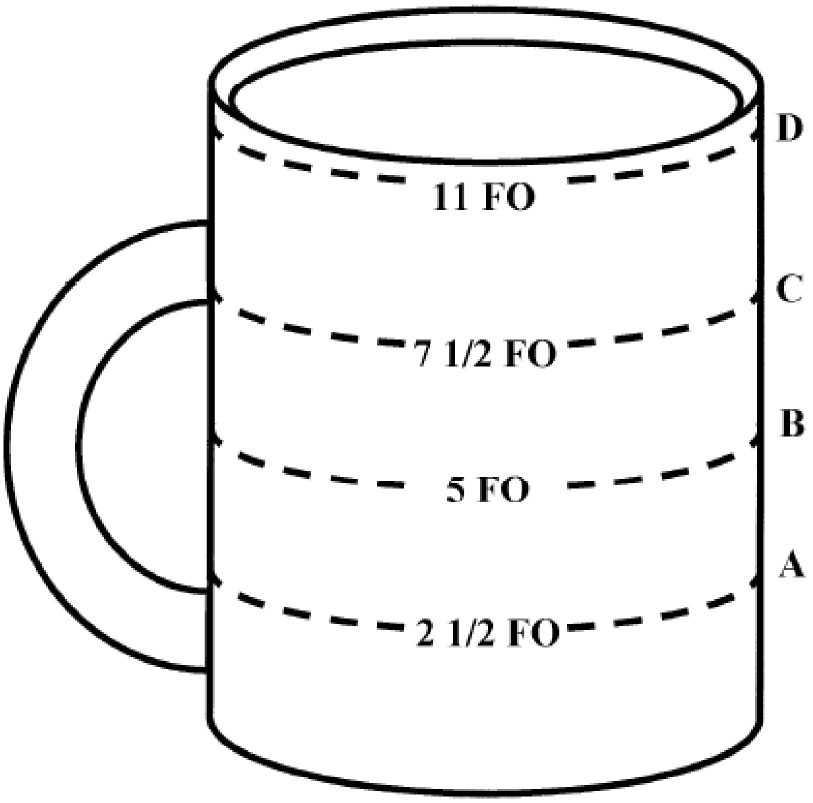

CONFIDENTIAL- Do not distribute, developed by Northwestern University

Mounds

1

1 CP

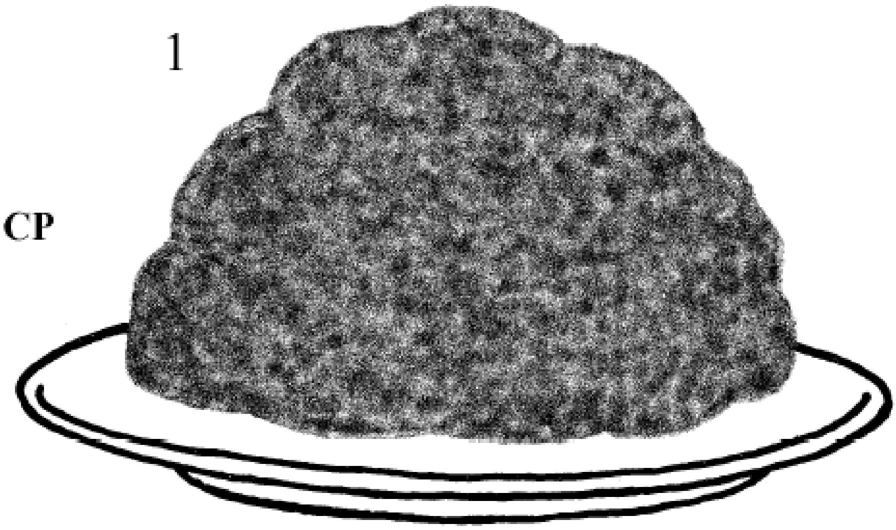

2

3/4 CP

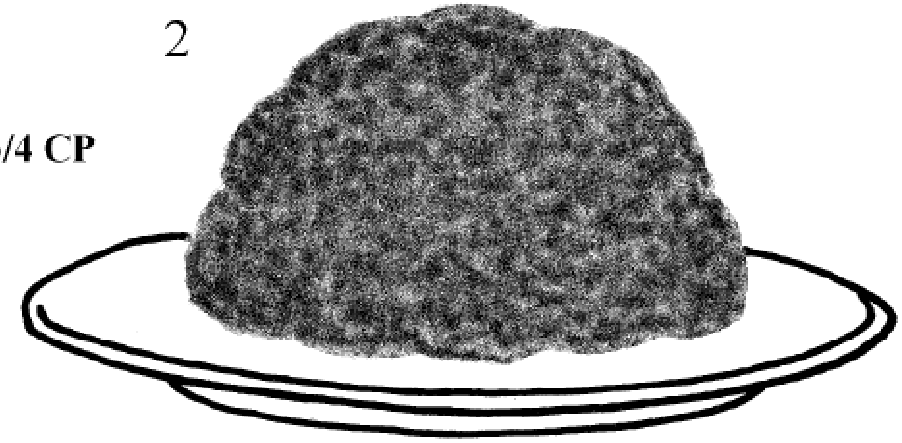

CONFIDENTIAL- Do not distribute, developed by Northwestern University

Mounds

3

1/2 CP

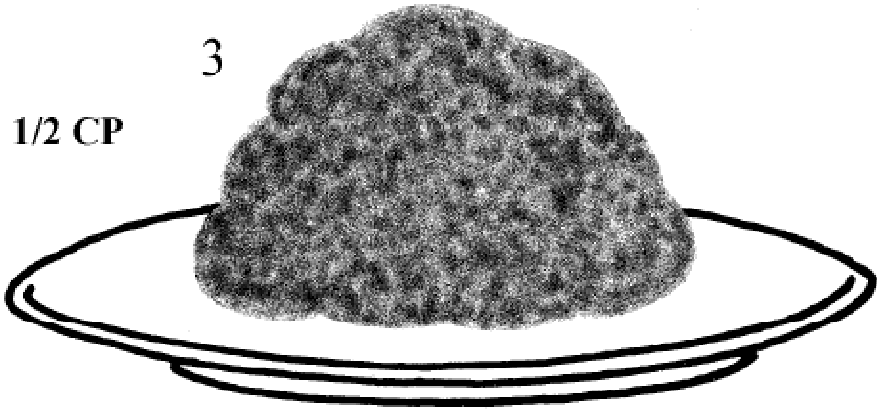

4

1/3 CP

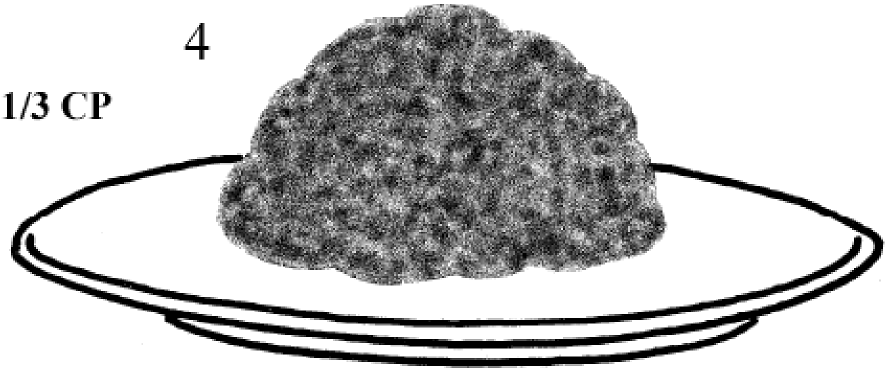

5

1/4 CP

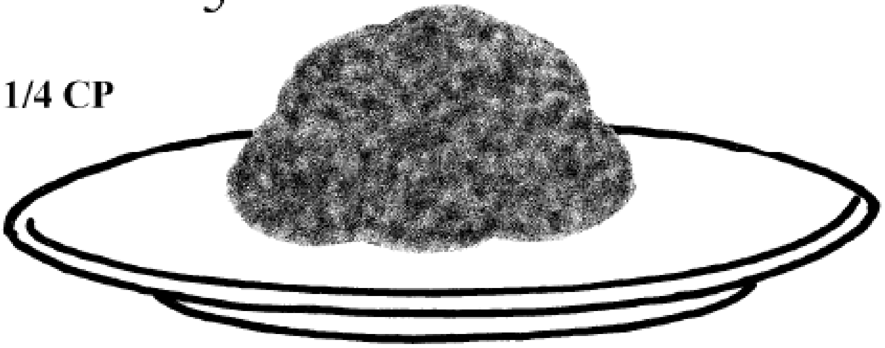

CONFIDENTIAL- Do not distribute, developed by Northwestern University

Meats

1

3 OZ

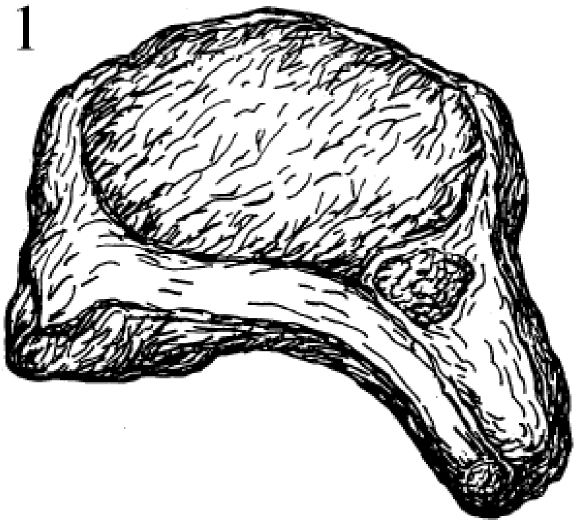

1 1/2 OZ

2

3 OZ

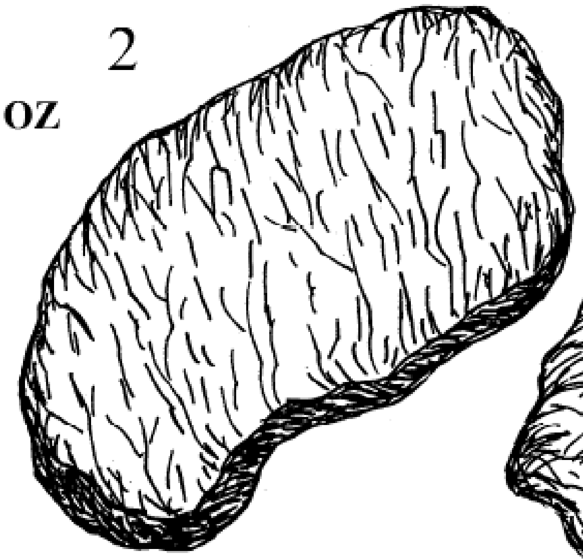

3

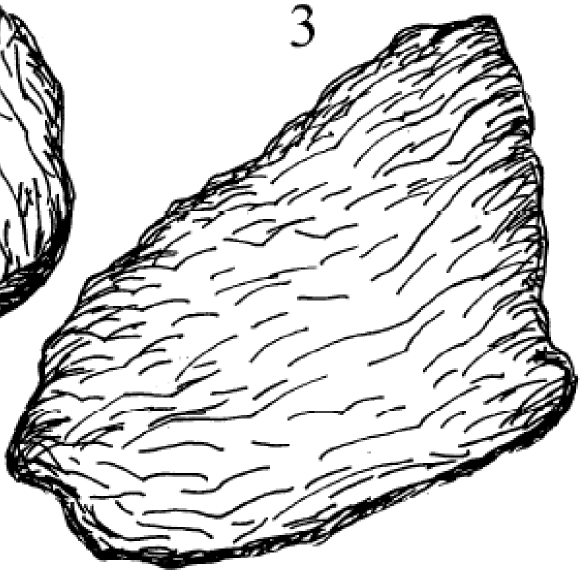

CONFIDENTIAL- Do not distribute, developed by Northwestern University

Chicken

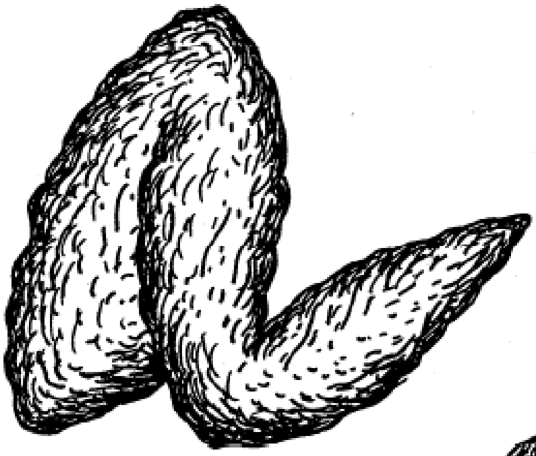

1 Wing

MD

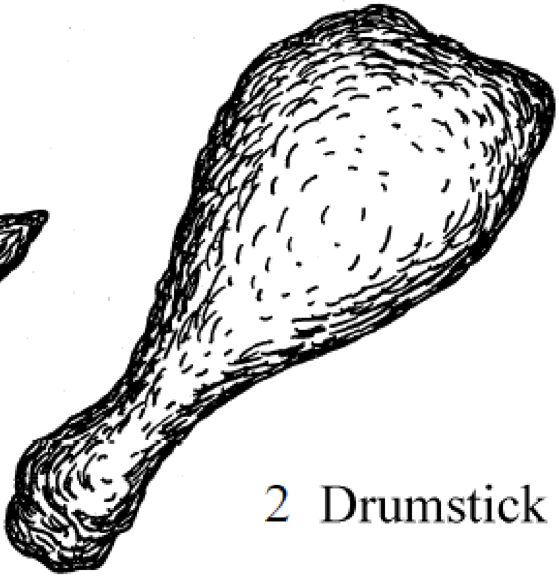

2 Drumstick

MD

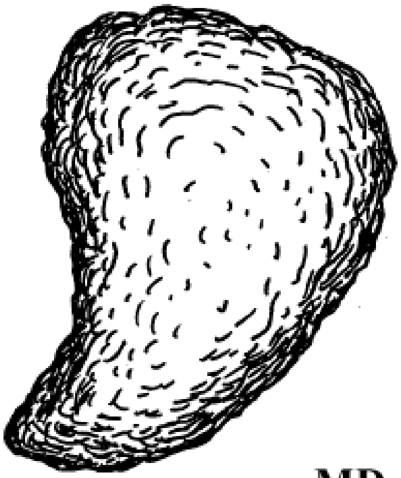

3 Thigh MD

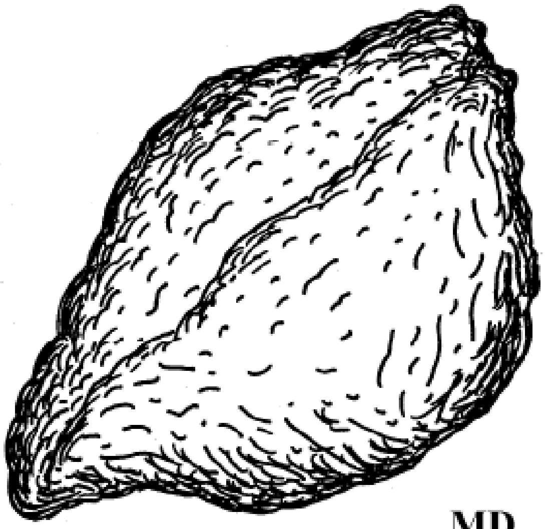

4 Breast MD

CONFIDENTIAL- Do not distribute, developed by Northwestern University

Fish

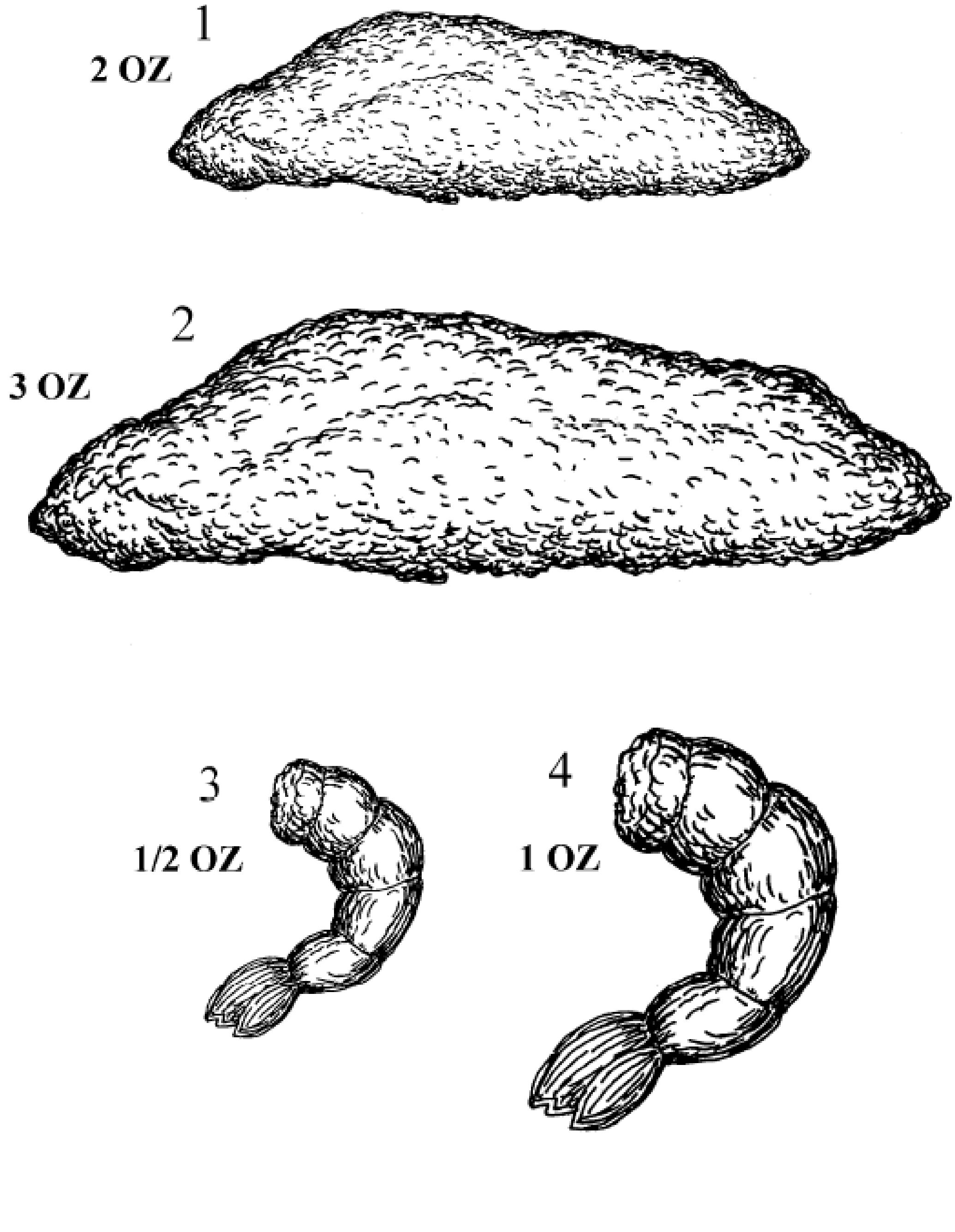

CONFIDENTIAL- Do not distribute, developed by Northwestern University

Adapted from: Van Horn LV, Stumbo P, Moag-Stahlberg A, Obarzanek E, Hartmuller VW, Farris RP, Kimm SY, Frederick M, Snetselaar L, Liu K. The Dietary Intervention Study in Children (DISC): dietary assessment methods for 8- to 10-year-olds. J Am Diet Assoc. 1993 Dec;93(12):1396-403.
Modified by Alejandra Valencia, Mary Stevens, Nutrition Coordinating Center, University of Minnesota for the Hispanic Community Health Study, Study of Latinos, 2007.

CHAPTER 10: SOCIAL NETWORK QUESTIONAIRRE

OVERVEIW
Social networks (SN) are social relationships that an individual has. Social Network Analysis (SNA) is one way to understand and analyze how social networks affect health.

The purpose of the SN questionnaire is to better understand how social relationships networks affect beliefs, attitudes and behaviors, and how social networks affect health, specifically diet and exercise. The SN questionnaire will be used to ask each study participant (also referred to as "ego") about his/her social network members' (also referred to as "alters") health attitudes, beliefs, and behaviors. Social network members are people that an individual interacts with or is close to, or someone whom they can discuss important matters about themselves.

SN QUESTIONNAIRE
Introduction
Provide a brief overview and purpose of the questionnaire to each study participant before beginning the questionnaire. A sample "overview" script is provided at the beginning of the questionnaire.

First name generator
A name generator is used to construct the participant's social network. Begin the questionnaire by asking the first name generator. Use the sample "introduction" script provided on the questionnaire to introduce the first name generator.

It may be hard for some people to understand whom the interviewer is asking about. Some people may respond by saying, "Are these people in my family?" In such cases, the interviewer needs to provide more clarification. Clarify by using the following prompt: ("This could be a person you tend to talk to about things that are important to you. This can include your family, friends, relatives, neighbors, co-workers or anyone else you discuss important matters about yourself").
Record the initials of the people the participant lists on "Roster A." Once the participant stops listing people, state the second prompt: "Is there anyone else that you discuss important matters about yourself?"). If the participant lists more people, continue to record their initials on Roster A. The general rule is to continue to prompt until the participant signals that those are all the people with whom they discuss important matters. A common response if the participant cannot think of anyone else might be, "No, those are all the people I discuss important things with."
If a participant lists two or more people with the same initials (ex. KS, KS, KS) use numbers after the initial to identify between them (ex. KS1, KS2, KS3).
Network questions
Record initials of all the people from "Roster A" on row A1 (see below for example).

Use the following prompt to let the participant know that you will now ask some questions about the people they have just listed: ("Now I will ask you some questions about these people that you have just listed"). Begin asking question A2 through question A21.

Many of the questions have responses that the interviewer can mark directly on the questionnaire (ex. Question A3). For other questions, the interviewer will need to use the appropriate "handcard" to enter the correct code for each response (ex. Question A2). If a question requires the use of a handcard, it will be listed with the question. Finally, there are some questions – for example "How old is [name]" – which requires the interviewer to enter the exact number given by the participant.

	Person 1	Person 2	Person 3	Person 4	Person 5	Person 6
A1 Initials or nickname	KS1	VP	KS2	YS	KS3	
A2 HANDCARD A Relationship (18=specify)	4	11	8	6	9	
A3 Is [NAME] male, female	1. Male 2. Female 3. REF	1. Male 2. Female 3. REF	1. Male 2. Female 3. REF	1. Male 2. Female 3. REF	1. Male 2. Female 3. REF	1. Male 2. Female 3. REF
A4 How old is [name]						
A5 HANDCARD B Race/Ethnicity (6 = specify)						
A6 HANDCARD C Religion						
A7 HANDCARD D Where does [name]						

For questions A2 through question A29, it is important to ask each question for all of the people listed before moving on to the next question. For example, question A2 asks about what the participant's relationship is with each of the people listed. The interviewer would ask how each network member (person 1, person 2, person 3, etc.) is related to the participant and would then enter the appropriate code using "Handcard A" (see above for example). Once question A2 has been asked for all the people listed, the interviewer will then move to question A3. Again, ask the question for all of the people listed before moving on to the next question.

Question A30 asks about the frequency of how often the participant talks to each person they list in "Roster A" and how often the people they list talk amongst themselves. Record the initials of the participant in the cells labeled "ego" and the initials of the people from "Roster A" in their corresponding cells. Remember to lists the initials on both row 1 and column 1 of the table. (See below for example)

A30 HANDCARD E
How often does [person 1] talk to [person 2], [person 3]...

0 Does not talk
1 Every day
2 Several times a week
3 Once a week
4 Once every two weeks
5 Once a month
6 A couple of time a year
7 Once a year
8 Less often than once a

		Ego	1	2	3	4	5	6
	Initials	AS	KS1	VP	KS2	YS	KS3	
Ego	AS							
1	KS1	1						
2	VP	1	1					
3	KS2	2	2	0				
4	YP	1	4	6	5			
5	KS3	4	4	0	5	6		
6								

Begin by asking how often the participant (ego) talks to each person (person 1, person 2, person 3 etc.) going down column two (see above for example). Once column two has been complete move to column three and ask how often person 1 talks to each of the other people listed (ex. how often does person 1 talks to person 2, person 3, person 4 etc.). Continue this pattern for all the people listed. Remember to enter the appropriate codes from "Handcard E" into the appropriate cells. Do ***not*** enter any codes into the gray cells.

Second name generator

The second name generator is more specific and asks about any other people the participant talks to specifically about their health that they have not already mentioned in the first name generator. Use question 1 on "Roster B" ("Do you talk to anyone else NOT on the list that you talk to about your health?") to prompt the participant to think about anyone else they have not mentioned with whom they talk to about their health.

Record the initials of the people listed in Roster B. Prompt ("Is there anyone else?"). Use "Handcard A" to ask about the relationship of the participant to each person listed in "Roster B." The interviewer does not need to ask question A2 through question A30 for people listed in "Roster B."

CLOSING

Review the questionnaire to make sure all the questions have been answered. If complete, thank the participant for their time.

CHAPTER 11: LINKAGE TO PRIMARY CARE

OVERVEIW

Involving and communicating with research participants primary care providers is an important part of the study. Study participants will be encouraged to discuss their tests results with their primary care provider and any follow-up or clarifying information about tests results will be provided by Dr. Namratha Kandula, if requested.

LINKAGE TO PRIMARY CARE THROUGH TELEPHONE ASSISTANCE

Participants who already have a primary care provider (PCP)

Study participants, in the control and intervention arms, will be asked to provide the name and contact information of their PCP. All test results, anthropometrics and blood work, will be sent to the participant's PCP with a brief letter letting the PCP know that the patient is in the study and alerting the PCP to any abnormal results.

Participants will be sent a letter with their test results and will be instructed to follow-up with their PCP. The research coordinator will contact any participant with urgent values and help participants arrange follow up with the PCP within 1 week. All other participants will be instructed to follow-up with their PCP within 8-12 weeks. The research coordinator who conducted the baseline visit will ask the participant if s/he wants assistance making the appointment. If the participants requests assistance, the research coordinator will call the PCP office and schedule the appointment within 6-12 weeks and notify participant.

Participants who do not have a primary care provider

If the study participant does not have a primary care doctor, the study team will provide referrals to local primary care providers and telephone assistance in making an appointment. The research assistant will assign the participant to a PCP in a free-clinic in the community. The research coordinator will call free clinics and make an appointment during the week, within the next 6-12 weeks. They will then relay the time and date of the appointment to the study participant through a phone conversation, a mailed letter, or a phone message. The participant can also elect to make their own appointment.

The main goal is that all the participants see their provider at least once during the 6-month study follow-up.

Figure 1. Flow of patients in linking to primary care

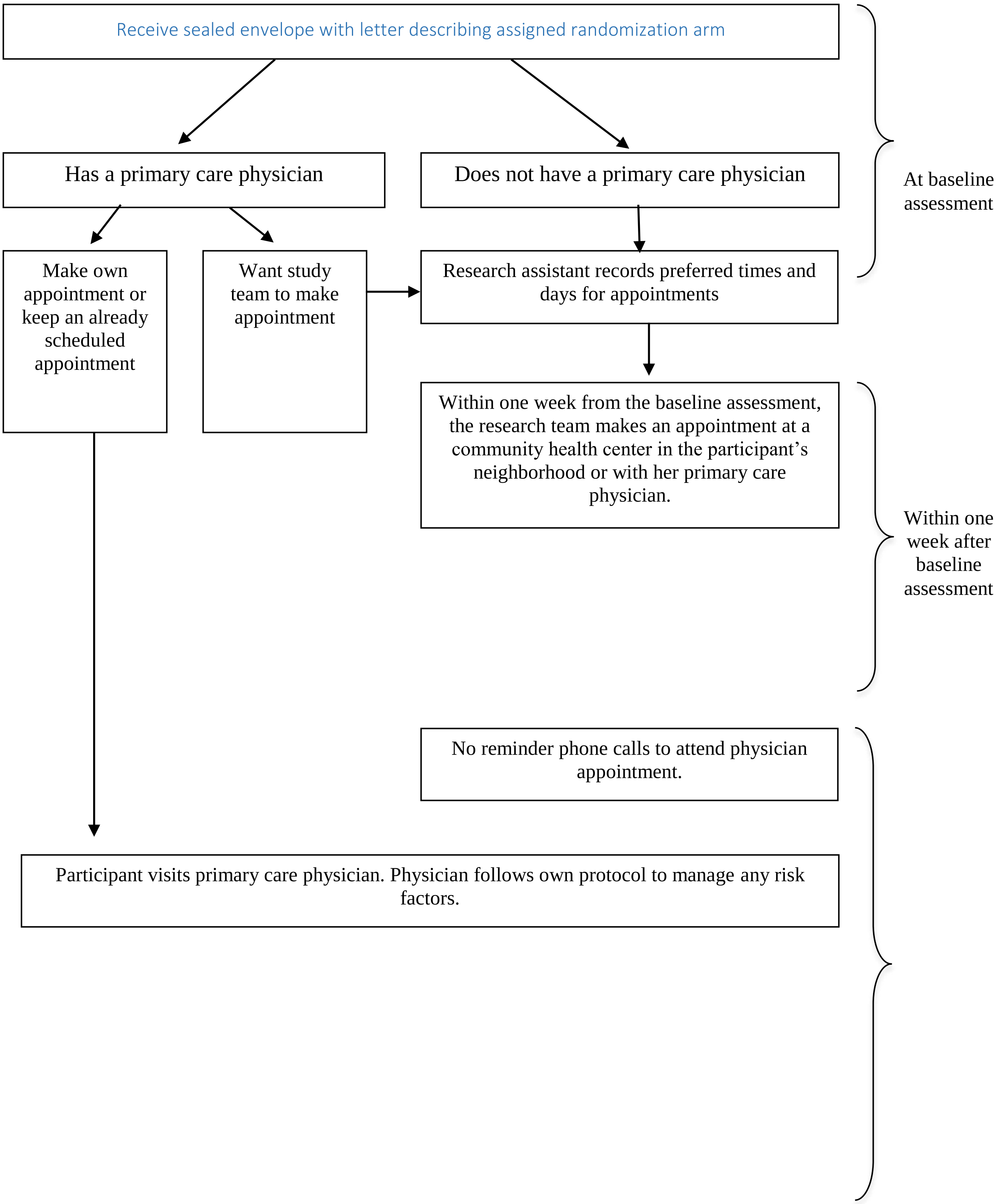

CHAPTER 12: CONTROL GROUP

OVERVIEW

In a Randomized Control Trial (RCT), participants are evenly placed in different study groups. Participants do not have control over which group they may be assigned to. People who take part in studies requiring randomization are generally placed in a group based on chance, not choice. Some participants are placed in the "intervention" group, and the others are placed in the "control" group. Below is an overview of what each group (control and intervention) will receive as part of this study.

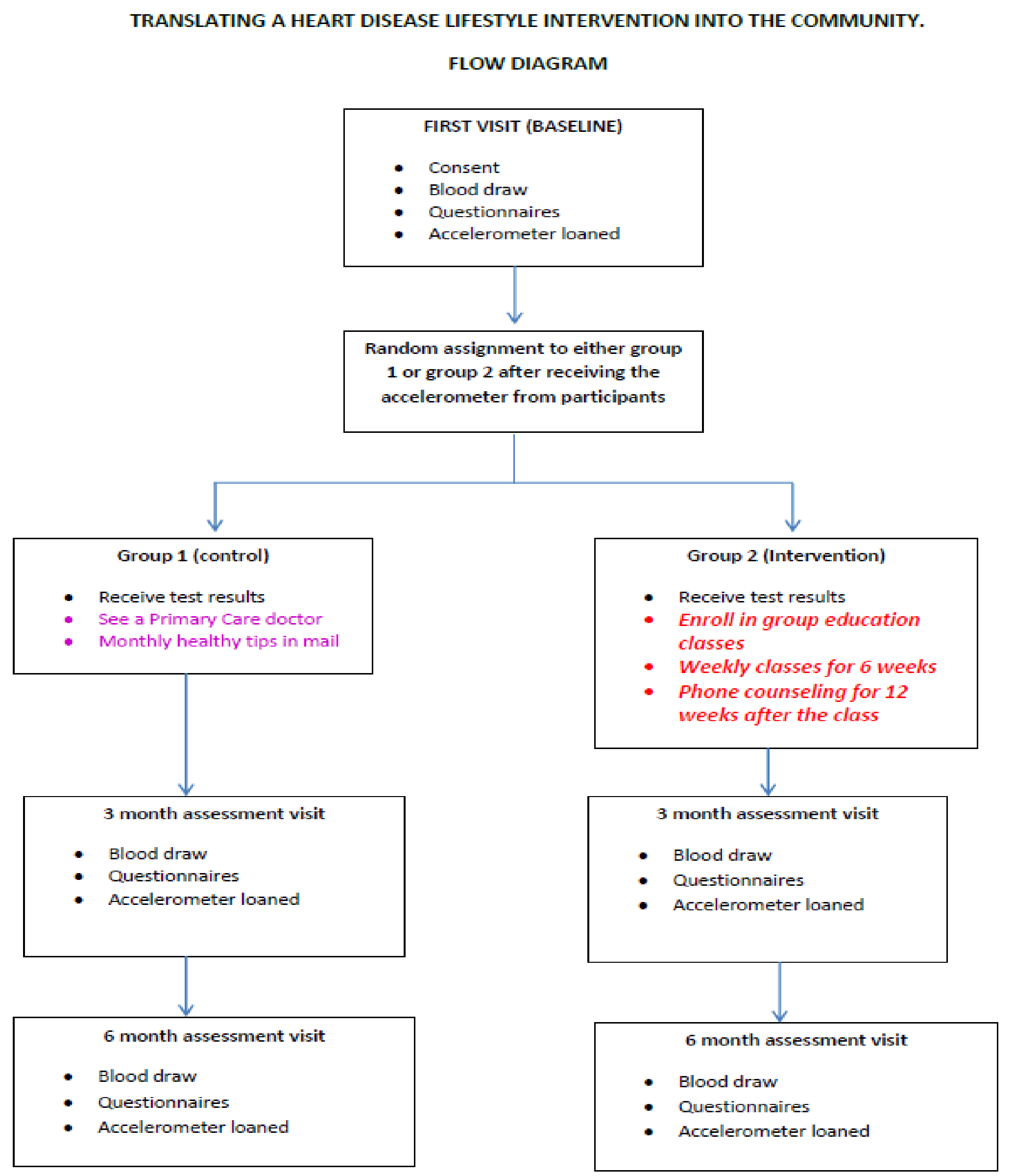

SAHELI CONTROL GROUP

The SAHELI study is a Randomized Control Trial (RCT). The SAHELI study has two study groups: the Print Health Education group (control group) and the Lifestyle Intervention group (intervention group). Participants will be randomly assigned to one of these two groups (See Chapter 15 Randomization for details). The randomization will occur after the baseline visit has been completed and after the accelerometer has been returned.

Participants in the Print Health Education group will receive blood tests and will be asked to follow-up with their primary care provider (PCP). In case they do not have a primary care provider, they will be referred to a provider. Participants will also receive monthly health education material about heart disease prevention as part of the study.

Assessment visits (Baseline, 3 months, and 6 months)

During each assessment visit, all control group participants will complete the following:

- Informed Consent and Participant Contact Information (only baseline visit)
- Demographics (only baseline visit)
- Urine Sample and Pregnancy Test (if applicable)
- Medications Questionnaire and Medication Inventory
- Seated Blood Pressure
- Fasting Labs
- Anthropometry
- Questionnaires
- 24-hour food recall
- Accelerometer usage instructions

Participants will receive their lab results when they return the accelerometer 7 days after each visit.

PRIMARY CARE REFERRAL

At the time when participant pick up their lab results, participants in the control group who do not have a primary care provider will receive primary care information (See Appendix 12A). The study staff will provide basic information about participants' lab results; however, it will be up to the participant to discuss their lab results in detail with their health care provider and seek appropriate care if needed. We will follow-up with these participants and ensure that adequate help, if needed, is provided in order to schedule an appointment to see a PCP. Help may be needed in areas like calling a providers office to set up an appointment for finding a clinic that provides free or care on a sliding fee scale.

PRINT HEALTH EDUCATION

Participant in the control group will receive health education material about heart disease prevention in the mail every month. The mailings will be sent via mail during the first week of the month. This information will be made available in both English and Hindi. Participant will be asked whether they prefer to receive information in Hindi or English.

Print materials will be housed in the folder on the Northwestern University server at:

P:\South Asian\R21\Control group materials

The health education material topics are the following:

1st month – Looking after your heart
2nd month – Eating healthy foods
3rd month – Staying active
4th month – Salt and fat
5th month – Coronary heart disease
6th month – Stress
Optional – Smoke cessation

Print materials will be reviewed every 6 months by study staff to ensure that the information is accurate and up to date. Decision to change or update the materials will be made in conjunction with the study PI.

APPENDIX 12A: PRIMACY CARE REFERRAL RECO

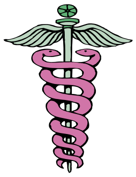

Where Can I Get Health Services?

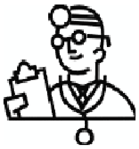

There are several community clinics that provide exams and other services at a low fee.
****You can see a doctor regardless of your immigration status or ability to pay.****
Following are some close to your school:

- -

Name: Access Community Health Network
Address: 2655 W. Peterson, Chicago, IL 60659
Hours: Monday-Friday 10:00am-5:00pm ; 1st Saturday of each month 9:00am-1:00pm
Cost: Sliding scale; Insurance accepted
Languages: English, Spanish, Urdu, Hindi, Romanian, American Sign Language
For information and appointments call: 773-271-8880

- -

Name: AHS Family Health Clinic
Address: 2820 W. Peterson, Chicago, IL 60659
Hours: Mon., Wed., Thurs., Fri. 9:00am-5:00pm; Tues. 11:00am-7:00pm; Saturday 9:00am-1:00pm
Cost: Sliding scale; Insurance and Medicaid accepted
Languages: Spanish, Urdu, Hindi, Marathi, Telugu, Gugarati, Korean, Thai, Vietnamese
For information and appointments call: 773-761-0300, 1-800-597-5077

- -

Name: Chicago Department of Public Health Uptown Neighborhood Health Center
Address: 845 W. Wilson, Chicago, IL 60640
Hours: Mon., Wed., Fri. 8:00 am—4:00 pm, Tues., Thurs. 10:00 am—6:00 pm
Cost: Sliding scale; Insurance and Medicaid accepted
Languages: English, Spanish, call for interpreter for other languages
For information and appointments call: 312-744-1938

- -

Name: Erie Helping Hands Clinic
Address: 4759 N Kedzie, Chicago, IL 60625
Hours: Mon., Wed., Thurs., Fri. 8:00am-4:00pm; Tue. 8:00am-8:00pm
Cost: Sliding scale (dependant on income and insurance)
Languages: English, Spanish
For information and appointments call: 773-588-9640

- -

Name: Heartland Health Outreach
Address: 1015 W. Lawrence, Chicago, IL 60640
Hours: Mon., Wed., Fri. 8:30am-5:00pm; Tue., Thurs. 8:30am-8:00pm; every other Sat. 9:00am-12:00pm
Cost: Sliding scale; Insurance accepted
Languages: More than 40 (call for an interpreter)
For information and appointments call: 773-275-2586

- -

Name: Swedish Covenant Hospital—Family Practice Center
Address: 5140 N. California Suite G400, Chicago, IL 60625
Hours: Monday-Friday 9:00 am—5:00 pm
Cost: Insurance, Medicaid accepted, discounted rate available for self-pay patients
Languages: English and Spanish
For more information and appointments call: 773-989-3806

Information Provided by Healthy Albany Park Coalition
Kathryn Kruse c/o World Relief, 3507 W. Lawrence, Suite 208 Chicago, IL 60625
Phone: 773-583-9191 ex269, healthyalbanypark@gmail.com

CHAPTER 13: SAHELI INTERVENTION

Session 1: What is a heart attack? & 3 things to talk to your doctor about

Purpose:

Participants will be introduced to a heart attack, heart attack risk factors and how a heart attack can be prevented.

Session length:

80 minutes

Notes:

Activity 4 & 5 are more discussion based and can be done in smaller groups if needed.

Overview of materials needed for the session:

Media equipment
- *Video recorder
- *Audio recorder(s)
- Laptop
- Projector
- Speakers

Media
- “What is a heart attack?” video
- “3 things to talk to your doctor about” video
- Activity 2 Understanding your results PowerPoint
- Activity 3 Checking Cholesterol Scenario PowerPoint
- Activity 5 Putting it in Perspective PowerPoint
- Session 1 Review PowerPoint

Other supplies
- Box of pens
- Participants’ folders with business cards, lab results, and small notepad
- Activity 1 “Cards” (five total)

Handout(s) for participants:
- Class evaluations

***Remember to start the video and audio recordings.** The video recorder should record one of the educators as they teach the session and an audio recorder should be used for each group.

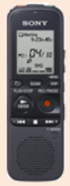
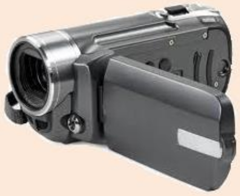

Introduction: 8 minutes

Materials needed:
Other supplies
- Participant's folders with business cards, lab results, and small notepad
- Box of pens

1. Thank the participants and introduce yourself. Let the participants know that the sessions will be video recorded.

    - *"Thank you for participating in the South Asian Heart Disease Prevention study. My name is __________ and I am the health educator who will be leading the sessions. We will be video recording the instructor and your voices may be heard during the sessions. We are doing this in case any of you need to review the class later on. This recording will only be used for the participants in this class. Does anyone have any questions about this before moving on?"*

2. Have all the participants introduce themselves and state what they hope to learn from these classes, and set class expectations.

    - *"We would like to start by getting to know a little about everyone here. Let's go around the room and state your name and what do you want to learn from these classes."*

3. Give an overview of the session layout and set class expectations.

    - *"Our goal for these six sessions is to provide you with clear information and to help you use that information in your everyday life. At each session, we will be watching videos. The videos were developed by doctors and researchers at Northwestern University with the help of patient and community member feedback. We will also be doing various activities to help us understand what we learn from the videos."*
    - *"Before we begin, we would like for everyone to remember that at each class:*
        - *Please turn off all cell phones,*
        - *Make sure to speak one at a time,*
        - *Remember that everyone has a right to their own opinion and we should respect each other's opinions,*
        - *Please <u>attend all six classes</u>, after the six classes we will also be calling you on the phone to help you use the information you learned in class. At the end of the six classes you will receive a certificate of completion. This is important because you can take what you have learned and help others."*
    - *"Thank you, and now let's begin with our first video!"*

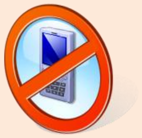

CONFIDENTIAL- Do not distribute, developed by Northwestern University

Watch video: "What is a heart attack?" 8 minutes

Video Objectives: After watching the "What is a heart attack?" video, the participant will be able to define a heart attack, understand how a heart attack happens, and explain how a heart attack can be prevented.

Materials needed:
Media equipment
- Laptop
- Projector
- Speakers

Media
- "What is a heart attack?" video

1. Introduce the video

    - *"Our first video is going to talk about what is a heart attack, how it happens and how we can prevent it. After the video, we will briefly discuss what we learned and then do activities that will help us better understand the information from the video."*

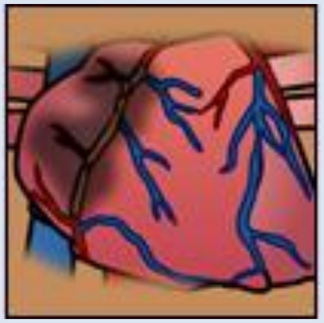

2. After the video, ask the participants to summarize the video and if they learned anything new from the video.

    - *"Can someone tell me what the video talked about?"*
    - *"Did you learn anything new that you didn't know before?"*

Activity 1: The link between lifestyle, the body and a heart attack 10 minutes

Objectives: The purpose of this activity is for the participant to recognize the causes of a heart attack and to differentiate between controllable and uncontrollable heart attack risk factors. The participant will also identify the lifestyle changes that can decrease the chances of having a heart attack.

Materials needed:
Other supplies

- Activity 1 “cards” (five total)

1. Introduce that there are some heart attack risk factors we cannot change and some that we can change.

    - *“The first activity will help us understand what we learned from the video about why a heart attack happens. As you saw in the video, there are many things that can cause a heart attack. Some things that can cause a heart attack cannot be changed, but there are also some things that can cause a heart attack that we can do something about.”*

2. Use the 1st card to explain the uncontrollable risk factors and how they cause a heart attack.

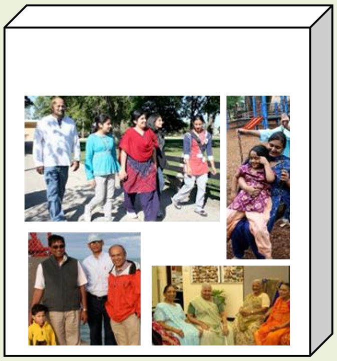

1st card

- Place the 1st card on the table so that it is standing.
- *“Some things that cause a heart attack that we cannot change are family history of a heart attack, being South Asian and old age. We learned from the video that Asian Indians and Pakistanis are more likely to have heart attacks than people from other communities. We are not exactly sure why some people have heart attacks while others don’t. But as you learned from the video, a heart attack does not have to happen and there are many things we can do to prevent a heart attack. In fact, 80% of heart disease can be prevented. We will discuss how we can prevent a heart attack in a little bit.”*

3. Use the 2nd card to explain diabetes, high cholesterol, and high blood pressure as controllable risk factors.

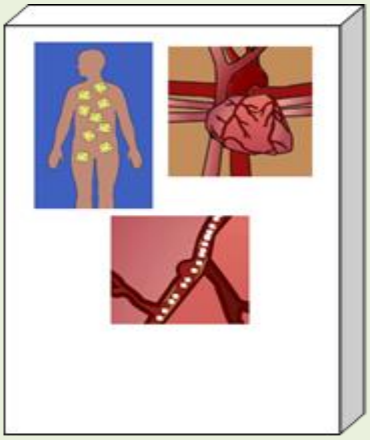

2nd card

- *“Can someone tell me what the video said that we should ask our doctor to check us for in order to prevent a heart attack?”* Answers should include: high cholesterol, diabetes, and high blood pressure.
- Place the 2nd card on the table so that it is standing behind the 1st card.
- *“Right, because having diabetes, high cholesterol, or high blood pressure can cause a heart attack. We will learn that these are things we can control.”*

4. Use the 3rd card to explain how diabetes, high cholesterol, and high blood pressure cause a heart attack.

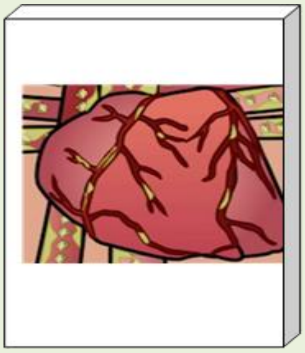

3rd card

- *"Why does having diabetes, high cholesterol, or high blood pressure increase your chances for heart disease? Think about what we learned in the video. If cholesterol, diabetes, and blood pressure are not controlled, what can happen <u>in our body (or on our heart)</u> that can cause a heart attack?"* (Answers should include: clogged arteries or blockage of arteries.)
- Place the 3rd card on the table so that it is standing behind the 2nd card.
- *"If we do not control our cholesterol, diabetes and blood pressure, this can lead to clogged arteries."*

5. Use the 4th card to explain that over time, clogged arteries cause a heart attack

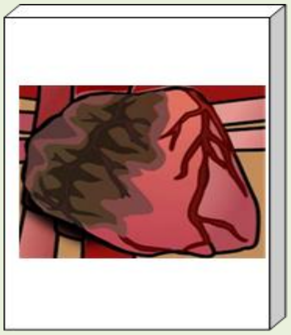

4th card

- *"What happens if our arteries are clogged?"* (Answers should include: part of the heart dies or heart attack)
- Place the 4th card on the table so that it is standing behind the 3rd card.
- *"Clogged arteries cause a heart attack. Overtime, blood vessels in the heart can get very clogged, your heart cannot get blood, and part of your heart dies. This is called a heart attack."*

6. Push the first card so that all of the cards fall to demonstrate that these risk factors can cause a heart attack.

- *"So how does a heart attack happen?"*
- Push the 1st card so that all of the cards behind it fall to demonstrate the risk factors that can cause a heart attack.

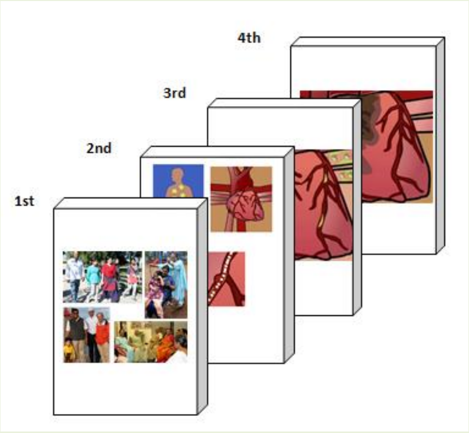

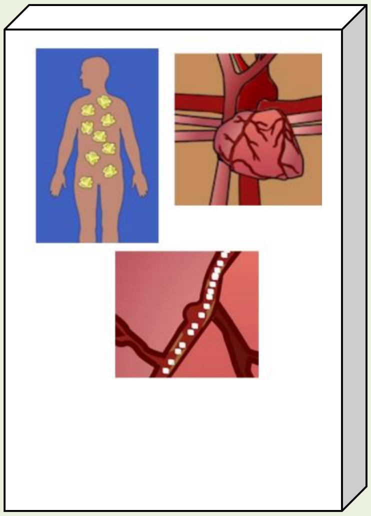

7. Have the participants identify things that they can do every day to prevent a heart attack (as seen in the video) and then show the "Ace card".

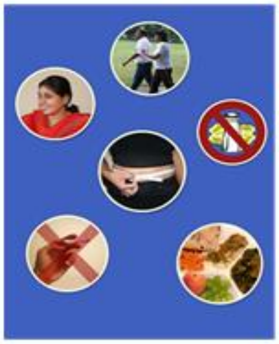

"Ace card"

- *"But there are things that we can do every day to prevent our cards from falling and causing a heart attack. What are some of these things that the video talked about?"* Answers should include: taking care of your stress, eating healthy, not using tobacco, maintaining a healthy weight, and getting more exercise
- Introduce the "Ace card"
  "Right, this is like your Ace card."

8. Explain that through healthy lifestyle changes, you can prevent the cards from falling and prevent a heart attack from happening. Demonstrate this by using the "Ace card" to stop the 1st and then 2nd card from falling and causing the other cards to fall.

   - *"If we have a family history of a heart attack, by making healthy lifestyle choices, we can help prevent a heart attack."*
     Demonstrate this by setting up the cards and putting the "Ace card" in between the 1st card and 2nd card. Push the 1st card so that the "Ace card" stops the 1st card from falling onto the other cards.
   - *"If we have high cholesterol, high blood pressure, or diabetes, we can control our diabetes and lower our cholesterol and blood pressure by making healthy lifestyle choices and by doing this we can prevent a heart attack."*
     Demonstrate this by setting up the cards and putting the "Ace card" in between the 2nd card and 3rd card. Push the 1st card so that the "Ace card" stops the 2nd card from falling on the other cards.

9. Pass the cards around to have the participants see the photos on the card and to take some time to have them think about the demonstration. Summarize as the cards are being passed around.
   - *"Family history, being South Asian and age can increase our chances of having a heart attack. Having diabetes, high cholesterol and high blood pressure can also increase our chances of having a heart attack because it causes the arteries to get clogged slowly over time and clogged arteries cause a heart attack."*
   - *"Think about how you can stop your cards from falling."*
   - *"During the next sessions, we will be learning about ways you can make small changes to improve your health and your family's health to help you stop your cards from falling and to help you prevent a heart attack."*
   - *"Does anyone have any questions?"*

Watch video: "3 things to talk to your doctor about" 8 minutes

Video Objective: After watching the "3 Things to talk to your doctor about" video, the participant will be able to state what tests they should have checked by a doctor to help prevent heart disease, identify why it is important to have diabetes, cholesterol and blood pressure checked and understand that they should get checked even if they do not feel any symptoms.

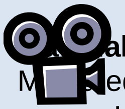

[illegible]als needed:

M[illegible] equipment

- Laptop
- Projector
- Speakers

Media

- "3 things to talk to your doctor about" video

1. Introduce the video

    - *"Our next video is going to talk about getting cholesterol, blood pressure and blood sugar checked. After the video, we will discuss what we learned and we will answer any questions about the video."*

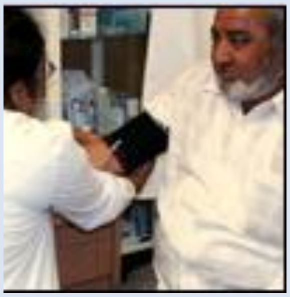

2. After the video, ask participants to summarize the video and if they learned anything new from the video

    - *"Can someone tell me what the video talked about?"*
    - *"Did you learn anything new that you didn't know before?"*

Activity 2: Understanding your results 15 minutes

Objectives: The purpose of this activity is for the participant to recall their goal ranges of BMI, waist circumference, blood pressure, cholesterol, blood sugar and A1C.

Materials needed:
Media equipment

- Laptop
- Projector

Media

- Activity 2 Understanding your results PowerPoint

Other supplies

- Box of pens
- Participants' folders with business cards, lab results, and small notepad

1. Pass out each participant's own results (they should have already received their results a week after the baseline visit).

    - "*A week after your baseline visit, you each received results from the tests given. We have a copy of those results and will pass out your results so you can follow along as Dr. Kandula will be going through what each test means and your goal number. It is important for you to know your goal number to help you prevent a heart attack. She will be available after the class to discuss results individually if you have any more questions.*"

2. Using the Activity 2 Understanding your results PowerPoint, Dr. Kandula will go over their goal BMI, waist circumference, blood pressure, cholesterol, blood sugar, and A1C emphasizing that although they may not reach this goal number, small changes towards the right direction can really help improve their health and that everyone's goal is going to depend on where they are starting. Dr. Kandula will also briefly introduce prediabetes. (See the next page for the sample test results used in the PowerPoint)

3. Let the participants know the importance of discussing their lab results with their health care provider. It will be up to the participant to discuss their lab results in detail with their health care provider and seek appropriate care if needed but we are available to help them in areas like calling the doctor's office to set up an appointment or finding a clinic that provides free or care on a sliding fee scale.

    - *"We want to make sure that everyone is seeing a health care provider or plans to see a health care provider, especially if their results are not at their goal. Please let us know at the end of the class if you have reached out to a health care provider. If you need help in setting up an appointment with a health care provider, we can assist you with that. Again we can discuss this at the end of the class."*

The South Asian Heart Disease Prevention Study

Physical Exam and Blood test Results

Date: ID # XXXX/ABCDE

Dear Mrs. XXXXABCD

We appreciate your participation in The South Asian Heart Lifestyle Intervention Study. The purpose of this letter is to provide you with some of the results of your examination.

HEIGHT: **5 feet 2 inches**

WEIGHT: **172 lbs**

BODY MASS INDEX: **31.4 kg/m^2**
A body mass index of 25 kg/m^2 or greater is considered overweight, and 30 kg/m^2 or greater is considered obese by the National Institutes of Health.

WAIST: 35 inches
Normal waist measurement for men < 35 inches
Normal waist measurement for women < 31 inches

BLOOD PRESSURE: **150/77 mmHg**

These values indicate that your blood pressure is:
□ Normal (SBP < 120 and DBP < 80)
□ Borderline Elevated (SBP 120-139 or DBP 80-90)
□ Elevated (SBP > 140 or DBP > 90)

BLOOD TEST RESULTS: (See Quest lab results enclosed)

Some of your blood test results that were out of normal range were

1. HDL cholesterol
2. [illegible]

Test Name	In Range	Out Of Range	Reference Range	Lab
LIPID PANEL				
CHOLESTEROL, TOTAL	199		125-200 mg/dL	CB
HDL CHOLESTEROL	55		> OR = 46 mg/dL	CB
TRIGLYCERIDES	136		<150 mg/dL	CB
LDL-CHOLESTEROL	117		<130 mg/dL (calc)	CB

Desirable range <100 mg/dL for patients with CHD or diabetes and <70 mg/dL for diabetic patients with known heart disease.

Test Name	In Range	Out Of Range	Reference Range	Lab
GLUCOSE	84		65-99 mg/dL<br>Fasting reference interval	CB
HEMOGLOBIN A1c		5.8 H	<5.7 % of total Hgb	

Increased risk of diabetes
<5.7 Decreased risk of diabetes
5.7-6.0 Increased risk of diabetes
6.1-6.4 Higher risk of diabetes
> or = 6.5 Consistent with diabetes

Standards of Medical Care in Diabetes-2010.
Diabetes Care, 33(Supp 1): S1-S61,2010.

Activity 3: Checking Cholesterol Scenario **5 minutes**

Objectives: The purpose of this activity is for the participant to recognize that a person should get checked for cholesterol even though they do not feel any symptoms and even if they are a vegetarian.

Materials needed:
Media equipment
- Laptop
- Projector

Media
- Activity 3 Checking Cholesterol Scenario PowerPoint

1. Use Activity 3 Checking Cholesterol Scenario PowerPoint to introduce Laxmi

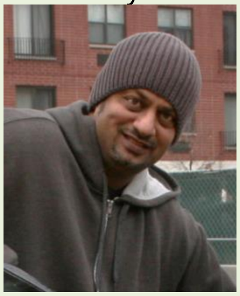

- *"Now let's talk about why it is important for you to get checked by your doctor.*
- *"This is Sunil. He is 40 years old and he feels healthy. He has always been a vegetarian.*
- *"Does he need to get his cholesterol checked?"*

2. Summarize their responses emphasizing that we may not feel any symptoms if we have high cholesterol and that even vegetarians can have high cholesterol.

   - "*Yes because even though he feels good, his cholesterol may be high. Even though we may feel fine, we could still have high cholesterol, high blood sugar, or high blood pressure. The only way to know that we have high cholesterol is to have it checked."*
   - *"Although he is a vegetarian, it is still important to get his cholesterol checked because as we learned in the last video, our body makes cholesterol and our body sometimes can make too much cholesterol. Even people that are vegetarians sometimes eat foods that increase the cholesterol in their body. We will learn more about these types of food in the later sessions."*

Activity 4: Barriers to change 8 minutes

Objective: The purpose of this activity is for the participant to identify barriers to taking care of their health and to inform the participants that despite these things, we will learn and work together to find ways to take care of our health throughout these sessions.

Materials needed:
None

1. Introduce the participant to this activity and ask the participants about barriers to taking care of health.

    - *"There are many things that make it hard for us to take care of our health. Let's take a few minutes to talk about why it can be hard to take care of our health."*
    - *"What are some things that make it difficult for people to take care of their health?"*

    If the participants are having a hard time brainstorming ideas or do not understand the question well, use weather as an example of a barrier to exercising.
    If the pariticpants are still having a hard time coming up barriers, ask the participants what are some things that make it difficult for them to eat healthy or exercise.

2. After the participants respond to the first question, go around the room to ask the following question:
    - *"Do any of those things make it difficult for you?"*

3. Acknowledge that there are many barriers that exisit and inform the participants that these sessions will help us find ways to take care of our health despite these barriers.

    - *"We do understand that there are many things that make it hard for us to take care of our health. We want you to think about these things and as you go through the classes, we will talk and learn about ways that we can take care of our health despite these things."*

CONFIDENTIAL- Do not distribute, developed by Northwestern University

Activity 5: Putting it in Perspective **8 minutes**

Objective: The purpose of this activity is for the participant to recognize the reasons of why it is important to take care of their health and to recognize how not taking care of their health affects the important things in their life. The participant will also recognize that their health and all the important things in their life are interconnected.

Materials Needed:
Media equipment
- Laptop
- Projector

Media
- Session 1 Activity 5 Putting it in perspective PowerPoint

1. Acknowledge that there are people lead busy lives and introduce *Sunil* as an example using the PowerPoint slides.

    - *"Now Let's talk about why it is important for you to take care of your health."*
    - *"Let's look at Sunil. Sunil has a family; he has a wife and three children. He works as a taxi driver and is busy with other family and friends as he spends time at this temple."*

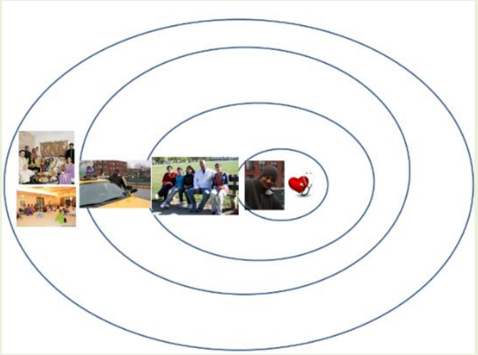

2. Ask the following questions to have the participants think about how not taking care of their health can affect the important things in their life.

    - *"How would Sunil's life be different if he were to have a heart attack?"*

3. Ask the participants if they can relate to this and use yourself as an example when explaining this model if needed. Summarize the participants' responses and conclude by acknowledging that their health and the important things in their life are interconnected.

    - *"We think that most of us spend a lot of time focusing on the outer parts of the circle. What do you think about that?"*
    - *"In this class we are going to focus on the inner circle, our health. You, your health, your family, your job, your friends and your community are all connected. Taking care of our health can help us take care of the important things in your life."*

Closing: 8 minutes

Materials needed:
Media equipment

- Laptop
- Projector

Media

- Session 1 Review PowerPoint

Handout(s) for participants

- Class evaluations

1. Pass out snacks to all the participants

2. Summarize activities done in the session using Session 1 Review PowerPoint

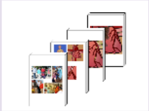

- *"This is the end of the session so let's review what we have learned today."*
- Slide 1: *"In activity one, we learned that having high blood pressure, high cholesterol and diabetes leads to clogged arteries and that clogged arteries causes a heart attack. We learned that there are things we cannot do something about and that there are things we can do something about to prevent a heart attack."*

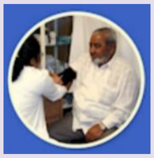

- Slide 2: *"In the second activity, Dr. Kandula explained what our goal ranges are for BMI, waist circumference, blood pressure, cholesterol, blood sugar, and A1C."*

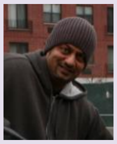

- Slide 3: *"In activity three, we talked about Laxmi and learned that it is important to get checked for high cholesterol, high blood pressure and diabetes because we can have these things even if we may feel okay and that even if we are a vegetarian, we could have high cholesterol. The only way to know is to get checked"*

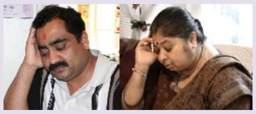

- Slide 4: *"In the fourth activity, we discussed things that make it difficult for us to take care of our health."*

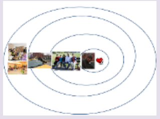

- Slide 5: *"Finally, in the last activity, we discussed that our health and the important things in our life like our families and our jobs are all connected and that by taking care of our health, it can help us do the things that are important to us."*

3. Briefly introduce next session and anything they need to prepare for next session

   - *"At the next session we will be talking about physical activity. Please wear comfortable clothes and gym shoes at our next session as we will be doing some exercise. Please make sure to bring your folders with you to every session."*

4. Hand out class evaluations to the participants and discuss questions that can be answered next session.

   - *"Before we leave, we would like for everyone to fill out this class evaluation. This will allow MAFS and Northwestern University help improve these classes."*
   - *"Also we would like for everyone to write down a question on the notepad in your folder. The questions should be about preventing heart attacks and staying healthy. For example, last time someone had heard that people in India having low cholesterol because of the warm temperature and she wanted to know more about this.* ***Please do not include any personal health issues****. We will be doing this at the end of each session because we want to make sure that everyone's questions get answered. We will collect this along with the evaluation you fill out and we will answer the questions before we start the next session."*

5. Thank the participants for attending and confirm date for next session.

   - *"Thank you for coming and we will see you next* __(day of the week)__ *at* __(time)__."

6. Ask participants if they have any questions

   - *"Remember we are available if you have any questions about the class and about your test results. Does anyone have any questions?"*

Session 2: Get More Exercise

Purpose:

Participants will learn the benefits of exercise, amount of exercise recommended, and different ways to incorporate physical activity into their lives. Participants will also do cardio and strength resistance exercises.

Session length:

70 minutes

Notes:

Collect session 1 evaluations, if any, as the participants are entering the class. Make sure to put masking tape lines on the ground before the class starts so the participants know where to stand and can do exercises for Activity 2. Men and women will separate during the exercise activities (Activity 2, 3 and 4). The activities that have been modified for the male participants are in blue.

Overview of materials needed for the session:

Media equipment
- *Video recorder
- *Audio recorder(s)
- Laptop
- Projector
- Speakers

Media
- “How to Get More Exercise” video
- Music: Dance pe chance song and other songs
- Session 1 Review PowerPoint
- Session 2 Review PowerPoint

Other supplies
- Box of pens
- Pitcher of water
- Plastic cups
- Stopwatch
- Resistance bands (one of each color for each participant)
- Step stools
- Soccer balls
- Pedometers (one for each participant)
- Masking tape (already on the ground for each participant)

Handout(s) for participants:
- Heart Rate During Exercise (Appendix 2A)
- Resistance Bands Workouts (Appendix 2B)
- My Current Daily Steps (Appendix 2C)
- Class evaluations

CONFIDENTIAL- Do not distribute, developed by Northwestern University

<u>*Remember to start the video and audio recordings.</u> The video recorder should record one of the educators as they teach the session and an audio recorder should be used for each group.

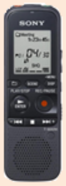
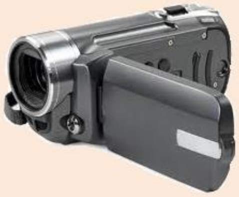

<u>Introduction: 5 minutes</u>

Materials needed:
Media equipment

- Laptop
- Projector

Media

- Session 2 Review PowerPoint

1. Review last session using Session 1 Review PowerPoint

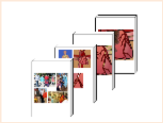

- Slide 1: *"Last session we talked about how a heart attack happens and about the things that increase our chances of having a heart attack. But we learned that there are things we can do to prevent a heart attack."*

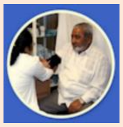

- Slide 2: *"Then Dr. Kandula explained what our results."*
- Slide 3: *"We also talked about Laxmi and learned that it is important to get checked because even though we may feel ok and even though we may be vegetarians, we can still have high cholesterol, high blood pressure, and diabetes."*

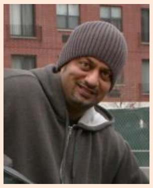

- Slide 4: *"We discussed the things that make it difficult for us to take care of our health"*

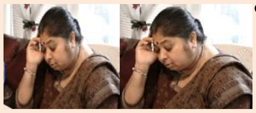

- Slide 5: *"And we also discussed that our health, our families and our jobs are all connected because taking care of our health can help us do the things that are important for us."*

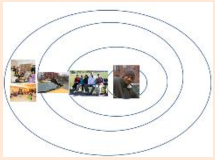

Introduction (continued): **5 minutes**

2. Go through the responses to the questions asked last week

- *"At the end of last class, we asked everyone to write down any questions that they had from that session. We will briefly review the responses to these questions."*

3. Introduce the current session topic

- *"In this session we are going to talk about getting more exercise. Everything that we learn today will help improve our health."*

Watch video: "How to Get More Exercise" **8 minutes**

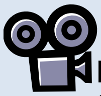

Video Objectives: After watching the "How to Get More Exercise" video, the participant will be able to describe the benefits of exercise, state how much exercise is recommended, and explain ways to incorporate exercise in their daily routine.

Materials needed:
Media equipment
- Laptop
- Projector
- Speakers

Media
- "How to get more exercise" video

1. Introduce the video

   - *"Our video today is going to talk about getting more exercise and ways to incorporate exercise in you daily routine. After the video, we will briefly discuss what we learned and then do activities that will help us better understand the information from the video. "*

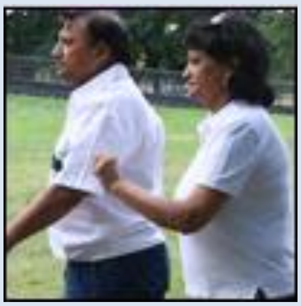

2. After the video, ask the participants to summarize the video and if they learned anything new from the video.

   - *"Can someone tell me what the video talked about?"*
   - *"Did you learn anything new that you didn't know before?"*

Activity 1: Determining Heart Rate 10 minutes

Objectives: The purpose of this activity is for the participant to recognize that heart rate will increase during exercise and to practice finding their pulse to determine if they are exercising at medium or fast speed.

Materials Needed:

Other supplies
- Box of pens
- Stopwatch

Handout(s) for participants
- Heart Rate During Exercise (Appendix 2A)

1. Establish that everyday chores helps keep us active but that we need to exercise 30 minutes a day at least five times a week in order to benefit our heart.
    - *"Doing our daily work and prayers keeps us active but to stay healthy and prevent a heart attack, we need more exercise. Can someone tell me how much exercise we need to protect our heart?*
      (Answers should include: 30 minutes a day, 5 times a week)
2. Introduce the concept that moderate to intense physical activity is most beneficial and address South Asians concern of an increasing heart rate during exercise.
    - *"That's right. It is recommended to do about 30 minutes of exercise a day for five times a week. The video mentioned we should also do exercise at a medium or fast speed. What did the video say that exercise at a medium or fast speed should feel like?"*
      (Answers should include: Your heart starts beating faster, you start to breathe a little harder, and you start to sweat lightly)
    - *"That's right. When we exercise at a medium or fast speed, our heart will start to beat faster, we will start to breathe a little harder and we may start to sweat lightly. Some of us may be scared to exercise because our hearts start to beat faster but we want you to not be startled because it is normal for your heart to beat faster when doing exercises. If you are just starting to exercise or haven't exercised before, it is important to start slowly because our body is not used to exercise. But overtime, your body will get used to exercise."*
3. Express that by determining our heart rate through finding our pulse we can make sure we are exercising at moderate intensity without overdoing it and define heart rate.
    - "*We want to make sure you are doing exercise at medium or fast speed that will benefit your heart. We are going to learn a simple way to make sure your heart is beating faster without beating too fast."*
    - *"In order to make sure we are doing enough exercise but not too much, we will learn what our heart rate should be when we exercise. Our heart rate is the number of times our heart beats per minute. We can know our heart rate by checking our pulse."*

4. Demonstrate how to properly find their pulse

- *“First I will show you how to find your pulse, and then we will do it together. Place two fingers on the thumb side of your wrist. Use the tips of your first two fingers (not your thumb) to press lightly over the blood vessels on your wrist. You should feel your heart beat.”*

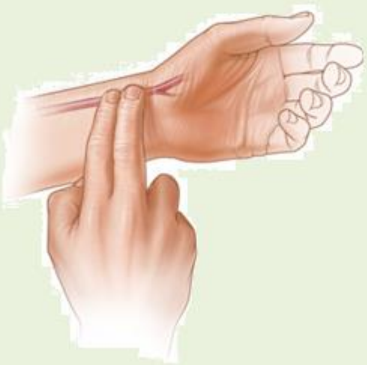

5. Have the participants find their pul [illegible] participants can find their pulse.

- *“Now let’s all practice finding our heart rate. We can help you check your pulse if you need help.”*

6. Time the participants for 30 seconds and have them count their own pulse within that 30 seconds. Multiply by 2 to find the beats per minute.

- *With the stopwatch I will time 30 seconds. When I say go, start counting your heart beat and when I say stop, stop and remember your number.”*

7. Ask each participant how many heart beats they counted and their heart rate. Explain that this is their heart rate at rest and everyone’s resting heart rate will be slightly different.

- *“We will go around the room and say how many beats they counted so we can determine their heart rate. This is our heart rate at rest.”*
- *“Everyone’s pulse at rest will be slightly different, about 60-100 beats per minute.”*
- *“Now I will pass out a chart so that you can see what your pulse should be when you exercise.”*

8. Pass out the Heart Rate During Exercise handout (Appendix 2A) and pens and have the participants find their target heart rate zone during exercise.

- *“On the paper, find your age. For your age, circle your target heart rate zone during exercise with a pen.”*
- *“We will be checking our pulse while we do exercise to see what our hear rate is. Our heart rate during exercise should fall between these numbers.”*
- *“If your pulse is above our zone, you need to slow down. If it is below your zone, then you can go a little faster. During the first weeks of working out, aim for the lowest part of your zone. Then, gradually build up to the higher part. After sometime, you may be able to exercise comfortably at the higher part of your zone.”*

Activity 2: Cardio exercises 10 minutes

Objectives: The purpose of this activity is for the participant to perform physical activity and to check their heart rate by finding their pulse.

Materials Needed:

Media equipment
- Laptop
- Projector
- Speakers

Media
- Dance pe Chance song

Other supplies
- Pitcher of water
- Plastic cups
- Stop watch
- Masking tape (already on the ground for each participant)

1. Before leading 4-minute warm up stepping over the tape line on the ground with music. let the participants know that they can drink water if needed and to do their best in following along.

    - *"Now we will be doing some exercise. We have water on the side so if you need to you can get a drink. It is ok to have some water while you exercise or after you exercise. The important thing to remember is to not drink too much water or too not drink too fast."*
    - *We will be stepping over the tape that is on the ground. Try to step over the line; back and forth. We will start slow and then go to a medium and then fast speed. If it is too much for your knees, move your arms. This will help keep your heart beating. Do the best you can to follow me as we go with music."*

2. Lead the 4-minute dancing workout with music.

    - *"Now we will be doing another exercise. I will be calling out the steps so do the best you can to follow along. If you get lost or can't follow along, just move back and forth. The important thing is to keep moving. Remember to breathe and have fun!"*

3. After exercising, use the stop watch to time the participants as they check their pulse for 30 seconds to determine if they are in their target heart rate zone.

4. Ask the participants how they will feel during exercise and summarize.

    - *"How do you feel now compared to before exercising?"*
    - *"Right, remember, when we exercise, it is normal for our hearts to beat faster and for us to breathe a little harder. But checking your pulse will help you make sure you are doing enough exercise to benefit your heart."*

Activity 2: Cardio exercises 10 minutes

Objectives: The purpose of this activity is for the participant to perform physical activity and to check their heart rate by finding their pulse.

Materials Needed:

Other supplies

- Pitcher of water
- Plastic cups
- Stop watch
- Step stools (one for each participant)
- Masking tape (already on the ground for each participant)

1. Before leading the 4-minute warm up stepping over the tape line on the ground, let the participants know that they can drink water if needed and to do their best in following along.

    - *"Now we will be doing some exercise. We have water on the side so if you need to you can get a drink. It is ok to have some water while you exercise or after you exercise. It is important thing to remember is to not drink too much water or too not drink too fast."*
    - *We will be stepping over the line on the ground back and forth. We will start slow and then go to a medium and then fast speed. If it is too much for your knees, move your arms. This will help keep your heart beating. Do the best you can to follow me as we go."*

2. Lead 4-minute cardio workout

    - *"Now we will be doing another exercise. "Do the best you can to follow along. The important thing is to start slow and keep moving. Remember to breathe"*

<u>Exercise</u>

Dynamic stretching: arms across body, arm circles and march in place
20-30 seconds – squat punching left and right
10 jumping jacks
20-30 seconds – squat punching left and right
10 high knees
20-30 seconds – squat punching left and right
10 butt kicks
20-30 seconds – squat punching left and right
10 "basketball throws" from the right
20-30 seconds – squat punching left and right
10 "basketball throws" from the left
Repeat sequence starting with 20-30 seconds – squat punching left and right

3. After exercising, use the stop watch to time the participants as they check their pulse for 30 seconds to determine if they are in their target heart rate zone.

4. Ask the participants how they will feel during exercise and summarize.

    - *"How do you feel now compared to before exercising?"*
    - *"Right, remember, when we exercise, it is normal for our hearts to beat faster and for us to breathe a little harder. But checking your pulse will help you make sure you are doing enough exercise to benefit your heart."*

Activity 3: Strength resistance exercises 10 minutes

Objectives: The purpose of this activity is for the participant to recognize the benefits of strength exercises and to perform strength exercises using resistance bands.
Materials Needed:

Other supplies
- Pitcher of water
- Plastic cups
- Step stools
- Soccer balls
- Resistance bands (one of each color for each participant)

Handout(s) for participants:
- Resistance Bands Workouts (Appendix 2B)

1. Introduce strength exercises

    - *"During strength exercise, your heart and breathing may not be as fast compared to the exercises we just did for our heart but this type of exercise is important too. Strength exercises are both safe and helpful for women and men of all ages, even for people with heart disease or arthritis."*

2. Emphasize benefits of strength exercises

    - *"Doing strength exercises helps in strengthening bones and building muscles. Having more muscles burns energy and helps improve blood sugar. When we work our muscles, more sugar is taken up from the muscle and this improves our blood sugar because then there isn't so much sugar in the blood. Exercising also helps increase your HDL or good cholesterol."*

3. Pass out the resistance bands while explaining how often to do strength exercises and how to use the resistance bands

    - *"It is recommended to do strength exercises twice a week. Start out with a weight that you can do only 8 times. Continue until you can do it 10 to 15 times. When you can do 2 sets of 10 to 15 repetitions easily, add more weight so that, again, you can lift it only 8 times. Keep repeating until you reach your goal, and then maintain that level as long as you can. The important thing is to start slow and gradually increase."*
    - *"It is important to work all muscle groups. We will do strength exercises that will work six muscle groups."*
    - *"You can either hold on to your bands or wrap it around your hand. If you hold it, make sure to hold on to it well so it doesn't snap and hurt you. If you wrap it around your hand, make sure to wrap it around your hand once so that it is comfortable and not too tight on your hand.*
    - *"To prevent injury, use smooth and steady movements. Do the exercises in a slow, controlled manner and remember to breathe regularly."*

4. Demonstrate each band exercise and have the participants do five repetitions of each exercise (eight-10 repetitions for men). For each exercise, describe what major muscle group is being worked.

Exercise Bands workout

- *“This exercise works the shoulders. We will do this five times (eight times). If it is too much, you can use one arm at a time.”*
- Lateral Raise – Exhale as you raise your arms and inhale as you bring your arms down to your sides.

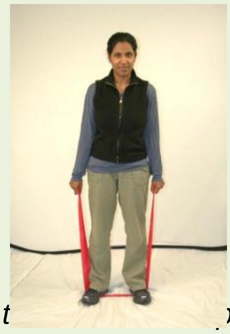
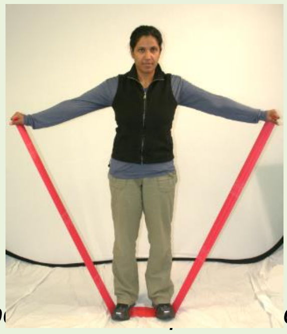

- *“This exercise works t[illegible]pp[illegible]ows in place as you bring the band towards you. We will do this five times (eight times). If it is too much, you can lift one arm at a time.”*
- Elbow Flexion/Bicep Curl –Exhale as you bring your forearms towards you and inhale as you bring your arms down.

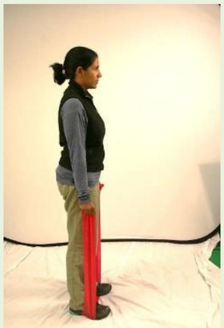
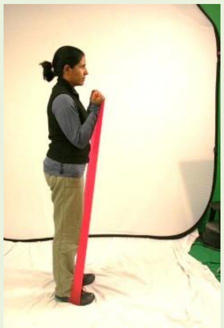

- *“This exercise works the sides of the midsection. Lean away from where the weight is. We will do this five times (eight times) on each side.”*
- Standing Oblique Crunch – The arms should remain on the side and the knees shouldn’t bend. The arm on the side you are leaning on should reach your knee. Exhale as you lean towards the side and inhale as you come up towards the middle.

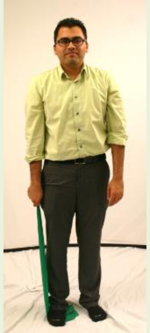
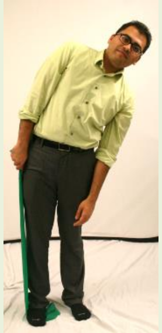

- *“This works the front of the upper legs. Squat as if you are about to sit on a chair.” We will do this five times (eight times).*
- Squats – Try not to let your knees pass your toes. Inhale as you go down and exhale as you come up.

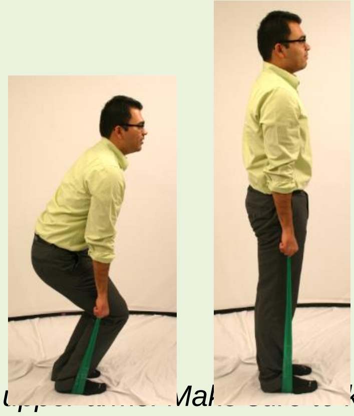

- *“This works the back of your [illegible] Ma[illegible]keep your elbow in place. We will do this five times (eight times) on each side.”*
- Overhead Triceps Extension – Exhale as you pull your arm up and inhale as you bring your arm down.

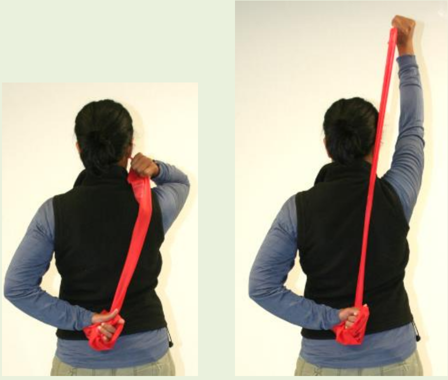

- *“This works the upper back. We will do this five times (eight times) on each side.”*
- Reverse Fly – this works the upper back. Exhale as you pull your arms out and inhale as you pull your arms in.

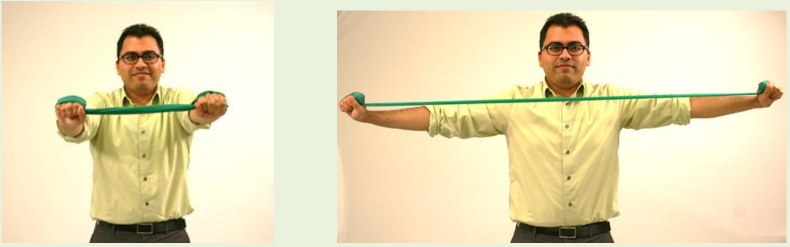

5. Demonstrate and have the participants do strength exercises they can do at home that can be done without weights or resistance bands.

    - *"If you don't have weights or your band with you, here are some other ways to do strength exercises":*
        - *Sitting squats having butt barely touch a chair*
        - *Stepping up and back down on one leg using small stool or stairs*
        - *Throwing a ball to a partner at chest level or overhead*

6. Introduce the importance of cooling down and stretching and perform stretches with the participants

    - *"It is important to stretch or cool down after you exercise because it can improve your ability to exercise and help prevent injury."*
    - *"We are going to do two basic stretches. For each stretch we will hold it about 10-20 seconds."*

Stretches

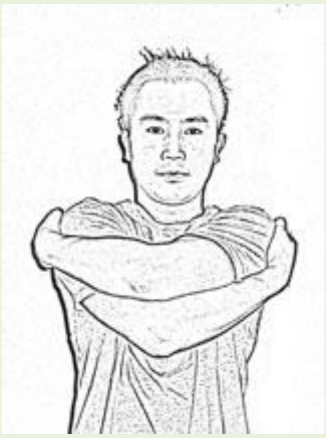

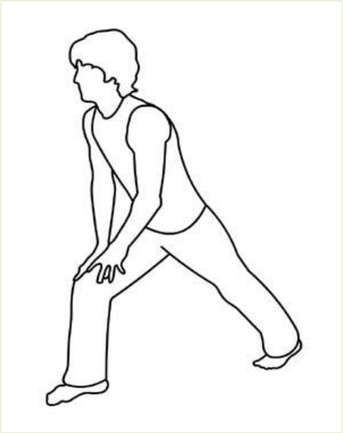

7. Pass out the Resistance Bands Workouts handouts (Appendix 2B)

Activity 4: Using a Pedometer 15 minutes

Objective: The purpose of this activity is for the participant to correctly practice using a pedometer as instructed.

Materials Needed:

Other supplies
- Pedometers (one for each participant)

Handout(s) for participants:
- My Current Daily Steps (Appendix 2C)

1. Introduce and distribute pedometers to everyone in the group.

   - *"To help you keep track of how much you walk, we will be giving everyone a pedometer. A pedometer counts how many steps you make when you are doing things such as climbing stairs, dancing, household chores, and walking. However, pedometers cannot be used in water."*

2. Familiarize everyone with the pedometer use.
   - How to open to check steps
   - How to place on clothing
     - Secure the pedometer snug against your waist at hip level; aligned directly over your knee. (If the participant has a tummy you may need to position differently at the side of the waistline in line with arm may be best.)
     - Held firmly to the body
     - Should not be tilted forward or backward, or tilted to the side
     - Should be clipped on innermost clothing

3. Have all the participants put the pedometers on and check the placement of all pedometers. Make sure the pedometers are at zero before they take any steps.

4. Have all the participants walk around the room for at least 20 steps and check to see if the pedometer reads between 18-22 steps. If not, reposition it on the belt or waistband and have them try the test again.

5. Pass out the My Current Daily Steps handout (Appendix 2C) to the participants. Have the participants record their daily steps and bring the worksheet with them at the next session.

   - *"We would like for you to record your steps and bring them in next class. We will talk about this next session."*
   - *"To give you an idea 2,000 steps is about 1 mile. It takes about 20 minutes to walk regularly 1 mile or 15 minutes walking briskly. A mile is about the distance from MAFS to red line at Howard."*

CONFIDENTIAL- Do not distribute, developed by Northwestern University

Closing: 10 minutes

Materials needed:
Media equipment

- Laptop
- Projector

Media

- Session 2 Review PowerPoint

Handout(s) for participants

- Class evaluations

7. Pass out snacks to all the participants

8. Summarize activities done in the session using Session 2 Review PowerPoint and discuss safety during physical activity

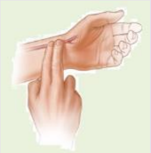

- *“This is the end of the session so let’s review what we have learned today.”*
- Slide 1: *“For our first activity we learned that when we exercise it is normal for our heart to beat faster. We learned this is healthy for our heart and that we can check our pulse to make sure our heart is beating at the right range during exercise.”*

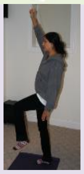

- Slide 2: *“For our second activity, we learned how to do exercises that will get our heart beating faster and we practiced feeling our pulse.”*

- Slide 3: *“In the activity three, we learned about the importance of doing strength exercises and we learned how to do some strength exercises using the exercise bands as well as some stretching after exercises.”*

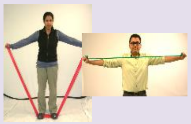

- Slide 4: *“In our last activity, we learned how to use a pedometer and how we can use this to record our steps.”*

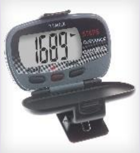

- *“We would like for everyone to try something they learned in class today. At the beginning of next class, we will talk about how it went.”*
- *“As you start to exercise, remember to wear proper shoes and to start slow and gradually increase as you get used to it. We don’t want anyone to get injured or hurt.”*

9. Briefly introduce next session and anything they need to prepare for next session

- *“At the next session we will be talking about whole grains, fruits and vegetables.”*
- *“Please remember to wear you pedometer, to record your steps in the worksheet and to bring it in to the next class. We will go over them briefly before we start our next session.”*

10. Hand out class evaluations to the participants and discuss questions that can be answered next session.

    - *"Before we leave, we would like for everyone to fill out this class evaluation. This will allow MAFS and Northwestern University help improve these classes."*
    - *"Also we would like for everyone to write down a question on the back of the class evaluation. The questions should be about this session.* ***Please do not include any personal health issues****. We will be doing this at the end of each session because we want to make sure that everyone's questions get answered. We will collect this when you are finished and we will answer the questions before we start the next session."*

11. Thank the participants for attending and confirm date for next session.

    - *"Thank you for coming and we will see you next* __(day of the week)__ *at* __(time)__.*"*

12. Ask participants if they have any questions

    - *"Does anyone have any questions?"*

During Exercise

Age	Target Heart Rate
**20**	100-170 beats per minute
**30**	95-162 beats per minute
**35**	93-157 beats per minute
**40**	90-153 beats per minute
**45**	88-149 beats per minute
**50**	85-145 beats per minute
**55**	83-140 beats per minute
**60**	80-136 beats per minute
**65**	78-132 beats per minute

Appendix 2A (Heart Rate During Exercise, page1 of 1)
*Taken from the American Heart Association
http://www.heart.org/HEARTORG/GettingHealthy/PhysicalActivity/Target-Heart-Rates_UCM_434341_Article.jsp

Appendix 2B (Resistance Bands Workout, page 1 of 2)

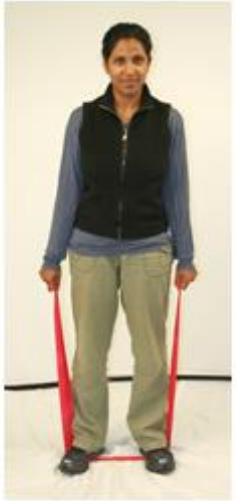

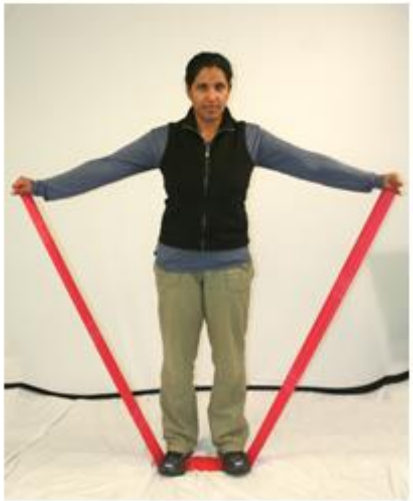

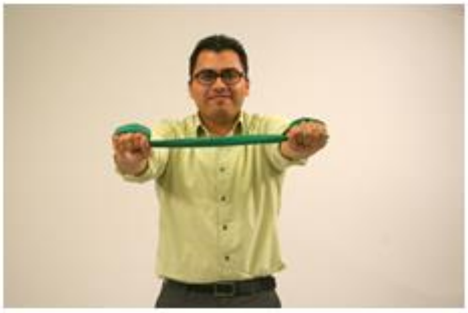

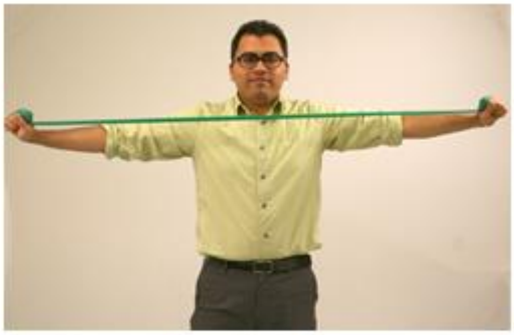

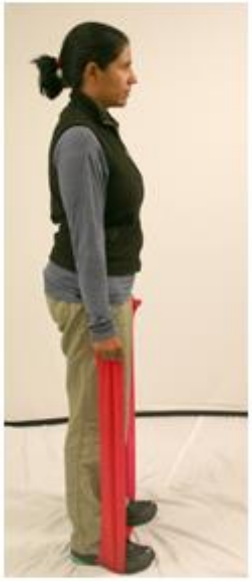

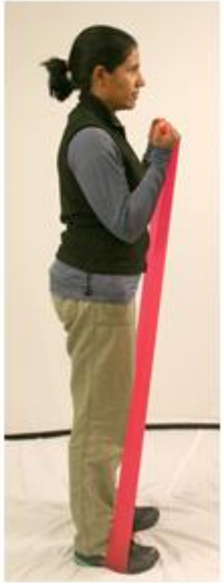

Appendix 2B (Resistance Bands Workout, page 2 of 2)

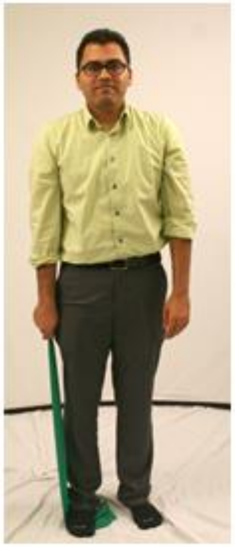

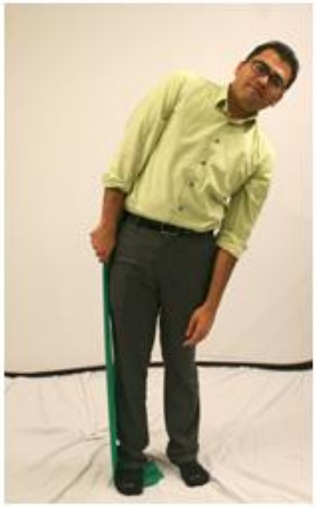

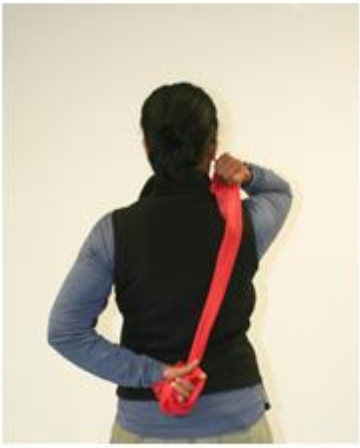

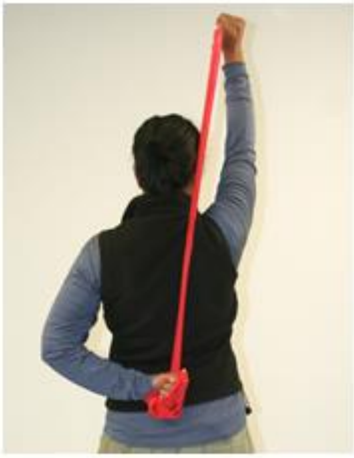

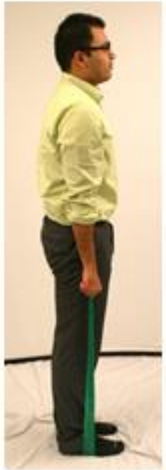

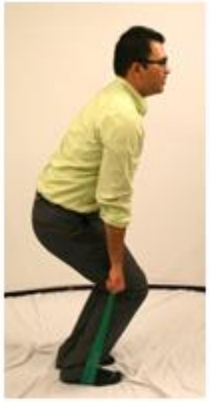

My Current Daily Steps

Day of the Week	Number of Steps I took
Sunday	
Monday	
Tuesday	
Wednesday	
Thursday	
Friday	
Saturday	

Session 3: Enjoy Fruits, Vegetables & Whole Grains 70 min

Learning Objectives:
At the end of this session, participants will be able to:

- Describe ways to eat more fruits, vegetables and whole grains
- Differentiate whole grains from refined grains
- Recognize the health benefits of whole grains
- Identify whole grains
- Recognize the recommended servings of fruits and vegetables per day
- Identify a serving of fruits and vegetables
- Recognize best methods to cook vegetables
- Identify disadvantages to overcooking and frying vegetables
- Create a tasty and healthy salad

Notes:

- Make sure that the whole grain dish and salad station is prepared ahead of time

Overview of materials:
Media equipment

- Laptop
- Projector
- Speakers

Media

- *Enjoy Fruits, Vegetables and Whole Grains* video
- Session 3: Enjoy Fruits, Vegetables and Whole Grains PowerPoint (see Appendix 3A for PowerPoint Slides)

Other Supplies

- Box of pens
- Whole grain model
- Disposable bowls and silverware for the participants
- Whole grain dish (see Appendix 3B for recipe)
- South Asian salad ingredients (see Appendix 3C for recipe)
- 12 baby carrots in a sandwich bag
- 2 1-cup spinach in a sandwich bag
- 1 medium apple
- ½ cup of raisins in a sandwich bag
- ½ cup of okra food model
- ½ cup of cauliflower food model
- 1 cup of green beans food model

Handouts for participants:

- Appendix 3D: My Steps
- Class Evaluations

Review of the Previous Session

Materials needed:

Media equipment

- Laptop
- Projector

Media

- Session 3: Enjoy Fruits, Vegetables and Whole Grains PowerPoint (see Appendix 3A for PowerPoint Slides)

Show the Session 3: Enjoy Fruits, Vegetables and Whole Grains PowerPoint:

> Slide 1: *"Last week, we learned that when we exercise, it is normal for our heart to beat faster We learned this is healthy for our heart and that we can check our pulse to make sure our heart is beating at the right range during exercise."*
>
> Slide 2: *"We then did some exercises that increase our heart rate and we practices feeling our pulse."*
>
> Slide 3: *"We also learned about the importance of doing strength exercises and we learned how to do some strength exercises using the exercise bands and we also stretched after exercising."*
>
> Slide 4: "*Finally, we learned how to use a pedometer and learned how we can use the pedometer to record our steps."*

Check-In with Progress

Materials needed:

Handouts for participants:

- Appendix 3D: My Steps

Ask the participants if they tried anything new from the last class. Facilitate a brief discussion on barriers to trying something new and encourage the participants to problem solve and brainstorm other ways they can get more exercise. Encourage the participants to try what they learned in class or to try other ways to get more exercise.

Review the participants' steps recorded. Provide affirmations to the participants that increased their steps. Take the time to have the participants share with one another the facilitators and barriers to increasing their steps.

Ask the participants:

> *"Why do you think we had you record your steps?"*

Summarize the participants' responses:

> *"Recording your steps is a way for you to become aware of your activity and to see if you can improve the amount of exercise you do. If you know where you are at with exercise, you have a better idea of what exercise goal you can set."*

Discuss goal setting with the pedometer:

"Last session we learned that 2,000 steps is about one mile. Doctors and fitness experts recommend walking 10,000 steps which is about five miles every day as a way to maintain a healthy weight and exercise your heart. If you are trying to lose weight, 12,000 – 15,000 steps a day is recommended."

"It is important to slowly increase your steps to your goal number. Continue to record your steps so you can see how you are doing with exercise and so you can see your progress. Please fill out your step log and bring it to each class so we can look at it together in the beginning of each class."

Review Questions Asked

Materials needed:
None

Go through the responses to the questions asked last week, if any:

"At the end of last class, we asked everyone to write down any questions that they had from that session. We will briefly review the responses to these questions."

Introduce New Session

Materials needed:
None

"In this session we are going to talk about the importance of eating fruits, vegetables and whole grains and we will learn about ways that we can enjoy fruits, vegetables and whole grains. Everything that we learn today will help improve our health."

"We will start with a video and briefly discuss what we learned. Then we will do some activities that will help us better understand the information from the video. At the end of the class, we will review the session and you will have an opportunity to write down any other questions that you may have about today's session."

Watch Video

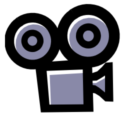

Materials needed:
Media equipment

- Laptop
- Projector
- Speakers

Media

- *Enjoy Fruits, Vegetables and Whole Grains* video

Play the Video

After playing the video, ask the participants to summarize the video and if they learned anything new from the video:

"Can someone tell me what the video talked about?"

"Did you learn anything new that you didn't know before?"

Activity 1: Why Whole Grains?

Materials Needed:
Other supplies

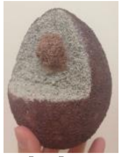

- Whole grain model

Define a whole grain and use the whole grain model to show participants the different parts of a whole grain:

"The video talked about eating whole grains like brown rice instead of white rice. Brown rice is one type of whole grain but there are different types of whole grains. We are going to learn the what a whole grain is and why it is important."

"Whole grains are grains that have the entire seed in one piece. The seed is made of three parts:

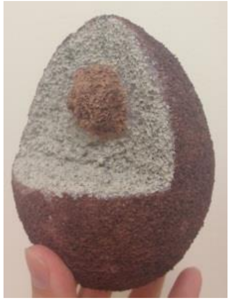

- *The seed has an outer part that protects the seed*
- *A large part that provides energy for the seed*
- *And a small part that provides nourishment for the seed. This small part is where the sprout comes from when a seed is soaked."*

Whole grain model

Ask the participants:

"White rice, which is not a whole grain, has the outer part and the small part removed. Why do you think these parts are removed?"

Summarize the participants' responses:

"These two parts have been removed so the food can last longer and doesn't go bad. These two parts have the most nutrients."

Explain the benefits of whole grains for our health:

"The smaller part of the whole grain contains many vitamins and minerals needed for our body and the outer part of the whole grains contains fiber. These two parts have a lot of nutrients that are beneficial for our heart and overall health which is why it is so important to eat whole grains."

"Eating whole grains can help:

- *Lower total cholesterol and LDL or bad cholesterol*
- *Lower blood pressure*
- *Reduce chances of certain cancers, especially colon and rectal cancers*
- *Keep you regular and prevent constipation*
- *Reduce chances od developing type 2 diabetes*
- *Lower blood sugar levels*
- *Control weight. The fiber in whole grains can help you feel fuller longer after a meal."*

Activity 2: A Closer Look at Whole Grains

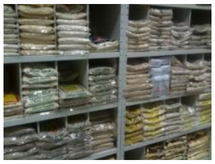

Materials needed:
Media equipment

- Laptop
- Projector

Media

- Session 3: Enjoy Fruits, Vegetables and Whole Grains PowerPoint

Other Supplies

- Disposable bowls and silverware for the participants
- Whole grain dish (see Appendix 3B for recipe)

Introduce the importance of eating a variety of whole grains:

"It's important to know that there is no 'healthiest' whole grain. Some have more of one nutrient than others. They all offer different benefits so it is best to enjoy a variety, for the best range of healthy nutrients and good tastes."

Show the Session 3: Enjoy Fruits, Vegetables and Whole Grains PowerPoint:

Slide 5: *"Brown rice is one type of whole grain. Let's look at other types of whole grains."*

Slide 6: *"This is wheat. Wheat is a grain. Sometimes wheat can be a whole grain, sometimes it is not a whole grain."*

Slide 7: *"These are wheat flours that are not made from whole wheat. The outer part and the smaller part are removed."*

Slide 8: *"These are wheat flours that are whole grain. We are just showing you different brands, but one brand is not better than another. To be certain that you buy whole grain wheat, look for the words 100% whole wheat on the package."*

Slide 9: *"Jav (barley) is another whole grain."*

Slide 10: *"Bajra (pear millet) and Ragi (finger millet) are also whole grains."*

Slide 11: *"Jowar (sorghum) is also a whole grain."*

Slide 12: *"Oatmeal is another whole grain."*

Slide 13: *"Rajgaro (buckwheat) is similar to whole grains and it has the benefits of a whole grain."*

Slide 14: *"Quinoa* (pronounced KEE-NUAW) *is also similar to whole grains and it has the benefits of a whole grain. You can find quinoa at grocery stores like Jewel or Dominick's and you cook it by boiling it in water."*

Emphasize eating a variety of whole grains:

"We are showing you a variety of whole grains because it is important to try different grains as they all vary in their nutrition. Some may have more fiber than others; some may have more protein and may different in amount of vitamins and minerals. Some of these grains may be familiar while some may be new to you."

Acknowledge cost as a barrier to eating whole grains:

"We understand that some whole grains may cost more than others. We encourage you to eat a variety of whole grains. Five years ago, Patel Brothers didn't carry all these whole grain flours. Now that we know more about the benefits and now that people are starting to ask for these whole grains, the stores are making it more available. Some of these whole grains are expensive, but the more we ask, the easier it will be for us to buy it."

Taste-test already prepared whole grain recipe (see Appendix 3B for recipe)

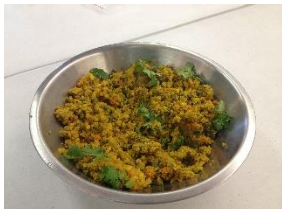

"The video mentioned some ways to eat more whole grains like eating brown rice and whole wheat chappattis. Preparing whole grains with vegetables, like this barley upma, is another way to eat more whole grains."

Quinoa dish

Activity 3: Am I eating enough fruits and vegetables?

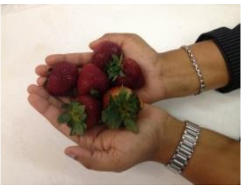

Materials Needed:
Other supplies
- 12 baby carrots in a sandwich bag
- 2 1-cup spinach in a sandwich bag
- 1 medium apple
- ½ cup of raisins in a sandwich bag
- ½ cup of okra food model
- ½ cup of cauliflower food model
- 1 cup of green beans food model

Explain the importance of fruits and vegetables and introduce the recommendations:

"Like whole grains, vegetables and fruits contain fiber and many important nutrients to keep us healthy and to protect us from heart disease. It is recommended that we eat about five or more servings of fruits and vegetables every day. That is about three servings of vegetables and two servings of fruits every day."

Demonstrate a serving of fruits and vegetables:

"In general, 1 serving of raw fruits or vegetables is about what you can hold or cover in two hands."

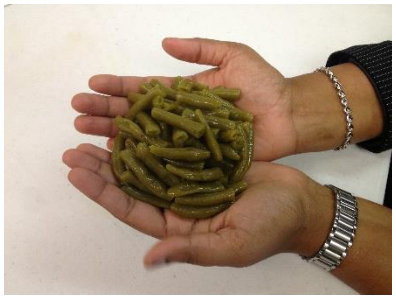

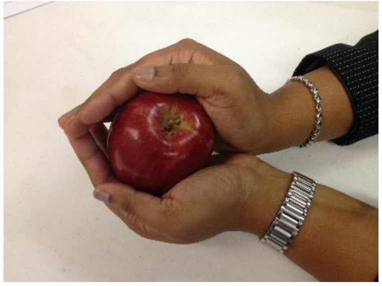

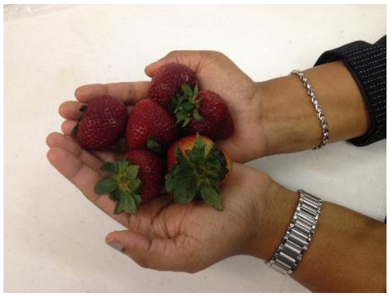

1 cup of raw green beans 1 medium size apple about 1 cup of strawberries

"For raw leafy greens like spinach, one serving size is four hands of raw leafy vegetables."

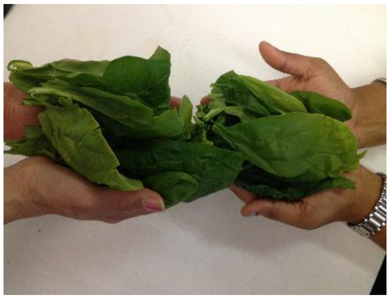

About 2 cups of spinach

"One serving of cooked vegetables is about what you can hold in one hand. For cooked leafy greens, one serving is two hands of cooked leafy greens."

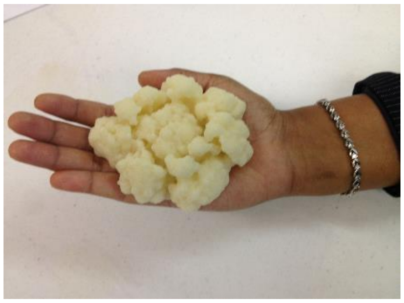

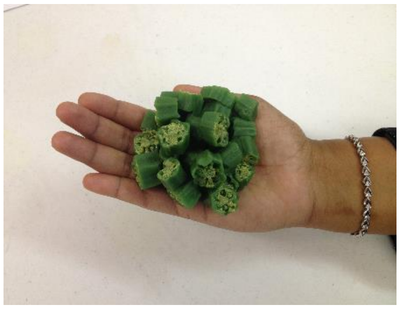

½ cup of cooked cauliflower ½ cup of cooked okra

"For dried fruit, one serving is also about how much you can fit in one hand."

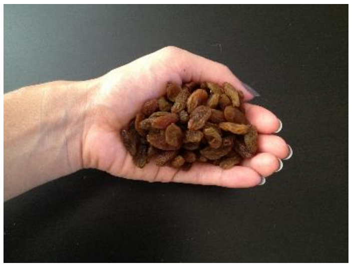

about ½ cup of raisins

Pass around the food models and the fruits and vegetables in a sandwich bag so the participants can feel how much is one serving.

Place five servings of fruits and vegetables on the table:

> *"This is an example of having five servings of fruits and vegetables in one day. Think about how you can eat this recommended amount of vegetables and fruits."*

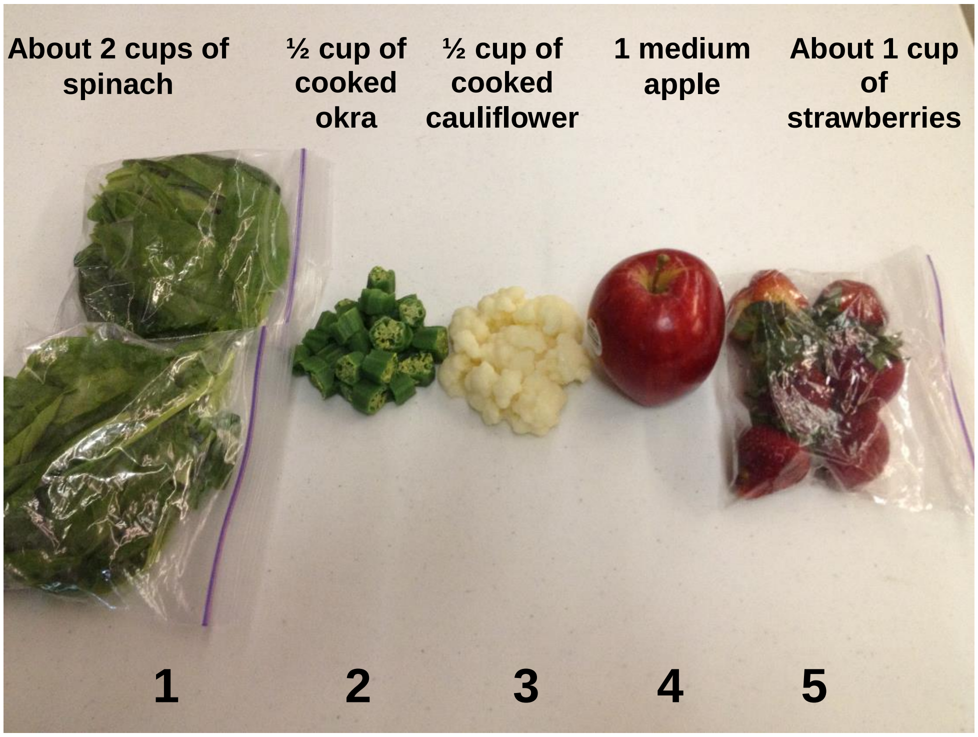

Activity 4: Preparing Vegetables

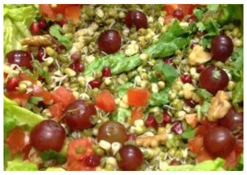

Materials needed:
Media equipment

- Laptop
- Projector

Media

- Session 3: Enjoy Fruits, Vegetables and Whole Grains PowerPoint (see Appendix 3A for PowerPoint Slides)

Other supplies

- Disposable bowls and silverware for the participants
- South Asian salad ingredients (see Appendix 3C for recipe)

"Now we are going to talk about the best ways to prepare vegetables. In order to maintain the vegetables' vitamins and minerals, the best ways to cook vegetables are to steam, pressure cook, or microwave vegetables until they are soft but not mushy. If we cook vegetables for too long we may lose important nutrients."

"There was a study done called the INTERHEART study that looked at people from different countries around the world. In the study, they saw high heart attack rates in people from South Asia. So to find out what could be causing it, they looked at all their habits like eating habits. One thing that they found was that even though South Asians are eating vegetables, we are frying them and overcooking them so we may not be benefiting from the vegetables."

Note: For more information on the INTERHEART study, visit http://www.phri.ca/interheart/.

"Frying vegetables also loses important nutrients because the temperature can get very hot and destroy the nutrients. Frying vegetables also add a lot of fat our foods and this is not good for us either. We will talk more about fat in the next session."

"In order to make sure we are getting the nutrients that we need from our vegetables, it is important to cook our vegetables for a short time until they are soft not mushy and not to fry vegetables too often. Another important way to eat vegetables is to eat them raw for example to include a salad in our meals."

Show the Session 3: Enjoy Fruits, Vegetables and Whole Grains PowerPoint:

Slide 15: *"When people think of a salad, they usually think of lettuce and tomatoes like this salad."*

Slide 16: *"But there are other ways to make salads. This is another salad."*

Slide 17: *"Which salad would you be more likely to eat? Why would you be more likely to eat this salad?"*

Slide 18: *"There are some of the salads that some of our participants prepared at home. They added a variety of vegetables and even fruits to make the salad look good and appealing to the whole family. They add herbs, spices, some oil, and lemon juice to make it tasty. Some even added dals, beans, sprouts, seeds, nuts, tofu and whole grains like barley to add more variety."*

"Now you will all make your own salad. You can add different vegetables, fruits, lemon juice, some oil, spices, herbs, nuts, and chickpeas; it is up to you!"

As the participants are preparing and eating their salads, ask the participants what they think of the salad and what other foods they could add to the salads.

Review of Current Session

Materials Needed:
Media equipment
- Laptop
- Projector

Media
- Session 3: Enjoy Fruits, Vegetables and Whole Grains PowerPoint (see Appendix 3A for PowerPoint Slides)

Show the Session 3: Enjoy Fruits, Vegetables and Whole Grains PowerPoint:

> Slide 19: *"In the first activity, we discussed fruits, vegetables and whole grains. We learned about the three parts of a whole grain and how eating whole grains can benefit our health."*
>
> Slide 20: *"In activity two, we looked at the different whole grains that we can find at Patel Brothers and other grocery stores and we even tried a whole grain dish prepared by MAFS kitchen staff."*
>
> Slide 21: *"In activity three, we learned about the recommendations to eat five servings of fruits and vegetables and learned what a serving of fruits and vegetables is."*
>
> Slide 22: *"In the last activity, we learned about the best ways to cook our vegetables and made a tasty salad."*

Encourage the participants to try something knew that they learned in today's session:

> *"We would like for you to try something you learned in class today. We will talk about it at the beginning of next class."*

Closing

Materials Needed:
Handouts for participants:
- Class Evaluations
- Appendix 3D: My Steps

Reminder for the next session:

> *"At the next session, we will be talking about how to eat less fat and salt. Please remember to continue wearing your pedometer, to record your steps in the worksheet and to bring it with you to the next class."*

Hand out Appendix 3D: My Steps and Class Evaluations (one per participant):

“Before next session, we would like for everyone to fill out this class evaluation. This will help MAFS and Northwestern University improve these classes.”

Collect Questions from this session:

“Also we would like for everyone to write down a question on the back of the class evaluation. The questions should be about this session. ***Please do not include any personal health issues****. We will be doing this at the end of each session because we want to make sure that everyone’s questions get answered. We will collect this when you are finished and we will answer the questions before we start the next session.”*

Thank the participants for attending and confirm the date and time for the next session.

Appendix 3 A:
Session 3: Enjoy Fruits, Vegetables and Whole Grains PowerPoint Slides

Slide 1
Session 2 Review (Activity 1: Determining Heart Rate)

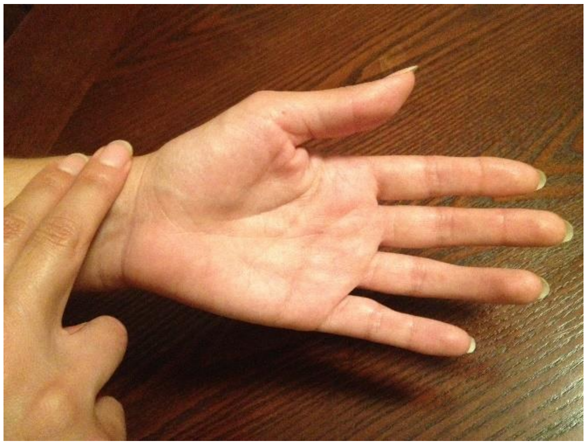

Slide 2
Session 2 Review (Activity 2: Cardio Exercises)

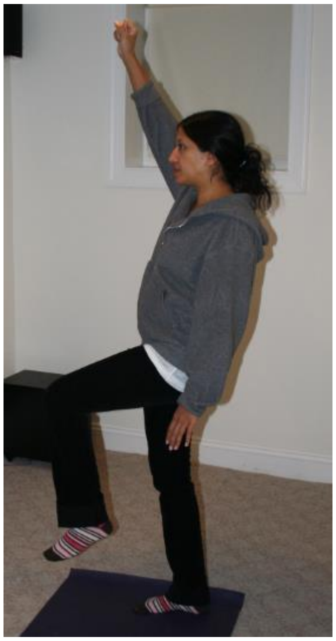

Slide 3

Session 2 Review (Activity 3: Strength Resistance Exercises)

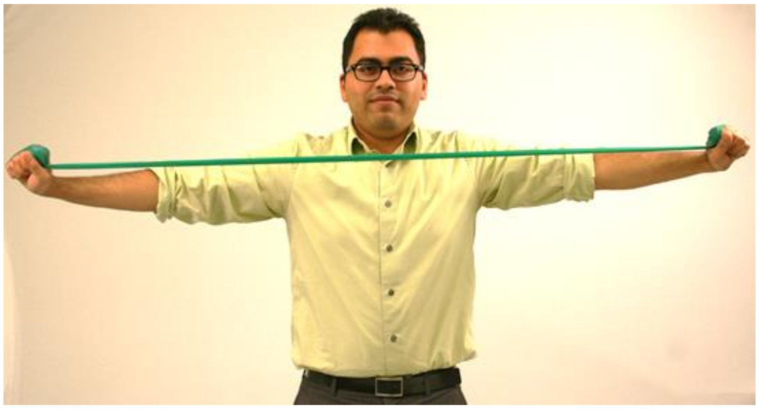

Slide 4
Session 2 Review (Activity 4: Using a Pedometer)

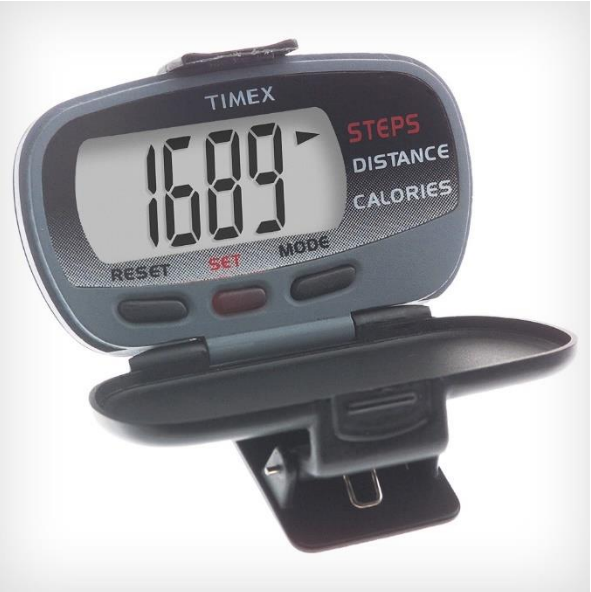

Slide 5
Activity 2: A Closer Look at Whole Grains (Brown Rice)

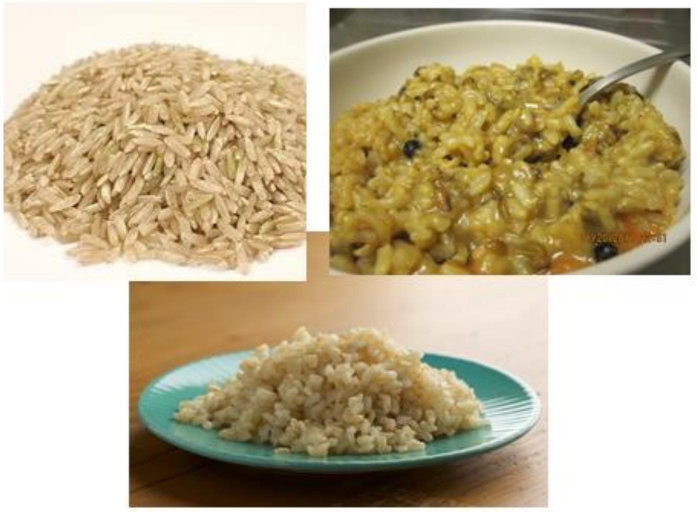

Slide 6
Activity 2: A Closer Look at Whole Grains (Wheat)

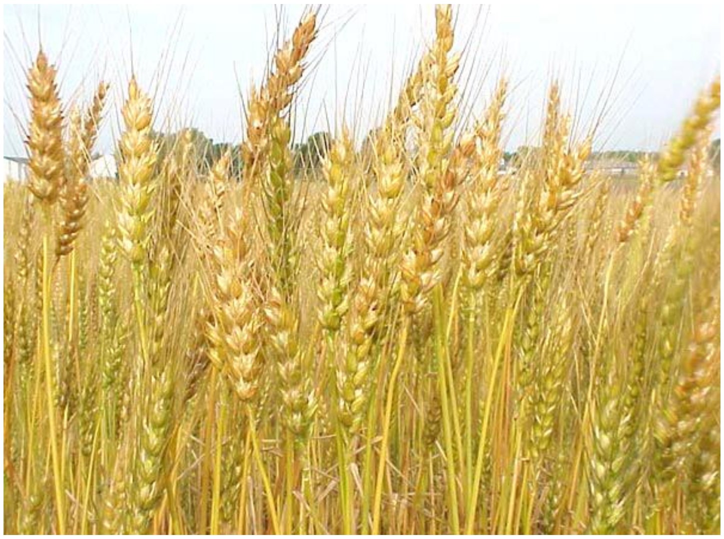

Slide 7
Activity 2: A Closer Look at Whole Grains (Refined Wheat)

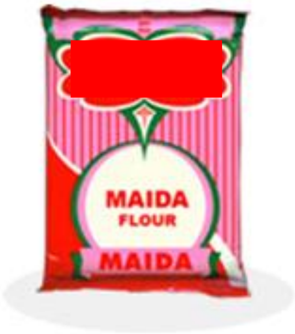

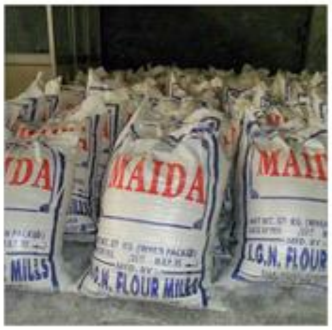

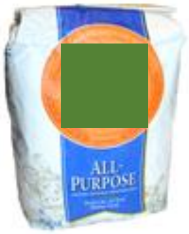

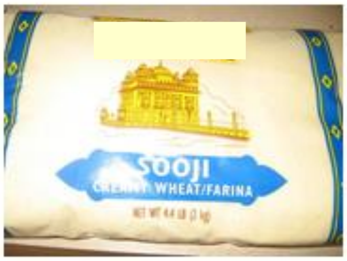

Slide 8
Activity 2: A Closer Look at Whole Grains (Whole Wheat)

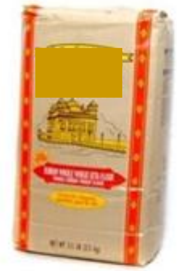

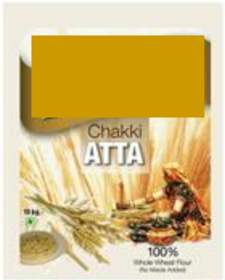

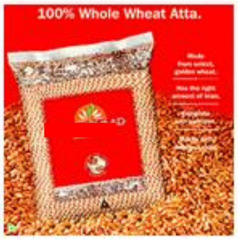

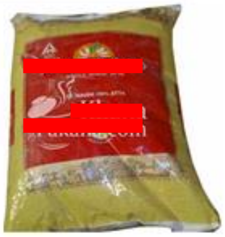

Slide 9
Activity 2: A Closer Look at Whole Grains (Jav (Barley))

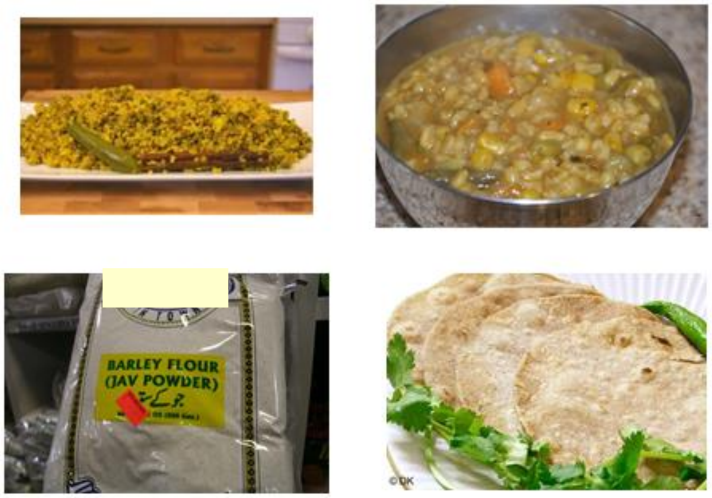

Slide 10
Activity 2: A Closer Look at Whole Grains (Bajra & Ragi (Millet))

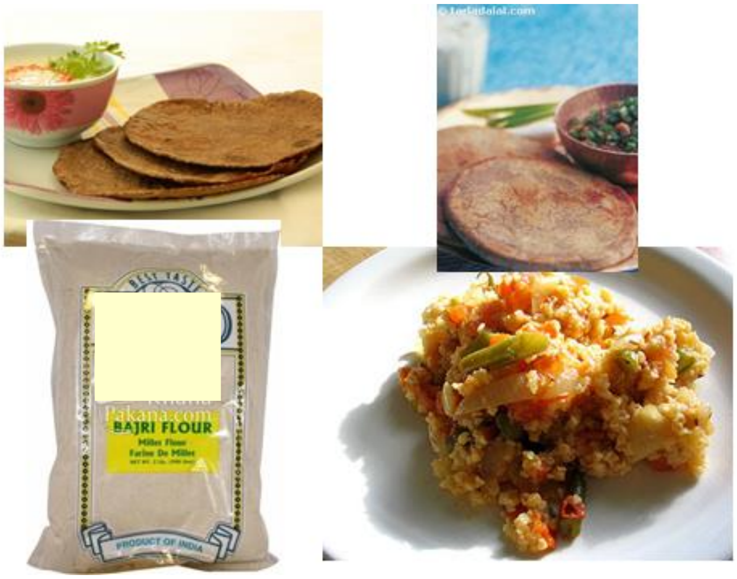

Slide 11
Activity 2: A Closer Look at Whole Grains (Jowar (Sorhum))

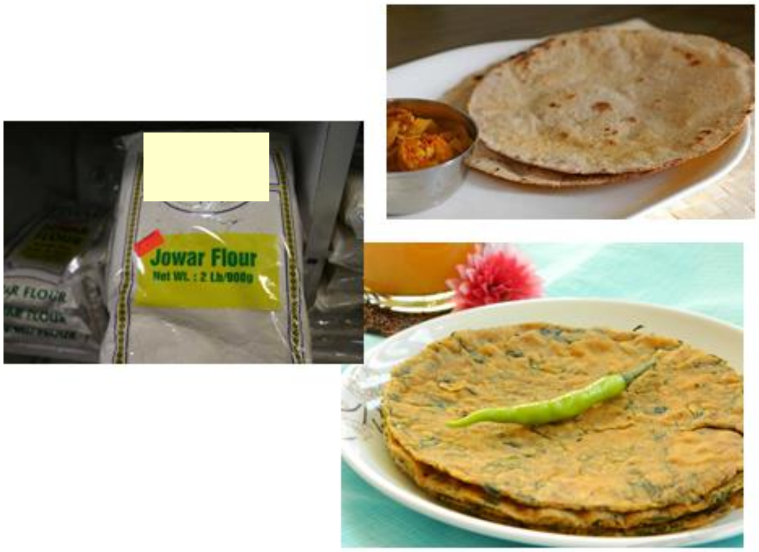

Slide 12
Activity 2: A Closer Look at Whole Grains (Oatmeal)

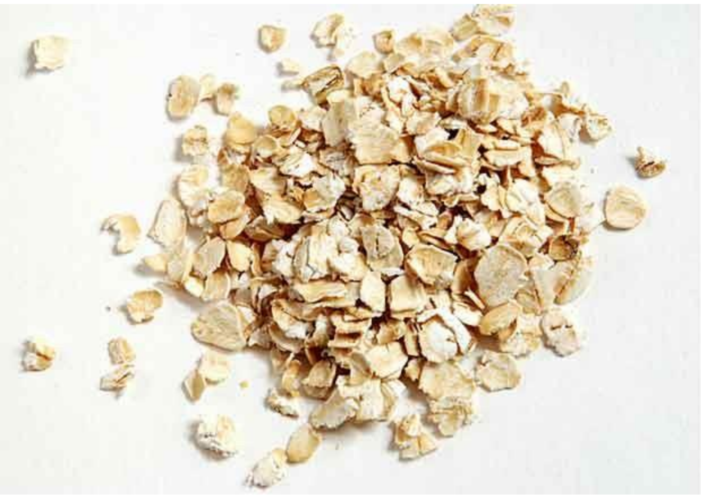

Slide 13
Activity 2: A Closer Look at Whole Grains (Rajgaro (Buckwheat))

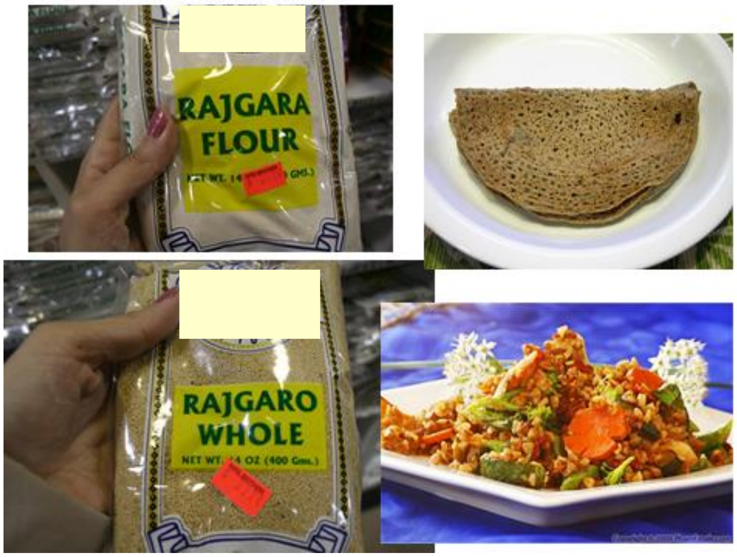

Slide 14
Activity 2: A Closer Look at Whole Grains (Quinoa)

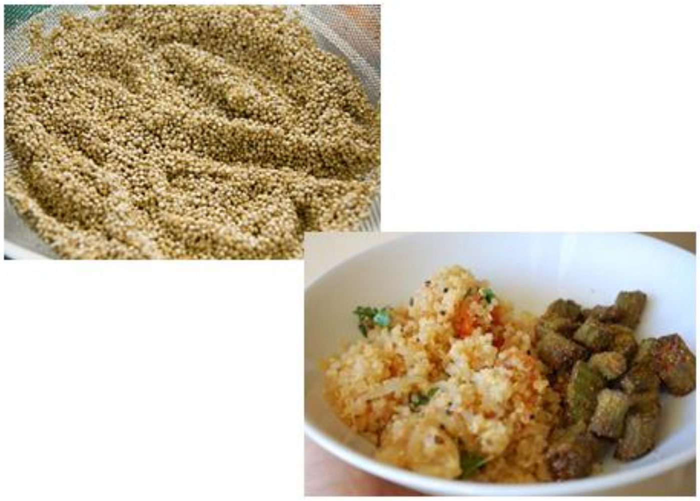

Slide 15
Activity 4: Preparing Vegetables (Typical Salad Photo)

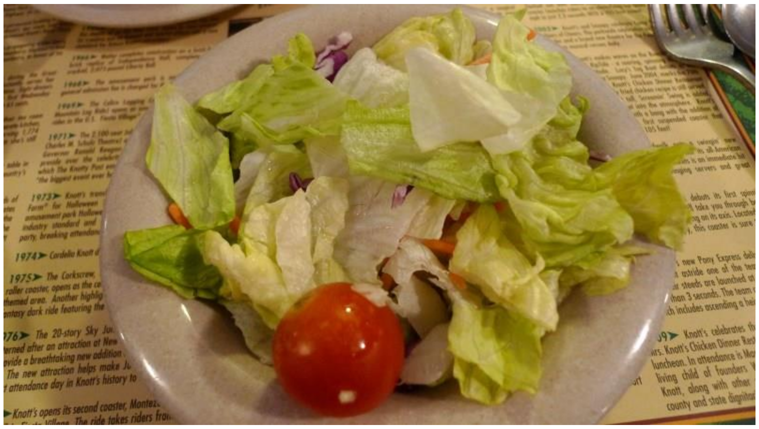

Slide 16:
Activity 4: Preparing Vegetables (South Asian Salad Example)

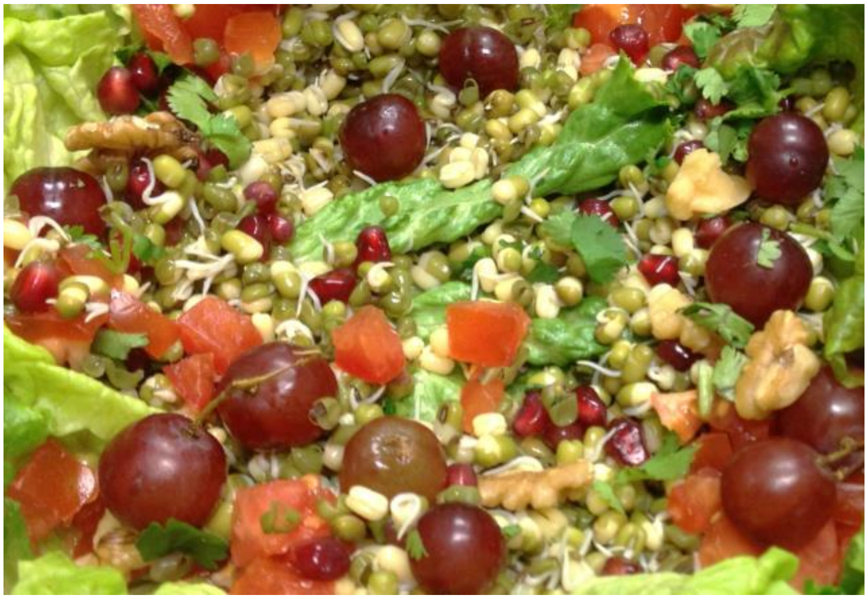

Slide 17:
Activity 4: Preparing Vegetables (Salad Comparison)

CONFIDENTIAL- Do not distribute, developed by Northwestern University

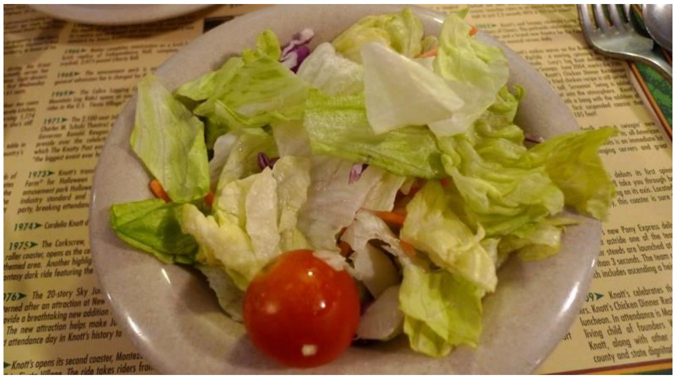

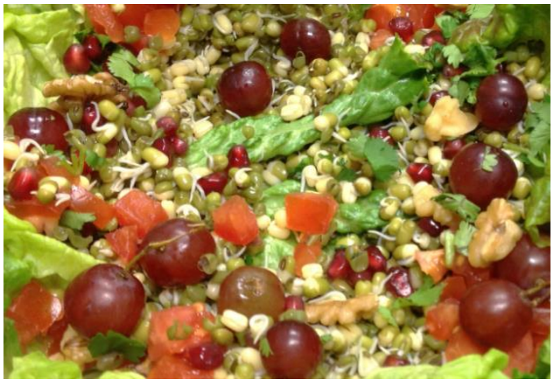

Slide 18
Activity 4: Preparing Vegetables (South Asian Salads)

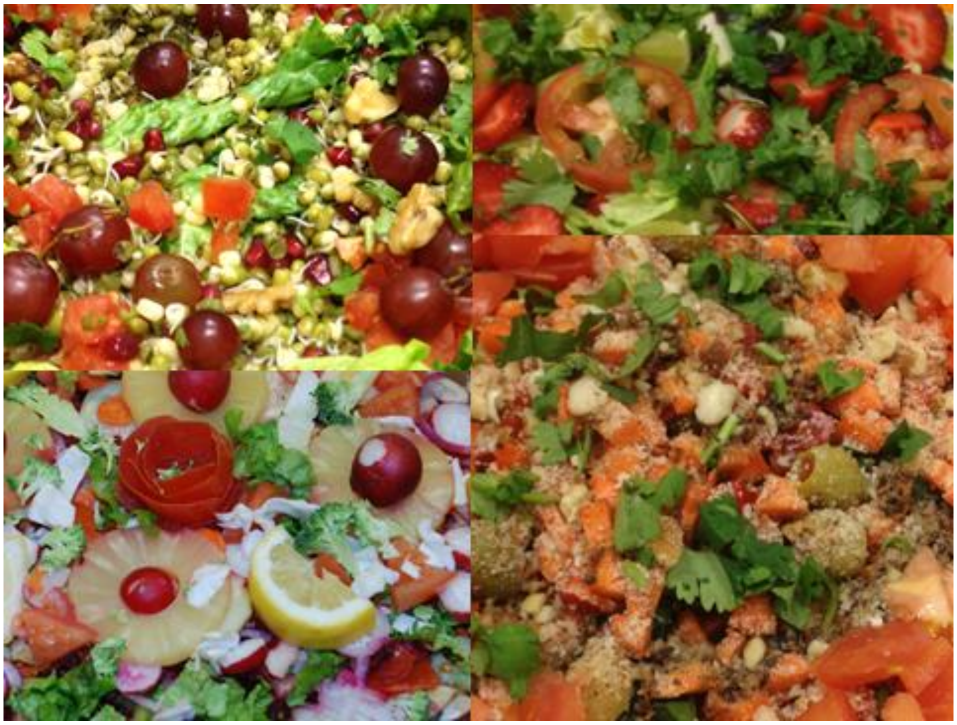

Slide 19
Session 3 Review (Activity 1: Why Whole Grains?)

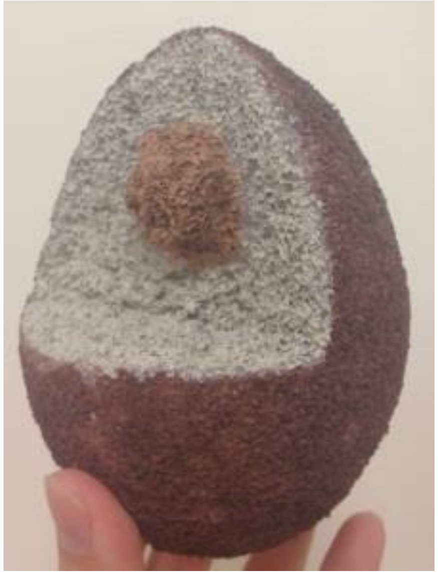

Slide 20
Session 3 Review (Activity 1: Why Whole Grains?)

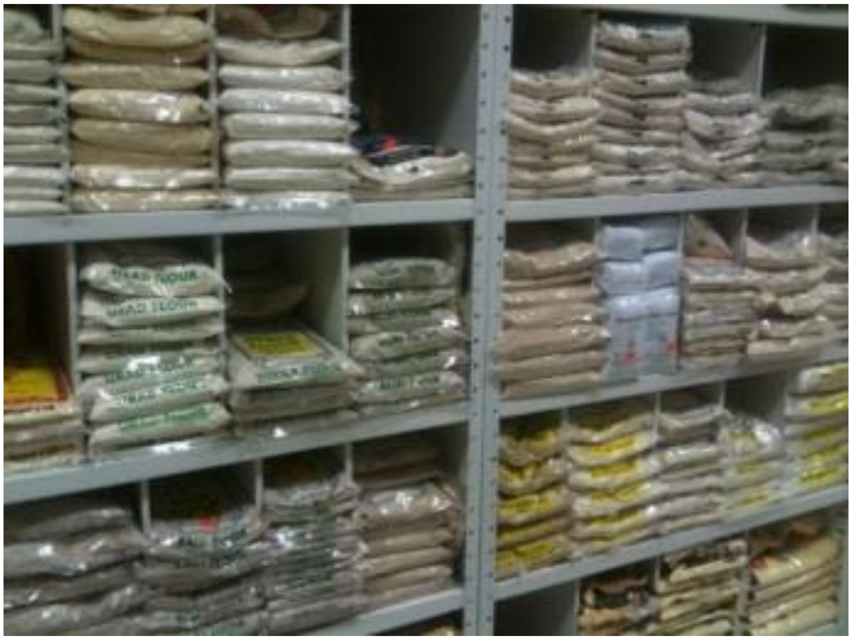

Slide 21
Session 3 Review (Activity 3: Am I eating enough fruits and vegetables?)

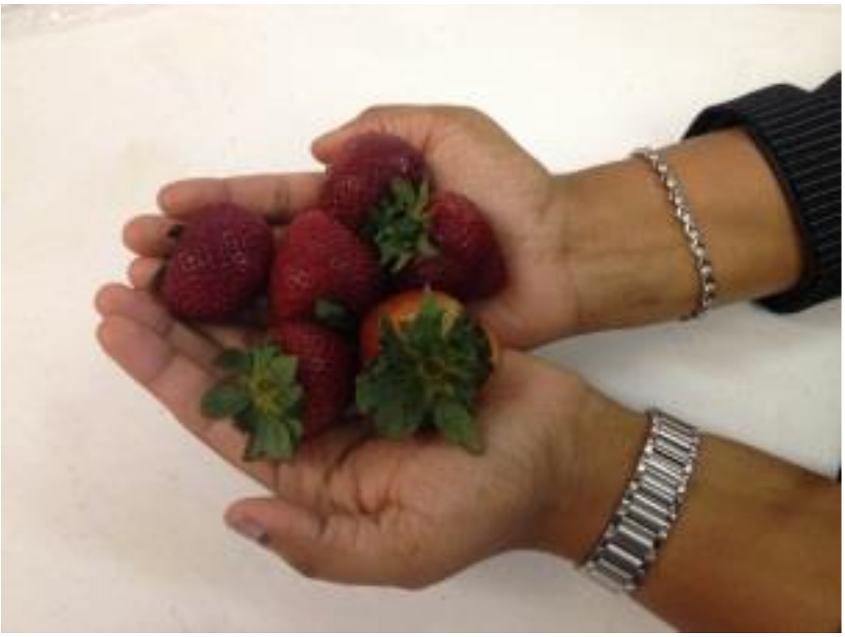

Slide 4
Session 3 Review (Activity 4: Preparing Vegetables)

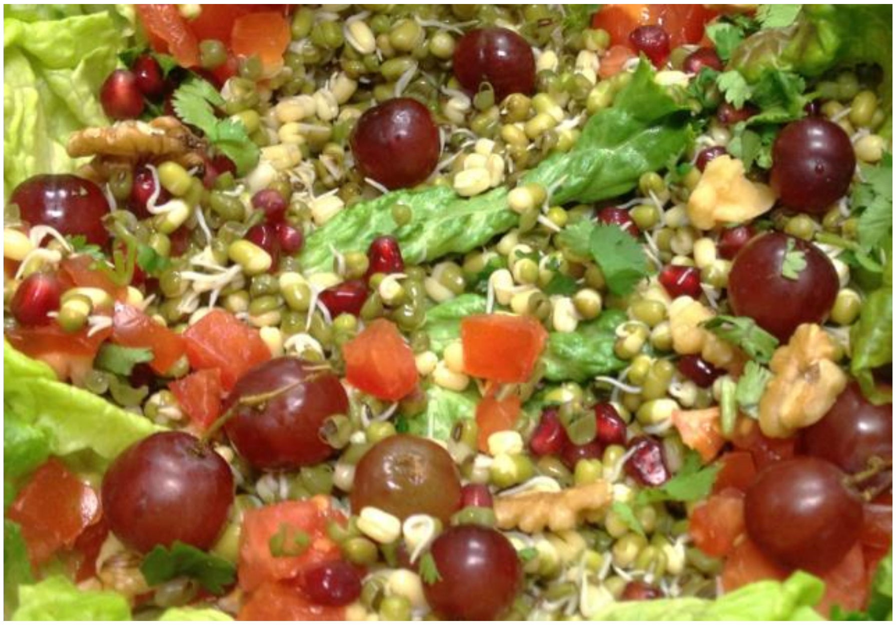

Appendix 3B: Whole Grain Dish

Barley Upma Recipe

For about 20 people

INGREDIENTS

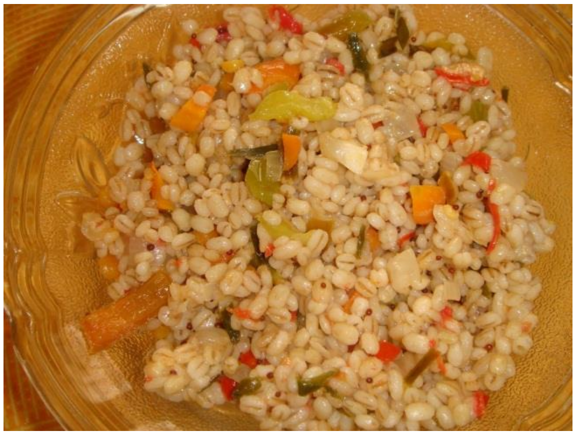

4 cups uncooked barley
4 onions, chopped
4 tomatoes, chopped
4 carrots, chopped
4 bell peppers, chopped
2 tablespoon canola oil
4 teaspoon mustard seeds
4 teaspoon urad dal
Green chilies
Curry leaves
Coriander leaves
Lemon juice from 1 lemon
Salt to taste

PREPARATION

1. Soak the barley the night before (for 8 to 10 hours)
2. Cook the barley in boiling water for 15 minutes or in a pressure cooker
3. Cut all the vegetables finely
4. In a pan, heat oil and add mustard seeds, urad dal, chilies
5. When the mustard seeds splutters add onions, peppers, carrot, and tomato and fry till for few minutes
6. Add the cooked barley
7. Add salt to taste and squeeze a lemon in it and cook it for another 5 minutes with the lid closed.
8. Garnish with curry leaves and coriander leaves

Adapted from: http://veenasvegnation.blogspot.com/2010/08/barley-upma.html

Appendix 3C: South Asian Salad Ingredients

Salad Preparation

For about 20 people

SALAD INGREDIENTS - put the following in separate bowls:

4 cans of chickpeas, drained and rinsed with water
2 small packages of sliced almonds (3 or 4 ounce bag)
5 lemons cut into wedges
2 oranges cut into wedges
4 tomatoes, finely chopped
3 cucumbers, not peeled, finely chopped
3 carrots, peeled, finely chopped
1 yellow bell pepper, finely chopped & 2 red bell peppers, finely chopped
3 green chilies, finely chopped
½ bunch fresh cilantro, finely chopped
3 apples, finely chopped
1 pomegranate
3 bags of mixed salad greens, finely chopped

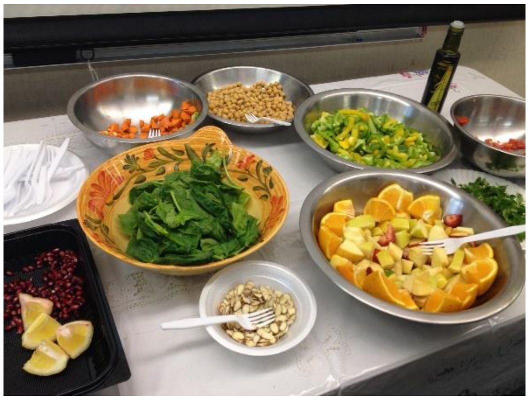

DRESSING INGREDIENTS

1 cup of olive oil
½ cup of mustard seeds
½ cup of cumin seeds
6 dried chilies
¼ cup of asafetida
20 dried curry leaves
Salt to taste

DRESSING PREPARATION

1. Put olive oil in a large frying pan with mustard seeds, cumin seeds, dried red chili, asafetida, and dried curry leaves. Turn the heat up to medium and wait for the seeds to start sizzling.
2. When the mustard seeds begin to pop, pour the oil and seeds in a separate bowl. Add salt to taste and mix thoroughly.

Appendix 3D: My Steps

My Steps

	Week 1	Week 2	Week 3	Week 4
Daily Goal				
Sunday				
Monday				
Tuesday				
Wednesday				
Thursday				
Friday				
Saturday				

Session 4: Eat Less Fat and Salt 70 min

Learning Objectives:
At the end of this session, participants will be able to:

- Describe ways to eat less fat and salt
- Identify foods high in saturated fat, trans fat, and unsaturated fat
- Recognize the benefits of using oils sparingly, limiting foods high in saturated fats, and avoiding foods high in trans fat
- Identify the serving size and the % Daily Value of total fat, saturated fat, and sodium amount in a packaged food

- Practice comparing fat and sodium content in packaged foods at the grocery store using the % Daily Value

Notes:

- Select a grocery store that participants frequently go to for the grocery store tour
- Contact the grocery store to arrange the grocery store tour
- If possible, chose a day and time that the grocery store is least busiest
- If possible, arrange for transportation to travel to grocery store as a group
- The closing will be completed before going to the grocery store to limit the amount of time spent at the grocery store
- Class evaluations for Session 4 may be completed at home and collected at the beginning of the next session

Overview of materials:

Media equipment

- Laptop
- Projector
- Speakers

Media

- *Eat Less Fat and Salt* video
- Session 4: Eat Less Fat & Salt PowerPoint (see Appendix 4A for PowerPoint Slides)

Other Supplies

- Box of pens
- Example of fats high in Saturated fat, Unsaturated fat, and Trans fat
- 3 Straws
- One teaspoon of salt in a sandwich bag
- About 1/10th of a teaspoon of salt in a sandwich bag
- Packaged foods with food labels

Handouts for participants:

- Appendix 4B: Food Label
- Class Evaluations

Review of the Previous Session

Materials needed:

Media equipment

- Laptop
- Projector

Media

- Session 4: Eat Less Fat & Salt PowerPoint (see Appendix 4A for PowerPoint Slides)

Show the Session 4: Eat Less Fat & Salt PowerPoint:

Slide 1: *"Last week we discussed fruits, vegetables and whole grains. We learned about the three parts of a whole grain and how eating whole grains can benefit our health."*

Slide 2: *"We then looked at the different whole grains that we can find at Patel Brothers and other grocery stores and we even tried a whole grain dish prepared by MAFS kitchen staff."*

Slide 3: *"We also learned about the recommendations to eat five servings of fruits and vegetables and learned what a serving of fruits and vegetables is."*

Slide 4: *"Finally, we learned about the best ways to cook our vegetables and made a tasty salad."*

Check-In with Progress

Materials needed:
None

Ask the participants if they tried anything new from the last class. Facilitate a brief discussion on barriers to trying something new and encourage the participants to problem solve and brainstorm other ways they can eat more whole grains, vegetables and fruits. Encourage the participants to try what they learned in class or to try other ways to eat more whole grains, vegetables and fruits.

Review the participants' steps recorded. Compare the steps recorded this past week from the steps recorded the first week. Provide affirmations to the participants that increased their steps. Take the time to have the participants share with one another the facilitators and barriers to increasing their steps.

Review Questions Asked

Materials needed:
None

Go through the responses to the questions asked last week, if any:

"At the end of last class, we asked everyone to write down any questions that they had from that session. We will briefly review the responses to these questions."

Introduce New Session

Materials needed:
None

"In this session we are going to talk about the importance of eating less fat and salt and we will learn about ways that we can eat less fat and salt. Everything that we learn today will help improve our health."

"We will start with a video and briefly discuss what we learned. Then we will do some activities that will help us better understand the information from the video. At the end of the class, we will review the

session and you will have an opportunity to write down any other questions that you may have about today's session."

Watch Video

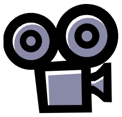

Materials needed:
Media equipment

- Laptop
- Projector
- Speakers

Media

- *Eat Less Fat and Salt* video

Play the Video

After playing the video, ask the participants to summarize the video and if they learned anything new from the video:

> *"Can someone tell me what the video talked about?"*
>
> *"Did you learn anything new that you didn't know before?"*

Activity 1: Understanding Different Types of Fat

Materials Needed:

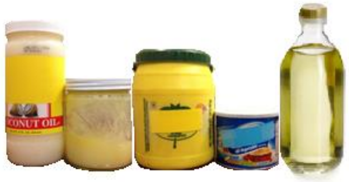

Other supplies

- Example of fats high in Saturated fat, Unsaturated fat, and Trans fat

"Now we are going to learn about the different types of fat and how they affect our health."

Show examples of foods high in saturated fat such as ghee and coconut oil:

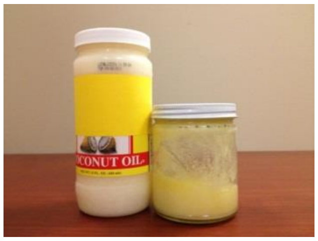

"Saturated fat is one type of fat. Foods that come from animals are high in saturated fat. These are foods like meat, milk, ghee and paneer. Palm oil and coconut oil are also high in saturated fat."

Explain that only foods from animals contain cholesterol:

> *"In session 1, we learned that our body can make cholesterol. Cholesterol is another type of fat. We also learned that we get cholesterol from the foods we eat. Cholesterol is found in animal foods like meat, milk, ghee and paneer because animals make cholesterol. Cholesterol is not found in plant foods."*

Show examples of foods high in trans fats such as shortening and vanaspati:

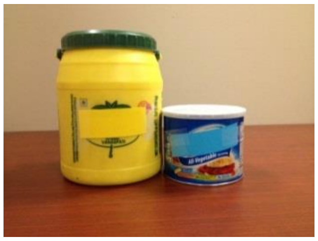

"There is another type of fat called trans fats. Shortening, vegetable ghee (vanaspati), stick margarines and some tub margarines are high in trans fat. These fats are common in fried foods made in restaurants, bakeries, packaged and street foods like samosa, pakoras, gulabjaman and jilebi."

Show examples of foods high in unsaturated fat such as vegetable oil, canola oil and olive oil:

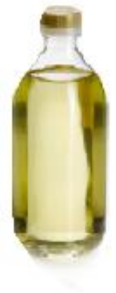

"Plant oils like vegetable oil, olive oil, canola oil, and peanut oil are high in unsaturated fat. Nuts, avocados, and fish like salmon and tuna are also high in unsaturated fat."

Explain how each type of fat can affect blood cholesterol levels:

"Our body needs some fat and cholesterol to be healthy. But, if we eat too much fat and cholesterol, it can be bad for our health."

"Foods high in saturated fats have been shown to increase LDL or bad cholesterol. Bad cholesterol is the fat that can clog arteries, so it is best to limit the amount of foods that are high in saturated fat."

"Trans fats also raise levels of bad cholesterol and they reduce levels of HDL or good cholesterol. Good cholesterol helps remove bad cholesterol that is left in the arteries. It is best to not eat foods high in trans fats. It is best to avoid foods that contain trans fat."

"Unsaturated fats can help raise good cholesterol and lower bad cholesterol. We should try to replace foods high in saturated fats for foods high in unsaturated fats. It is important not to eat too much of foods high in unsaturated fat because it can lead to weight gain."

**Key talking points:**	**Found in:**	**Effect on cholesterol:**
**Limit:**<br>**Saturated fats**	Animal fat and dairy foods: ghee, butter, paneer, cheese, milk, meat<br>Exception: Tropical oils like palm and coconut oil	Raises LDL (bad) cholesterol
**Avoid:**<br>**Trans fat**	Vegetable ghee (Vanaspati), shortening, stick margarines, and some tub margarines<br>May also be found in some packaged foods in the grocery store and fried foods from restaurants or bakeries	Raises LDL (bad) cholesterol and lower HDL (good) cholesterol

Use sparingly: **Unsaturated fats**	Plants foods: liquid vegetable oils such as olive oil, canola oil, corn oil, soybean oil, safflower oil and sunflower oil; seeds like sunflower seeds, flaxseeds, sesame seeds, avocados; nuts like almonds, cashews, peanuts, walnuts<br>Exception: Fatty fish like salmon	Improves blood cholesterol level when you use them in place of saturated and trans fats. May decrease LDL (bad) cholesterol as well as maintain HDL (good) cholesterol

Activity 2: How Clogged Arteries are caused

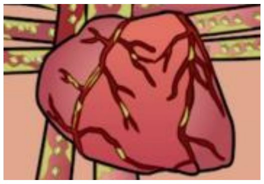

Materials Needed:
Other supplies

- Example of fats high in Saturated fat, Unsaturated fat, and Trans fat
- 3 straws

Demonstrate with the straw and the examples of fats to show how arteries can become clogged. Show the straw and explain that the straw is like an artery in the body:

"When we are born, our arteries are smooth like the inside of this straw."

Crinkle the straw so that it is no longer smooth but has rough bends and cracks:

"If you have diabetes, high blood cholesterol or high blood pressure, the arteries become rough and damaged like you saw in the video. The damaged arteries make it easier for bad cholesterol to stick to the walls of the arteries and cause a clog."

Dip the straw in the different fats to show how the fat can stick to the artery walls:

"This represents that if you eat a lot of foods high in saturated fat and trans fat, you can raise your levels of bad cholesterol and increase your chances of clogged arteries."

"This represents that if you replace foods high in saturated fat for foods high in unsaturated fat, you can improve your cholesterol levels. Use sparingly, though, because if you use too much oil it can lead to weight gain. Remember that exercise also helps make good cholesterol."

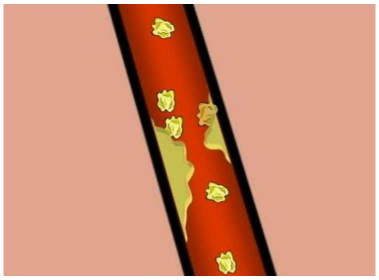

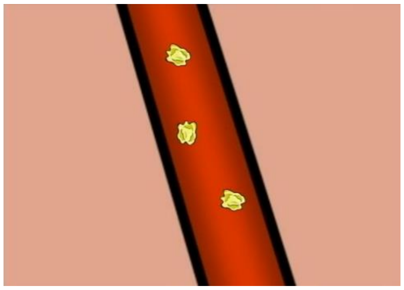

Clogged artery represented by straw dipped in ghee, vanaspati, shortening and coconut oil

Healthy artery represented by straw dipped in liquid plant oil

Activity 3: How to Read Food Labels

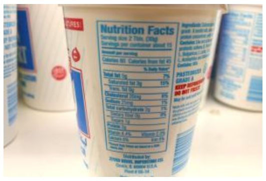

Materials Needed:
Media equipment

- Laptop
- Projector

Media

- Session 4: Eat Less Fat & Salt PowerPoint (see Appendix 4A for PowerPoint Slides)

Other supplies

- One teaspoon of salt in a sandwich bag
- About 1/10th of a teaspoon of salt in a sandwich bag
- Packaged foods with food labels

Handouts for participants:

- Appendix 4C:Food Label

Explain food sources of sodium and its effect on blood pressure:

> *"Sodium can be found in many different places. Sodium is found in salt. Sodium is also found in very small amounts in vegetables, grains, and beans. But most of the sodium that we eat comes from packaged foods and foods eaten outside of the home."*
>
> *"Eating too much salt can increase blood pressure and this can increase our chances of a heart attack."*

Hand out Appendix 4C: Food Label (one per participant):

> *"Food labels can be used to see how much fat and sodium there is in food. We are going to learn how to determine which foods are high in saturated fat and sodium using the food labels."*

Show the Session 4: Eat Less Fat & Salt PowerPoint:

> Slide 5: *"This section of the label is the serving size. If you eat this amount, then you will get the amount of fat and sodium that is listed below. What is the serving size on this food label?"*

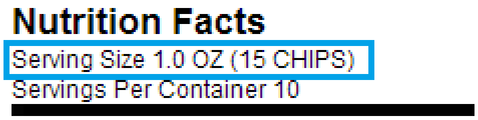

> *"Right, the serving size is one ounce which is 15 chips."*
>
> Slide 6: *"It is important to remember that the serving size may not always be the amount in the entire package. To see how much is in the entire package, look at the servings per container. What is the servings per container?"*

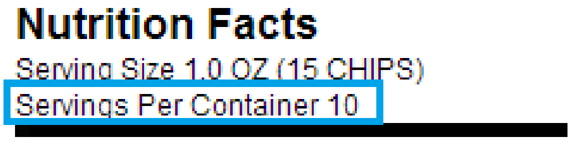

"Right, the servings per container is about 10. This means there are about 10 ounces or 150 chips in the entire package."

Slide 7: *"Now let's find the fat and sodium on the food label. All food labels will display:*

- *Total Fat*
- *Saturated Fat*
- *Trans Fat*
- *Cholesterol*
- *Sodium."*

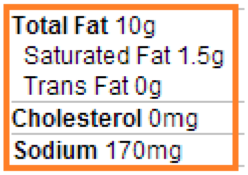

"Saturated fat, trans fat and unsaturated fat add up to the total fat but it is not required to have the unsaturated fat on the food label."

"In order to see how much is too much of total fat, saturated fat, cholesterol and sodium we can look at the % Daily Value (DV)."

Slide 8: *"The % DV shows how much of total fat, saturated fat, cholesterol or sodium is in one serving size on a scale of 0% to 100%, with 100% being the most you should eat in one day."*

"The %DV is based shows that total fat is less than 65 grams, saturated fat is less than 20 grams, cholesterol is less than 300 milligrams, and sodium is less than 2,400 milligrams.

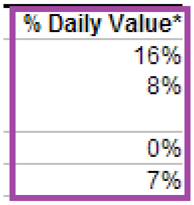

Show the one teaspoon of salt in a sandwich bag:

"This amount of salt is the maximum amount of sodium you should eat in one day. It contains 2400 milligrams of sodium or 100% DV of sodium. Remember, this sodium amount comes from the salt used when cooking and from the sodium that is in packaged foods."

"What is the %DV of sodium in this food?"

Show the 1/10th of a teaspoon of salt in a sandwich bag:

"Right, the % DV of sodium is 7%. This means that in one ounce, there is 7% of the total sodium recommended for one day. That is about how much is in this bag."

Explain general guidelines to follow when readings % DV

"The general rule is 5% DV is low and 20% DV is high per serving size. To reduce the amount of total fat, saturated fat, cholesterol and sodium, choose packaged foods with low %DV for fat and sdoium."

Show the Session 4: Eat Less Fat & Salt PowerPoint to explain trans fat on the food labels:

Slide 9: *"There are two things to remember when looking at trans fat on the label:*

1. *When you go to the grocery store, you will see that some foods like vanaspati and shortening have zero grams listed next to the trans fat. Even though some foods have zero grams trans fat they can still contain trans fat. Food companies have the permission to write 0 grams on the label when the trans fat is less than 0.5 grams in one serving size. One serving on the label of vanaspati and shortening is one tablespoon. When we cook, we may add more than that and this adds up and you may end up with a lot of trans fats through the day. Vanaspati, shortening and some margarines contain trans fat because they are made with partially hydrogenated oils. To see if a food had trans fat, look at the ingredients list for the words partially hydrogenated oils.*

2. *Also, trans fat do not list a %DV because experts could not provide a value for trans fat but we should try to avoid foods high in trans fat. Like we talked about earlier, trans fat may increase bad cholesterol and decrease good cholesterol and this can increase your chances of a heart attack."*

Nutrition Facts

Serving Size 1 TABLESPOON
Servings Per Container 38

Amount Per Serving	
**Calories** 110	Calories from Fat 110
	**% Daily Value***
**Total Fat** 12g	18%
Saturated Fat 3g	16%
Trans Fat 0g	
**Cholesterol** 0mg	0%
**Sodium** 0mg	0%
**Total Carbohydrate** 0g	0%
Dietary Fiber 0g	0%
**Protein** 0g	

Ingredients

Ingredients: Soybean Oil, Fully Hydrogenated Palm Oil, Partially Hydrogenated Palm and Soybean Oils, Mono and Diglycerides, TBHQ and Citric Acid (Antioxidants).

Pass out the packaged foods with food labels to the participants and have them practice finding the serving size and %DV for total fat, saturated fat, cholesterol and sodium:

Key talking points:

- The amount of fat and sodium that is listed on the food label is the amount found in one serving size. Always make sure to check the serving size amount.
- Choose foods with low %DV in total fat, saturated fat, cholesterol, and sodium.
- The general rule is 5% DV is low per serving size.
- Packaged foods like spice packets, frozen meals, jarred pickle mixes, and snack mixes are usually high in sodium.
- Only animal products or foods made with animal products like milk, yogurt, ghee, and meat contain cholesterol.
- Foods made with animal products or made with coconut oil or palm oil are high in saturated fat.

- Vegetable ghee (vanaspati), shortening, stick margarines and some tub margarines can contains trans fat.
- To see if a food contains trans fat, look for ‘partially hydrogenated oils’ in the ingredient list.

Review of Current Session

Materials Needed:
Media equipment

- Laptop
- Projector

Media

- Session 4: Eat Less Fat & Salt PowerPoint (see Appendix 4A for PowerPoint Slides)

Show the Session 4: Eat Less Fat & Salt PowerPoint:

> *"Before we go to the grocery store, let's review what we have learned today*
>
> Slide 10: *"In the first activity, we discussed the different types of fat and learned that it is important to limit foods high in saturated fats, avoid foods high in trans fat and use oil high in unsaturated fat instead because it is better for our heart. But remember that using too much oil can lead to weight gain."*
>
> Slide 11: *"In activity two, we talked about how the different types of fat affect the arteries in our body. Foods high in saturated fat and trans fat can raise bad cholesterol and can clog arteries. Foods high in unsaturated fat are good for your heart and should be used in small amounts."*
>
> Slide 12: *"In activity three, we learned how to determine if a food has too much fat, saturated fat, and sodium by looking at the serving size and %DV on the food label."*
>
> Slide 13: "*In the last activity, we will practice looking at the serving size and %DV on the food labels to see if it has too much fat, saturated fat, and sodium."*

Encourage the participants to try something knew that they learned in today's session:

> *"We would like for you to try something you learned in class today. We will talk about it at the beginning of next class."*

Closing

Materials Needed:
Handouts for participants:

- Class Evaluations

Reminder for the next session:

> *"At the next session, we will be talking about how to control your weight and maintain a healthy weight. Please remember to continue wearing your pedometer, to record your steps in the worksheet and to bring it with you to the next class."*

Hand out Class Evaluations (one per participant):

> *"Before next session, we would like for everyone to fill out this class evaluation. This will help MAFS and Northwestern University improve these classes."*

Collect Questions from this session:

> *"Also we would like for everyone to write down a question on the back of the class evaluation. The questions should be about this session.* ***Please do not include any personal health issues****. We will be doing this at the end of each session because we want to make sure that everyone's questions get answered. We will collect this when you are finished and we will answer the questions before we start the next session."*

Thank the participants for attending and confirm the date and time for the next session after Activity 4: Grocery Store Trip (page 12).

Activity 4: Grocery Store Trip

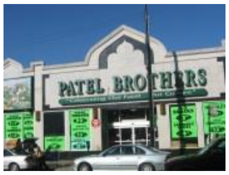

Materials Needed:
None

Split into groups; about two or three groups depending on the size of the class. Groups should start in different parts of the grocery store and rotate clockwise. Below is an example of stations within a grocery store:

Packaged food aisle such as packaged Indian snacks, packaged spice mixes, and jarred pickle mixes

In this station, have the participants work together to pick out at least one spice package and at least one snack package that they usually buy to see how much fat, saturated fat, trans fat, cholesterol and sodium that food has.

Dairy aisle

In this station, have the participants work together to compare whole milk and skim milk, full fat yogurt and low fat yogurt, and full fat paneer and low fat paneer by reading the total fat and saturated fat on the food label.

Frozen foods aisle

In this station, have the participants work together to pick out at least one frozen vegetable bag and one frozen meal package to see how much fat, saturated fat, trans fat, and cholesterol and sodium that food has.

Note: The participants may start reading food labels of grains and dals. Explain to the participants that what we learned in class about food labels focused on sodium and different types of fats. Packaged flours and other grains and dals will have very little or no sodium or fat. The important thing to remember about grains is to choose whole grains. If time is allotted, encourage the participants to recall examples of whole grains and have the participants read atta (flour) labels to identify 100% whole grain flours.

Appendix 4 A: Session 4: Eat Less Fat & Salt PowerPoint Slides

Slide 1
Session 3 Review (Activity 1: Why Whole Grains?)

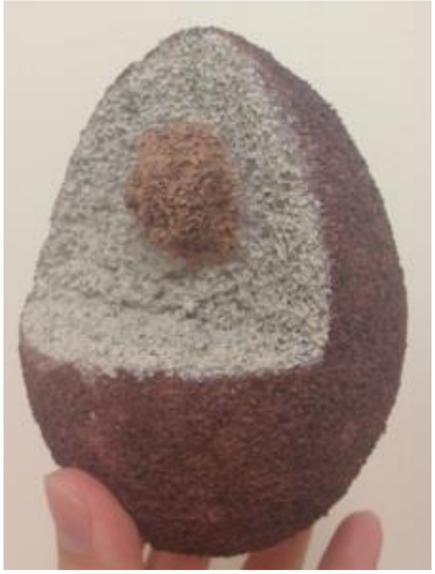

Slide 2
Session 3 Review (Activity 2: A Closer Look at Whole Grains)

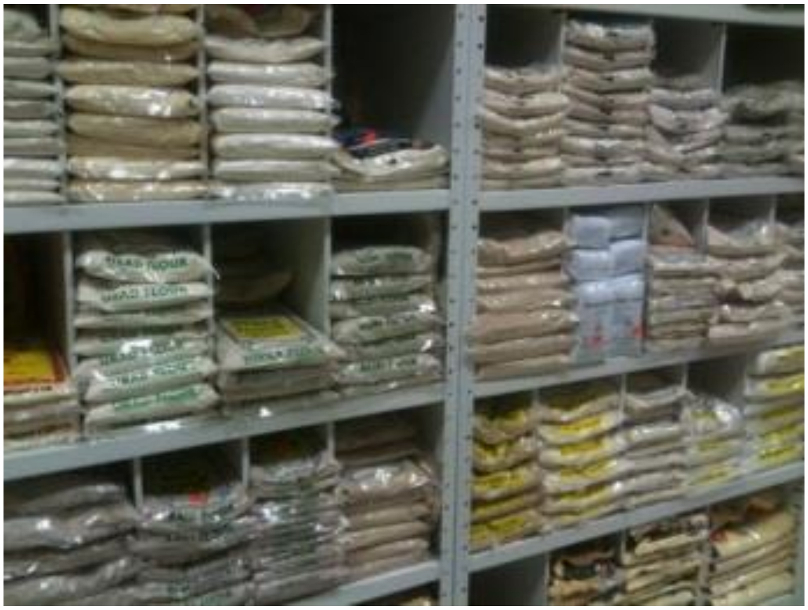

Slide 3
Session 3 Review (Activity 3: Am I eating enough fruits and vegetables?)

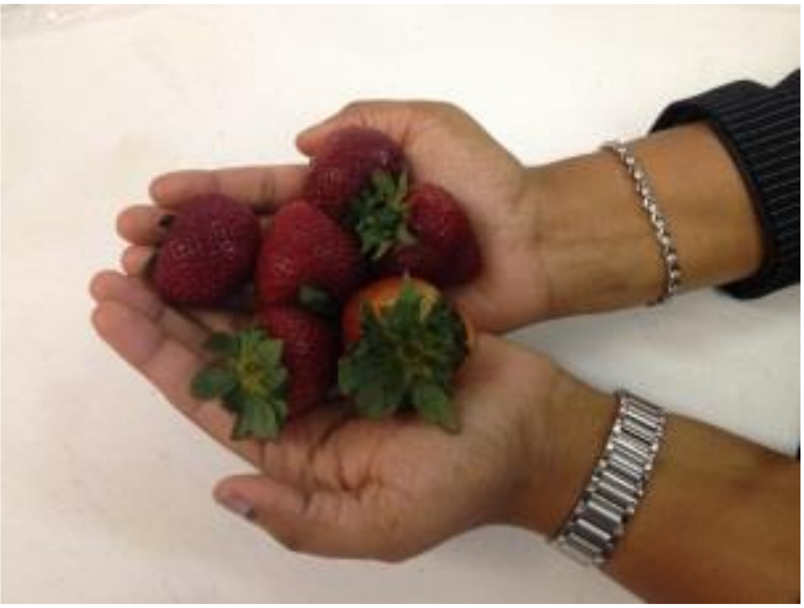

Slide 4
Session 3 Review (Activity 4: Preparing Vegetables)

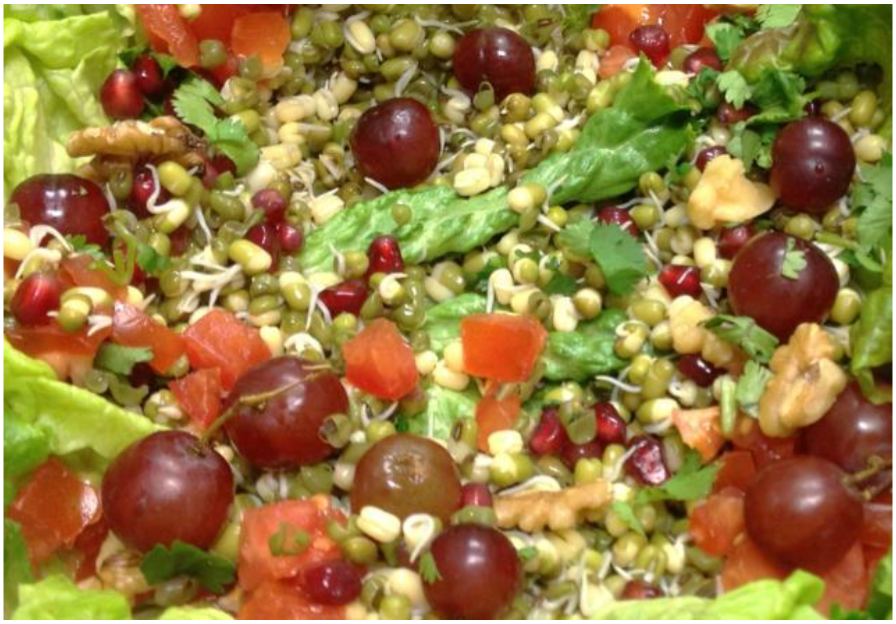

Slide 5
Session 4 Activity 3: How to Read Food Labels (Serving Size)

Nutrition Facts
Serving Size 1.0 OZ (15 CHIPS)
Servings Per Container 10

Amount Per Serving	
**Calories** 160	Calories from Fat 90
	**% Daily Value***
**Total Fat** 10g	16%
Saturated Fat 1.5g	8%
Trans Fat 0g	
**Cholesterol** 0mg	0%
**Sodium** 170mg	7%
**Potassium** 350mg	10%
**Total Carbohydrate** 15g	5%
Dietary Fiber 1g	5%
Sugars 1g	
**Protein** 2g	

Slide 6
Session 4 Activity 3: How to Read Food Labels (Servings Per Container)

Nutrition Facts
Serving Size 1.0 OZ (15 CHIPS)
Servings Per Container 10

Amount Per Serving	
**Calories** 160	Calories from Fat 90
	**% Daily Value***
**Total Fat** 10g	16%
Saturated Fat 1.5g	8%
Trans Fat 0g	
**Cholesterol** 0mg	0%
**Sodium** 170mg	7%
**Potassium** 350mg	10%
**Total Carbohydrate** 15g	5%
Dietary Fiber 1g	5%
Sugars 1g	
**Protein** 2g	

Slide 7
Session 4 Activity 3: How to Read Food Labels (Total Fat, Saturated Fat, Trans Fat, Cholesterol, and Sodium)

Nutrition Facts
Serving Size 1.0 OZ (15 CHIPS)
Servings Per Container 10

Amount Per Serving	
**Calories** 160	Calories from Fat 90
	**% Daily Value***
**Total Fat** 10g	16%
Saturated Fat 1.5g	8%
Trans Fat 0g	
**Cholesterol** 0mg	0%
**Sodium** 170mg	7%
**Potassium** 350mg	10%
**Total Carbohydrate** 15g	5%
Dietary Fiber 1g	5%
Sugars 1g	
**Protein** 2g	

Slide 8
Session 4 Activity 3: How to Read Food Labels (% Daily Value)

Nutrition Facts
Serving Size 1.0 OZ (15 CHIPS)
Servings Per Container 10

Amount Per Serving	
**Calories** 160	Calories from Fat 90
	**% Daily Value***
**Total Fat** 10g	16%
Saturated Fat 1.5g	8%
Trans Fat 0g	
**Cholesterol** 0mg	0%
**Sodium** 170mg	7%
**Potassium** 350mg	10%
**Total Carbohydrate** 15g	5%
Dietary Fiber 1g	5%
Sugars 1g	
**Protein** 2g	

Slide 9
Session 4 Activity 3: How to Read Food Labels (Trans Fat %DV & Ingredients)

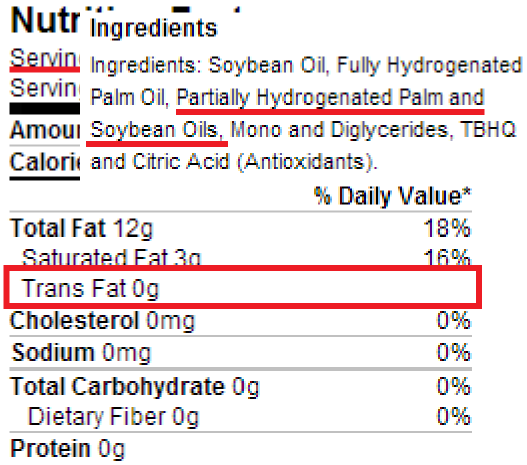

Slide 10
Session 4 Review (Activity 1: Understanding Different Types of Fat)

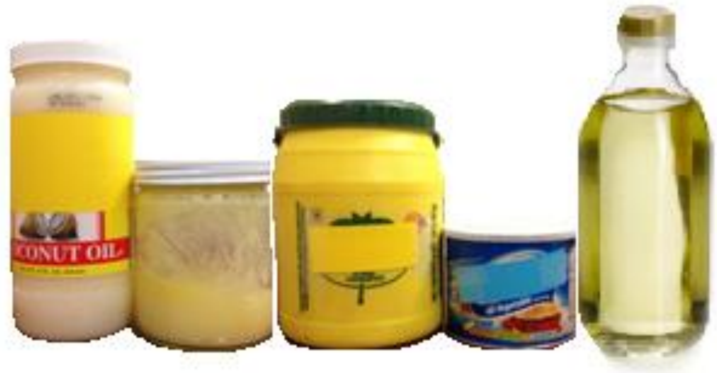

Slide 11
Session 4 Review (Activity 2: How Clogged Arteries are caused)

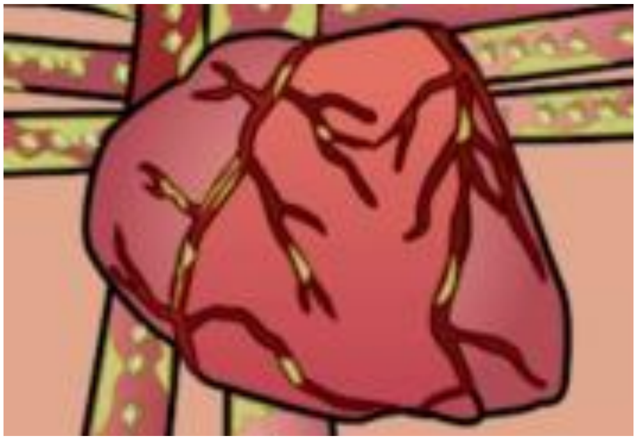

Slide 12
Session 4 Review (Activity 3: How to Read Food Labels)

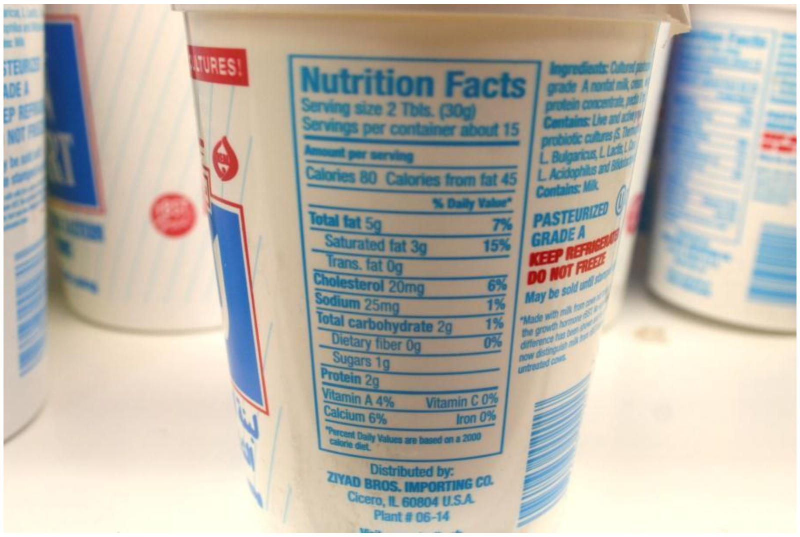

Slide 13
Session 4 Review (Activity 4: Grocery Store Tour)

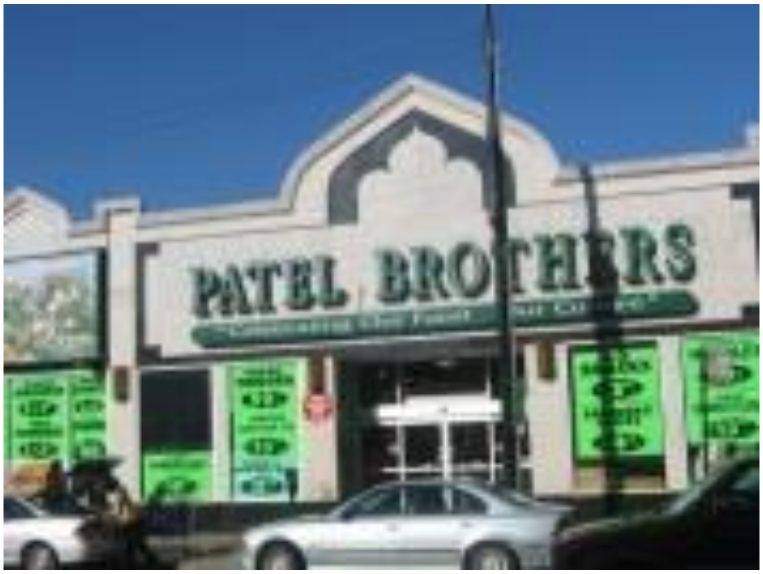

Appendix 4 B: Food Label

Step 1: Look at the serving size

Step 2: Find fat and sodium

Step 3: Choose foods with <u>low</u> % Daily Value of fat and sodium (about 5% or less)

Nutrition Facts

Serving Size 1.0 OZ (15 CHIPS)
Servings Per Container 10

**Amount Per Serving**	
**Calories** 160	Calories from Fat 90
	**% Daily Value***
**Total Fat** 10g	16%
Saturated Fat 1.5g	8%
Trans Fat 0g	
**Cholesterol** 0mg	0%
**Sodium** 170mg	7%
**Potassium** 350mg	10%
**Total Carbohydrate** 15g	5%
Dietary Fiber 1g	5%
Sugars 1g	
**Protein** 2g	

Session 5: Controlling Your Weight 70 min

Learning Objectives:
At the end of this session, participants will be able to:

- Explain why it is important to maintain a healthy weight
- Recognize the ideal waist measurement for a man and a woman
- Recognize the reasons weight loss diet plans that limit foods, promote rapid weight loss and cut out exercise can be unhealthy for our bodies
- Identify ways to maintain a healthy weight
- Demonstrate ways to modify a recipe to include fruits, vegetables and whole grains and less fat
- Practice making a healthy plate
- Identify ways to avoid overeating

Notes:

- Collect the class evaluations before the class starts if the participants completed it at home

Overview of materials:
Media equipment

- Laptop
- Projector
- Speakers

Media

- *Controlling Your Weight* video
- Session 5: Controlling Your Weight PowerPoint (see Appendix 5A for PowerPoint Slides)

Other Supplies

- Box of pens
- Food models
- Rotis/Chappattis
- 9-inch paper plates
- 3 8-ounce clear plastic cups labeled ‘Skim’, ‘2%’, and ‘Whole’
- Ghee
- Measuring spoons

Handouts for participants:

- Appendix 5B: Plate Method
- Class Evaluations

Review of the Previous Session

Materials needed:
Media equipment

- Laptop
- Projector

Media

- Session 5: Controlling Your Weight PowerPoint (see Appendix 5A for PowerPoint Slides)

Show the Session 5: Controlling Your Weight PowerPoint:

> Slide 1: *"Last week, we discussed the different types of fat and learned that it is important to limit foods high in saturated fats, avoid foods high in trans fat and use oil high in unsaturated fat instead because it is better for our heart. But remember that using too much oil can lead to weight gain."*
>
> Slide 2: *"We then talked about how the different types of fat affect the arteries in our body. Foods high in saturated fat and trans fat can raise bad cholesterol and can clog arteries. Foods high in unsaturated fat are good for your heart and should be used in small amounts."*
>
> Slide 3: *"We also learned how to determine if a food has too much fat, saturated fat, and sodium by looking at the serving size and %DV on the food label."*
>
> Slide 4: "*Finally, we went to a grocery store to practice looking at the serving size and %DV on the food labels to see if it has too much fat, saturated fat, and sodium."*

Check-In with Progress

Materials needed:
None

Ask the participants if they tried anything new from the last class. Facilitate a brief discussion on barriers to trying something new and encourage the participants to problem solve and brainstorm other ways they can eat more whole grains, vegetables and fruits. Encourage the participants to try what they learned in class or to try other ways to eat more whole grains, vegetables and fruits.

Review the participants' steps recorded. Compare the steps recorded this past week from the steps recorded the first week. Provide affirmations to the participants that increased their steps. Take the time to have the participants share with one another the facilitators and barriers to increasing their steps.

Review Questions Asked

Materials needed:
None

Go through the responses to the questions asked last week, if any:

"At the end of last class, we asked everyone to write down any questions that they had from that session. We will briefly review the responses to these questions."

Introduce New Session

Materials needed:
None

"In this session we are going to talk about the importance of controlling our weight and we will learn about ways that we can control our weight. Everything that we learn today will help improve our health."

"We will start with a video and briefly discuss what we learned. Then we will do some activities that will help us better understand the information from the video. At the end of the class, we will review the session and you will have an opportunity to write down any other questions that you may have about today's session."

Watch Video

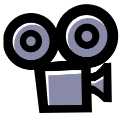

Materials needed:
Media equipment

- Laptop
- Projector
- Speakers

Media

- *Controlling Your Weight* video

Play the Video

After playing the video, ask the participants to summarize the video and if they learned anything new from the video:

"Can someone tell me what the video talked about?"

"Did you learn anything new that you didn't know before?"

Activity 1: Weight Loss Diets Discussion

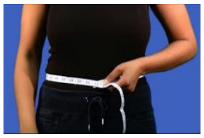

Materials Needed:
None

Ask the participants about weight loss information that they have heard and read:

"There is a lot of information about the best ways to lose weight. What are some things you have heard from friends or seen about how to lose weight?"

Summarize the participants' responses:

"These weight loss diet plans, pills and products offer rapid weight loss, limit some foods and do not include exercise. It can be tempting to try these weight loss methods but they can be unhealthy for our bodies":

Limiting foods
"It is important to not focus on individual foods but rather think about eating a variety of foods in a balanced way. If you limit some foods, you may not get the nutrients your body needs."

Rapid Weight loss
"Losing weight quickly may seem great but it can be unsafe. If you lose weight quickly, you may also lose muscle, bone and water. Also, if you lose weight quickly, you may be more likely to regain the weight back quickly. A healthy weight to lose is about ½ - 1 pound a week."

No need for exercise
"Exercise is not only important for maintaining a healthy weight but also important for keeping your heart healthy, keeping your bones strong and maintaining your muscles."

"The best way to lose weight is to make healthy food choices, eat small portions and incorporate exercise into your daily life. Today we will review what we learned in Sessions 3 & 4 and discuss how to eat healthy to control our weight."

Activity 2: Recipe Modification

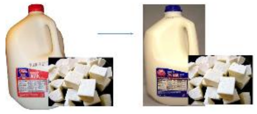

Materials needed:
Media equipment
- Laptop
- Projector

Media
- Session 5: Controlling Your Weight PowerPoint

Handouts for participants
- 'Appendix 5B: Recipe Modification

"In sessions 3 & 4, we talked about the importance of eating more fruits, vegetables and whole grains and less fat and salt to help prevent a heart attack. Now we will see how we can use fruits, vegetables and whole grains, and less fat in the dishes that we make to help control our weight."

Show the Session 5: Controlling Your Weight PowerPoint:

Slide 5: *"For example, these ingredients can be used to make traditional Palak Paneer. The ingredients are full fat paneer, cream, ghee and salt. How can we change this dish to make it healthier and to help with controlling our weight?"*

Summarize the participants' responses.

Slide 6: *"Right, instead of using paneer made with whole milk, you can try using paneer made with 2% milk. You can even use tofu because it has less fat than paneer. Instead of using cream, try using half cream and half low fat milk or evaporated skim milk to cut back on the fat. Instead*

of using ghee, try using less ghee or replacing with less oil. Also, when cooking, it is important to use a small amount of salt for our health."

Have the participants choose one recipe below to work in groups to modify the recipe:

Slide 7 & 8: *"Here is a recipe for Upma and for Kebab. In groups, choose one of these dishes and discuss ways to modify the dish. I will give everyone about five minutes and then we will share our ideas with one another."*

After about five minutes, ask each group to share their ideas with the entire class. Make sure to summarize their responses:

"Now I'd like for everyone to share their ideas with the class.

Slide 9: (Upma Recipe) *"Use whole grains like brown rice, barley or quinoa. Add some vegetables. In session 3 we talked about how fruits, vegetables and whole grains have fiber which help with making us feel fuller for longer. Also make sure to use a smaller amount of ghee and oil and a smaller amount of salt. Eating too much oil and ghee can lead to weight gain."*

Slide 10: (Kebab) *"Use leaner cuts of meat and remove the fat. You can also try using chicken or dal instead of beef. Make sure to use a smaller amount of ghee and oil. Broil or grill the meat instead of frying so you use less fat. If you do use eggs, you can use two egg whites instead of using one egg."*

Emphasize that making gradual changes to the way food is prepared will help with improving their health.

Key talking points:

- Find ways to add fruits and vegetables to your dishes
- Choose whole grains as ingredients
- Choose ingredients with less fat like chicken with no skin
- Cook foods with less oil
- Choose food preparation methods that use no fat like pressure cooking, steaming, broiling, grilling or microwaving

Activity 3: Building a Healthy Plate

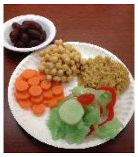

Materials needed:
Other supplies

- Food models
- Rotis/Chappattis
- 9-inch paper plates
- 3 8-ounce clear plastic cups labeled 'Skim', '2%', and 'Whole'
- Ghee
- Measuring spoons

Handouts for participants

- Appendix 5C: Plate Method

"As the video mentioned, the best way to lose weight is to eat a smaller amount of food at each mea. We are going to learn about the right amount of food to eat and we are going to review what we learned about the types of food we should eat by making a plate."

"We want to make sure we have enough of the right foods on our plate so that we are getting all the nutrients we need. This will also help us make sure that we feel satisfied and full without having too much food on our plate."

Have the participants build a healthy plate in groups using the food models and rotis/chappattis:

> *"We would like everyone to get into groups to build a plate based on what you have learned in these classes and seen in the videos. This is the plate we should eat for lunch or dinner."*

Pass out the plates, food models and rotis/chappattis and help the participants identify the food models if needed.

After about five minutes, ask each group to present their plates. Make sure to summarize their responses:

Key talking points:

- Make half your plate vegetables
- Try to incorporate both cooked and raw vegetables
- Making half your plate vegetables ensures that we get our three servings of vegetables in one day
- Make ¼ of your plate whole grains (about the amount that fits in one hand)
- Reduce the amount of rotis/chappattis eaten (if you are used to eating four rotis during one meal, try cutting down to three or two rotis.)
- Make ¼ your plate protein like dals (about the amount that fits in your hand) or meat (about the size of the palm of your hand)

Emphasize choosing low fat or skim milk

> *"Sometimes we have milk, cheese or yogurt with our meal. It is important to choose milk that is lower in fat. 2% and skim milk also have less total fat and less saturated fat than whole milk."*

Use the measuring spoons, the labeled plastic cups and ghee to show how much fat there is in skim, 2% and whole milk and pass it around the table for the participants to see:

- In the cup labeled ‘Whole’, show eight grams of fat in whole milk by putting ½ tablespoon of Ghee
- In the cup labeled ‘2%’, show five grams of fat in 2% milk by putting 1/3 tablespoon of Ghee
- Leave the cup labeled ‘Skim’ empty to show that there are zero grams of fat in skim milk

Emphasize including fruits in during or in between their meals.

> *“If you are still hungry, we encourage you to have more vegetables or to have a fruit after lunch or dinner. Fruit can also be eaten at breakfast. If you do not want to eat fruit during breakfast, fruit can also be eaten in between meals if we feel a little hungry. This will ensure that we get 2 servings of fruits in one day.”*

Handout Appendix 5C: Plate Method (one for each participant)

Activity 4: Ways to Avoid Overeating

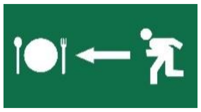

Materials needed:
None

"Now we would like to talk about eating out. This is important because when we eat out, there may be a lot of food. Think about the times you have gone out to eat at a friend or family member's house for a special occasion like a birthday or wedding. What types of foods do you eat?"

"When we are invited to a special event or a party, it is a special occasion and it is okay to eat special foods that are a part of our culture, like samosas, pakoras, sev, gulabjaman and jilebi. But it is important to limit the amount of these foods we eat and to not overdo it. What are some ways to avoid overeating these special foods and avoid eating too much food overall?"

Make sure to summarize the participants' responses:

Key talking points:

- Eat only one sweet or only one fried food (Control your portion sizes)
- Eat something small like a fruit before leaving the house so that way you don't arrive hungry
- Drink water
- Eat more salad or more vegetables
- Practice saying no
- At a party, dance or socialize to avoid eating more food

Discuss eating meals throughout the day as a way to avoid overeating:

"Eating our meals throughout the day is another way to help control our weight. Why do you think this is?"

Summarize the participants' responses:

"Right, if we skip a meal or wait to eat one meal at the end of the day, we can become hungry and can overeat."

"Spreading out our meals throughout the day will also help us get the energy and nutrients that we need during the entire day."

Discuss healthy eating while fasting:

"Sometimes when people fast they lose weight, stay the same or even gain weight. What are some things you can do to avoid gaining weight while you are fasting?"

Summarize the participants' responses:

"Right, we can watch the amount of food that we eat and focus on having vegetables, fruits and whole grains. We can still have sweets and fried foods but we want to make sure we are not eating too many of them because eating too much can lead to weight gain."

Review of Current Session

Materials Needed:
Media equipment

- Laptop
- Projector

Media

- Session 5: Controlling Your Weight PowerPoint (see Appendix 5A for PowerPoint Slides)

Show the Session 5: Controlling Your Weight PowerPoint:

Slide 11: *"In the first activity, we learned that there are many weight loss diets."*

Slide 12: *"In activity two, we learned how to modify recipes to make them healthier for you and your family."*

Slide 13: *"In activity three, we learned about the components of a healthy plate and built a healthy plate."*

Slide 14: *"In the last activity, we discussed ways to avoid overeating when we eat out at restaurants and special events and ways to eat healthier to control our weight."*

Encourage the participants to try something knew that they learned in today's session:

"We would like for you to try something you learned in class today. We will talk about it at the beginning of next class."

CONFIDENTIAL- Do not distribute, developed by Northwestern University

Closing

Materials Needed:
Handouts for participants:

- Class Evaluations

Reminder for the next session:

> *"At the next session, we will be talking about how to take care of stress and tension. Please remember to continue wearing your pedometer, to record your steps in the worksheet and to bring it with you to the next class."*

Hand out Class Evaluations (one per participant):

> *"Before next session, we would like for everyone to fill out this class evaluation. This will help MAFS and Northwestern University improve these classes."*

Collect Questions from this session:

> *"Also we would like for everyone to write down a question on the back of the class evaluation. The questions should be about this session. **Please do not include any personal health issues**. We will be doing this at the end of each session because we want to make sure that everyone's questions get answered. We will collect this when you are finished and we will answer the questions before we start the next session."*

Thank the participants for attending and confirm the date and time for the next session.

Appendix 5 A: Session 4: Controlling Your Weight PowerPoint Slides

Slide 1
Session 4 Review (Activity 1: Understanding Different Types of Fat)

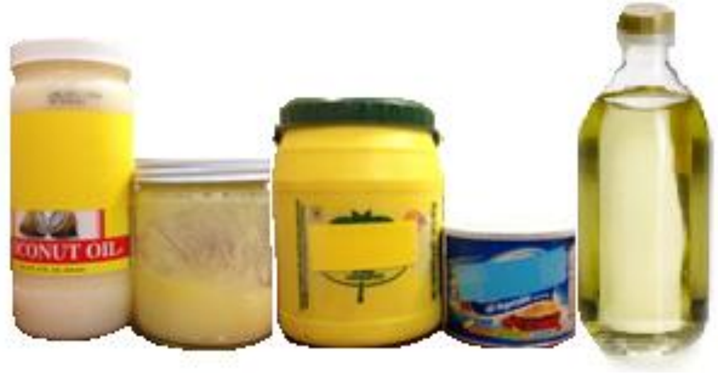

Slide 2
Session 4 Review (Activity 2: How Clogged Arteries are caused)

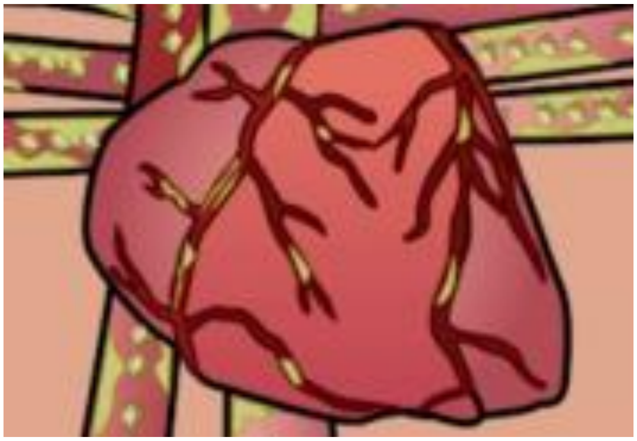

Slide 3

Session 4 Review (Activity 3: How to Read Food Labels)

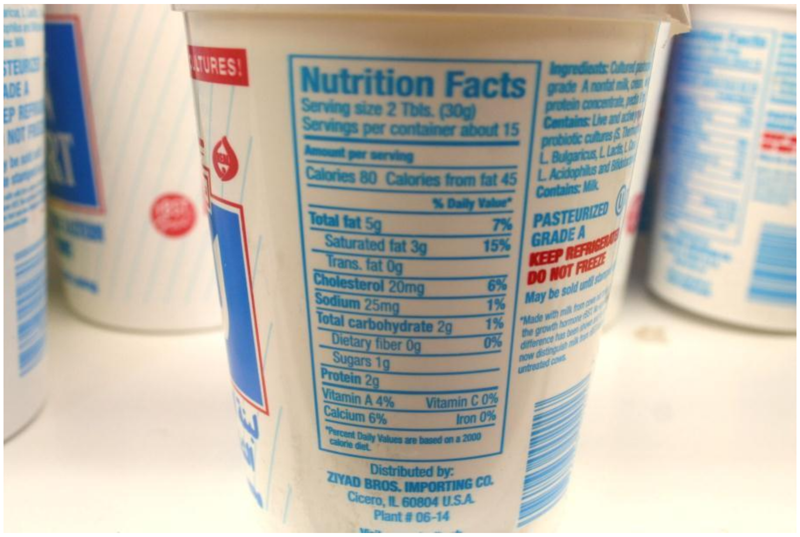

Slide 4
Session 4 Review (Activity 4: Grocery Store Tour)

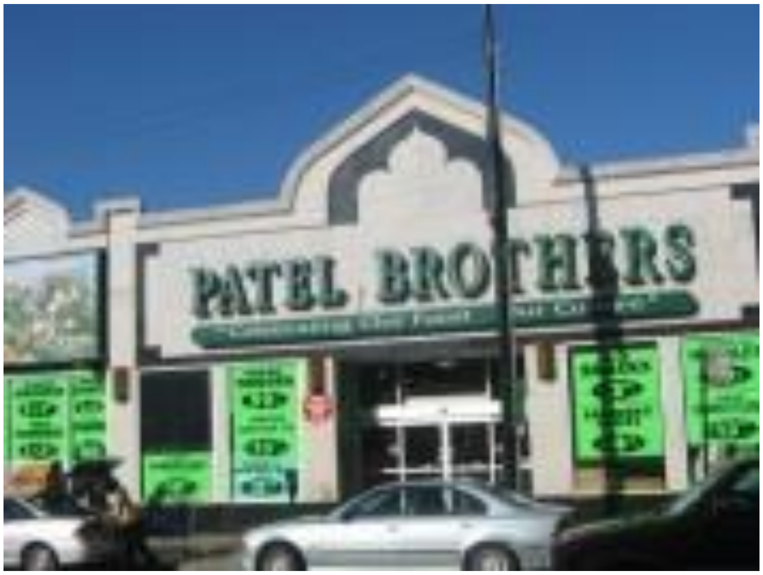

Slide 5
Palak Paneer Recipe

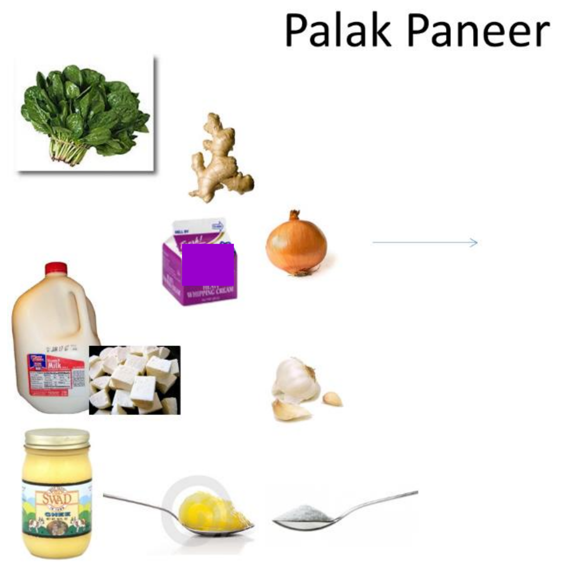

Slide 6
Modified Palak Paneer Recipe

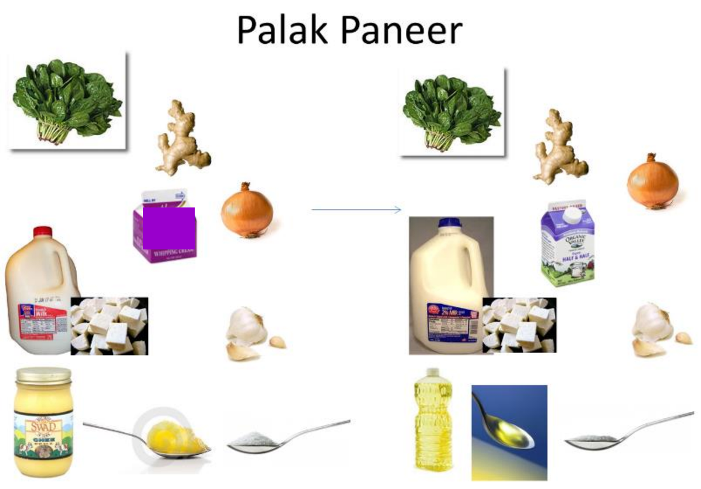

Slide 7
Upma Recipe

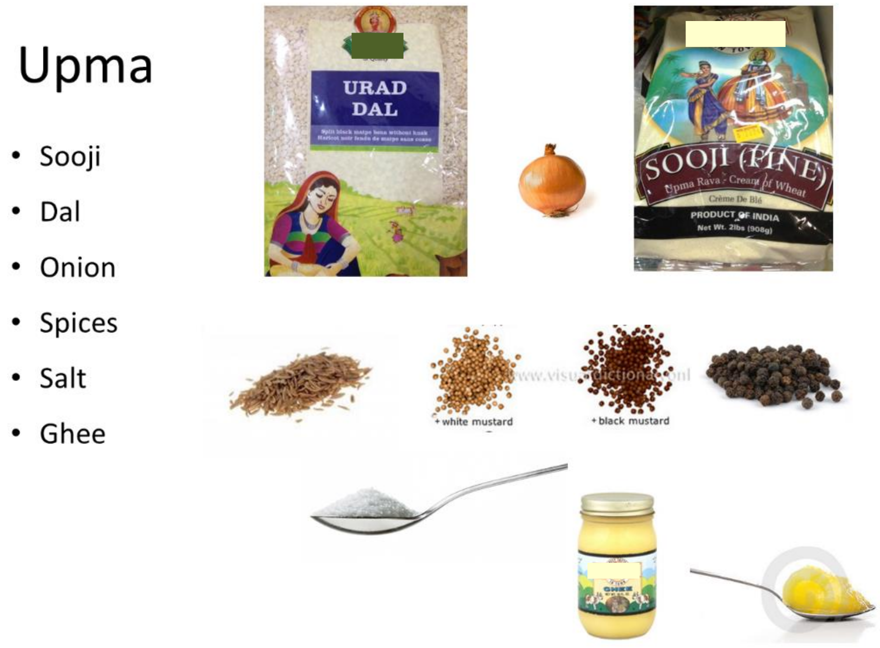

Slide 8
Kebab Recipe

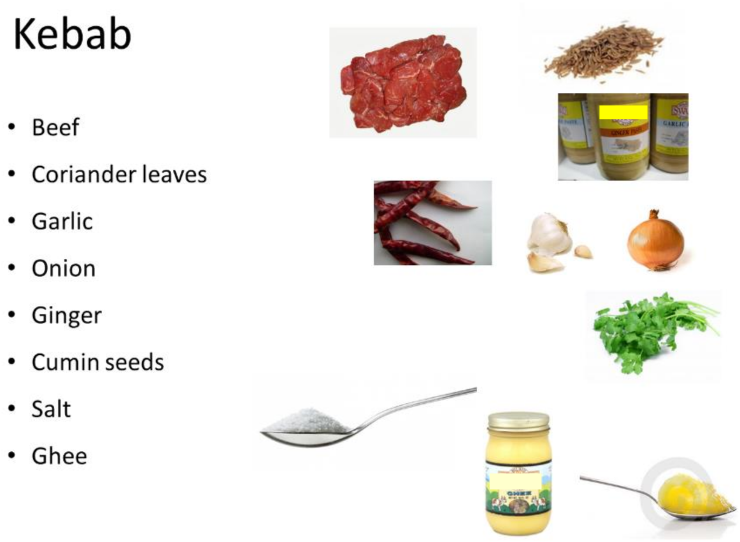

Slide 9
Modified Upma Recipe

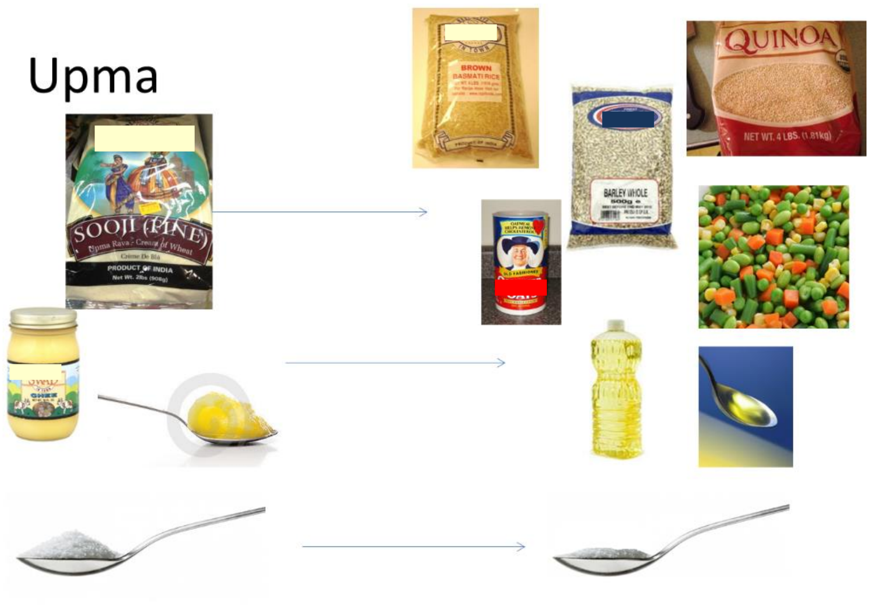

Slide 10
Modified Kebab Recipe

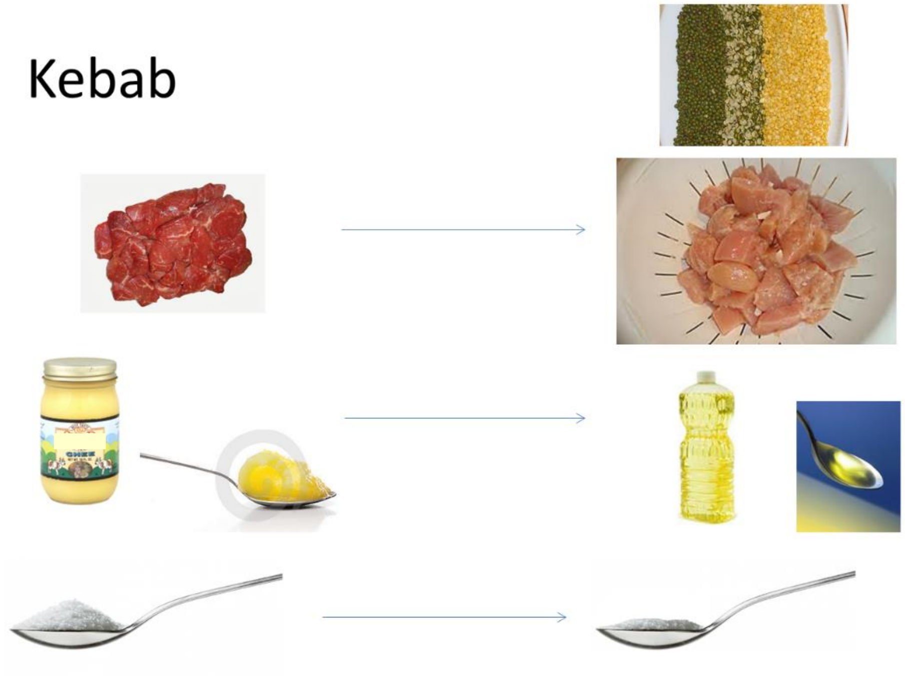

Slide 11
Session 5 Review (Activity 1: Weight Loss Diets Discussion)

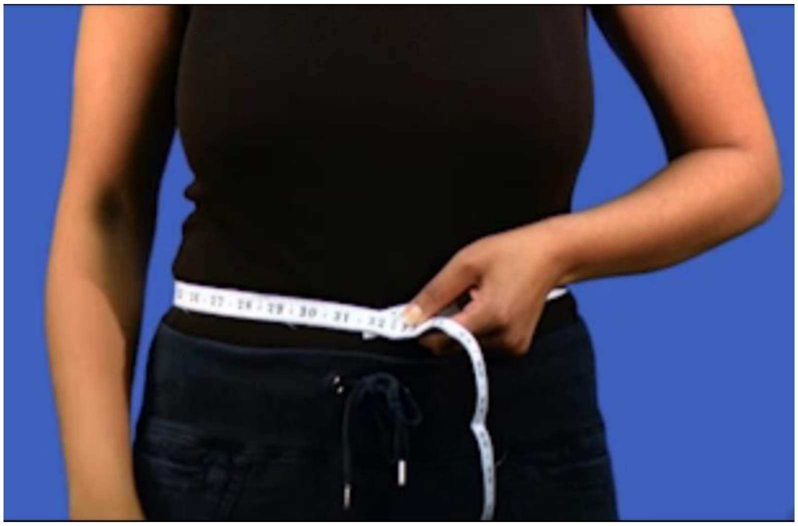

Slide 12
Session 5 Review (Activity 2: Recipe Modification)

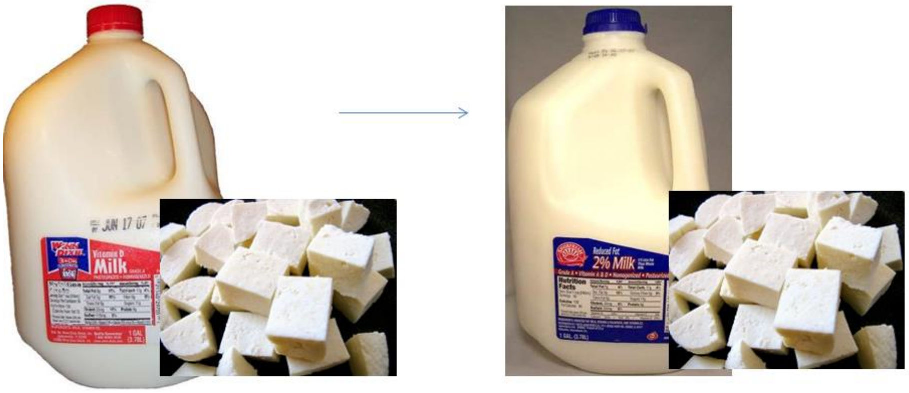

Slide 13
Session 5 Review (Activity 3: Building a Healthy Plate)

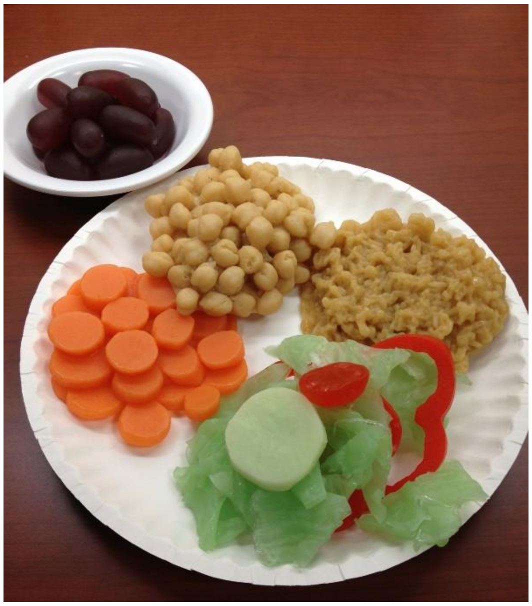

Slide 14
Session 5 Review (Activity 4: Ways to Avoid Overeating)

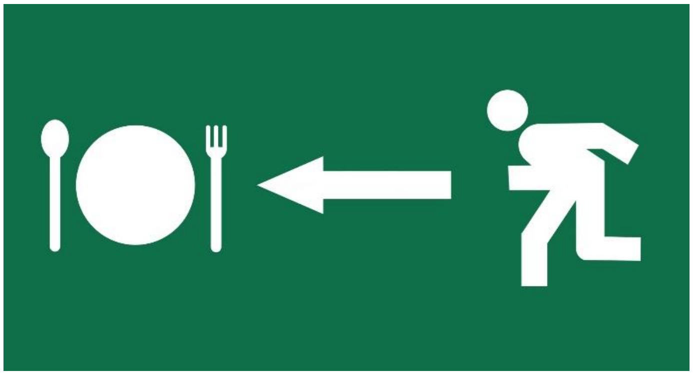

Appendix 5B: Plate Method

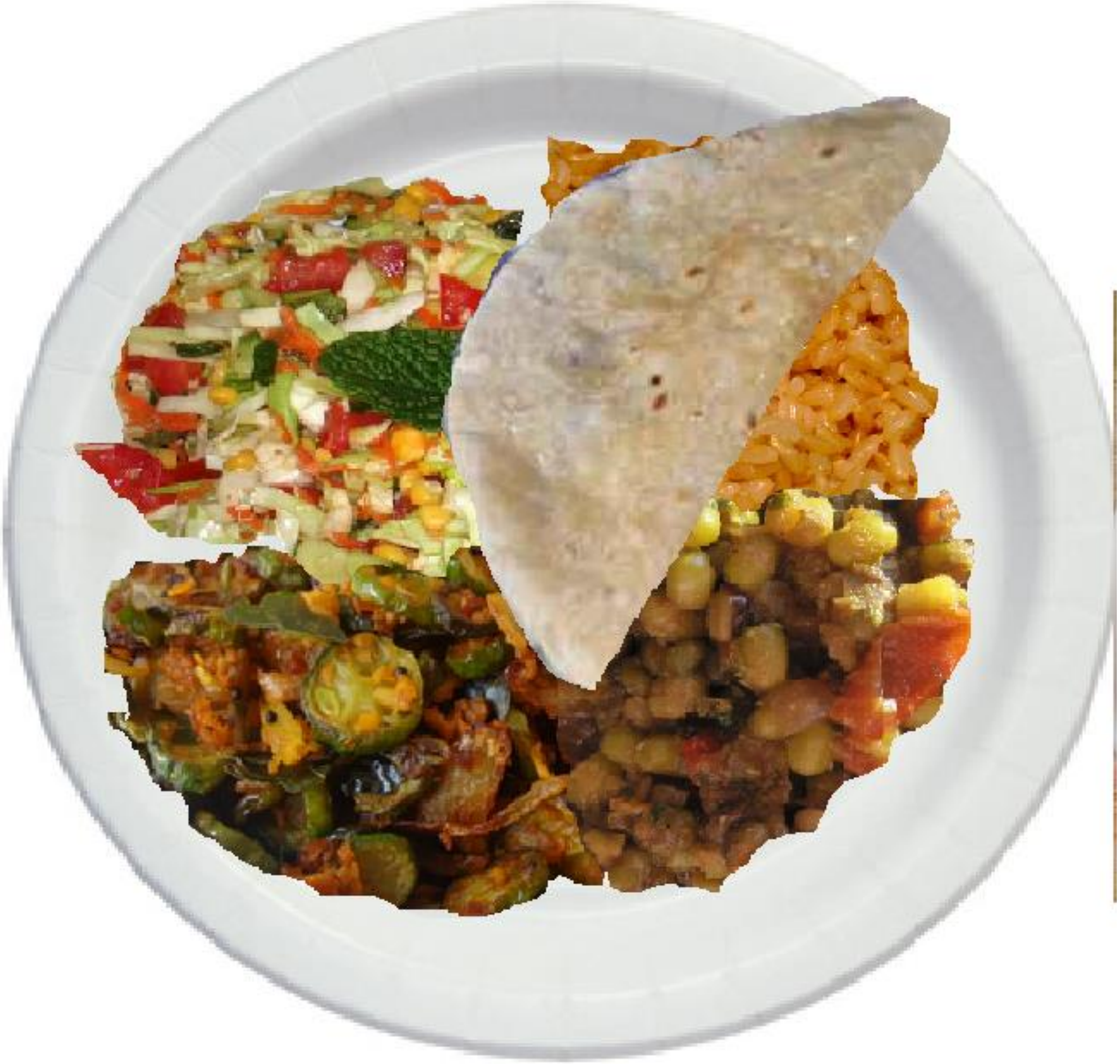

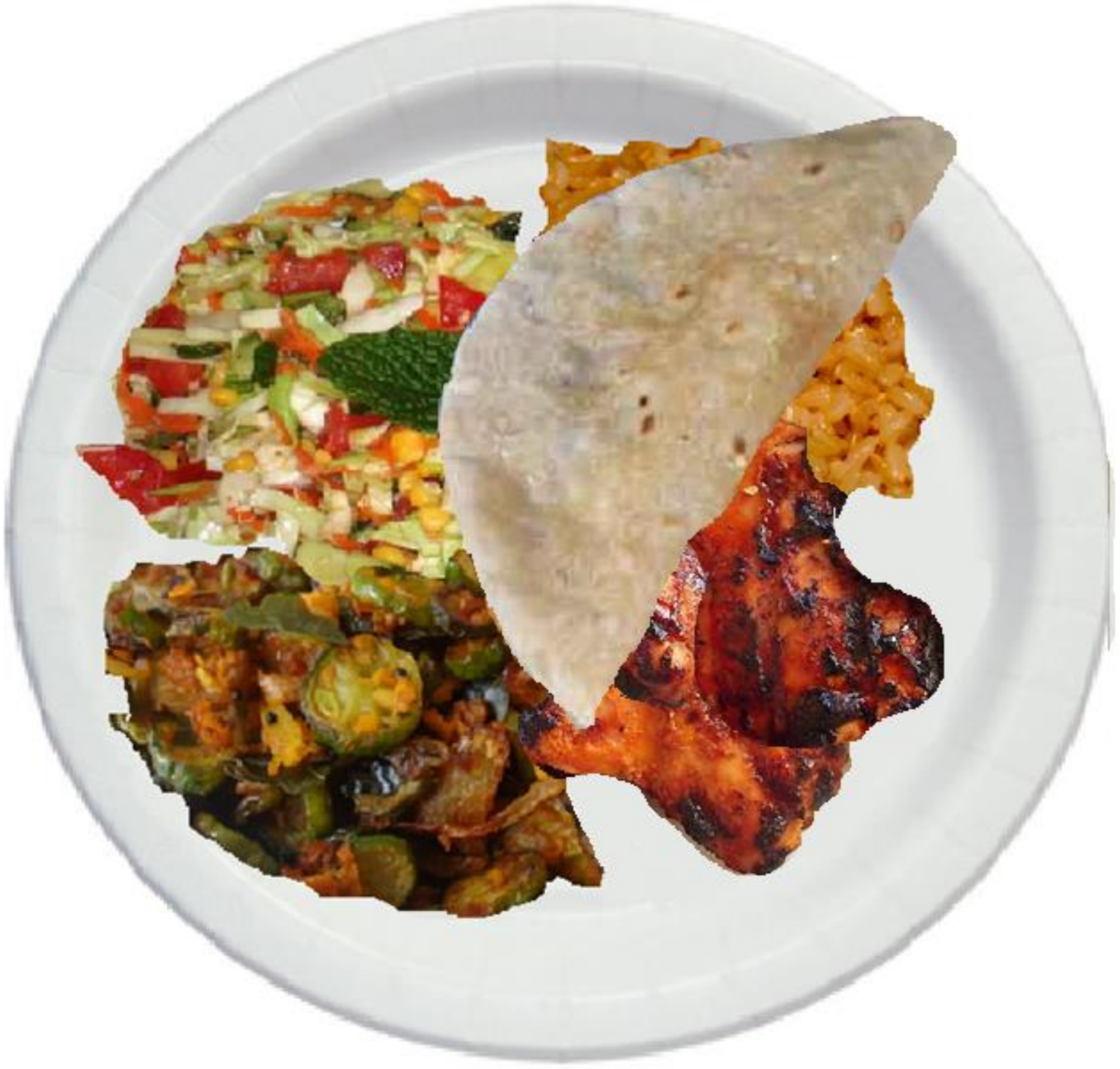

Session 6: Take Care of Your Stress and Tension

Purpose:

Participants will identify ways to cope with their stress.

Session length:

70 minutes

Overview of materials needed for the session

Media equipment
- Video recorder*
- Audio recorder*
- Laptop
- Projector
- Speakers

Media
- PowerPoint Session 5 Review
- "Take Care of Your Stress and Tension" Video
- PowerPoint 'Ways to Manage Stress'
- PowerPoint 'Scenario Pictures'
- Session 6 Review PowerPoint

Other supplies
- Yoga mats
- Song for meditation
- Certificate of Completion for each participant
- DVD of Northwestern University Heart Disease Prevention videos for each participant
- Pitcher of water and plastic cups

Handout(s) for participants
- Printed slides of PowerPoint 'Scenario Pictures' (Appendix 6A)
- 5 pages of the Pedometer Logs for each participant (Appendix 6B)
- Class Surveys

***Remember to start the video and audio recordings.** The video recorder should record one of the educators as they teach the session and an audio recorder should be used for each group.

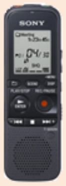

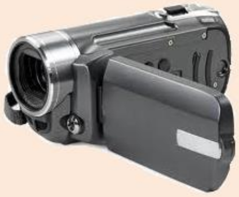

Introduction: 8 minutes

Materials needed:
Media equipment
- Laptop
- Projector

Media
- Session 5 Review PowerPoint

1. Review last session using PowerPoint Session 5 Review and ask if the participants if they tried anything new from last session.

    - *"Before we begin our session on taking care of our stress and tension, we are going to review what we learned last session."*

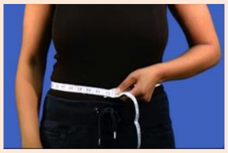

- Slide 1: *"Last week, we learned about the importance of controlling our weight. We also learned that there are many things that we hear about for losing weight but that the best way to lose weight is to reduce the portions of foods, use the plate method to make half your plate vegetables, and to use a pedometer to track how many steps you take to increase walking and exercising."*

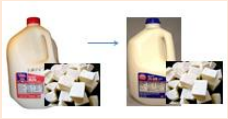

- Slide 2: *"We then talked about ways to modify some recipes that we cook at home to make them healthier for us and our family."*

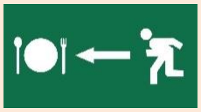

- Slide 3: *"We also discussed ways to avoid overeating when we go to parties or other special events and ways to eat healthier to control our weight."*

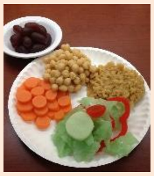

- Slide 4: *"Finally, we learned the most effective way to lose weight is to have a healthy plate with the right portions and limit the amount of fat, salt, sweets, and fried foods."*

    - *"Did anyone try something new that we learned last session?"*
    Provide affirmations to the participants that tried something from last session. Take the time to have the participants talk about barriers to trying something new and encourage the participants to problem solve and brainstorm other ways they can eat less fat and salt. Encourage the participants to try what they learned in class or to try other ways to eat less fat and salt.

2. Go through the responses to the questions asked last week

    - *"At the end of last class, we asked everyone to write down any questions that they had from that session. We will briefly review the responses to these questions."*

3. Review the participants' steps recorded. Provide affirmations to the participants that increased their steps. Take the time to have the participants review their goals and share with one another the facilitators and barriers to increasing their steps.

4. Introduce Session 6 topic.

    - *"In this session, we are going to talk about ways to take care of your stress and tension. Everything that we learn today will help improve our health."*

Watch video: "Take Care of Your Stress and Tension" 8 minutes

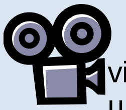

Video Objectives: After watching the 'Take Care of Your Stress and Tension' video the participant will be able to identify ways to manage stress and to understand the relationship between stress and heart disease.

Materials needed:
Media equipment
- Laptop
- Projector
- Speakers

Media
- "Take Care of Your Stress and Tension" video

1. Introduce the video

    - *"Our video today is going to talk about the relationship between stress and heart disease and talk about ways to manage stress. After the video, we will briefly discuss what we learned and then do activities that will help us better understand the information from the video."*

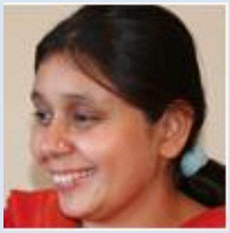

2. After the video, ask the participants to summarize the video and if they learned anything new from the video

    - *"Can someone tell me what the video talked about?"*
    - *"Did you learn anything new that you didn't know before?"*

Activity 1: Savasana (Corpse pose) or Viparita Karani (Legs-up-the-Wall Pose) 15 minutes

Objectives: The purpose of this activity is for the participant to practice Savasana (corpse pose) or viparita karani (legs up the wall pose) and recognize this is a relaxation technique that may be used to manage stress.

Materials needed:
Media equipment

- Laptop
- Speakers

Other Supplies

- Yoga mats
- Song for meditation

1. Introduce Viparita Karani

    - *"Before we get started on the activities, we want everyone to try a relaxation technique that they can use at home. The pose is called Viparita Karani (legs-up-the-wall pose) and this pose can help you calm your anxiety."*

2. Give instructions on how to get into the pose and relax in pose for 10 minutes while music is playing.

    - *"To get into the pose, sit next to a wall on a mat with the side of your body on the wall and your knees bent into the chest."*
    - *"Bring the lower back onto the floor while bringing the legs up the wall. Keep the upper body supported with the elbows on the floor."*
    - *"Slowly release the elbows and lower the whole back down to the floor."*
    - *"If you do not want to lift your legs, you can just lie flat on your back on the mat."*
    - *"We will stay in this position for 10 minutes. Close your eyes and relax."* Play song: Raag Sindhi Bhairavi (Flute) -by Pt. Hariprasad Chaurasia http://www.youtube.com/watch?v=erEiFOOBvxs&feature=youtu.be&noredirect=1

3. After 10 minutes, have the participants come out of the pose and ask how the 10 minutes of relaxation in this pose felt.

    - *"Slowly bring the knees into your chest and roll to your side to come out of the pose."*
    - *"How did it feel to take 10 minutes and relax in this pose?"*
    - *"This is just one way to manage stress. We are going to take about more ways to manage stress in our next activity."*

Activity 2: Identifying ways to take care of your stress 12 minutes

Objectives: The purpose of this activity is for the participant to review the different ways to manage stress presented in the video and to identify other ways that they might manage their stress.

Materials needed:
Media

- PowerPoint 'Ways to Manage Stress'

1. Acknowledge that stress happens but that there are many ways to manage stress.

   - *"The video mentioned several ways to handle stress. Let's review the many ways to take care of stress."*

2. Show first PowerPoint Slide of 'Ways to Manage Stress'

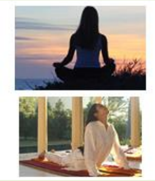

   - *"One way to take care of our stress is to use relaxation techniques, like the pose we just did, meditation and yoga. These relaxation techniques will help calm the tension in your mind and body."*

3. Show 2nd PowerPoint Slide of 'Ways to Manage Stress'

   - *"There are also things that you can do every day to help you manage your stress. What are some things that you can do to help you manage your stress?"*
   - *"What are some other things that you can do every day to help you manage your stress?"*
   - If the participants don't mention the following, make sure to emphasize them:
     - Eating healthy
     - Exercise
     - Get enough sleep
     - Talk to family and friends when feeling stressed
     - Ask for help from friends and family
     - Set an alarm to avoid the stress of being late
     - Plan ahead so you are prepared for an event
     - Spend time with family and friends playing games or going on walks

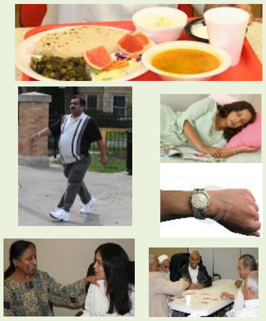

   - Other ideas that participants may have:
     - Do daily prayers
     - Read a book, magazine or newspaper
     - Sew, knit or crochet
     - Listen to music
     - Take a class to learn something new

4. Show 3rd PowerPoint Slide of 'Ways to Manage Stress'. Explain positive thinking as a way to manage stress and give the following two examples. (We encourage the educator to use examples that the participants can relate to)

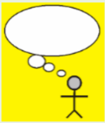

- *"Sometimes the way we think and the way we feel can increase our stress and tension. Negative thoughts can bring us down, especially if we always have them and keep them inside. Having positive thoughts can help us manage our stress.*
- *"For example, let's say your boss calls you and tells you that you did not do your job well. You are not supposed to do it like this but it is supposed to be done another way. How will that make you feel?"*
- After the participants respond to the question, emphasize thinking positively in this situation: *"Instead of thinking negatively, we can think about how to solve the problem; on how to be better. The boss took the time out of their busy schedule to tell you, so it must be important. Their feedback can help you improve. If the boss wanted to, they could have fired you, but they didn't do that. This is positive thinking which will help manage stress."*
- *"Let's say you are walking on Devon and say hi to someone you know but that person doesn't respond back. How would you feel?*
- After the participants respond to the question, emphasize thinking positively in this situation: *"We may think negatively and may think to ourselves, that the person doesn't like us. But perhaps the other person didn't see you or the other person was having a bad day and didn't want to be bothered."*
- *"Before we move on to the next activity does anyone have any questions?"*

Activity 3: Scenarios of stressful situations 12 minutes

Objectives: The purpose of this activity is for the participant to identify ways to manage stress for a scenario that deals with stressful situations.

Materials needed:
Media equipment
- Laptop
- Projector

Media
- PowerPoint 'Scenario Pictures'

Handout(s) for participants
- Printed slides of PowerPoint 'Scenario Pictures'

1. Introduce Activity 3

   - *"We would like for everyone to get into groups of four. Each group will be given a stressful situation and as a group, we would like for you to work together to come up with some ways that could help these people take care of their stress and tension."*
   - *"After your discussion, one person from each group will present their scenario to the rest of the class and a different person from each group will summarize their discussion."*

2. Handout the one PowerPoint slide of a scenario to each group and read their scenario example to each group.

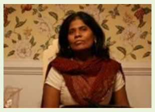

i. Usha recently emigrated from India to the U.S. with her husband and two children. Her husband works during the day and her children are in school. She feels sad, lonely, and tense because her family is back in India. She thinks to herself 'I can't do this; I want to go back to India.'

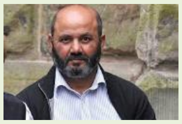

ii. Ali's mother lives with him. His mother needs more help now because she is getting older. Ali also works during the week. Ali has been getting headaches often and is worried about his mother's health. He thinks to herself 'I am so busy and I don't think I am doing a good job at taking care of my mother.' All of this is causing a lot of stress for him.

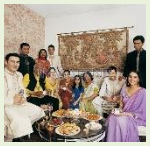

iii. Aditi has family visiting and has to help her sister plan her wedding. She is having some family stay with her and is excited but she feels overwhelmed. She thinks to herself 'I won't be able to take care of everyone. If I can't do this well, I'm not a good sister.'

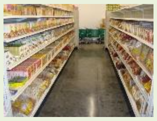

iv. Deepak is late for work. His supervisor asked him to not be late next time as they need him to open the store and there are people who are waiting for him. While at work, he makes a mistake at the cash register. He feels a lot of tension and pressure and is thinking negatively. He thinks to himself 'I should have not been late, now everything is going wrong.'

3. Give the participants 5-6 minutes to discuss the scenario as a group.

4. Have each group present their scenario and share their responses. Summarize their ideas.

    - Usha is facing a lot of hardships. She is new to this country and feels lonely because she doesn't have her family nearby. Some things she can do to help take care of her tension is to get to know other people. She can take an English class or other classes at community organizations like Indo-American Center to help her meet other people who are facing a similar situation, to help her relieve her stress and to learn something new. She can volunteer or get a job, too. She can go on walks, meditate, or do yoga to help take care of her stress. Instead of thinking 'I can't do this; I want to go back to India', she can think 'I will do the best that I can' or 'I will try but I can get help if I need it.'
    - Ali is also facing a lot of difficulties. It is important that if he is feeling worried and stressed that he gets help and support from friends and family. Most importantly, he needs to take care of himself so he is able to care for his mother. Things he can do every day are eating healthy and eating his meals on time, making sure he gets enough sleep, doing his daily prayers and meditating. Instead of thinking 'I am so busy and feel overwhelmed; everything is going wrong', he can think 'I can handle things if I take one step at a time.'
    - Aditi is dealing with a lot of things all at once. Sometimes it helps to plan ahead and make a list of things that need to be done and to start with a small task. Once you accomplish this task, it can help you feel better because you are closer to the end. Sometimes, we think we can do things by ourselves but it is important to ask family and friends for help if you feel busy and overwhelmed. It is important to remember that this is a happy occasion and to give thanks instead of being upset. It is also important to think about things you've been able to accomplish in the past. Instead of thinking 'I won't be able to get all of this done. If I can't do this, I'm not a good sister', she can think 'I've been able to get over tough situations before, I can do it again' or 'I will do the best that I can.'
    - Deepak is having a tough day at work. Deepak may need to take a five minute break to try a relaxation technique like meditating or deep breathing. Instead of thinking 'I should have not been late, now everything is going wrong', he can think 'I won't let this problem get me down'. It's important to think positively because the negative thoughts might get in the way of getting his work done.

5. Summarize positive thinking as a way to manage stress and ask the participants if they have any questions before moving on.

    - *"Thinking positively is one of the ways that we talked about to help you manage stress."*
    - *"Does anyone have any questions before we move on to our last activity?"*

Activity 4: Exercise as a way to manage stress 10 minutes

Objectives: The purpose of this activity is for the participant to go on a short walk outside and to recognize that exercise can be helpful in managing stress.

Materials needed:
Supplies
- Pitcher of water and cups for participants

1. Briefly introduce the importance of exercise as a way to manage stress and complete a short walk around the block.

    - *"Any type of exercise and physical activity will help us manage our stress. We are going to go for a short walk outside to show how exercise can reduce stress and lead to a more relaxed state of mind. Please get your coats and leaving everything else in the classroom. As you walk, think about how it makes you feel and think about making your walk a brisk walk."*

2. After the short walk, have the participants describe how they feel after the walk and summarize their response.

- *"How do you feel after going on a short walk?"*
- *"Is this something that you can do to help manage your stress?"*
- After the participants have a chance to respond, emphasize walking as a way to exercise and manage stress:
- *"Walking, even for 5-10 minutes, can help us feel more relaxed, can help us reflect and helps improve our health."*

CONFIDENTIAL- Do not distribute, developed by Northwestern University

Closing 5 minutes

Materials needed:
Media equipment
- Laptop
- Projector

Media
- Session 6 Review PowerPoint

Handout(s) for participants
- 5 Pedometer logs for each participant
- Class surveys

Other Supplies
- Folder with: Certificate of Completion
- DVD of videos

1. Summarize activities done in the session using PowerPoint Session 6.

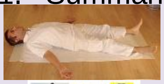
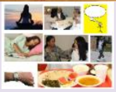
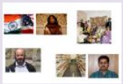
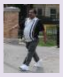

- *"This is the end of the session, so let's review what we have learned today."*
- Slide 1: *"Today, we practiced one relaxation technique, Viparita Karani, to help us manage our stress."*
- Slide 2: *"We then learned that there are many other ways to take care of our stress."*
- Slide 3: *"Then we got into groups and practiced identifying ways to manage stress."*
- Slide 4: *"Finally we went on a walk as exercise is another way to help take care of stress."*

2. Introduce telephone-support phone calls role-play to give an example of setting a goal.

- *"Think about the things you learned in these classes and the things that you would like to work on. As part of the program, we will be making six phone calls to everyone in the class to help you use the information that you learned in class in your day to day life. These phone calls will last about 10-15 minutes. We will work with you to come up with a goal that you want to do and that you can do to help you improve your health."*

3. Role-play to give an example of setting a goal.

- The following are examples of goals. Use one example and demonstrate setting a goal with one other person.
  - Eat a fruit between breakfast and lunch.
  - Instead of sitting down while watching TV, walk around or go up and down the stairs during commercials.
  - I will increase my steps from 5,000 a day to 7,000 a day in one month.
  - I will reduce the amount of oil for cooking by 1/3.
  - I will make half my plate vegetables at dinner.
  - I will take 5 minutes every day to meditate.
  - I will use my exercise bands and do the exercise band movements two times a week.

4. Let the participants know that they can decide to have weekly or biweekly phone calls for the first four phone calls.

    - *"Some participants thought phone calls every week may be too much so they asked if we could call every two weeks. Please let us know if you would like for us to call every week or every two weeks. The last two phone calls will be done once a month. Does anyone have any questions about the phone calls?"*

5. Present the Certificate of Completion and DVDs of videos and thank the participants for participating in the sessions.

    - *"This is the end of the session and we want to thank everyone for attending the sessions. We hope you learned a lot and that you can use what you learned in your everyday life."*
    - *"We would like to present everyone with a Certificate of Completion and a DVD of the videos we watched in class so you can have it at home to review and watch with your family."*

6. Hand out the Pedometer logs and class evaluations to the participants.

    - *"Before we leave, we would like for everyone to have a Pedometer log to continue tracking their steps."*
    - *"We would also like for everyone to fill it out the class evaluations. This will help us improve our classes."*
    - *"If anyone has any questions about today's class or other things that we talked about in the other sessions, please feel free to ask us."*

CONFIDENTIAL- Do not distribute, developed by Northwestern University

My Steps

	Week 1	Week 2	Week 3	Week 4
**Daily Goal**				
Sunday				
Monday				
Tuesday				
Wednesday				
Thursday				
Friday				
Saturday				

CONFIDENTIAL- Do not distribute, developed by Northwestern University

CHAPTER 14: 3 MONTH AND 6 MONTH ASSESSMENTS

OVERVIEW

The second visit will include several questionnaires and procedures (i.e., anthropometry, blood pressure measurement, fasting blood collections, etc). We estimate that the complete visit will require between 1 to 1.5 hours and must be completed in one day.

VISIT GUIDELINES

General

- Anthropometry and blood collection should be performed while the participant is fasting. (If participant is not fasting, record date and time he/she last ate or drank.) Blood pressure measurement should be done before venipuncture. Questionnaires do not require fasting.
- Blood drawing should be done after a minimum of a 10-hour fast and before 10:30am.

Examination Guidelines

Guidelines for second visit at 3 months are listed below:

- Seated blood pressure should be done immediately following the greeting and informed consent, and before venipuncture.
- Resting blood pressures should be obtained after the participant has been in the seated position for at least five minutes.
- Venipuncture should be performed in the fasting state after blood pressure measurement. If a participant comes to the clinic non-fasting, exam components that do not require fasting may be completed, and then schedule the participant for another visit for fasting blood collection.
- Provide a snack to participant after the blood draw.
- Questionnaires and other exam procedures may be administered after the fasting blood draw. During the interviews, make every effort to avoid distractions, ensure privacy, and maintain confidentiality for the participant. Do not conduct interviews during the lunch or in the waiting area in the clinic.

BEFORE THE VISIT

Visit 2 Forms

The coordinator should check the Daily Calendar showing clinic visits scheduled for the following day. The Calendar should list the preferred language of each person scheduled for a clinic visit, which will help determine how many sets of each form is needed for the day.

Print a set of the forms that will be completed for interviews and procedures. These will be pre-printed with participant IDs, which will be matched to an individual when he/she begins the exam. Forms can be printed somewhat in advance of upcoming visits, and it is advisable to have enough printed forms on hand for all the participants. Forms will be printed in English, with the Hindi translations available. For

each participant, gather all the forms required for a visit, and place into a folder labeled with his/her participant ID.

Supplies and Equipment

- Make sure the accelerometers are ready and completely charged before handing out to the participants. The instructions for the accelerometer should also be printed and laminated.
- Prepare the examination room for the anthropometry measurement, and seated BP.
- Check all instruments that will be used for the examination e.g. Amount estimation tools, phlebotomy supplies.

Staffing

- Prepare staff assignment sheet and make sure everyone knows his/her responsibilities.

Suggested 3 month visit schedule

Participant Arrives	9:00 am
Seated Blood Pressure	09:00 – 09:10 am
Fasting labs	09:10 - 09:15 am
Anthropometry	09:15 – 09:20 am
Snack	09:20 – 09:30 am
Questionnaires	09:30 – 09:50 am
24 hour food recall	09:50 – 10:25 am
Accelerometer usage instructions	10:25 – 10:30 am

Instructions to Participants Before the Visit

Mail the Pre-Visit Instructions to the participant 5–7 days before second visit and explain to them over the telephone when you schedule the visit. If possible, make a reminder call to the participant the day before second visit and reiterate the instructions. (If the participant is acutely ill—e.g. “flu” or bronchitis—when you make this reminder call, *tell him/her not to come to the clinic.* Arrange to contact him/her again to reschedule when he/she has recovered.) Before the examination, make sure the participants understand the following instructions.

6. Participants must fast for at least 10 hours before the examination. This restriction applies to all food and beverages (except water), including alcohol. Instruct them to consume dinner at least 10 hours before their scheduled appointment at the clinic. Only water and prescription medications are allowed from dinner until the start of the examination the next morning. Diabetic patients should *not* take their hypoglycemic medications the morning of the clinic visit; they should *bring the morning dose to the clinic* to be taken after venipuncture.

7. Participants should not smoke on the morning of the visit.

8. Participants should bring all current medications, both prescription and over-the-counter, including vitamin preparations and dietary supplements, to the clinic. If the participant forgets to bring the medications, schedule another visit to obtain this information or collect the information over phone.

9. Participants should bring the name and complete address of their personal physician or health plan, particularly if they wish to have examination results sent to that provider.

10. Participants should wear or bring loose-fitting clothes, preferably t-shirt, sweat pants, and slip-on shoes or sneakers.

VISIT RECEPTION

The reception process is very important in setting the participant's frame of mind for the rest of the exam day. Greet each participant warmly as soon as he/she arrives at the clinic. (If a participant arrives at the clinic acutely ill—e.g., "flu" or bronchitis—do not continue with the clinic examination. Make arrangements to contact him/her to reschedule the appointment after he/she has recovered.)

SEATED BLOOD PRESSURE

Purpose: Blood pressure (BP) level is a major risk factor for coronary heart disease, congestive heart failure, and stroke. Heart rate reflects autonomic nervous system function and cardiovascular fitness. The measured BP level is subject to biological and observer variability.

Only study staff that are trained and certified can take blood pressure measurements for the SAHELI study.

Procedure: Refer to Chapter 7, Blood Pressure for instructions.

PHLEBOTOMY

Purpose: To measure traditional (cholesterol, glucose, fasting sugar) risk factors for cardiovascular disease.

ANTHROPOMETRY

Purpose: To measure body weight, height, abdominal girth and hip girth. The measures of weight and height will be used to calculate body mass index; waist and hip circumferences are other measures of body composition that have been linked to cardiovascular disease risk.

Procedure: Refer to Chapter 6, Anthropometry

QUESTIONNAIRES

Purpose: At Visit 2, participants will be asked to complete 6 interviewer-conducted questionnaires that are listed below.

INTERVIEWER-ADMINISTERED QUESTIONNAIRES TO BE ADMINISTERED IN THE ORDER BELOW:

24 HOUR FOOD RECALL

MEDICATION QUESTIONNAIRE AND INVENTORY

PHYSICAL ACTIVITY
HEART DISEASE QUESTIONS
EXERCISE CONFIDENCE SURVEY
COPE SURVEY

24 Hour Food Recall

Purpose: The 24 Hour Food Recall will record the participants' food and beverage intake of the previous day.

Procedure: Refer to Chapter 9, 24 Hour Food Recall

MEDICATIONS QUESTIONNAIRE AND MEDICATIONS INVENTORY

Purpose: At the second visit, participants will be asked to bring all medications that they are currently taking to the clinic, including prescription medications, over-the-counter (OTC) medications, vitamins, herbs and dietary supplements.

Procedure: Follow the directions on the data forms.

Filling out the MEDICATIONS QUESTIONNAIRE

Question 1:

- All prescription medications, over-the-counter (OTC) medications, vitamins, herbs and dietary supplements will be recorded on the MEDICATIONS INVENTORY FORM.
- ***If participant has brought in some, but not all of their medications***, record them on the MEDICATIONS INVENTORY FORM and complete the form when the participant brings in or call you with the remaining medications.
- ***If participant is currently not taking any medications*** do not complete a MEDICATIONS INVENTORY FORM.

Filling out the MEDICATIONS INVENTORY

On each completed MEDICATION INVENTORY, indicate the page number of each form by filling in the corresponding bubble. (For the first form, fill in the “1” bubble, for the second form, fill in the “2” bubble, etc.) The ***total*** number of forms will be recorded on the MEDICATIONS QUESTIONNAIRE.

Participant ID #	Acrostic	Date of Interview	Staff ID #
		Mo / Day / Year	

SAHELI MEDICATION INVENTORY

Visit: Baseline 3 month 6 month

Page ○ **1** ○ **2** ○ **3** ○ **4** ○ **5**

3. Record the trade name (or generic name, if necessary). If the name of the medication is longer than the number of data entry boxes on the form, just fill out as much as possible.

4. Indicate the frequency the medication is taken.

1. Name:

Frequency ○ Daily ○ Weekly ○ Monthly ○ As needed

SPECIAL INSTRUCTIONS

Issue	Resolution
Participant took a medication 1 week ago but stopped taking it	• Record the medication
Participant has not brought in all of his/her medications or has forgotten to bring all of them	• Record the medications the Participant has with him/her and ask the participant to call you later with the name of the other medication(s).
Participant is unable to list or name all of his/her herbal remedies and supplements	• Ask the participant why s/he is taking the medication

Physical Activity

Purpose: The Physical Activity questionnaire will measure the level of physical activity across different activity categories (household chores, gardening, dancing, exercise etc) and different intensities (light, moderate, and heavy).

Participant ID #	Acrostic	Date of Interview	Staff ID #
		Mo / Day / Year	

MEDICATION QUESTIONNAIRE

SAHELI BASELINE VISIT

PHYSICAL ACTIVITY

Coordinator Instructions: Please use a black pen and fill in bubbles completely.

Think about the types of activities you did in a typical week in the past month. For each activity, note which of these activities you did in a typical week by filling in the circle for YES or NO. For each item you mark as YES, fill in the circle for the number of DAYS in a typical week you did these activities and the AVERAGE TIME per day in hours <u>and/or</u> minutes you did these activities.

Intensity Levels:
Light → easy effort
Moderate → harder than light but not all-out effort
Heavy → all-out effort

Example:
Conditioning Activities
<u>Moderate Effort:</u>
Low impact aerobics, slow bicycling, rowing, leisurely swimming, health club machines - moderate intensity

Y N	Days/Week	Hours/Day	Minutes/Day
Y N<br>● ○	1 2 3 4 5 6 7<br>○ ○ ● ○ ○ ○ ○	1 2 3 4 5 5+<br>● ○ ○ ○ ○ ○	5 15 30 45<br>○ ○ ● ○

In this example, the activity was done 3 days per week, 1 hour and 30 minutes per day.

HEART DISEASE QUESTIONS

Purpose: This is designed to understand the existing knowledge and perceptions of heart disease among South Asians. Perceptions are measured using a scale ranging from Strongly agree to Strongly disagree. Knowledge is measured based on the response to questions on heart disease prevention and risk factors for heart disease.

Participant ID #	Acrostic	Date of Interview	Staff ID #
		Mo / Day / Year	

SAHELI HEART DISEASE QUESTIONS

Visit: ○ Baseline   3 Month   6 Month

Coordinator Instructions: Read the question and answers (except for #10 on page 4, write the response given). Circle or fill in the answer given by the participant.
Read these instructions to the participant: The following are some questions about heart disease.

Perceptions

	Strongly agree	*Agree*	*Neither agree nor disagree*	*Disagree*	*Strongly disagree*
1. Asian Indians and Pakistanis are more likely to have heart attacks compared to people from other communities	1	2	3	4	5
2. Regular exercise can reduce my chances of having a heart attack	1	2	3	4	5
3. An older person does not need to exercise	1	2	3	4	5
4. If your heart starts to beat fast while you are exercising, you should stop.	1	2	3	4	5
5. Men need more exercise than women	1	2	3	4	5
6. Eating fruits, vegetables and whole grains will help prevent a heart attack	1	2	3	4	5
7. Eating less fat and salt will help prevent a heart attack	1	2	3	4	5
8. Being overweight increases my chances of having a heart attack	1	2	3	4	5

CONFIDENTIAL- Do not distribute, developed by Northwestern University

Participant ID #	Acrostic	Date of Interview	Staff ID #
		Mo / Day / Year	

SAHELI HEART DISEASE QUESTIONS

Visit: ○ Baseline   3 Month   6 Month

Knowledge

1. True or False: A person who has high blood sugar or diabetes is more likely to have a heart attack.
2. Major risk factors for heart disease and stroke include:
    1. High cholesterol
    2. Chewing or smoking tobacco (e.g. cigarettes, gutkha, bidi)
    3. Family history of heart disease
    4. All of the above
3. True or False: Everyone's body makes cholesterol.
4. A person with high blood pressure:
    1. Has high cholesterol
    2. Has a higher risk of stroke and heart attack
    3. Has a nervous condition
    4. Has a fast heart beat
5. Which blood pressure level is too high?
    1. 130/80
    2. 140/90
    3. 150/85
    4. Both b & c
6. How much exercise is recommended to keep your heart healthy?
    1. 20 minutes every day
    2. 90 minutes a day once you are in shape
    3. 30 minutes everyday
    4. It depends on your age
7. True or False: Medium or fast exercise keeps your heart strong and prevents a heart attack
8. Which of these people should exercise regularly? SHOW PARTICIPANT PAGE 5

1. 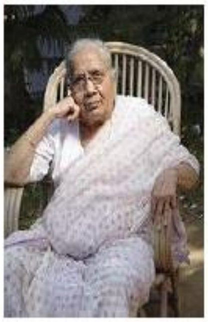
2. 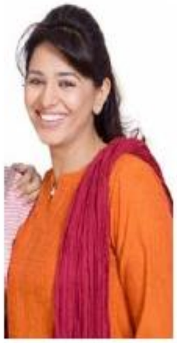
3. 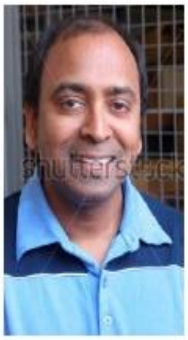
4. All of the above

EXERCISE CONFIDENCE SURVEY

Purpose: Will help understand how sure the participants feel that they will be able to follow the exercise routine.

Participant ID #	Acrostic	Date of Interview	Staff ID #
☐☐☐☐☐	☐☐☐☐	☐☐/☐☐/☐☐☐☐	☐☐☐

SAHELI EXERCISE CONFIDENCE SURVEY

VISIT: BASELINE 3 MONTH 6 MONTH

Below is a list of things people might do while trying to increase or continue regular exercise. We are interested in exercises like running, swimming, brisk walking, bicycle riding, aerobics classes or yoga.

Whether you exercise or not, please rate how confident you are that you could really motivate yourself to do things like these consistently, for at least six months.

Please circle one number for each question.

How sure are you that you can do these things?

	I know I can		*Maybe I can*		*I know I cannot*	*Does not apply*
1. Get up early, even on weekends, to exercise.	1	2	3	4	5	(8)
2. Stick to your exercise program after a long, tiring day at work.	1	2	3	4	5	(8)
3. Exercise even though you are feeling depressed	1	2	3	4	5	(8)
4. Set aside time for a physical activity program; that is, walking, jogging, swimming, biking, or other continuous activities for at least 30 minutes, 3 times per week.	1	2	3	4	5	(8)
5. Find a friend, family member, or group to exercise with.	1	2	3	4	5	(8)
6. Stick to your exercise program when your family is demanding more time from you.	1	2	3	4	5	(8)
7. Stick to your exercise program when you have household chores to attend to.	1	2	3	4	5	(8)

COPE SURVEY

PURPOSE :

The COPE survey helps identify the processes that people use in coping with stressful situations. In this study it will help us understand how the South Asian community copes with stressful encounters of everyday living. In a variety of studies, researchers have used it to investigate the components and determinants of coping.

Participant ID #	Acrostic	Date of Interview	Staff ID #
☐☐☐☐☐	☐☐☐☐	☐☐/☐☐/☐☐☐☐	☐☐☐

SAHELI STUDY

COPE QUESTIONNAIRE

Baseline 3 Month 6 Month

Introduction: There are lots of ways to try to deal with stress. Indicate what you generally do and feel, when you experience stressful events. Please circle the correct response.

	All of the time	*Most of the time*	*Sometimes*	*Rarely*	*Never*	*Does not apply*
1. I try to get advice from someone about what to do.	1	2	3	4	5	(8)
2. I put my trust in God.	1	2	3	4	5	(8)
3. I make a plan of action	1	2	3	4	5	(8)
4. I just give up trying to reach my goal	1	2	3	4	5	(8)
5. I try to come up with a strategy about what to do.	1	2	3	4	5	(8)
6. I look for something good in what is happening.	1	2	3	4	5	(8)
7. I do something I enjoy to take my mind off the stress.	1	2	3	4	5	(8)
8. I ask people who have had similar experiences what they did.	1	2	3	4	5	(8)
9. I try to find comfort in my religion.	1	2	3	4	5	(8)
10. I talk to someone about how I feel.	1	2	3	4	5	(8)
11. I do what has to be done, one step at a time.	1	2	3	4	5	(8)

Accelerometer instructions and handouts

After the completion of the questionnaires the participants will be given instructions to use the accelerometer. The accelerometers will be loaned and an appointment will be scheduled for the return of the accelerometer.

Participants will be informed that they will receive their test results when they come after one week to return the accelerometer.

See Chapter 8 for accelerometer instructions

REPORTING OF TEST RESULTS WHEN PARTICIPANTS COME TO RETURN THE ACCELEROMETER

Giving Participants Results of Visit Measurements and Lab Values

The participant will be given anthropometry and blood pressure results when they come to return their accelerometers, a week after the 3 month visit.
The blood test from quest and physical exam results will be ready in an envelope when they are scheduled to come for their appointment for returning their accelerometer.

ALERTS AND ABNORMAL RESULTS

The purpose of defining medical alerts is to make sure that the participant and his/her physician are aware of any significant medical findings that arise as a result of the SAHELI exam.

Definitions

Alert: Any of the medical findings, listed in Table 1 that may have adverse health consequences to the participant if untreated.

Immediate Referrals: Medical emergencies which require immediate notification of both the participant and his/her primary physician. Participants receiving immediate referrals should be considered as those who would go directly from the Clinical Site to their physician or hospital. Immediate notification of the participant should occur during the clinic visit. Immediate notification of the participant’s physician should be accomplished by telephone, to be completed before the participant leaves the clinic. A follow-up letter documenting information discussed by phone should also be sent to the participant’s physician for findings included in the initial report, or immediately upon receipt from the central agency for findings on the later reports.

Urgent Referrals: Urgent referrals are made for abnormalities detected which require medical attention but not on an emergency basis. Urgent notification of the participant should occur before the participant leaves the clinic (for findings included in the initial participant report), or immediately upon receipt from the central laboratory (for findings on later reports). Urgent notification of the participant’s physician should be sent within the week.

Table 1. SAHELI Alerts and Alert Levels

Finding	Alert Level
Systolic BP > 210	Immediate
Diastolic BP > 120	Immediate
180 < Systolic BP < 211	Urgent
110 < Diastolic BP < 121	Urgent
Total cholesterol > 360 mg/dL	Urgent
Triglyceride > 1000 mg/dL	Urgent
HDL cholesterol < 20 mg/dL	Urgent
Calculated LDL cholesterol > 260 mg/dL	Urgent
Fasting glucose < 50 mg/dL or > 400 mg/dL	Urgent

Methods

General Instructions
Whenever one of the alerts listed above is identified for a participant, the actions defined under Immediate or Urgent Referrals, above, must be completed. Actions taken must be documented with a copy of the letter to the participant archived in the participant file.

Specific Instructions for Urgent or Immediate Referrals

3.1 If an “Urgent” or “Immediate” Referral result occurs, the Study Coordinator should notify the NU PI as soon as possible.
3.2 The NU PI should telephone the participant within 24 hours and instruct the participant to contact their primary care provider for follow-up immediately.
3.3 The NU PI should document the date and time of the telephone call in the participant’s file.
3.4 A letter and a copy of the test should be mailed to the participant. (Appendix 3C)

If any other actions were taken or unusual circumstances were involved, please document this in the participant file.

“Other” alerts: If any unusual incidents occur in the clinic, please document this in the participant file. These should include anything that would be important to have documented later, including a participant fall in the clinic, participant feeling dizzy or fainting during the blood draw, etc. If in doubt, document!

3 MONTH VISIT CHECKLIST

Purpose: The 3 MONTH VISIT CHECKLIST provides an outline of all procedures that should be completed during the Visit. After completion of each component of 3 MONTH VISIT, staff will complete the 3 MONTH VISIT CHECKLIST.

The 3 MONTH VISIT CHECKLIST gives a suggested order of procedures for the visit. . It is highly recommended that the interviewer follow the order of the checklist for the remainder of the visit.

Procedure: Complete and check for accuracy and missing information.

Filling out the form:

- Indicate participant status at the top of this form. If the interviewer is unable to contact the candidate to complete Visit 2, the interviewer must use his/her judgment to decide the appropriate time to indicate participant status as "Visit 2 scheduled but did not show."

- If a participant completes the visit, make sure all procedures on the checklist were marked "Yes." *Do not leave any fields blank.*

- If the participant becomes ineligible at any point during the visit, check off which procedures were fully or partially done by marking "Yes." Mark "No, other" for the rest of the incomplete procedures. *Do not leave any fields blank.*

Participant ID #	Acrostic	Date of Interview	Staff ID #
☐☐☐☐☐	☐☐☐☐	☐☐/☐☐/☐☐☐☐<br>Mo Day Year	☐☐☐

SAHELI VISIT CHECKLIST
BASELINE 3 MONTH 6 MONTH

Coordinator Instructions: For checklist items, mark only one bubble per line. *Do not leave any lines blank.* Items in **bold** are teleforms therefore all pages must be faxed in if procedure was marked as done.

Participant Status from Visit 1:

O Visit completed
O Visit not completed, specify reason ______________________________
O Visit scheduled but did not show

PARTICIPANT ID# ☐☐☐☐☐☐

**Item**	**Please mark if done** Yes	No	Not applicable	**Comments**
**PRIOR TO THIS VISIT**				
1. Print forms with Participant ID & Acrostic	O	O	O	
**AT THE VISIT**				
2. Review and sign Informed Consent	O	O	O	
3. Review and sign HIPAA	O	O	O	
4. Review Participant Contact Information	O	O	O	
5. Demographics	O	O	O	
6. Urine pregnancy test (if applicable)	O	O	O	
7. Medication Questionnaire	O	O	O	
8. Medication Inventory Form(s) (if applicable)	O	O	O	
9. Seated Blood Pressure	O	O	O	
10. Urine/Phlebotomy	O	O	O	
11. Fasting Blood Draw	O	O	O	Time: ___ : ___ AM/PM*<br>Enter draw time on the Quest Lab Form.

APPENDIX 12A: APPOINTMENT LETTER

South Asian Heart Disease Prevention Study
Instructions for the visit

CLINIC APPOINTMENT REMINDER
Appointment Date: 01/07/2010
Time: 8:15 am

Begin Fasting Date: 01/08/2010
Time: 8:15 pm
(10 hrs before appointment)

Date:

Dear Mr/Ms.__________,

Thank you very much for agreeing to participate in the "Translating a heart disease lifestyle intervention into the community" study. You visit is scheduled at Metropolitan Asian Family Services on ____________, **Date** ________ at **time** _____. Directions from your residence to the facility are attached.

Below are some items to keep in mind prior to your visit:

1. You will have a fasting blood test during your visit. **Do not eat anything after 10 p.m. the day before your visit.** You will not be eligible for blood test if you do not fast.
2. If you take diabetes medicine, **do not take your diabetes medicine the morning of your visit.**
3. Take all other medicine.
4. We ask that you **bring all current medications to the visit.**
5. If you have a doctor and you want your results sent to your doctor, bring your doctors information.
6. Wear comfortable cloths.
7. If you need glasses for reading, please bring them with you.
8. If you are ill, please call us at (312) 503-6995 to reschedule your visit.

CONFIDENTIAL- Do not distribute, developed by Northwestern University

APPENDIX 14B : EXAMINATION RESULTS FORM

The South Asian Heart Disease Prevention Study

Physical Exam and Blood test Results

Date:________ ID # ____________________

Dear ___________________

We appreciate your participation in The South Asian Heart Lifestyle Intervention Study. The purpose of this letter is to provide you with some of the results of your examination.

HEIGHT: __________**feet**________**inches**

WEIGHT: _________**pounds**

BODY MASS INDEX: ___________**kg/m²**

A body mass index of 25 kg/m^2 or greater is considered overweight, and 30 kg/m^2 or greater is considered obese by the National Institutes of Health.

WAIST:___________**inches**

Normal waist measurement for men < 35 inches

Normal waist measurement for women < 31 inches

BLOOD PRESSURE: ___________**mmHg**

These values indicate that your blood pressure is:

- ☐ Normal (SBP < 120 and DBP < 80)
- ☐ Borderline Elevated (SBP 120-139 or DBP 80-90)
- ☐ Elevated (SBP > 140 or DBP > 90)

BLOOD TEST RESULTS: (See Quest lab results enclosed)

Some of your blood test results that were out of normal range were:

1. xxx
2. xxx

We recommend that you discuss any abnormal results with your doctor.

Sincerely,

Namratha R. Kandula

Namratha Kandula, MD, MPH
Principal Investigator
Northwestern University

6 MONTH ASSESSMENT

OVERVIEW

The third visit will include several questionnaires and procedures (i.e., anthropometry, blood pressure measurement, fasting blood collections, etc). We estimate that the complete visit will require between 1 to 1.5 hours and must be completed in one day.

VISIT GUIDELINES

General

- Anthropometry and blood collection should be performed while the participant is fasting. (If participant is not fasting, record date and time he/she last ate or drank.) Blood pressure measurement should be done before venipuncture. Questionnaires do not require fasting.
- Blood drawing should be done after a minimum of a 10-hour fast and before 10:30am.

Examination Guidelines

Guidelines for the third visit at 6 months are listed below:

- Seated blood pressure should be done immediately following the greeting and informed consent, and before venipuncture.
- Resting blood pressures should be obtained after the participant has been in the seated position for at least five minutes.
- Venipuncture should be performed in the fasting state after blood pressure measurement. If a participant comes to the clinic non-fasting, exam components that do not require fasting may be completed, and then schedule the participant for another visit for fasting blood collection.
- Provide a snack to participant after the blood draw.
- Questionnaires and other exam procedures may be administered after the fasting blood draw. During the interviews, make every effort to avoid distractions, ensure privacy, and maintain confidentiality for the participant. Do not conduct interviews during the lunch or in the waiting area in the clinic.

BEFORE THE VISIT

Visit 3 Forms

The coordinator should check the Daily Calendar showing clinic visits scheduled for the following day. The Calendar should list the preferred language of each person scheduled for a clinic visit, which will help determine how many sets of each form is needed for the day.

Print a set of the forms that will be completed for interviews and procedures. These will be pre-printed with participant IDs, which will be matched to an individual when he/she begins the exam. Forms can be printed somewhat in advance of upcoming visits, and it is advisable to have enough printed forms on hand for all the participants. Forms will be printed in English, with the Hindi translations available. For each participant, gather all the forms required for a visit, and place into a folder labeled with his/her participant ID.

Supplies and Equipment

- Make sure the accelerometers are ready and completely charged before handing out to the participants. The instructions for the accelerometer should also be printed and laminated.
- Prepare the examination room for the anthropometry measurement, and seated BP.
- Check all instruments that will be used for the examination e.g. Amount estimation tools, phlebotomy supplies.

Staffing

- Prepare staff assignment sheet and make sure everyone knows his/her responsibilities.

Suggested 6 month visit schedule

Participant Arrives	9:00 am
Seated Blood Pressure	09:00 – 09:10 am
Fasting labs	09:10 - 09:15 am
Anthropometry	09:15 – 09:20 am
Snack	09:20 – 09:30 am
Questionnaires	09:30 – 09:50 am
24 hour food recall	09:50 – 10:25 am
Accelerometer usage instructions	10:25 – 10:3011.30 pm

Instructions to Participants Before the Visit

Mail the Pre-Visit Instructions (Appendix 13A) to the participant 5–7 days before 6 month visit and explain to them over the telephone when you schedule the visit. If possible, make a reminder call to the participant the day before 6 month visit and reiterate the instructions. (If the participant is acutely ill—e.g. “flu” or bronchitis—when you make this reminder call, *tell him/her not to come to the clinic.* Arrange to contact him/her again to reschedule when he/she has recovered.) Before the examination, make sure the participants understand the following instructions.

Participants must fast for at least 10 hours before the examination. This restriction applies to all food and beverages (except water), including alcohol. Instruct them to consume dinner at least 10 hours before their scheduled appointment at the clinic. Only water and prescription medications are allowed from dinner until the start of the examination the next morning. Diabetic patients should *not* take their hypoglycemic medications the morning of the clinic visit; they should *bring the morning dose to the clinic* to be taken after venipuncture.

Participants should not smoke on the morning of the visit.

Participants should bring all current medications, both prescription and over-the-counter, including vitamin preparations and dietary supplements, to the clinic. If the participant forgets to bring the medications, schedule another visit to obtain this information or collect the information over phone.

Participants should bring the name and complete address of their personal physician or health plan, particularly if they wish to have examination results sent to that provider.

Participants should wear or bring loose-fitting clothes, preferably t-shirt, sweat pants, and slip-on shoes or sneakers.

VISIT RECEPTION

The reception process is very important in setting the participant's frame of mind for the rest of the exam day. Greet each participant warmly as soon as he/she arrives at the clinic. (If a participant arrives at the clinic acutely ill—e.g., "flu" or bronchitis—do not continue with the clinic examination. Make arrangements to contact him/her to reschedule the appointment after he/she has recovered.)

.

SEATED BLOOD PRESSURE

Purpose: Blood pressure (BP) level is a major risk factor for coronary heart disease, congestive heart failure, and stroke. Heart rate reflects autonomic nervous system function and cardiovascular fitness. The measured BP level is subject to biological and observer variability.

Only study staff that are trained and certified can take blood pressure measurements for the SAHELI study.

Procedure: Refer to Chapter 8, Blood Pressure for instructions.

13.6 PHLEBOTOMY

Purpose: To measure traditional (cholesterol, glucose, fasting sugar) risk factors for cardiovascular disease.

ANTHROPOMETRY

Purpose: To measure body weight, height, abdominal girth and hip girth. The measures of weight and height will be used to calculate body mass index; waist and hip circumferences are other measures of body composition that have been linked to cardiovascular disease risk.

Procedure: Refer to Chapter 6, Anthropometry

13.8 QUESTIONNAIRES

Purpose: At Visit 3, participants will be asked to complete 7 interviewer-conducted questionnaires that are listed below.

INTERVIEWER-ADMINISTERED QUESTIONNAIRES TO BE ADMINISTERED IN THE ORDER BELOW:

24 HOUR FOOD RECALL
MEDICATION QUESTIONNAIRE AND INVENTORY
PHYSICAL ACTIVITY

HEART DISEASE QUESTIONS
SOCIAL NETWORK SURVEY
EXERCISE CONFIDENCE SURVEY
COPE SURVEY

13.8.1 24 Hour Food Recall

Purpose: The 24 Hour Food Recall will record the participants' food and beverage intake of the previous day.

Procedure: Refer to Chapter 9, 24 Hour Food Recall

13.8.2 MEDICATIONS QUESTIONNAIRE AND MEDICATIONS INVENTORY

Purpose: At the secondvisit , participants will be asked to bring all medications that they are currently taking to the clinic, including prescription medications, over-the-counter (OTC) medications, vitamins, herbs and dietary supplements.

Procedure: Follow the directions on the data forms.

Filling out the MEDICATIONS QUESTIONNAIRE

Question 1:

- All prescription medications, over-the-counter (OTC) medications, vitamins, herbs and dietary supplements will be recorded on the MEDICATIONS INVENTORY FORM.
- ***If participant has brought in some, but not all of their medications***, record them on the MEDICATIONS INVENTORY FORM and complete the form when the participant brings in or call you with the remaining medications.
- ***If participant is currently not taking any medications*** do not complete a MEDICATIONS INVENTORY FORM.

Filling out the MEDICATIONS INVENTORY

On each completed MEDICATION INVENTORY, indicate the page number of each form by filling in the corresponding bubble. (For the first form, fill in the "1" bubble, for the second form, fill in the "2" bubble, etc.) The ***total*** number of forms will be recorded on the MEDICATIONS QUESTIONNAIRE.

Participant ID #	Acrostic	Date of Interview	Staff ID #
		Mo / Day / Year	

SAHELI MEDICATION INVENTORY

Visit: Baseline 3 month 6 month

Page ○ **1** ○ **2** ○ **3** ○ **4** ○ **5**

5. Record the trade name (or generic name, if necessary). If the name of the medication is longer than the number of data entry boxes on the form, just fill out as much as possible.

6. Indicate the frequency the medication is taken.

1. Name:

Frequency ○ Daily ○ Weekly ○ Monthly ○ As needed

SPECIAL INSTRUCTIONS

Issue	Resolution
Participant took a medication 1 week ago but stopped taking it	• Record the medication
Participant has not brought in all of his/her medications or has forgotten to bring all of them	• Record the medications the Participant has with him/her and ask the participant to call you later with the name of the other medication(s).
Participant is unable to list or name all of his/her herbal remedies and supplements	• Ask the participant why s/he is taking the medication

CONFIDENTIAL- Do not distribute, developed by Northwestern University

Physical Activity

Purpose: The Physical Activity questionnaire will measure the level of physical activity across different activity categories (household chores, gardening, dancing, exercise etc) and different intensities (light, moderate, and heavy).

Participant ID #	Acrostic	Date of Interview (Mo / Day / Year)	Staff ID #

MEDICATION QUESTIONNAIRE

SAHELI BASELINE VISIT

PHYSICAL ACTIVITY

Coordinator Instructions: Please use a black pen and fill in bubbles completely.

Think about the types of activities you did in a typical week in the past month. For each activity, note which of these activities you did in a typical week by filling in the circle for YES or NO. For each item you mark as YES, fill in the circle for the number of DAYS in a typical week you did these activities and the AVERAGE TIME per day in hours and/or minutes you did these activities.

Intensity Levels:
Light → easy effort
Moderate → harder than light but not all-out effort
Heavy → all-out effort

Example:
Conditioning Activities
Moderate Effort::
Low impact aerobics, slow bicycling, rowing, leisurely swimming, health club machines - moderate intensity

Y	N	Days/Week	Hours/Day	Minutes/Day
●	○	1 ○ 2 ○ 3 ● 4 ○ 5 ○ 6 ○ 7 ○	1 ● 2 ○ 3 ○ 4 ○ 5 ○ 5+ ○	5 ○ 15 ○ 30 ● 45 ○

In this example, the activity was done 3 days per week, 1 hour and 30 minutes per day.

HEART DISEASE QUESTIONS

Purpose: This is designed to understand the existing knowledge and perceptions of heart disease among South Asians. Perceptions are measured using a scale ranging from Strongly agree to Strongly disagree. Knowledge is measured based on the response to questions on heart disease prevention and risk factors for heart disease.

Participant ID #	Acrostic	Date of Interview	Staff ID #
		Mo / Day / Year	

SAHELI HEART DISEASE QUESTIONS

Visit: ○ Baseline 3 Month 6 Month

Coordinator Instructions: Read the question and answers (except for #10 on page 4, write the response given). Circle or fill in the answer given by the participant.
Read these instructions to the participant: The following are some questions about heart disease.

Perceptions

	Strongly agree	*Agree*	*Neither agree nor disagree*	*Disagree*	*Strongly disagree*
1. Asian Indians and Pakistanis are more likely to have heart attacks compared to people from other communities	1	2	3	4	5
2. Regular exercise can reduce my chances of having a heart attack	1	2	3	4	5
3. An older person does not need to exercise	1	2	3	4	5
4. If your heart starts to beat fast while you are exercising, you should stop.	1	2	3	4	5
5. Men need more exercise than women	1	2	3	4	5
6. Eating fruits, vegetables and whole grains will help prevent a heart attack	1	2	3	4	5
7. Eating less fat and salt will help prevent a heart attack	1	2	3	4	5
8. Being overweight increases my chances of having a heart attack	1	2	3	4	5

Participant ID #	Acrostic	Date of Interview	Staff ID #
		__ / __ / ____ Mo Day Year	

SAHELI HEART DISEASE QUESTIONS

Visit: ○ Baseline 3 Month 6 Month

Knowledge

1. True or False: A person who has high blood sugar or diabetes is more likely to have a heart attack.
2. Major risk factors for heart disease and stroke include:
    1. High cholesterol
    2. Chewing or smoking tobacco (e.g. cigarettes, gutkha, bidi)
    3. Family history of heart disease
    4. All of the above
3. True or False: Everyone's body makes cholesterol.
4. A person with high blood pressure:
    1. Has high cholesterol
    2. Has a higher risk of stroke and heart attack
    3. Has a nervous condition
    4. Has a fast heart beat
5. Which blood pressure level is too high?
    1. 130/80
    2. 140/90
    3. 150/85
    4. Both b & c
6. How much exercise is recommended to keep your heart healthy?
    1. 20 minutes every day
    2. 90 minutes a day once you are in shape
    3. 30 minutes everyday
    4. It depends on your age
7. True or False: Medium or fast exercise keeps your heart strong and prevents a heart attack
8. Which of these people should exercise regularly? SHOW PARTICIPANT PAGE 5

1. 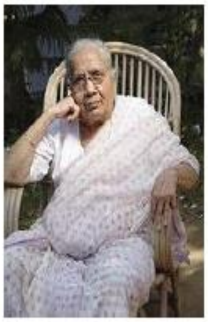
2. 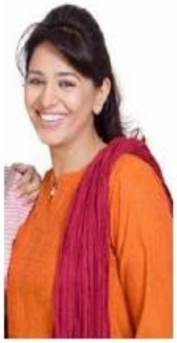
3. 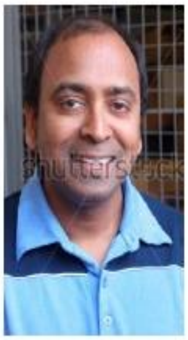
4. All of the above

SOCIAL NETWORK SURVEY

SEE CHAPTER 10 FOR DETAILS

Participant ID #	Acrostic	Date of Interview	Staff ID #
☐☐☐☐☐	☐☐☐☐	☐☐/☐☐/☐☐☐☐<br>Mo Day Year	☐☐☐

SAHELI SOCIAL NETWORK

Visit: Baseline 3 month 6 month

SAHELI-Net: Social and cultural influences on dietary patterns and physical activity

Overview

The purpose of this interview is to better understand how social relationships affect beliefs, attitudes and behaviors, and how these relationships affect physical and mental health. We will ask about your own and your social network members' health attitudes, beliefs, and behaviors.

As a reminder, your participation in this study is completely voluntary. You may decide to skip questions you prefer not to answer or end the survey at any time. Your decision to participate or not will in no way affect your future treatment or participation in research at this facility. We are very grateful for your assistance with this project.

We hope you will answer as many questions as you can, because your answers will help us to understand how social relationships influence health-related behaviors. This information will be used to design specific programs to directly improve community health.

Do you have any questions before we get started? Is it okay to begin?

Introduction:

"Now we are going to ask you some questions about your relationships with other people. We will begin by identifying some of the people you interact with on a regular basis. You do not have to give me the names of these people, simply their initials."

"From time to time, most people discuss things that are important to them with others. For example, these may include good or bad things that happen to you, problems you are having, or important concerns you may have. Looking back over the last 12 months, who are the people with whom you most often discussed things that were important to you? (**Prompt if do not know:** This could be a person you tend to talk to about things that are important to you. This can include your family, friends, relatives, neighbors, co-workers or anyone else you think you can discuss important matters about yourself.")

Roster A: **Initials of Persons:**

1.	5.	7.	9.
2.	6.	8.	10.

(**Prompt:** "Is there anyone else that you discuss important matters about yourself?")

EXERCISE CONFIDENCE SURVEY

Purpose: Will help understand how sure the participants feel that they will be able to follow the exercise routine.

Participant ID #	Acrostic	Date of Interview	Staff ID #
		/ /	

SAHELI EXERCISE CONFIDENCE SURVEY

VISIT: BASELINE 3 MONTH 6 MONTH

Below is a list of things people might do while trying to increase or continue regular exercise. We are interested in exercises like running, swimming, brisk walking, bicycle riding, aerobics classes or yoga.

Whether you exercise or not, please rate how confident you are that you could really motivate yourself to do things like these consistently, for at least six months.

Please circle one number for each question.

How sure are you that you can do these things?

	I know I can		*Maybe I can*		*I know I cannot*	*Does not apply*
1. Get up early, even on weekends, to exercise.	1	2	3	4	5	(8)
2. Stick to your exercise program after a long, tiring day at work.	1	2	3	4	5	(8)
3. Exercise even though you are feeling depressed	1	2	3	4	5	(8)
4. Set aside time for a physical activity program; that is, walking, jogging, swimming, biking, or other continuous activities for at least 30 minutes, 3 times per week.	1	2	3	4	5	(8)
5. Find a friend, family member, or group to exercise with.	1	2	3	4	5	(8)
6. Stick to your exercise program when your family is demanding more time from you.	1	2	3	4	5	(8)
7. Stick to your exercise program when you have household chores to attend to.	1	2	3	4	5	(8)

COPE SURVEY

PURPOSE :

Participant ID #	Acrostic	Date of Interview	Staff ID #
☐☐☐☐☐	☐☐☐☐	☐☐/☐☐/☐☐☐☐	☐☐☐

SAHELI STUDY
COPE QUESTIONNAIRE

Baseline 3 Month 6 Month

Introduction: There are lots of ways to try to deal with stress. Indicate what you generally do and feel, when you experience stressful events. Please circle the correct response.

	All of the time	*Most of the time*	*Sometimes*	*Rarely*	*Never*	*Does not apply*
1. I try to get advice from someone about what to do.	1	2	3	4	5	(8)
2. I put my trust in God.	1	2	3	4	5	(8)
3. I make a plan of action	1	2	3	4	5	(8)
4. I just give up trying to reach my goal	1	2	3	4	5	(8)
5. I try to come up with a strategy about what to do.	1	2	3	4	5	(8)
6. I look for something good in what is happening.	1	2	3	4	5	(8)
7. I do something I enjoy to take my mind off the stress.	1	2	3	4	5	(8)
8. I ask people who have had similar experiences what they did.	1	2	3	4	5	(8)
9. I try to find comfort in my religion.	1	2	3	4	5	(8)
10. I talk to someone about how I feel.	1	2	3	4	5	(8)
11. I do what has to be done, one step at a time.	1	2	3	4	5	(8)

The COPE survey helps identify the processes that people use in coping with stressful situations. In this study it will help us understand how the South Asian community copes with stressful encounters of everyday living. In a variety of studies, researchers have used it to investigate the components and determinants of coping.

Accelerometer instructions and handouts

After the completion of the questionnaires the participants will be given instructions to use the accelerometer. The accelerometers will be loaned and an appointment will be scheduled for the return of the accelerometer.

Participants will be informed that they will receive their test results when they come after one week to return the accelerometer.

See Chapter 8 for accelerometer instructions

REPORTING OF TEST RESULTS WHEN PARTICIPANTS COME TO RETURN THE ACCELEROMETER

Giving Participants Results of Visit Measurements and Lab Values

The participant will be given anthropometry and blood pressure results when they come to return their accelerometers, a week after the 6 month visit. See Appendix 12B
The blood test from quest and physical exam results will be ready in an envelope when they are scheduled to come for their appointment for returning their accelerometer.

ALERTS AND ABNORMAL RESULTS

The purpose of defining medical alerts is to make sure that the participant and his/her physician are aware of any significant medical findings that arise as a result of the SAHELI exam.

Definitions

Alert: Any of the medical findings, listed in Table 1 that may have adverse health consequences to the participant if untreated.

Immediate Referrals: Medical emergencies which require immediate notification of both the participant and his/her primary physician. Participants receiving immediate referrals should be considered as those who would go directly from the Clinical Site to their physician or hospital. Immediate notification of the participant should occur during the clinic visit. Immediate notification of the participant's physician should be accomplished by telephone, to be completed before the participant leaves the clinic. A follow-up letter documenting information discussed by phone should also be sent to the participant's physician for findings included in the initial report, or immediately upon receipt from the central agency for findings on the later reports.

Urgent Referrals: Urgent referrals are made for abnormalities detected which require medical attention but not on an emergency basis. Urgent notification of the participant should occur before the participant leaves the clinic (for findings included in the initial participant report), or immediately upon receipt from the central laboratory (for findings on later reports). Urgent notification of the participant's physician should be sent within the week.

Table 1. SAHELI Alerts and Alert Levels

Finding	Alert Level
Systolic BP > 210	Immediate
Diastolic BP > 120	Immediate
180 < Systolic BP < 211	Urgent
110 < Diastolic BP < 121	Urgent
Total cholesterol > 360 mg/dL	Urgent
Triglyceride > 1000 mg/dL	Urgent
HDL cholesterol < 20 mg/dL	Urgent
Calculated LDL cholesterol > 260 mg/dL	Urgent
Fasting glucose < 50 mg/dL or > 400 mg/dL	Urgent

Methods

General Instructions
Whenever one of the alerts listed above is identified for a participant, the actions defined under Immediate or Urgent Referrals, above, must be completed. Actions taken must be documented with a copy of the letter to the participant archived in the participant file.

Specific Instructions for Urgent or Immediate Referrals

If an "Urgent" or "Immediate" Referral result occurs, the Study Coordinator should notify the NU PI as soon as possible.
The NU PI should telephone the participant within 24 hours and instruct the participant to contact their primary care provider for follow-up immediately.
The NU PI should document the date and time of the telephone call in the participant's file.
A letter and a copy of the test should be mailed to the participant. (Appendix 3C)

If any other actions were taken or unusual circumstances were involved, please document this in the participant file.

"Other" alerts: If any unusual incidents occur in the clinic, please document this in the participant file. These should include anything that would be important to have documented later, including a participant fall in the clinic, participant feeling dizzy or fainting during the blood draw, etc. If in doubt, document!

CONFIDENTIAL- Do not distribute, developed by Northwestern University

Purpose: The 6 MONTH VISIT CHECKLIST provides an outline of all procedures that should be completed during the Visit. After completion of each component of 6 MONTH VISIT, staff will complete the 6 MONTH VISIT CHECKLIST.

The 6 MONTH VISIT CHECKLIST gives a suggested order of procedures for the visit. . It is highly recommended that the interviewer follow the order of the checklist for the remainder of the visit.

Procedure: Complete and check for accuracy and missing information.

Filling out the form:

- Indicate participant status at the top of this form. If the interviewer is unable to contact the candidate to complete Visit 3, the interviewer must use his/her judgment to decide the appropriate time to indicate participant status as "Visit 3 scheduled but did not show."

- If a participant completes the visit, make sure all procedures on the checklist were marked "Yes." *Do not leave any fields blank.*

- If the participant becomes ineligible at any point during the visit, check off which procedures were fully or partially done by marking "Yes." Mark "No, other" for the rest of the incomplete procedures. *Do not leave any fields blank.*

Participant ID #	Acrostic	Date of Interview	Staff ID #
☐☐☐☐☐	☐☐☐☐	☐☐/☐☐/☐☐☐☐ Mo Day Year	☐☐☐

SAHELI VISIT CHECKLIST
BASELINE 3 MONTH 6 MONTH

Coordinator Instructions: For checklist items, mark only one bubble per line. *Do not leave any lines blank.* Items in **bold** are teleforms therefore all pages must be faxed in if procedure was marked as done.

Participant Status from Visit 1:
O Visit completed
O Visit not completed, specify reason ____________________
O Visit scheduled but did not show

PARTICIPANT ID# ☐☐☐☐☐☐

Item	Please mark if done			Comments
	Yes	No	Not applicable	
**PRIOR TO THIS VISIT**				
1. Print forms with Participant ID & Acrostic	O	O	O	
**AT THE VISIT**				
2. Review and sign Informed Consent	O	O	O	
3. Review and sign HIPAA	O	O	O	
4. Review Participant Contact Information	O	O	O	
5. Demographics	O	O	O	
6. Urine pregnancy test (if applicable)	O	O	O	
7. Medication Questionnaire	O	O	O	
8. Medication Inventory Form(s) (if applicable)	O	O	O	
9. Seated Blood Pressure	O	O	O	
10. Urine/Phlebotomy	O	O	O	
11. Fasting Blood Draw	O	O	O	Time: ___ : ___ AM/PM*<br>Enter draw time on the Quest Lab Form.

APPENDIX 14A: APPOINTMENT LETTER

South Asian Heart Disease Prevention Study
Instructions for the visit

CLINIC APPOINTMENT REMINDER
Appointment Date: 01/07/2010
Time: 8:15 am

Begin Fasting Date: 01/08/2010
Time: 8:15 pm
(10 hrs before appointment)

Date:

Dear Mr/Ms.__________,

Thank you very much for agreeing to participate in the "Translating a heart disease lifestyle intervention into the community" study. You visit is scheduled at Metropolitan Asian Family Services on ____________, **Date** ________ at **time** _____. Directions from your residence to the facility are attached.

Below are some items to keep in mind prior to your visit:

1. You will have a fasting blood test during your visit. **Do not eat anything after 10 p.m. the day before your visit.** You will not be eligible for blood test if you do not fast.
2. If you take diabetes medicine, **do not take your diabetes medicine the morning of your visit.**
3. Take all other medicine.
4. We ask that you **bring all current medications to the visit.**
5. If you have a doctor and you want your results sent to your doctor, bring your doctors information.
6. Wear comfortable cloths.
7. If you need glasses for reading, please bring them with you.
8. If you are ill, please call us at (312) 503-6995 to reschedule your visit.

CONFIDENTIAL- Do not distribute, developed by Northwestern University

APPENDIX 14B : EXAMINATION RESULTS FORM

The South Asian Heart Disease Prevention Study

Physical Exam and Blood test Results

Date:________ ID # ____________________

Dear ___________________

We appreciate your participation in The South Asian Heart Lifestyle Intervention Study. The purpose of this letter is to provide you with some of the results of your examination.

HEIGHT: __________**feet**________**inches**

WEIGHT: _________**pounds**

BODY MASS INDEX: __________**kg/m^2**
A body mass index of 25 kg/m^2 or greater is considered overweight, and 30 kg/m^2 or greater is considered obese by the National Institutes of Health.

WAIST:___________inches
Normal waist measurement for men < 35 inches
Normal waist measurement for women < 31 inches

BLOOD PRESSURE: __________**mmHg**

These values indicate that your blood pressure is:
□ Normal (SBP < 120 and DBP < 80)
□ Borderline Elevated (SBP 120-139 or DBP 80-90)
□ Elevated (SBP > 140 or DBP > 90)

BLOOD TEST RESULTS: (See Quest lab results enclosed)
Some of your blood test results that were out of normal range were:

1. xxx
2. xxx

We recommend that you discuss any abnormal results with your doctor.
Sincerely,

Namratha R. Kandula

Namratha Kandula, MD, MPH
Principal Investigator
Northwestern University

OVERVIEW

The third visit will include several questionnaires and procedures (i.e., anthropometry, blood pressure measurement, fasting blood collections, etc). We estimate that the complete visit will require between 1 to 1.5 hours and must be completed in one day.

VISIT GUIDELINES

General

- Anthropometry and blood collection should be performed while the participant is fasting. (If participant is not fasting, record date and time he/she last ate or drank.) Blood pressure measurement should be done before venipuncture. Questionnaires do not require fasting.
- Blood drawing should be done after a minimum of a 10-hour fast and before 10:30am.

Examination Guidelines

Guidelines for the third visit at 6 months are listed below:

- Seated blood pressure should be done immediately following the greeting and informed consent, and before venipuncture.
- Resting blood pressures should be obtained after the participant has been in the seated position for at least five minutes.
- Venipuncture should be performed in the fasting state after blood pressure measurement. If a participant comes to the clinic non-fasting, exam components that do not require fasting may be completed, and then schedule the participant for another visit for fasting blood collection.
- Provide a snack to participant after the blood draw.
- Questionnaires and other exam procedures may be administered after the fasting blood draw. During the interviews, make every effort to avoid distractions, ensure privacy, and maintain confidentiality for the participant. Do not conduct interviews during the lunch or in the waiting area in the clinic.

BEFORE THE VISIT

Visit 3 Forms

The coordinator should check the Daily Calendar showing clinic visits scheduled for the following day. The Calendar should list the preferred language of each person scheduled for a clinic visit, which will help determine how many sets of each form is needed for the day.

Print a set of the forms that will be completed for interviews and procedures. These will be pre-printed with participant IDs, which will be matched to an individual when he/she begins the exam. Forms can be printed somewhat in advance of upcoming visits, and it is advisable to have enough printed forms on hand for all the participants. Forms will be printed in English, with the Hindi translations available. For each participant, gather all the forms required for a visit, and place into a folder labeled with his/her participant ID.

Supplies and Equipment

- Make sure the accelerometers are ready and completely charged before handing out to the participants. The instructions for the accelerometer should also be printed and laminated.
- Prepare the examination room for the anthropometry measurement, and seated BP.
- Check all instruments that will be used for the examination e.g. Amount estimation tools, phlebotomy supplies.

Staffing

- Prepare staff assignment sheet and make sure everyone knows his/her responsibilities.

Suggested 6 month visit schedule

Participant Arrives	9:00 am
Seated Blood Pressure	09:00 – 09:10 am
Fasting labs	09:10 - 09:15 am
Anthropometry	09:15 – 09:20 am
Snack	09:20 – 09:30 am
Questionnaires	09:30 – 09:50 am
24 hour food recall	09:50 – 10:25 am
Accelerometer usage instructions	10:25 – 10:3011.30 pm

Instructions to Participants Before the Visit

Mail the Pre-Visit Instructions (Appendix 13A) to the participant 5–7 days before 6 month visit and explain to them over the telephone when you schedule the visit. If possible, make a reminder call to the participant the day before 6 month visit and reiterate the instructions. (If the participant is acutely ill—e.g. "flu" or bronchitis—when you make this reminder call, *tell him/her not to come to the clinic.* Arrange to contact him/her again to reschedule when he/she has recovered.) Before the examination, make sure the participants understand the following instructions.

Participants must fast for at least 10 hours before the examination. This restriction applies to all food and beverages (except water), including alcohol. Instruct them to consume dinner at least 10 hours before their scheduled appointment at the clinic. Only water and prescription medications are allowed from dinner until the start of the examination the next morning. Diabetic patients should *not* take their hypoglycemic medications the morning of the clinic visit; they should *bring the morning dose to the clinic* to be taken after venipuncture.

Participants should not smoke on the morning of the visit.

CONFIDENTIAL- Do not distribute, developed by Northwestern University

Participants should bring all current medications, both prescription and over-the-counter, including vitamin preparations and dietary supplements, to the clinic. If the participant forgets to bring the medications, schedule another visit to obtain this information or collect the information over phone.

Participants should bring the name and complete address of their personal physician or health plan, particularly if they wish to have examination results sent to that provider.

Participants should wear or bring loose-fitting clothes, preferably t-shirt, sweat pants, and slip-on shoes or sneakers.

VISIT RECEPTION

The reception process is very important in setting the participant's frame of mind for the rest of the exam day. Greet each participant warmly as soon as he/she arrives at the clinic. (If a participant arrives at the clinic acutely ill—e.g., "flu" or bronchitis—do not continue with the clinic examination. Make arrangements to contact him/her to reschedule the appointment after he/she has recovered.)

.

SEATED BLOOD PRESSURE

Purpose: Blood pressure (BP) level is a major risk factor for coronary heart disease, congestive heart failure, and stroke. Heart rate reflects autonomic nervous system function and cardiovascular fitness. The measured BP level is subject to biological and observer variability.

Only study staff that are trained and certified can take blood pressure measurements for the SAHELI study.

Procedure: Refer to Chapter 8, Blood Pressure for instructions.

PHLEBOTOMY

Purpose: To measure traditional (cholesterol, glucose, fasting sugar) risk factors for cardiovascular disease.

ANTHROPOMETRY

Purpose: To measure body weight, height, abdominal girth and hip girth. The measures of weight and height will be used to calculate body mass index; waist and hip circumferences are other measures of body composition that have been linked to cardiovascular disease risk.

Procedure: Refer to Chapter 6, Anthropometry

QUESTIONNAIRES

Purpose: At Visit 3, participants will be asked to complete 7 interviewer-conducted questionnaires that are listed below.

INTERVIEWER-ADMINISTERED QUESTIONNAIRES TO BE ADMINISTERED IN THE ORDER BELOW:

24 HOUR FOOD RECALL
MEDICATION QUESTIONNAIRE AND INVENTORY
PHYSICAL ACTIVITY
HEART DISEASE QUESTIONS
SOCIAL NETWORK SURVEY

EXERCISE CONFIDENCE SURVEY
COPE SURVEY

13.8.1 24 Hour Food Recall

Purpose: The 24 Hour Food Recall will record the participants' food and beverage intake of the previous day.

Procedure: Refer to Chapter 9, 24 Hour Food Recall

MEDICATIONS QUESTIONNAIRE AND MEDICATIONS INVENTORY

Purpose: At the second visit, participants will be asked to bring all medications that they are currently taking to the clinic, including prescription medications, over-the-counter (OTC) medications, vitamins, herbs and dietary supplements.

Procedure: Follow the directions on the data forms.

Filling out the MEDICATIONS QUESTIONNAIRE

Question 1:

- All prescription medications, over-the-counter (OTC) medications, vitamins, herbs and dietary supplements will be recorded on the MEDICATIONS INVENTORY FORM.
- ***If participant has brought in some, but not all of their medications***, record them on the MEDICATIONS INVENTORY FORM and complete the form when the participant brings in or call you with the remaining medications.
- ***If participant is currently not taking any medications*** do not complete a MEDICATIONS INVENTORY FORM.

Filling out the MEDICATIONS INVENTORY

On each completed MEDICATION INVENTORY, indicate the page number of each form by filling in the corresponding bubble. (For the first form, fill in the "1" bubble, for the second form, fill in the "2" bubble, etc.) The ***total*** number of forms will be recorded on the MEDICATIONS QUESTIONNAIRE.

Participant ID #	Acrostic	Date of Interview	Staff ID #
		Mo / Day / Year	

SAHELI MEDICATION INVENTORY

Visit: Baseline 3 month 6 month

Page ○ 1 ○ 2 ○ 3 ○ 4 ○ 5

7. Record the trade name (or generic name, if necessary). If the name of the medication is longer than the number of data entry boxes on the form, just fill out as much as possible.

8. Indicate the frequency the medication is taken.

1. Name:

Frequency ○ Daily ○ Weekly ○ Monthly ○ As needed

SPECIAL INSTRUCTIONS

Issue	Resolution
Participant took a medication 1 week ago but stopped taking it	• Record the medication
Participant has not brought in all of his/her medications or has forgotten to bring all of them	• Record the medications the Participant has with him/her and ask the participant to call you later with the name of the other medication(s).
Participant is unable to list or name all of his/her herbal remedies and supplements	• Ask the participant why s/he is taking the medication

Physical Activity

Purpose: The Physical Activity questionnaire will measure the level of physical activity across different activity categories (household chores, gardening, dancing, exercise etc) and different intensities (light, moderate, and heavy).

Participant ID #	Acrostic	Date of Interview	Staff ID #
		Mo / Day / Year	

MEDICATION QUESTIONNAIRE

SAHELI BASELINE VISIT

PHYSICAL ACTIVITY

Coordinator Instructions: Please use a black pen and fill in bubbles completely.

Think about the types of activities you did in a typical week in the past month. For each activity, note which of these activities you did in a typical week by filling in the circle for YES or NO. For each item you mark as YES, fill in the circle for the number of DAYS in a typical week you did these activities and the AVERAGE TIME per day in hours <u>and/or</u> minutes you did these activities.

Intensity Levels:
Light → easy effort
Moderate → harder than light but not all-out effort
Heavy → all-out effort

Example:
Conditioning Activities
<u>Moderate Effort:</u>
Low impact aerobics, slow bicycling, rowing, leisurely swimming, health club machines - moderate intensity

	Days/Week	Hours/Day	Minutes/Day
Y N	1 2 3 4 5 6 7	1 2 3 4 5 5+	5 15 30 45
● ○	○ ○ ● ○ ○ ○ ○	● ○ ○ ○ ○ ○	○ ○ ● ○

In this example, the activity was done 3 days per week, 1 hour and 30 minutes per day.

HEART DISEASE QUESTIONS

Purpose: This is designed to understand the existing knowledge and perceptions of heart disease among South Asians. Perceptions are measured using a scale ranging from Strongly agree to Strongly disagree. Knowledge is measured based on the response to questions on heart disease prevention and risk factors for heart disease.

Participant ID #	Acrostic	Date of Interview	Staff ID #
		Mo / Day / Year	

SAHELI HEART DISEASE QUESTIONS

Visit: ○ Baseline 3 Month 6 Month

Coordinator Instructions: Read the question and answers (except for #10 on page 4, write the response given). Circle or fill in the answer given by the participant.
Read these instructions to the participant: The following are some questions about heart disease.

Perceptions

	Strongly agree	*Agree*	*Neither agree nor disagree*	*Disagree*	*Strongly disagree*
1. Asian Indians and Pakistanis are more likely to have heart attacks compared to people from other communities	1	2	3	4	5
2. Regular exercise can reduce my chances of having a heart attack	1	2	3	4	5
3. An older person does not need to exercise	1	2	3	4	5
4. If your heart starts to beat fast while you are exercising, you should stop.	1	2	3	4	5
5. Men need more exercise than women	1	2	3	4	5
6. Eating fruits, vegetables and whole grains will help prevent a heart attack	1	2	3	4	5
7. Eating less fat and salt will help prevent a heart attack	1	2	3	4	5
8. Being overweight increases my chances of having a heart attack	1	2	3	4	5

Participant ID #	Acrostic	Date of Interview (Mo / Day / Year)	Staff ID #
		/ /	

SAHELI HEART DISEASE QUESTIONS

Visit: ○ Baseline 3 Month 6 Month

Knowledge

1. True or False: A person who has high blood sugar or diabetes is more likely to have a heart attack.
2. Major risk factors for heart disease and stroke include:
    1. High cholesterol
    2. Chewing or smoking tobacco (e.g. cigarettes, gutkha, bidi)
    3. Family history of heart disease
    4. All of the above
3. True or False: Everyone's body makes cholesterol.
4. A person with high blood pressure:
    1. Has high cholesterol
    2. Has a higher risk of stroke and heart attack
    3. Has a nervous condition
    4. Has a fast heart beat
5. Which blood pressure level is too high?
    1. 130/80
    2. 140/90
    3. 150/85
    4. Both b & c
6. How much exercise is recommended to keep your heart healthy?
    1. 20 minutes every day
    2. 90 minutes a day once you are in shape
    3. 30 minutes everyday
    4. It depends on your age
7. True or False: Medium or fast exercise keeps your heart strong and prevents a heart attack
8. Which of these people should exercise regularly? SHOW PARTICIPANT PAGE 5

1. 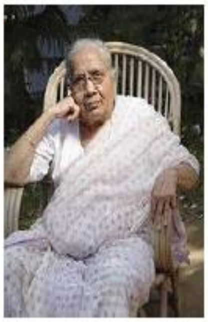
2. 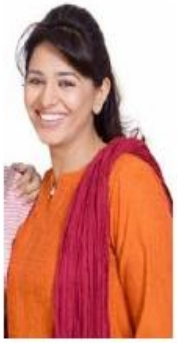
3. 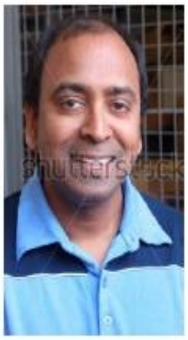
4. All of the above

SOCIAL NETWORK SURVEY

SEE CHAPTER 10 FOR DETAILS

Participant ID #	Acrostic	Date of Interview	Staff ID #
		Mo / Day / Year	

SAHELI SOCIAL NETWORK

Visit: Baseline 3 month 6 month

SAHELI-Net: Social and cultural influences on dietary patterns and physical activity

Overview

The purpose of this interview is to better understand how social relationships affect beliefs, attitudes and behaviors, and how these relationships affect physical and mental health. We will ask about your own and your social network members' health attitudes, beliefs, and behaviors.

As a reminder, your participation in this study is completely voluntary. You may decide to skip questions you prefer not to answer or end the survey at any time. Your decision to participate or not will in no way affect your future treatment or participation in research at this facility. We are very grateful for your assistance with this project.

We hope you will answer as many questions as you can, because your answers will help us to understand how social relationships influence health-related behaviors. This information will be used to design specific programs to directly improve community health.

Do you have any questions before we get started? Is it okay to begin?

Introduction:

"Now we are going to ask you some questions about your relationships with other people. We will begin by identifying some of the people you interact with on a regular basis. You do not have to give me the names of these people, simply their initials."

"From time to time, most people discuss things that are important to them with others. For example, these may include good or bad things that happen to you, problems you are having, or important concerns you may have. Looking back over the last 12 months, who are the people with whom you most often discussed things that were important to you? (**Prompt if do not know:** This could be a person you tend to talk to about things that are important to you. This can include your family, friends, relatives, neighbors, co-workers or anyone else you think you can discuss important matters about yourself.")

Roster A: **Initials of Persons:**

1.	5.	7.	9.
2.	6.	8.	10.

(**Prompt:** "Is there anyone else that you discuss important matters about yourself?")

EXERCISE CONFIDENCE SURVEY

Purpose: Will help understand how sure the participants feel that they will be able to follow the exercise routine.

Participant ID #	Acrostic	Date of Interview	Staff ID #
☐☐☐☐☐	☐☐☐☐	☐☐/☐☐/☐☐☐☐	☐☐☐

SAHELI EXERCISE CONFIDENCE SURVEY

VISIT: BASELINE 3 MONTH 6 MONTH

Below is a list of things people might do while trying to increase or continue regular exercise. We are interested in exercises like running, swimming, brisk walking, bicycle riding, aerobics classes or yoga.

Whether you exercise or not, please rate how confident you are that you could really motivate yourself to do things like these consistently, for at least six months.

Please circle one number for each question.

How sure are you that you can do these things?

	I know I can		*Maybe I can*		*I know I cannot*	*Does not apply*
1. Get up early, even on weekends, to exercise.	1	2	3	4	5	(8)
2. Stick to your exercise program after a long, tiring day at work.	1	2	3	4	5	(8)
3. Exercise even though you are feeling depressed	1	2	3	4	5	(8)
4. Set aside time for a physical activity program; that is, walking, jogging, swimming, biking, or other continuous activities for at least 30 minutes, 3 times per week.	1	2	3	4	5	(8)
5. Find a friend, family member, or group to exercise with.	1	2	3	4	5	(8)
6. Stick to your exercise program when your family is demanding more time from you.	1	2	3	4	5	(8)
7. Stick to your exercise program when you have household chores to attend to.	1	2	3	4	5	(8)

COPE SURVEY

PURPOSE :

The COPE survey helps identify the processes that people use in coping with stressful situations. In this study it will help us understand how the South Asian community copes with stressful encounters of everyday living. In a variety of studies, researchers have used it to investigate the components and determinants of coping.

Participant ID #	Acrostic	Date of Interview	Staff ID #
☐☐☐☐☐	☐☐☐☐	☐☐/☐☐/☐☐☐☐	☐☐☐

SAHELI STUDY

COPE QUESTIONNAIRE

Baseline 3 Month 6 Month

Introduction: There are lots of ways to try to deal with stress. Indicate what you generally do and feel, when you experience stressful events. Please circle the correct response.

	All of the time	*Most of the time*	*Sometimes*	*Rarely*	*Never*	*Does not apply*
1. I try to get advice from someone about what to do.	1	2	3	4	5	(8)
2. I put my trust in God.	1	2	3	4	5	(8)
3. I make a plan of action	1	2	3	4	5	(8)
4. I just give up trying to reach my goal	1	2	3	4	5	(8)
5. I try to come up with a strategy about what to do.	1	2	3	4	5	(8)
6. I look for something good in what is happening.	1	2	3	4	5	(8)
7. I do something I enjoy to take my mind off the stress.	1	2	3	4	5	(8)
8. I ask people who have had similar experiences what they did.	1	2	3	4	5	(8)
9. I try to find comfort in my religion.	1	2	3	4	5	(8)
10. I talk to someone about how I feel.	1	2	3	4	5	(8)
11. I do what has to be done, one step at a time.	1	2	3	4	5	(8)

After the completion of the questionnaires the participants will be given instructions to use the accelerometer. The accelerometers will be loaned and an appointment will be scheduled for the return of the accelerometer.

Participants will be informed that they will receive their test results when they come after one week to return the accelerometer.

See Chapter 8 for accelerometer instructions

REPORTING OF TEST RESULTS WHEN PARTICIPANTS COME TO RETURN THE ACCELEROMETER

Giving Participants Results of Visit Measurements and Lab Values

The participant will be given anthropometry and blood pressure results when they come to return their accelerometers, a week after the 6 month visit. See Appendix 12B
The blood test from quest and physical exam results will be ready in an envelope when they are scheduled to come for their appointment for returning their accelerometer.

ALERTS AND ABNORMAL RESULTS

The purpose of defining medical alerts is to make sure that the participant and his/her physician are aware of any significant medical findings that arise as a result of the SAHELI exam.

Definitions

Alert: Any of the medical findings, listed in Table 1 that may have adverse health consequences to the participant if untreated.

Immediate Referrals: Medical emergencies which require immediate notification of both the participant and his/her primary physician. Participants receiving immediate referrals should be considered as those who would go directly from the Clinical Site to their physician or hospital. Immediate notification of the participant should occur during the clinic visit. Immediate notification of the participant's physician should be accomplished by telephone, to be completed before the participant leaves the clinic. A follow-up letter documenting information discussed by phone should also be sent to the participant's physician for findings included in the initial report, or immediately upon receipt from the central agency for findings on the later reports.

Urgent Referrals: Urgent referrals are made for abnormalities detected which require medical attention but not on an emergency basis. Urgent notification of the participant should occur before the participant leaves the clinic (for findings included in the initial participant report), or immediately upon receipt from the central laboratory (for findings on later reports). Urgent notification of the participant's physician should be sent within the week.

Table 1. SAHELI Alerts and Alert Levels

Finding	Alert Level

Systolic BP > 210	Immediate
Diastolic BP > 120	Immediate
180 < Systolic BP < 211	Urgent
110 < Diastolic BP < 121	Urgent
Total cholesterol > 360 mg/dL	Urgent
Triglyceride > 1000 mg/dL	Urgent
HDL cholesterol < 20 mg/dL	Urgent
Calculated LDL cholesterol > 260 mg/dL	Urgent
Fasting glucose < 50 mg/dL or > 400 mg/dL	Urgent

Methods

4. General Instructions

Whenever one of the alerts listed above is identified for a participant, the actions defined under Immediate or Urgent Referrals, above, must be completed. Actions taken must be documented with a copy of the letter to the participant archived in the participant file.

5. Specific Instructions for Urgent or Immediate Referrals
   - 5.1 If an "Urgent" or "Immediate" Referral result occurs, the Study Coordinator should notify the NU PI as soon as possible.
   - 5.2 The NU PI should telephone the participant within 24 hours and instruct the participant to contact their primary care provider for follow-up immediately.
   - 5.3 The NU PI should document the date and time of the telephone call in the participant's file.
   - 5.4 A letter and a copy of the test should be mailed to the participant. (Appendix 3C)

If any other actions were taken or unusual circumstances were involved, please document this in the participant file.

6. "Other" alerts: If any unusual incidents occur in the clinic, please document this in the participant file. These should include anything that would be important to have documented later, including a participant fall in the clinic, participant feeling dizzy or fainting during the blood draw, etc. If in doubt, document!

6 MONTH VISIT CHECKLIST

Purpose: The 6 MONTH VISIT CHECKLIST provides an outline of all procedures that should be completed during the Visit. After completion of each component of 6 MONTH VISIT, staff will complete the 6 MONTH VISIT CHECKLIST.

The 6 MONTH VISIT CHECKLIST gives a suggested order of procedures for the visit. . It is highly recommended that the interviewer follow the order of the checklist for the remainder of the visit.

Procedure: Complete and check for accuracy and missing information.

Filling out the form:

- Indicate participant status at the top of this form. If the interviewer is unable to contact the candidate to complete Visit 3, the interviewer must use his/her judgment to decide the appropriate time to indicate participant status as "Visit 3 scheduled but did not show."

- If a participant completes the visit, make sure all procedures on the checklist were marked "Yes." *Do not leave any fields blank.*

- If the participant becomes ineligible at any point during the visit, check off which procedures were fully or partially done by marking "Yes." Mark "No, other" for the rest of the incomplete procedures. *Do not leave any fields blank.*

Participant ID #	Acrostic	Date of Interview	Staff ID #
		___/___/_____ Mo Day Year	

SAHELI VISIT CHECKLIST

BASELINE 3 MONTH 6 MONTH

Coordinator Instructions: For checklist items, mark only one bubble per line. *Do not leave any lines blank.* Items in **bold** are teleforms therefore all pages must be faxed in if procedure was marked as done.

Participant Status from Visit 1:

O Visit completed
O Visit not completed, specify reason ______________________________
O Visit scheduled but did not show

PARTICIPANT ID# ☐☐☐☐☐☐

Item	Please mark if done			Comments
	Yes	No	Not applicable	
**PRIOR TO THIS VISIT**				
1. Print forms with Participant ID & Acrostic	O	O	O	
**AT THE VISIT**				
2. Review and sign Informed Consent	O	O	O	
3. Review and sign HIPAA	O	O	O	
4. Review Participant Contact Information	O	O	O	
5. Demographics	O	O	O	
6. Urine pregnancy test (if applicable)	O	O	O	
7. Medication Questionnaire	O	O	O	
8. Medication Inventory Form(s) (if applicable)	O	O	O	
9. Seated Blood Pressure	O	O	O	
10. Urine/Phlebotomy	O	O	O	
11. Fasting Blood Draw	O	O	O	Time: ___ : ___ AM/PM*<br>Enter draw time on the Quest Lab Form.

APPENDIX 14A: APPOINTMENT LETTER

South Asian Heart Disease Prevention Study
Instructions for the visit

CLINIC APPOINTMENT REMINDER
Appointment Date: 01/07/2010
Time: 8:15 am

Begin Fasting Date: 01/08/2010
Time: 8:15 pm
(10 hrs before appointment)

Date:

Dear Mr/Ms.__________,

Thank you very much for agreeing to participate in the "Translating a heart disease lifestyle intervention into the community" study. You visit is scheduled at Metropolitan Asian Family Services on ____________, **Date** ________ at **time** _____. Directions from your residence to the facility are attached.

Below are some items to keep in mind prior to your visit:

1. You will have a fasting blood test during your visit. **Do not eat anything after 10 p.m. the day before your visit.** You will not be eligible for blood test if you do not fast.
2. If you take diabetes medicine, **do not take your diabetes medicine the morning of your visit.**
3. Take all other medicine.
4. We ask that you **bring all current medications to the visit.**
5. If you have a doctor and you want your results sent to your doctor, bring your doctors information.
6. Wear comfortable cloths.
7. If you need glasses for reading, please bring them with you.
8. If you are ill, please call us at (312) 503-6995 to reschedule your visit.

CONFIDENTIAL- Do not distribute, developed by Northwestern University

APPENDIX 14B : EXAMINATION RESULTS FORM

The South Asian Heart Disease Prevention Study

Physical Exam and Blood test Results

Date:________ ID # ____________________

Dear ___________________

We appreciate your participation in The South Asian Heart Lifestyle Intervention Study. The purpose of this letter is to provide you with some of the results of your examination.

HEIGHT: __________**feet**________**inches**

WEIGHT: _________**pounds**

BODY MASS INDEX: ___________**kg/m^2**
A body mass index of 25 kg/m^2 or greater is considered overweight, and 30 kg/m^2 or greater is considered obese by the National Institutes of Health.

WAIST:____________inches
Normal waist measurement for men < 35 inches
Normal waist measurement for women < 31 inches

BLOOD PRESSURE: ___________**mmHg**

These values indicate that your blood pressure is:
- □ Normal (SBP < 120 and DBP < 80)
- □ Borderline Elevated (SBP 120-139 or DBP 80-90)
- □ Elevated (SBP > 140 or DBP > 90)

BLOOD TEST RESULTS: (See Quest lab results enclosed)
Some of your blood test results that were out of normal range were:

1. xxx
2. xxx

We recommend that you discuss any abnormal results with your doctor.
Sincerely,

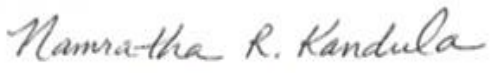

Namratha Kandula, MD, MPH
Principal Investigator
Northwestern University

CHAPTER 15: INDIVIDUAL TELEPHONE SUPPORT

INTRODUCTION

The purpose of the telephone support calls is to keep participants motivated to develop and achieve their goals to prevent a heart attack using the skills they learned throughout the sessions. Phone counseling will use a motivational interviewing framework to focus on self-reflection, behavior goals, and problem solving. Calls follow a semi-structured script, last about 15 minutes, and will be systematically tracked.

Individual telephone support will start two weeks after classes end. Participants will be called biweekly for eight weeks and then one call will be made every four weeks for eight weeks after the biweekly calls. A total of six calls will be made.

Table 1: Telephone support schedule

Weeks	Schedule
1-6	6 sessions (weekly)
7-8	Break
9-16	4 scheduled contacts (biweekly)
17-20	1 scheduled contact (monthly)
21-24	1 scheduled contact (monthly)

Telephone Support Procedure

Phone counseling will be done by the educators. The telephone calls will be audio recorded for research and training purposes. Permission for audio-recording of the telephone calls will be attained during initial visit but will not be mandatory to participate in the study.

The educator will attempt two phone calls for each scheduled telephone support call. If the educator is not able to reach the participant and complete the call during the first attempt, the educator should attempt another phone call 2-3 days later. If the educator is not able to reach the participant and complete the call during the second attempt, the educator should leave a message if possibly letting the participant know they will call them at the next scheduled telephone support call (either in two weeks or in a month) but that the participant can call them back if needed before the next scheduled telephone support call.

If requested, the participants can also receive a combination of text messages and phone calls. This option is being offered to accommodate participants' requests and reduce participant burden. The first scheduled contact will be done via telephone. Two weeks after the first phone call, the educator will send a text message to the participant to see how the participant is doing with their goal. If the participant is not able to accomplish their goal or maintain their goal and needs additional help, the educator will call the participant within 2-3 days to provide support. See diagram on the next page.

If the participant does not respond to the text message after two days, the educator will attempt to reach the participant once via telephone. If the participant does not respond to the telephone phone call, the educator can leave a message saying that they will attempt to contact them at their next scheduled contact (either in two weeks or in a month) but that the participant can call them back if needed before the next scheduled contact.

Diagram 1: Telephone & text messaging procedure

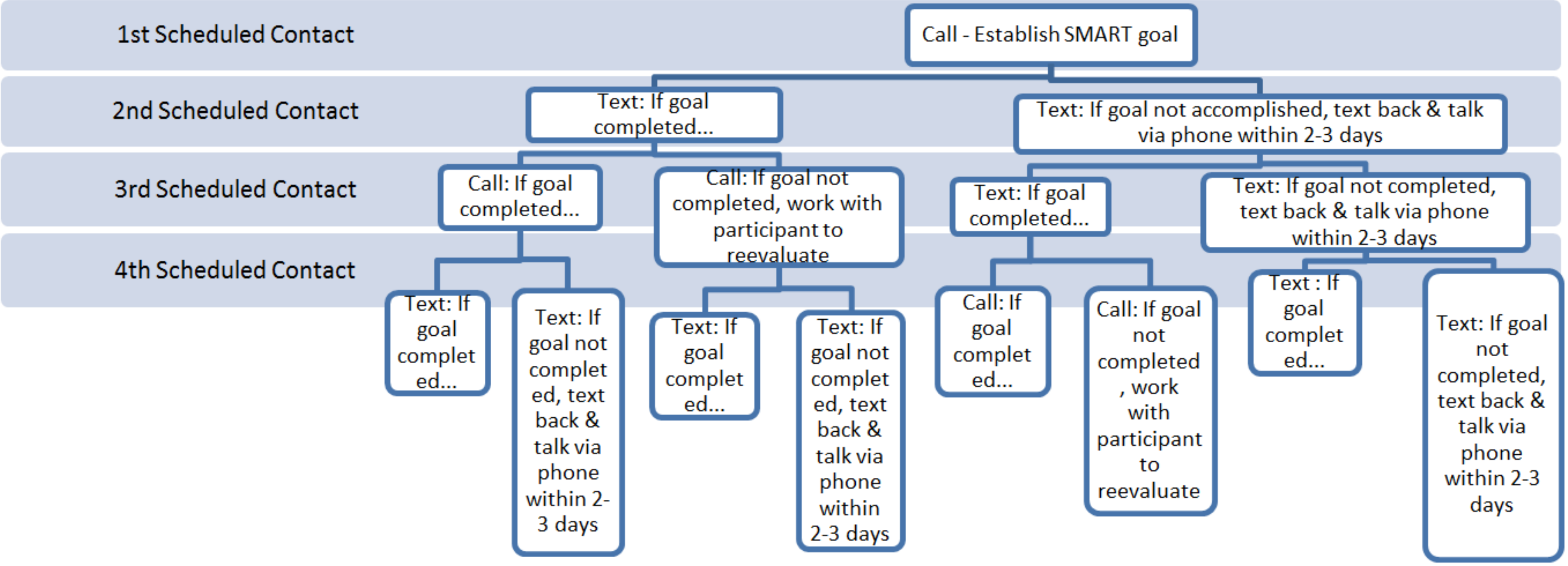

- "Hi _____ this is ________. How are you doing with __(goal)________? Please let us know by responding to this text message in the next two days."

If goal completed:

- "__(provide affirmation)__! Keep up the good work! I will be calling you on _________ to talk more about it."

If goal not completed:

- "__(empathize using reflective listening)__. Can we set up a time to talk in the next 2-3 days?

If a participant notifies the educator that they will be out of town after the six sessions, then the individual telephone support calls will begin 1-2 weeks after that participant returns. If the participant still needs to attend make-up sessions for the classes they missed after the 6th session has been completed, then the individual telephone support calls will start 1-2 weeks after that participant completes all of the make-up sessions.

BRIEF OVERVIEW OF THE PROCESS

Preparation for Telephone Call

- Prepare your work space and eliminate distractions
- Review the participant's information and results and have it in front of you during the call
- Have the South Asian Heart Disease Prevention Lesson Plan Overview in front of you during the call to refer to class content
- Have the Individual Telephone Support Semi-Structured Script in front of you during the call as a guide (see Appendix 15A)
- Have the Telephone Support Call Log to record information (see Appendix 15B)
- Prepare audio recorder

During the Call

- Introduce yourself warmly
- Ask if this is a good time to talk and whether the participant can speak freely
- Use language easy enough for anyone to understand
- Pay attention to the tone of your voice, breathing patterns pauses and speaking pace
- Make an effort to understand in a non-judgmental way and be respectful
- Use open-ended questions
- Allow for thinking with pauses and silences as these may foster more discussion
- Show you're listening by using reflective listening and verbal cues e.g. I see, yes etc.
- Compliment the participant on reaching goals and on acknowledging a need for change through affirmations
- Determine participant importance, confidence and readiness to act on a goal
- Strengthen the participant's self-efficacy
- Avoid giving the participant information to try to convince him/her to change and avoid taking an authoritative stance
- Ask permission to make suggestions if the participant is struggling in developing their goals
- Guide the participant in making a SMART (Specific, Measurable, Achievable, Realistic, Timely) goal

Ending the Call

- Summarize the main points of the conversation once the participant has identified and stated their SMART goal
- Set a time for the next scheduled phone call with the participant
- Thank the participant for taking the time to talk to you

USING MOTIVATIONAL INTERVIEWING DURING PHONE CALLS

Open questions, affirming, reflecting and summarizing (OARS) are the primary skills necessary to practice motivational interviewing. These skills get the participant talking. More importantly, these skills encourage the participant to explore their ambivalence to change and clarify reasons for change. Instead of the educator advocating for change, which often puts the participant in the position of defending against it, motivational interviewing takes a different approach. The idea is to have the educator facilitate the participant's expression of change talk, that is, for the participant to present the arguments for change. The general pattern in motivational interviewing is to ask an open question, setting the topic of exploration, and then follow with reflective listening, affirmations and summarizing. Avoid asking three questions in a row. Reflective listening is not just restating what the participant said, but capturing their feelings about the facilitators and barriers to change.

Eliciting and Responding to Change Talk

Change talk is any participant speech that favors movement in the direction of change. Eliciting change talk is a strategy that gets participants to identify and give voice to the need or reasons for changing. Rather than the educator lecturing or telling participants the importance of and reasons why they should change, change talk comes from the participant.

The educator should be able to identify statements of change talk. Change talk falls into four general categories:

Recognizing disadvantages of the status quo (current situation)

- "I guess there's more of a problem here than I realized."
- "I never really thought that much before about how this affects my family"
- "This is serious"
- "Maybe I have been taking foolish risks"
- "I can see that in the long run, this is going to do me in if I don't make a change."

Recognizing advantages of change

- One thing is that I would have a lot more time ,and it would help financially too
- My boys would like it. They're always after me to quit
- Probably I'd feel a lot better
- At least this would get the courts off my back
- I'd probably be around to enjoy my grandchildren as they grow up

Expressing optimism about change, these statements reflect self-efficacy to make a difference in the problem area

- I think I could probably do it if I decided to
- I'm a fairly stubborn person. If I put my mind to something, I don't let go until it's done.
- I did quit smoking a few years ago. That was tough, and it took a few tries, but I did it.

Expressing intention to change

- I think it's time for me to think about quitting
- I definitely don't want to keep going the way I have been
- I've got to do something
- This isn't what I want for my family. What can I do?
- I don't know how I'm going to do it, but I'm going to get through this.

The educator can also use the following strategies to elicit change talk:

1. Ask evocative questions - Example Open Questions to Evoke Change Talk:
   - Disadvantages of the status quo
     - What worries you about your current situation?
     - What makes you think that you need to do something about your blood pressure?
     - What difficulties or hassles have you had in relation to your drug use?
     - What is there about your drinking that you or other people might see as reasons for concern?
     - How has this stopped you from doing what you want to do in life?
     - What do you think will happen if you don't change anything?
   - Advantages of change
     - How would you like for things to be different?
     - What would be the good things about losing weight?
     - What would you like your life to be like 5 years from now
     - The fact that you've attended the sessions indicates that at least part of you thinks it's time to do something. What are the main reasons you see for making a change?
     - What would be the advantages of making this change?
   - Optimism about change
     - What makes you think that if you decide to make a change, you could do it?
     - What encourages you that you can change if you want to?
     - When else in your life have you made a significant change like this? How did you do it?
     - How confident are you that you can make this change?
     - What personal strengths do you have that will help you succeed?
     - Who could offer you helpful support in making this change?
   - Intention to change
     - What are you thinking about _______ at this point?
     - I can see that you're feeling stuck at the moment. What's going to have to change?
     - What do you think you might do?
     - How important is this to you? How much do you want to do this?
     - What would you be willing to try?
     - Of the options I've mentioned, which one sounds like it fits you best?
     - Never mind the "how" for right now – what do you want to have happen?
     - So what do you intend to do?

2. Use the importance ruler
   - Why are you at a _________ and not a zero? What would it take for you to go from __________to a (higher number)?

3. Exploring the Decisional Balance – this is where the participant lists the pros and cons to their current situation and the change they are thinking about making

4. Elaborating – once a reason for change has been named, it is useful to have the client elaborate on a topic before moving on. It is a way of eliciting further change talk, and it helps reinforce the motivational theme

CONFIDENTIAL- Do not distribute, developed by Northwestern University

- Asking for clarification: In what ways? How much? When?
- Asking for a specific example
- Asking for a description of the last time this occurred
- Asking What else, within the change topic

5. Querying Extremes
   - What concerns you the most about your high blood pressure in the long run?
   - Suppose you continue on as you have been, without changing. What do you imagine are the worst things that might happen to you?
   - How much do you know about what can happen if you have a heart attack, even if you don't see this happening to you?
   - What might be the best results you could imagine if you make a change?
   - If you were completely successful in making the changes you want, how would things be different?

6. Looking Back
   - Do you remember a time when things were going well for you? What has changed?
   - What were things like before you started using drugs? What were you like back then?
   - Tell me about how you two met, and what attracted you to each other back then. What was it like?
   - What are the difference between the Pat of 10 years ago and the Pat of today?

7. Looking Forward
   - If you decide to make a change, what do you hope might be different in the future?
   - How would you like things to turn out for you 10 years from now?
   - I can see that you're feeling really frustrated right now. How would you like things to be different?
   - Suppose you don't make any changes, but just continue as you have been. What do you think your life would be like 10 years from now?
   - Given what has happened so far, what do you expect might be happening 5 years from now if you don't make any changes?

8. Exploring Goals & Values – when the client's highest or most central values and goals are defined, you can ask how the problem you are discussing fits into this picture. The central point here is to explore and develop themes of discrepancy between these important goals or values and the client's current behavior.

When the educator hears change talk, the educator should be particularly interested and attentive. Once a reason for change has been named by the participant, it is important to respond to it. The educator can use the acronym EARS: elaborating, affirming, reflecting, and summarizing to respond to change talk. Ways for asking for elaboration include asking for clarification, asking for a specific example, or using straightforward encouragement to have the participant continue.

- In what ways? When?
- Give me an example.

- Tell me about the last time that happened.
- What else?
- What else have you noticed or wondered about _____?

Use reflections selectively to reinforce change talk and keep the conversation moving in the direction of change. Occasionally a reflection will evoke resistance (often if it is too strong). In this case, the educator can recover with a double sided reflection. Summarizing can also be used selectively. The educator can affirm change talk.

- That's a good point
- That sounds like a good idea
- I think that could work
- I can see how that would concern you

Enhancing Confidence

Self-efficacy is the participant's belief or confidence in one self that he/she has the ability to succeed in reaching a specific goal. Self-efficacy is one of the most important determinants of whether behavioral change takes place, because unless people believe that they can produce desired effects by their actions, they have little incentive to act for behavioral change. The desire to change is the degree of perceived discrepancy between status and goal, between what is happening at present and what one values for the future but people cannot be ready to change until they perceive both that they want to and are able to do so.

Self-efficacy also affects whether people mobilize the motivation and perseverance needed to succeed, and finally their ability to recover from failures and relapses, and how well they continue their behavior changes once their goals have been achieved.

The following strategies can be used to strengthen the participant's self-efficacy:

1. Evocative Questions – these are open questions that are used to evoke confidence talk
   - How might you go about making this change?"
   - What would be a good first step?
   - What obstacles do you foresee, and how might you deal with them?
   - What gives you some confidence that you can do this?

2. Using the confidence ruler
   - How confident are you that you could ___________? On a scale from 0 to 10, where 0 is not at all confident and 10 is extremely confident, where would you say you are?
   - Why are you at a _____ and not a 0? What would it take for you to go from _____ to [a higher number]?

3. Reviewing past successes
   - When in your life have you made up your mind to do something and did it? It might be something new you learned or a habit that you quit, or some other significant change that you made in your life. When have you done something like that?
   - What did the client do that worked? Was there specific preparation for change? Look for personal skills or strengths that might be generalized and applied in the current situation. What did the person do to initiate and maintain change? What obstacles were there, and how did he or she surmount them?

4. Personal strengths and support
   - What is there about you, what strong points do you have that could help you succeed in making this change?
   - When the client identifies a personal strength, ask for elaboration and examples and follow with reflective listening
   - Also explore sources of social support the client has for pursuing change. Are there others on whom he or she could call for support? In what ways? Who else could help with change?

5. Brainstorming – this involves freely generating as many ideas as possible for how a change might be accomplished. This list is generated without critique – all ideas are acceptable, no matter how silly or unrealistic they might seem. It is OK to suggest ideas here, but mostly you should rely on the participant's creativity and what they recall from the classes to generate possibilities. Once a list has been generated, ask the client which ideas on the list seem most promising or acceptable and why.

6. Giving information and advice – The educator should ask for permission to give some ideas:
   - I spoke with a participant who dealt with a similar problem; would it be ok if I share some information with you about how to approach ______?
   - There is one thing we learned in class that I think could help you _____________; is it ok if I share this with you?

7. Reframing – For participants who focus on the failure, the educator should reframe failure in a way that encourages rather than blocks further change attempts. The concept of 'try' can be useful. Explanations of failure as being due to internal, stable factors like inability can be reattributed to external and unstable factors like effort or luck:
   - The time wasn't right.
   - I wasn't quite ready.
   - I was unlucky that time.
   - I didn't try hard enough, or long enough.
   - Maybe next time is my time.

8. Hypothetical change – if the person is struggling with practicalities, it may be helpful to use hypotheticals:
   - Suppose that you did succeed and are looking back on it now: What most likely is it that worked? How did it happen?
   - Suppose that this one big obstacle weren't there. If that obstacle were removed, then how might you go about making this change?
   - Clearly you are feeling very discouraged, even demoralized about this. So use your imagination: if you were to try again, what might be the best way to try?

If the participant faces complex barriers related to limited economical resources, lend hope and continue to enhance confidence using the above-mentioned strategies and affirmations:

- I certainly don't have the answers for you, but I have a lot of confidence that you do, and that working together we can find a way.

Setting a SMART goal

A SMART goal is defined as one that is specific, measurable, achievable, realistic, and timely. Setting small SMART goals that the participant will be able to achieve will help build the participant's self-efficacy. Higher outcome expectations are stronger motivators; however, unrealistic, unattainable outcome expectations may discourage participants to continue. The following questions can be used to help the participants set a SMART goal:

- What would you say your goal is between now and next time we talk?
- How often would you do this to get a good outcome?
- How often would you need to do this?
- How much time do you need to achieve that?
- Will that give you enough time to get started?

If a participant selects a goal that is not realistic, consider using the confidence scale or ask specific questions to determine if the SMART goal may need to be modified.

- What would it take for you to go from _____ to [a higher number]?
- What are some things that could make it challenging for you to accomplish your goal?
- If ______ happened, how would it affect you from accomplishing your goal?

If a participant is having trouble coming up with a SMART goal, ask for permission to give suggestions.

- In class, we learned that we should start slow when doing exercises to avoid injuries. What are your thoughts about reducing the amount of exercise you would like to do to avoid injuries?
- I spoke with another participant who also wanted to work on _________. Would you mind if I share with you her ideas on how to accomplish __________?

Identifying Stages of Change

It can be important for the educator to identify the participant's stage of change and provide support according to the stage in order to minimize resistance to behavior change. See Table 2 for examples. The majority of the participants for this study will be in the contemplation, preparation and action stage mainly because the participants have at least one heart attack risk factor and because they have knowledge from the classes on heart attack prevention and therefore they may be more willing and able to make the appropriate changes. It is important for the educator to know that a participant could alternate between stages and go back and forth. As the phone calls progress, the educator should increase awareness regarding these normal fluctuations in acquiring change.

If the participant reports that they are doing the things that were taught in class, the participant is in the action stage. Provide support and encouragement for these participants, however, it is important to assess if the participant is truly taking action and working towards a goal or if the participant is saying they are taking action for approval. Let the participant talk about the actions taken and their experiences. Ask open questions of specific examples of actions taken if needed to truly determine the actions that they have taken towards change.

Determine if the participant can sustain the changes made. If the participant needs help with maintenance, highlight self-efficacy by reinforcing effort not ability and work with the participant to modify and set a SMART goal. Build on existing achievements when setting a new goal.

Participants that report no need for change are in the pre-contemplation stage. If the participant is at this stage, the educator should explain that the decision is theirs to make changes. The educator should encourage re-evaluation and provide support in gathering additional information or reviewing concepts from the sessions if the participant agrees. The educator should let the participant know that they are available to talk when they are ready.

Table 2

**Application of Stages of Change and Motivational Interviewing Techniques**		
**Stage of change**	**Motivational interviewing techniques**	**Examples**
**Pre-contemplation**: Need for lifestyle change is denied.	• Evaluate participant's stage and clarify decision is theirs to make changes<br>• Ask open-ended questions to encourage re-evaluation<br>• Support information gathering, not action<br>• Offer open door policy when ready	• "It is OK if you aren't ready to change. It's up to you."<br>• "What would have to change for you to be concerned?"<br>• "What additional information about heart attacks would help you?"<br>• "You don't have to do anything now, but when you are ready, we can talk about it."
**Contemplation**: Ambivalent about change; sees pros and cons of change	• Evaluate and acknowledge present stage<br>• Elicit change talk by encouraging evaluation of pros and cons of behavior change to improve health<br>• Identify self-motivating statements the participant can repeat to themselves<br>• Strengthen participant's self-efficacy	• "I see you're thinking about change; that's important."<br>• "What would improve if you became more active?" "What would you lose if you gave up fast food?"<br>• "Yes, you would be a good role model. Anything else? "<br>• "When in the past were you more active? Doing what?"
**Preparation:** Seriously thinking about lifestyle change, laying the ground-work	• Reinforce positive progress<br>• Help identify social support<br>• Verify skills for behavior change and encourage small initial steps	• "Checking out inexpensive walking shoes is great."<br>• "Who can talk this over with you?"<br>• "You already addressed similar issues. What modest steps can you take now?"
**Action**: Practicing new behavior in nutrition and physical activity	• Provide support and encouragement<br>• Bolster self-efficacy for dealing with obstacles	• "You are doing beautifully!"<br>• "Last week you solved a similar problem. How can you apply what you learned to this situation?"
**Maintenance**: Continued commitment to change, sustaining new behavior	• Plan for follow-up support<br>• Reinforce internal rewards<br>• Help the participant identify relapse prevention strategies	• "Who can help you do this?"<br>• "How do you feel when you take better care of yourself?"<br>• "How will you know when you've slipped up?" "What can you do if that happens?"

Adapted from Live Well, Be Well Program Manual. University of California San Francisco and City of Berkeley Division of Public Health

Progressing Well With Goal

If the participant is progressing well with their goals, highlight their self-efficacy:

- "Even when a goal seemed difficult, you were able to follow through".

.

As the participant is explaining their success, find out what is going well and use that to craft affirmation. Give plenty of affirmations for goal directed attempts and actions:

- "You've discovered that doing ___________ helps you feel healthier!"
- "If someone else was just getting started with this, what would you tell them?"
- "What would you tell your 8-weeks-ago-self about this?"

Congratulate the participant on their success but determine if the participant can sustain change by asking:

- "Where does _____________ fit into the future?"
- "What can you do to ensure you maintain this change?"
- "How will you be able to monitor your change?"

Continue to enhance the patient's self-efficacy as this will help increase their ability to recover from failures and relapses to sustain behavior changes once their goals have been achieved.

To see if the participant is interested in tackling another goal, ask:

- "What are your next steps towards improving your health?"
- "What else would you like to work on to improve your health?"

If you can't build motivation for new things, go back to what worked in the past. In this case, discuss whether to attempt to maintain some previous changes rather than continue adding new things.

Difficulty Achieving Goal

If the participant is having difficulty achieving a goal, explore the issue and understand why. Assess barriers by asking open-ended questions like:

- "What has been getting in the way?"
- "Setting a time for _____________ didn't work out. I'd like to get a sense of what was going on", "What do you imagine you would get out of the activity?"
- "I wonder if you're not finding this very helpful or if you're feeling discouraged."

If people are facing a daunting task, their instinct may be to avoid it, so you should work with the participant to break down the task. Re-evaluate and modify the SMART goal with the participant to make sure it is a small and visible goal.

Make an effort to remind the participant about the challenges they have already overcome. Do this through affirmations that focus on goal directed attempts.

- "You seem to be comfortable with removing salt from the table and adding a vegetable to your meals, but on the other hand, you notice your pants have been fitting tighter. I wonder if you might have more success with weight loss by trying something different. What do you think of that?"

Consider using the aforementioned strategies to elicit change talk to re-explore benefits of continuing to try a new skill.

Try to get the participant's agreement to continue trying the skill without forcing it on them:

- “How many times might it be helpful for you to try walking for you to know that you gave it a really good shot?”

Re-evaluate the reason the participant choose the goal:
- “Why are you interested in doing this?”

Managing Session Length

In order to keep the time, use a reflection followed by directive question:

- “That sounds like a tough time. Where you able to use your positive activities/thought records/etc. to keep your mood under control even during this tough time?”
- “I’m noticing the clock and we’ve already at about 10 minutes. I don’t want to take up too much time, as this is supposed to be about doing things on your own time. Anything else?”
- “We have about 3 minutes left – anything else we should talk about?”
- “It sounds like you want to talk about x,y and z but I would like to make sure we also talk about ______________________.”

MOTIVATIONAL INTERVIEWING TREATMENT INTEGRITY

20% of randomly selected support call transcripts will be reviewed for protocol adherence using the Motivational Interviewing Treatment Integrity 3.1.1 (MITI 3.1.1) The MITI will be used to assess how many components of motivational interviewing the educator is using while providing telephone support. It is also a way to provide feedback to the educator on their use of MI skills.

The coder will go through a transcript of the recording once to determine an overall score for five global dimensions: Evocation, Collaboration, Autonomy/Support, Direction and Empathy on a five point Likert scale. Then the coder will review the transcript a second time to count specific behaviors.

Rating Global Dimension

The coder should assume a beginning score of “3” and move up or down from there. It is recommended that the coder average to two decimal points.

1. Evocation – MI evokes from participants that which they already have to help activate their own motivation and resources for change. The educator should convey an understanding that motivation for change or the ability to change resides mostly within the client and this motivation can be brought out by eliciting the participant’s own reasons, arguments and ideas for change.
2. Collaboration – MI is a cooperative and collaborative relationship between the patient and clinician. The educator should demonstrate active collaborative conversation and should behave as if the interview is occurring between two equal partners, both of whom have knowledge that might be useful for the problem under consideration instead of an uneven power of relationship in which the educator is the expert that directs the passive participant what to do.
3. Autonomy/Support – MI acknowledges that it is up to the patient to decide what to do and they have the choice to change or not. The educator should support and actively foster the participant’s perception of choice as opposed to attempting to control the client’s behavior or choices.
4. Direction – The educator should maintain appropriate focus on a specific target behavior or concerns directly tied to it. A high score on this scale does not necessarily reflect better use of MI.
5. Empathy – The educator should demonstrate understanding and should make an effort to comprehend the participant’s perspective and feelings. Empathy should not be confused with

warmth, acceptance, or genuineness. Reflective listening is an important part of this characteristic although this global rating is intended to capture all the efforts that the educator makes to understand the participant's perspective and to show this understanding to the participant.

See MITI 3.1.1 for more information and examples of rating global scores.

Determining behavior codes

The coder will review an utterance and assign one of five behavior codes:

1. Giving Information
2. MI Adherent
3. MI Nonadherent
4. Questions
    a. Closed Question
    b. Open Question
5. Reflections
    a. Simple Reflection
    b. Complex Reflection

An utterance is defined as a complete thought. An utterance ends when one thought is completed and when a new idea is introduce or the participant responds to the interviewer utterance. Each utterance only receives one code and not interviewer utterances will receive behavior codes.

See MITI 3.1.1 for more information and examples on rules for determining utterances and coding behaviors.

The following are summary scores calculated from codes that may be helpful for educators instead of the individual scores themselves:

1. Global Spirit Rating = (Evocation + Collaboration + Autonomy/Support)
2. Percent Complex Reflections = Complex Reflections/Total Reflections
3. Percent Open Questions = Open questions/(Open Questions + Closed Questions)
4. Reflection to Question Ration = Total Reflections/(Open Questions + Closed Questions)
5. Percent MI Adherent = MI Adherent/(MI Adherent + MI Nonadherent)

CONFIDENTIAL- Do not distribute, developed by Northwestern University

APPENDIX 15A: INDIVIDUAL TELEPHONE SUPPORT SEMI-STRUCTURED SCRIPT

Initial Phone Call

Goal:
- Elicit change talk
- Strengthen the participant's self-efficacy
- Guide the participant in setting a SMART goal

1. Introduction

Hi, my name is _____________ and I'm calling from the South Asian Heart Disease Prevention program. May I please speak with _________________?

Hi, we wanted to see how you are doing. Is this a good time to talk? It should only take 15 minutes.

IF NO: *Is there another time I can call you? When would you like me to call you back? Record time on Telephone Support Log. Thank you, I will be calling you back at ___________ around ____________.*

IF YES: *Great! We hope that you enjoyed the sessions and learned a lot. We know that when we learn something new, it can be difficult to actually do it. So that it is why we are calling you, to help you use the things you learned and do them in you day to day life.*

2. Check-in with participant's status

- Review the participant's results to help them understand the importance of setting a heart healthy goal:
  Your results showed that you had high *<u>(blood sugar, cholesterol and/or blood pressure).</u>* *What are some things you have been doing or thinking about doing that you learned in class to help change this?*
- Review Session 1 Activity 1 & 2 if the participant is having a hard time with recalling normal ranges of their results and the heart attack risk factors.

3. Identify the stage of change and provide support according to the stage. Use OARS – Open questions, affirmations, reflective listening, and summarize.

<u>If the participant reports that they are doing the things they were taught in class, work with the participant to continue or build on what they are currently doing.</u>
- Provide support and encouragement
- Probe for new skills learned, if small and realistic goals were made, past success that built self-efficacy, social support received, importance of goal made, advantages and disadvantages to achieving goal:
  I'm glad you were able to use what you learned in the classes. What are some things that helped you do this?
- Determine if the participant can sustain the changes they made:
  What can you do to make sure you continue doing this?

- If the participant would like to continue to work on the goal but modify it (e.g. walk three times a week instead of twice), highlight self-efficacy by reinforcing effort not ability:
  You were able to accomplish ___(goal)___________ by ____(effort given)__________.
- To determine if the participant would like to act on other areas:
  What are your next steps towards improving your health?
  What else would you like to work on to improve your health?

If the participant has tried some things or wants to try some things but expresses difficulty or barriers to prevent heart disease

- Help the participant understand their ambivalence by using reflective listening
- Elicit change talk
- Enhance confidence

If the participant expresses no need for change

- Clarify that the decision is theirs to make changes:
  It is OK if you aren't ready to change. It's up to you.
- Ask open-ended questions to encourage re-evaluation:
  What would have to change for you to think about ____________?
- Offer open door policy when ready:
  You don't have to do anything now but when you are ready, we can talk about it.

4. **Help the participant formulate a SMART (Specific, Measurable, Achievable, Realistic, Timely) goal for the next week based on the conversation.**

5. **Summarize the conversation & ask: *What do you think are your next steps?***

6. **Establish date & time for next phone call in a week**

- *When would be the best time to call you to talk about your goal?*
- Record information on Telephone Support Call Log

END CALL

Adapted from Duffecy J, Mohr DC. Telephone Support Protocol: Coach Manual. Sept 15, 2010.

CONFIDENTIAL- Do not distribute, developed by Northwestern University

Individual Telephone Support Semi-Scripted Guide

Follow Up Phone Calls

Goal:

- Review SMART goal
- Respond to change talk
- Continue to strengthen the participant's self-efficacy
- Modify SMART goal if needed
- Increase awareness regarding normal fluctuations in acquiring change

1. Introduction

Hi this is ______________ calling from the South Asian Heart Disease Prevention program. May I please speak with _____________?
Hi, I'm going to check in with you for 10-15 minutes about how things have been going for you and your progress on your goal(s). Is this a good time to talk?

IF NO: *Is there another time I can call you? When would you like me to call you back?* Record time on Telephone Support Log. *Thank you, I will be calling you back at __________ around __________.*

2. Check-in with participant's status

IF YES: *Great! How is it going? Last week, we decided that working on __________ would be most helpful for you. How did it go last week with your goal?*

3. Review progress on current goal

If goal was NOT achieved

- Determine what positive actions took place and help the participant identify success in taking those actions:
  Was there anything that went well in your attempt to achieve this?
  Even though you didn't achieve the goal, you accomplished ______________ which can help in ______________.
- Give plenty of affirmations for goal directed attempts.
  You did a great job with ______________.
- Assess barriers
  What didn't go so well about this?
- Help participant problem solve to achieve goal
  (E.g. your goal was to ___________ but when you ate a meal at your aunt's house, it was too hard to turn down the fried food she offered. Would it be ok if we discussed some ways to politely turn down unhealthy food?)
- Focus on pros to change
  (E.g. you mentioned last week over the phone that you felt this change would help improve not only your health, but your family's health. What are some other things that would improve if you accomplished this goal?)

Adapted from Duffecy J, Mohr DC. Telephone Support Protocol: Coach Manual. Sept 15, 2010.

- Ask them what they learned from both successes and failures to help them understand that they have learned and changed already and to help the participant realize that change takes time.
  What changed about you after attending the classes and setting goals?
  If someone else was just getting started with this, what would you tell them?
  What would you tell your 8-weeks-ago-self about this?

If goal was achieved

- Provide support and encouragement
  That's great! You are on the right path!
- Highlight self-efficacy by reinforcing effort and positive progress not ability:
  You were able to accomplish____(goal)___________ by ____(effort given)________________.

4. Modify or extend on SMART goal

If goal was NOT achieved

- Discuss whether the experiences of the week suggest that modifying the goal would be most helpful:
  Your goal was to ___________ but __(barrier)__________ made it out of the question.
  How would you approach it differently if you keep this goal for next week?
  Does it make sense to consider making a different goal for next week?
- Bolster self-efficacy for dealing with obstacles:
  Last week you solved a similar problem. How can you apply what you learned to this situation?
  You already addressed similar issues. What small steps can you take now?

If goal was achieved

- Build on goals:
  (E.g. your goal from last week was to learn some new, healthy ways to cook vegetables. And although you didn't eat as many vegetables as you would have liked, you did a great job of meeting your goal by learning a healthy recipe for spinach from your sister!)
  In setting your goals for this week, what would be a way to build on the things you've already achieved?
- Decide if the participant would like to act on other areas:
  What are your next steps towards improving your health?
  What else would you like to work on to improve your health?

5. After deciding on a SMART goal, help the participant consider the benefits of their goal

- *What would be the advantages of making this change?*
- *How might your life improve if you achieve this goal?*

6. Leave a few minutes for the participant to ask questions

- *Is there anything else I can be helpful with?*

7. **Summarize the conversation & ask: *What do you think are your next steps?***

8. **Establish date & time for next phone call in a week**

- *When would be the best time to call you to talk about your goal?*
- Record information on Telephone Support Call Log

END CALL

Individual Telephone Support Semi-Scripted Guide

Final Session

Goal:
- Determine ideas for continued commitment to change and for sustaining new behaviors
- Finalize awareness regarding normal fluctuations in acquiring change

1. Introduction

Hi this is ______________ calling from the South Asian Heart Disease Prevention program. May I please speak with _____________?
Hi, I'm going to check in with you for 10-15 minutes about how things have been going for you and your progress on your goals. Is this a good time to talk?

IF NO: *Is there another time I can call you? When would you like me to call you back?* Record time on Telephone Support Log. *Thank you, I will be calling you back at _________ around __________.*

2. Check-in with participant's status

IF YES: *Great! How is it going? Last time, we decided that working on _________ would be most helpful for you. Were you able to achieve this last week?*

3. Review progress on current goal

<u>If goal was NOT achieved</u>
- Encourage participant's assessment and problem solving and give plenty of affirmations for goal directed attempts. Reassess goal.

<u>If goal was achieved</u>
- Highlight self-efficacy by reinforcing effort not ability.

4. Modify or extend on SMART goal

- *Although this is our last phone call, we can still set a goal together.* Encourage the participant to set a SMART goal.

5. Help identify maintenance skills
- *How would you be able to assess whether or not you accomplished your goal?*
- *What are some ways you can monitor your progress? Who can help you do this?*
- *Who can talk this over with you?*
- *Are there others on whom he or she could call for support? In what ways?*
- *Who else could help with change?*
- *How do you feel when you take better care of yourself?*
- *How will you know when you've slipped up? What can you do if that happens?*

Adapted from Duffecy J, Mohr DC. Telephone Support Protocol: Coach Manual. Sept 15, 2010.

CONFIDENTIAL- Do not distribute, developed by Northwestern University

6. **Review what they have accomplished over the phone**
    - *What has been most helpful?*
    - *How can you integrate it into your life?*
    - *Where will you go from here?*
    - *How will you continue to use these skills?*
    - *What are some reasons it make sense to continue with the new skills you've learned?*

7. **Summarize the conversation & ask: *What do you think are your next steps?***

    - *Is there anything else I can be helpful with?*

8. **Reinforce that change is a lifelong process and that we are available for questions in the future.**

We want to thank you for participating in the program and for taking the time to talk with us. It is important to remember that change takes time and it doesn't happen overnight so we appreciate the time and effort you put into developing and realizing your goals. We are available for any questions you may have in the future as we know that these changes to improve your health can be a lifelong process. Take care!

END CALL

Adapted from Duffecy J, Mohr DC. Telephone Support Protocol: Coach Manual. Sept 15, 2010.

APPENDIX 15B: INDIVIDUAL TELEPHONE SUPPORT LOG

Participant ID #	Acrostic	Staff ID #

SAHELI STUDY
TELEPHONE SUPPORT CALL LOG

Individual telephone support will start two weeks after classes end. A total of six calls will be made.

Weeks	Schedule
1-6	6 sessions (weekly)
7-8	Break
9-16	4 phone calls (biweekly)
17-20	1 phone call
21-24	1 phone call

Telephone number (Check preferred number they would like to be reached)
- O Home: ___________________________
- O Mobile: ___________________________

Best times to call (Check and circle all that apply)
- O Mornings
- O Afternoons
- O Evenings
- O Weekdays: Mon Tues Wed Thurs Fri
- O Weekends: Sat Sun

	Scheduled Date & Time	Call or Text	Actual Date & Time	*For text messages only – talked via phone when goal not completed (Y or N)
1				
2				
3				
4				
5				
6				

Page 1 of 21 Version: 4/2/2013

Participant ID #	Acrostic	Staff ID #
☐☐☐☐☐	☐☐☐☐	☐☐☐

SAHELI STUDY
TELEPHONE SUPPORT CALL LOG

Phone call # _____ Date: _______________

	No Change	Change
Benefits (Pros)		
Costs (Cons)		

<u>Things they are currently doing</u>

<u>Things need to improve (circle goal they would like to work on)</u>

Page 2 of 3 Version: 11/20/2012

CONFIDENTIAL- Do not distribute, developed by Northwestern University

Participant ID #	Acrostic	Staff ID #

SAHELI STUDY
TELEPHONE SUPPORT CALL LOG

Phone call # _____ Date: _______________

Importance

Confidence

Barriers

APPENDIX 15C: REFERENCES

1. Miller, William R & Rollnick, Stephen. Motivational Interviewing: Preparing People for Change, 2nd Edition. The Guilford Press, 2002.
2. WIC Works Resource System. WIC Program Nutrition Education Guidance. http://www.nal.usda.gov/wicworks/Learning_Center/ntredguidance.pdf. January 2006
3. Hongu N et al. Behavior Change Strategies for Successful Long-Term Weight Loss: Focusing on Dietary and Physical Activity Adherence, Not Weight Loss. Journal of Extension. February 2011.
4. Manuel JK, Moyers TB. Introduction to Motivational Interviewing Training Manual: Developed for the TEAM Project. http://casaa.unm.edu/download/AF_MImanual.pdf
5. Moyers TB et al. Revised Global Scales: Motivational Interviewing Treatment Integrity (MITI 3.1.1) http://casaa.unm.edu/download/MITI3_1.pdf . January 2010
6. Duffecy J, Mohr DC. Telephone Support Protocol: Coach Manual. September 2010. http://cbits.northwestern.edu/
7. Rollnick S. Motivational Interviewing in Health Care. New York: Guilford Publications, Inc. 2007.
8. Delgadillo AT, Grossman M, Santoyo-Olsson J, Gallegos-Jackson E, Kanaya AM, and Stewart AL. Description of a community-based lifestyle program for lower-income, minority adults at risk for diabetes. Diabetes Educ, 2010, Jul-Aug 2010;36(4):640-650.
9. Heath C, Heath D. Switch: How to Change Things When Change is Hard. Broadway Books 2010.

CHAPTER 16: LAB PROCEDURES

OVERVIEW

The blood sample will be drawn at the baseline, 3 month and 6 month visit after 10 or more hours of fasting.

EQUIPMENT AND SUPPLIES

Quest diagnostics will provide the following supplies in bulk by emailing Drew Kelley.
His contact information is

Andrew Kelly
Internal Account Representative | 1355 Mittel Blvd. | Wood Dale, IL 60191 USA
Phone +1.630.475.4989
Fax +1.610.271.3495
Andrew.M.Kelly@QuestDiagnostics.com

SAHELI study Quest Account number is **22677739**

The supplies that can be ordered from Quest are

- Blood collection tubes:
  - 4 mL EDTA plasma tubes (BD 367862 or 367844)
  - 7 mL gel-topped serum tubes (BD 367977 or 367812)
- Tourniquets (quick-release tourniquet is recommended; please do not use blood pressure cuff)
- Vacutainer barrels
- Alcohol prep pads

MAFS blood collection area should have the following supplies:

- Lab coats and gloves
- Phlebotomy chair
- Plastic cart with wheels (or plastic tray with compartments) for supplies
- Blood tube rack
- 21 gauge Butterfly needles with luer adapter (BD #7251)
- Participant ID labels (already labeled on appropriate tubes)
- Pens
- Band-Aids
- Gauze (2x2-inch)
- Blood spill kit
- Biohazards waste container (for needles/sharps)
- 10% bleach solution or approved biohazard disinfectant

PROCEDURES

Safety Issues and Precautions for Handling Blood and Urine Specimens

In accordance with the OSHA regulations on bloodborne pathogens recommends the following laboratory safety protocol for the sites:

- Use of non-permeable lab coats, nitrile or latex gloves, and face shields when handling any blood in any situation where splashes, spray, spatter, or droplets of blood may be generated and eye, nose, or mouth contamination can be reasonably anticipated.
- Use of aerosol containers in all centrifuges.
- Follow 'Universal Precautions' when handling any blood and urine products.
- Immediately place contaminated needles and sharps in a puncture-resistant, leak-proof container.
- Never recap or break needles.
- Offer Hepatitis B vaccine to all unvaccinated technicians who handle blood. Documentation of vaccination, or technician's refusal to be vaccinated, should be kept on file at the Clinical Site.

Participant ID Labels

Blood and urine samples must be correctly labeled throughout the collection and processing stages.
The label number for the blood samples will be assigned by the participant database. Please use the label number which contains participant id number, acrostic and date of blood draw and enter the number on the test tubes as IDs. This number also needs to be entered on the Quest requisition form.

16.3.3 Forms: see section 20.8 below for all details for completion of these forms.

- Quest lab form: This is a lab test requisition form that needs to be ordered from the respective Quest Lab and be pre-printed with the account information and labels at bottom.

COLLECTION, PROCESSING AND SHIPPING OF URINE SAMPLES

16.4.1 Processing of Urine Pregnancy Samples

Purpose: Collect urine from all female participants with child-bearing potential to ensure that they are not pregnant before they undergo the CT scan. The results of these urine pregnancy tests will be recorded on the Visit 1 Checklist teleform. Women who are post-menopausal or those who have had a hysterectomy or bilateral oophorectomy or tubal ligation do not need to have a pregnancy test.

Supplies:
- First Response Early Pregnancy Test™ (Church & Dwight Co., Inc).

Urine Pregnancy Test Processing
- Use approximately 5 ml of the collected urine sample for the pregnancy test.
- Remove the test stick from the foil wrapper and take off the Overcap.
- Immerse the entire Absorbent Tip in the urine for <u>5 seconds only.</u>
- With the Absorbent Tip still pointing downward, replace the Overcap* and lay the stick on a flat surface with the Result Window facing up.
  *Replacement of the Overcap is not necessary for the proper test function.
- You will see a pink color moving across the Result Window to indicate the test is working.
- Discard any extra urine

- Do not use after the expiration date stamped on the side of the carton. Store unused tests in a dry place below 86°F (30°C). Do not freeze.

Pregnancy Test Interpretation
Result: One pink line is not pregnant; two pink lines is a positive pregnant result.

- Record the test result on the Urine/Phlebotomy form question #5 (positive or negative) and inform the participant of her result.
- Positive result: Any woman with a positive test result cannot have an abdominal or cardiac CT scan and several other procedures (weight, anthropometry, OGTT) for the clinical visit will need to be omitted.
- Negative result: please inform the participant.

16.5 BLOOD COLLECTION

16.5.1 Preparation

Make sure venipuncture supplies are stocked (see EQUIPMENT & SUPPLIES).
Make sure tubes are labeled.

Make sure the phlebotomy room is tidy and stocked with all items needed,

16.5.2 Phlebotomy Room

The blood draw is done in an isolated room, or participants are separated by room dividers. The room is equipped with all of the necessary blood drawing supplies (see EQUIPMENT & SUPPLIES).

16.5.3 Participants

This study depends on and requires the voluntary cooperation of the participants. These people are giving their time – and precious bodily fluids – and their only reward is the knowledge that they are contributing to progress in medicine. Thus, the experience must be as pleasant as possible. Give the participant enough time to feel comfortable, both before and after the blood collection. In many cases the most memorable part of the experience for the participant will be contact with, and the attitude and competence of, the technician who draws the blood. Do not under any circumstances force or coerce the participant to have blood drawn.

16.5.4 Draw Tubes

Correct labeling and accurate tracking of collected specimens is vital, and correct draw tube order is important. Setting up pre-labeled draw tubes in a blood collection tube rack prior to the participant's arrival is recommended.
A maximum of 12 mL of blood will be collected from each participant into 2 draw tubes. There are no additional tubes collected specifically for quality control purposes.

16.5.5 Participant Preparation

We require the participant to abstain from all food and drink (except water and prescription medications) for a minimum of 8 hours prior to clinic visit. Please contact the participant (phone, mail, e-mail) with the following information as close to the visit as possible:

- Do not eat or drink anything but water for at least 8 hours before the visit (but not longer than 18 hours).
- Drink plenty of water.
- Do not drink alcohol for 36 hours before the visit.
- Do not smoke the morning of the visit.
- Do not engage in vigorous physical activity 8 hours prior to the visit.
- Prescription medications may be taken with a sip of water that morning
- Do call to reschedule the appointment if you have been ill during the previous seven days before the visit. Illness is defined as a cold, flu, fever, or any physical stress that in the opinion of the investigator may affect the blood glucose of the participant. Seasonal allergies would not be considered an illness.

The visit may need to be rescheduled if the above requirements are not met (see specific guidelines below).

The following conditions would prohibit the completion of the OGTT:

- Illness within the past 7 days
- Fasted < 8 hours or > 18 hours
- Has not had typical or usual diet over the past 3 days
- Exercised within the past 8 hours
- Smoked morning of test (within 1 hour of test)

ALWAYS WEAR NITRILE OR LATEX GLOVES AND LAB COAT

Blood drawing is standardized for the sitting position. You may have participants clench their fists (moderately) during phlebotomy, for up to two minutes. Venipuncture is performed with a 21-gauge butterfly needle with 12 inches of plastic tubing between the venipuncture site and the blood collection tubes. The butterfly has a small, thin walled needle that minimizes trauma to the skin and vein. Using 12 inches of tubing allows tubes to be changed without any movement of the needle in the vein. Step-by-step procedures are as follows:

1. Arrange draw tubes in order of draw on the table top or in the tube rack within easy reach. Assemble butterfly apparatus and vacutainer holders, gauze, and alcohol prep prior to tourniquet application.

2. Apply tourniquet (quick-release tourniquet is recommended; please do not use a blood pressure cuff).

3. Examine participant's arms for the best site for venipuncture. Release tourniquet.

4. Cleanse venipuncture site by wiping with alcohol prep pad in a circular motion from center to periphery. Allow area to dry.

5. Re-apply tourniquet. (It is best to release the tourniquet as soon as possible after flow has been established. The tightened tourniquet should be on no longer than two minutes; if it is necessary to have it on longer than two minutes, loosen the tourniquet and then re-apply. However, this may result in cessation of blood flow, especially in sick and/or elderly participants, and may result in the need for a second venipuncture.)

6. Grasp the participant's arm firmly, using your thumb to draw the skin taut to anchor the vein. The thumb should be one or two inches below the venipuncture site.

7. With the needle bevel upward, enter the vein in a smooth continuous motion.

8. Make sure the participant's arm is in a flat or downward position while maintaining the tube below the site when the needle is in the vein. It may be helpful to have the participant make a fist with the opposite hand and place it under the elbow for support.

9. Grasp the flange of the vacutainer holder and gently push the tube forward until the butt end of the needle punctures the stopper, exposing the full lumen of the needle. (Minimize turbulence whenever possible. Small steps, such as slanting the vacutainer to have the blood run down the side of the tube instead of shooting all the way to the bottom, may result in significant improvement.)

10. Note the blood flow into the first collection tube. If blood is flowing freely, the butterfly needle can be taped to the participant's arm for the duration of the draw. If the flow rate is very slow, the needle may not be positioned correctly. Try moving the needle slightly without causing discomfort to the participant.

11. Keep a constant, slight forward pressure (in the direction of the needle) on the end of the tube. This prevents release of the shut-off valve and cessation of blood flow. Do not vary pressure or reintroduce pressure after completion of the draw

12. Fill each vacutainer tube as completely as possible (until the vacuum is exhausted and blood flow ceases). If a vacutainer tube fills only partially, remove the tube and attach another of the same type. Plasma tubes less than ½ full are not acceptable. Partially-filled serum tubes are okay but will result in a reduced number of aliquots. If a tube is not completely filled, clearly document on URINE/PHLEBOTOMY FORM.

13. When the blood flow ceases, remove the tube from the vacutainer holder. The shut-off valve re-covers the point and stops blood flow until the next tube is inserted (if necessary).

14. Release tourniquet, if still applied. The ideal tourniquet time is two minutes.

15. To remove the needle, lightly place clean gauze over venipuncture site. Remove the needle quickly and immediately apply pressure to the site with a gauze pad. Have the participant hold the gauze pad firmly for one to two minutes to prevent formation of a hematoma. Discard needle into puncture-proof sharps container.

16. If the participant continues to bleed, apply pressure to the site with a gauze pad. Keep the arm elevated until the bleeding stops. If necessary, tightly wrap a gauze bandage around the pad and leave in place for at least 15 minutes.

17. Clean up the venipuncture area (if necessary). Dispose of needle and tubing in the appropriate biohazard needle sharps containers.

18. Take the filled blood collection tubes to the processing area,

19. The serum tube needs to be set aside for 5 minutes and then centrifuged for 10 minutes

CONFIDENTIAL- Do not distribute, developed by Northwestern University

20. . The EDTA tube going to Quest labs does not need to be processed in any way.

21. Both Quest tubes need to be stored in the refrigerator with the Quest lab slip for the Quest courier to pick up on the same day.

22. Call Quest courier at 1-866-697-8378 to schedule a pick up during normal business hours. Note the confirmation number from Quest.

16.5.6 Venipuncture Difficulties

- Assisting participants who are extremely apprehensive about having blood drawn. Explain to the participant that the blood draw is designed to be as painless as possible. It may help to let the participant go on with another part of the visit and return later for the blood draw. Have the participant relax in the blood drawing chair just so the phlebotomist can check the veins in the participant's arms without actually drawing blood. If the participant has 'good veins', reassuringly say, "Oh, you have good veins; there should be no problem." Do not, under any circumstances force the participant to have blood drawn.

- Procedures for a difficult draw. If a blood sample is not forthcoming, the following manipulations may be helpful:

  If there is a sucking sound, turn needle slightly or lift the holder in an effort to move the bevel edge away from the wall of the vein.

  If no blood appears, move needle slightly in hope of entering the vein. Do not probe. If not successful, release tourniquet and remove needle. A second attempt can be made on the other arm.

  Loosen the tourniquet. It may have been applied too tightly, thereby stopping the blood flow. Reapply the tourniquet loosely. If the tourniquet is a Velcro type, quickly release and press back together. Be sure, however, that the tourniquet remains on for no longer than two minutes at a time.

  Do not attempt a venipuncture more than twice. If possible, have another nurse or phlebotomist try the second attempt.

  Reassure the participants that your inability to obtain a clean venipuncture is not any sign of a medical problem on their part.

- Assisting participants who look or feel faint.

  Have the participant remain in the chair and if necessary sit with their head between their knees until his/her color returns and he/she feels better.

  Provide a basin if the participant feels nauseated.

  Place a cold wet cloth on the back of the neck.

CONFIDENTIAL- Do not distribute, developed by Northwestern University

If the participant faints, use smelling salts to revive by crushing the ampoule and waving it under the nose for a few seconds.

If the person continues to feel ill, contact a medical staff member for advice.

- If all tubes are not collected (blood flow ceases, difficult venipuncture, etc.), make a note of the difficulties on the VISIT CHECKLIST in the Comment section for future reference. If the participant is willing, another attempt should be made to complete the draw, collecting only those tubes that were not filled in the first attempt.

Quest Lab Form

Quest lab requisition forms should be pre-printed with the research account information on top of the form.
Other fields that will need to be filled at each visit include:

1. Patients Last Name: enter CODE (This is the standard research participant last name; DO NOT USE PERSONAL IDENTIFYING INFORMATION)
2. First Name: use Participant ID number-acrostic: for example for participant ID#10099 acrostic ABCF, fill in: 10099-ABCF
3. Sex: M or F
4. DOB: 99/99/99
5. Date of Collection: 99/99/99
6. Time of Collection: XX: XX (Military Time)
7. Fasting Box: Fasting is to be confirmed and box should be checked
8. Ordering Provider: Namratha Kandula
9. FAX results to: 312-503-3350
10. Bill to box: check "Our account" (this information should be pre-printed on top)
11. You do not need to fill in complete address information or insurance information
12. Type of test you are ordering:
    - please check the test boxes for the HbA1c test (#496), the lipid panel (#7600) and the serum glucose test (#483).
13. At the bottom of the Quest lab form, you will need to write in the Participant ID-acrostic (example: 10099-ABCF) on each of the labels that will be fixed to the tubes going to Quest labs (two tubes at most).
14. This Quest lab form needs to accompany the tube(s) of blood going to Quest labs at each visit. Retain the yellow copy of the form for your records.
15. Quest will fax you the results of the blood tests within 1 business day (for the lipids/metabolic panel) and 2 business days for the HbA1c. If you do not receive these results in a timely manner, please call Quest to ask for results.
